# Mild and Functional Group-Tolerant Aerobic *N*-Dealkylation of Tertiary Amines Promoted by Photoredox Catalysis

**DOI:** 10.1021/acs.joc.3c00656

**Published:** 2023-06-09

**Authors:** Ozgur Yilmaz, Marion H. Emmert

**Affiliations:** † Department of Chemistry, Faculty of Sciences, Mersin University, 33343 Mersin, Turkey; ‡ Process Research & Development, MRL, Merck & Co., Inc., 126 E Lincoln Avenue, Rahway, New Jersey 07065, United States

## Abstract

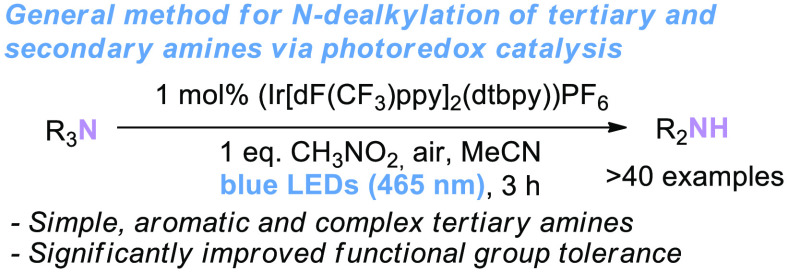

This article describes the development of a mild method for the *N*-dealkylation of tertiary amines via photoredox catalysis and its application in late-stage functionalization. Using the developed method, more than 30 diverse aliphatic, aniline-type, and complex substrates are shown to undergo *N*-dealkylation, providing a method with broader functional group tolerance compared to methods found in the literature. The scope also includes tertiary and secondary amine molecules with complex substructures and drug substrates. Interestingly, α-oxidation to imines was observed in several cyclic substructures instead of *N*-dealkylation, suggesting that imines are relevant reaction intermediates.

## Introduction

*N*-dealkylation reactions represent an important type of biochemical reaction.^1^ Besides their occurrence in DNA repair,^2,3^ the *N*-dealkylation of tertiary amines, and in particular *N*-demethylation, is one of the main reactions in the metabolism of xenobiotics.^2,4^

This metabolic process removes chemicals that contain tertiary amines and are foreign to the body through an oxidative pathway. *N*-dealkylation is commonly catalyzed by monooxygenases such as cytochrome P450^5,6^ and typically proceeds through intermediacy of iron-oxo species.^2,7^

Previous efforts to mimic this metabolic transformation in a reaction flask (e.g., for synthetic access to metabolites) include catalytic reactions presumed to also be proceeding via metal-oxo pathways.^8−10^ However, these methods are typically limited to substrates with aniline or tetrahydroisoquinoline substructures. In addition, several low-valent transition-metal complexes are known to catalyze *N*-dealkylations.^11−14^

**Scheme 1:**
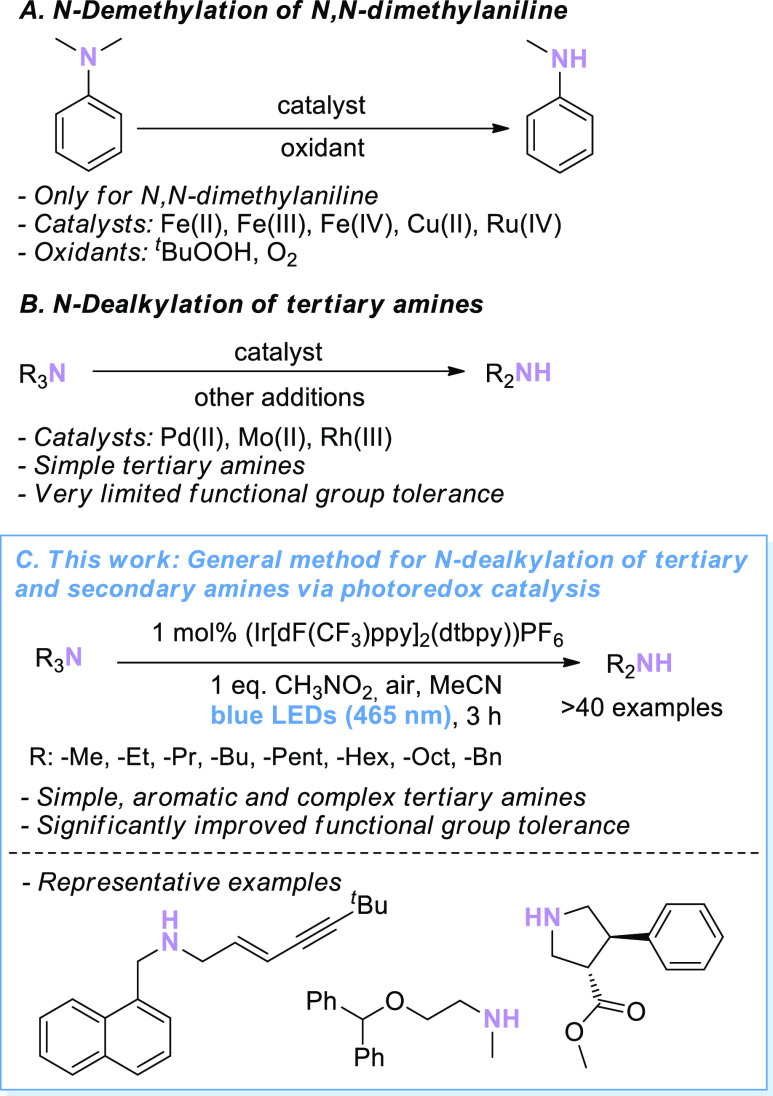
Previous Approaches and Herein Reported Protocol for *N*-Dealkylation of Tertiary Amines

The *N*-demethylation of tertiary amines has been established by several methods, including photochemical transformations^15−19^ as well as Cu,^14^ Ru,^5^ Pd,^11^ and Fe^2,3,7^ catalysis (Scheme 1A,B). However, most of these studies are performed primarily with simple substrates. As such, it is unclear if any of these methods can achieve the *N*-dealkylation of functionalized, complex tertiary anilines or amines or general *N*-dealkylation beyond *N*-demethylation.

Herein, a general method for the *N*-dealkylation of tertiary amines via photoredox catalysis is described. These methods are applicable to various different tertiary amine structures, including aliphatic amines, anilines, and complex amines (Scheme 1C). The established conditions offer a mild and highly functional group-tolerant protocol to achieve general *N*-dealkylations.

## Results and Discussion

### Reaction Optimization

Initial reaction optimizations employed simple NBu_3_ (tri-*n*-butylamine) as a substrate. We reasoned that NBu_3_ would be an ideal test substrate, since it does not contain activating groups (i.e., arenes and double bonds) that could lower the activation barrier for C–H scission. Based on our experience from previous studies on the photoredox-catalyzed C_α_–H cyanation of tertiary amines,^20^ we hypothesized that Ru(bpy)_3_(PF_6_)_2_ would be a suitable photoredox catalyst in combination with blue LEDs (465 nm) as a light source. However, only traces (<5%, Table 1, entry 1) of the desired compound were obtained under these conditions. The yield could not be increased by screening various base or acid additives (see the Supporting Information for details). Using (Ir[dF(CF_3_)ppy]_2_(dtbpy))PF_6_ instead of Ru(bpy)_3_(PF_6_)_2_ as the photoredox catalyst resulted in an increased yield (11%, entry 2). In order to further increase the efficiency of the reaction, we considered that the liberated alkyl group may need to be trapped with a suitable nucleophile. To test this hypothesis, we added CH_3_NO_2_ to the reaction mixture. Gratifyingly, the yield increased to 88% (entry 3) under these conditions. Changing the solvent from MeOH to MeCN resulted in almost quantitative yields of the desired *N*-dealkylation product **2** (94%; entry 4).

**Table 1:**
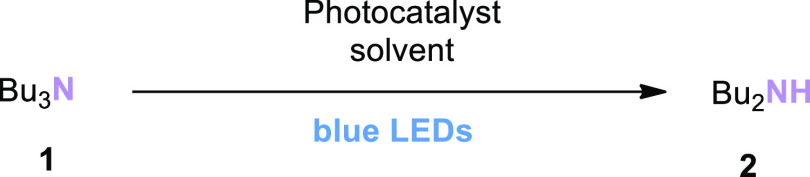
Selected Optimization Studies

entry	changes to conditions^a^	yield^b^
1	2 mol % Ru(bpy)_3_(PF6)_2_, MeOH	<5%
2	4 mol % (Ir[dF(CF_3_)ppy]_2_(dtbpy))PF_6_, MeOH	11%
3	4 mol % [Ir], 1 equiv CH_3_NO_2_, MeOH	88%
4	4 mol % [Ir], 1 equiv CH_3_NO_2_, MeCN	94%
5	1 mol % [Ir], 1 equiv CH_3_NO_2_, MeCN	93%
6	1 mol % [Ir], 0.5 equiv CH_3_NO_2_, MeCN	49%
7	1 mol % [Ir], 1 equiv CH_3_NO_2_, MeCN, 3 h	93%

a Conditions: NBu_3_ (0.27 mmol, 64 μL, 1.0 equiv), 3 mL of solvent (0.09 M), r.t., 24 h, blue LEDs.

b Yields were determined by quantitative, crude ^1^H NMR using 1,3-dinitrobenzene or *p*-xylene as the internal standard or by GC using decane as the internal standard.

Further reaction optimization focused on minimizing the amounts of reagents and catalysts employed. First, the reactions were repeated using 2, 1, and 0.5 mol % Ir catalyst (for details, see the Supporting Information), which afforded the desired product **2** in 94, 93, and 77% yields, respectively. This suggested that a catalyst loading of 1 mol % is sufficient for high yields (Table 1, entry 5). Interestingly, decreasing the amount of CH_3_NO_2_ significantly reduced the yield (49% with 0.5 equiv CH_3_NO_2_; entry 6). Finally, a time study (see the Supporting Information for details) revealed that conversion is complete after 3 h (Entry 7).

### Substrate Scope: Simple Substrates

**Scheme 2:**
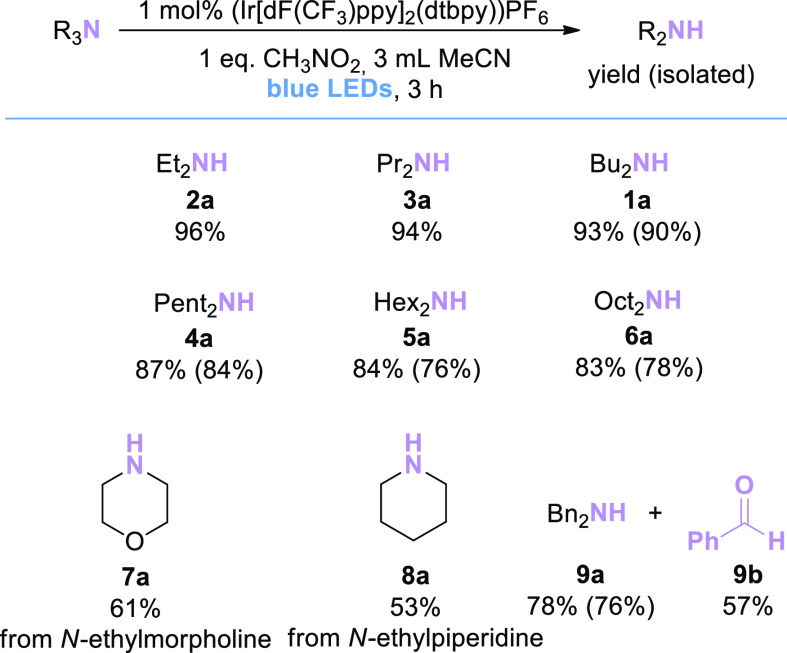
Substrate Scope of Simple Tertiary Amines Assay yields (determined by quantitative ^1^H NMR of the crude reaction mixture or calibrated GC/FID in the presence of an internal standard) are shown outside the brackets; isolated yields are shown in brackets.

After successful optimization of the reaction conditions, we aimed to show that the obtained catalytic system would be useful for the *N*-dealkylation of different alkyl groups. Since most of the methods in the literature only provide data for *N*-dealkylation of aromatic tertiary amines or *N*-demethylation,^2,5−7,15,21,22^ we considered it important to demonstrate that the described method is successful for the *N*-dealkylation of simple tertiary amines. Excitingly, *N*-dealkylation products of non-activated tertiary amines (Scheme 2) were readily obtained: all simple aliphatic amines [NEt_3_, **2**; N(n-Pr)_3_, **3**; N(n-Pent)_3_, **4**; N(n-Hex)_3_, **5**; and N(n-Oct)_3_, **6**] provided similarly high assay yields as obtained for the initial test substrate (96% **2a**, 94% **3a**, 87% **4a**, 84% **5a**, and 83% **6a**).

Piperidine (**8a**) and morpholine (**7a**) are frequently found in the structures of drug active substances or their metabolites. Therefore, the *N*-dealkylation reaction of piperidine or morpholine structures containing *N*-alkyl groups is important for synthesizing the metabolites of drug substances containing these molecules. *N*-dealkylation of the model substrates *N*-ethyl morpholine (**7**) and *N*-ethyl piperidine (**8**) proceeded in 61% and 53% yield, respectively. Although the yields are lower than for other simple tertiary amine substrates, successful *N*-dealkylation of **7** and **8** demonstrates the potential of the method to be applied to more complex substrates. Finally, *N*-dealkylation of tribenzylamine (**9**) to produce dibenzylamine (**9a**; 78% yield) was demonstrated. In this reaction, benzaldehyde (**9b**) was also detected as a byproduct with a yield of 57%; this suggests that hemiaminals or imines are possible intermediates in the reaction pathway.

### Substrate Scope: Aniline-Type Substrates

**Scheme 3:**
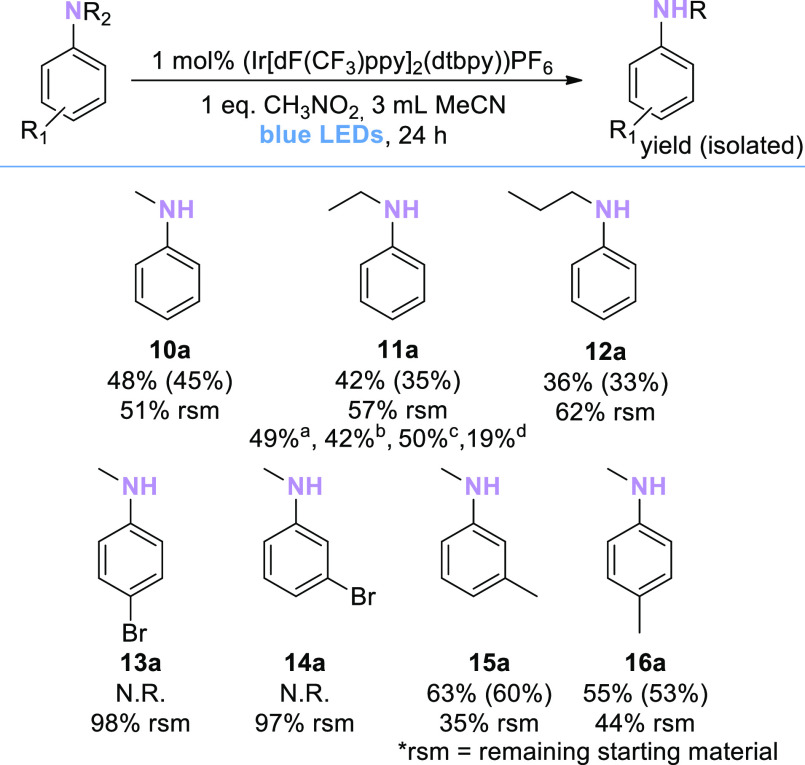
Substrate Scope of Aniline-Type Tertiary Amines Assay yields (determined by quantitative ^1^H NMR of the crude reaction mixture or calibrated GC/FID in the presence of an internal standard) are shown outside the brackets; isolated yields are shown in brackets. ^a^48 h. ^b^2 mol % (Ir[dF(CF_3_)ppy]_2_(dtbpy))PF_6_, ^c^2 equiv CH_3_NO_2_^d^MeOH as the solvent.

We then turned our attention to aniline-type substrates (Scheme 3) in order to compare the developed protocol to other catalytic methodologies in the literature.^2,5−7,15,21,22^ Known protocols typically have been developed using the *N*-demethylation of *N,N*-dimethyl aniline as a model reaction, taking advantage of the activating influence of the aryl substituent. Initial studies underour conditions optimized for NBu_3_ showed only little product formation after 3 h; gratifyingly, extending the reaction time to 24 h produced reasonable yields of all *N*-dealkylation products, with significant amounts of remaining starting materials present in all reactions. Interestingly, para- or meta-bromo substitution of the aniline substructure resulted in complete deactivation of the catalytic system.

As product yields with aniline type substrates were generally lower than the yields obtained with simple tertiary amines, we tested several approaches to increase product yields. Encouraged by the absence of byproducts and the presence of remaining starting materials, we reasoned that longer reaction times, higher catalyst loadings, or higher loadings of CH_3_NO_2_ may further reaction progress. Indeed, when reactions with *N,N*-diethyl aniline (**11**) were repeated by changing the reaction time or CH_3_NO_2_ loading, a slight increase in yield (from 42 to 49% and 50%) was observed. In contrast, increasing the loading of the photoredox catalyst did not increase the yield.

### Substrate Scope: Complex Tertiary Amines and Drug Substrates

**Scheme 4:**
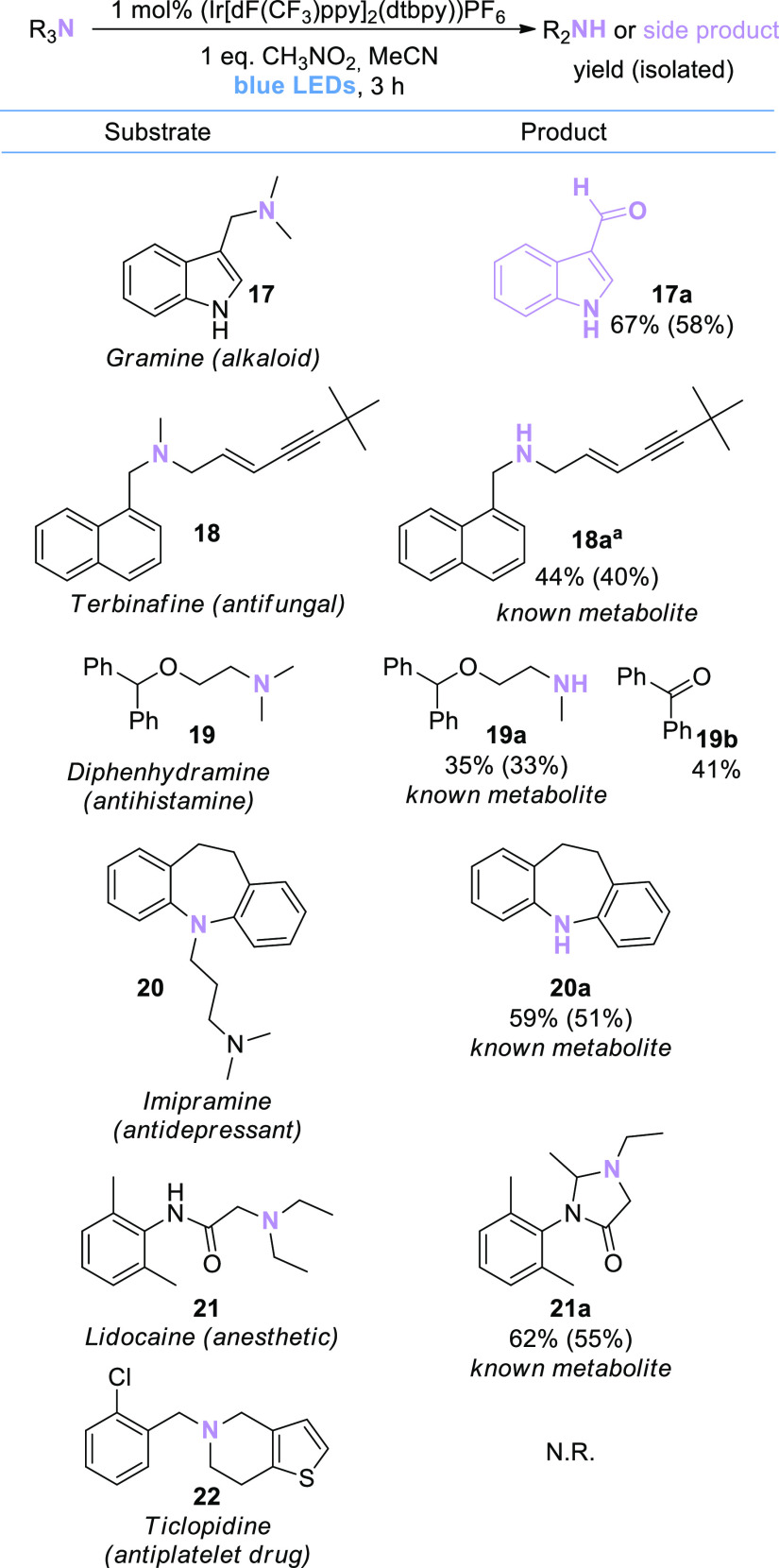
Substrate Scope of Complex Tertiary Amines and Drug Substrates Assay yields (determined by quantitative ^1^H NMR of the crude reaction mixture or calibrated GC/FID in the presence of an internal standard) are shown outside the brackets; isolated yields are shown in brackets.

Due to the mild reaction conditions, we were particularly interested to explore how the developed method would perform with complex substrates. To this end, we chose a series of drug active pharmaceutical ingredients (APIs) (Scheme 4) as test substrates (gramine **17**, terbinafine **18**, diphenhydramine **19**, imipramine **20**, and lidocaine **21**). Gratifyingly, the majority of these more complex structures showed productive reactivity, demonstrating that substructures such as alkenes, alkynes, ethers, amides, and indoles are well tolerated under the reaction conditions.

Importantly, several known metabolites of APIs were obtained (**18a**,^23^**19a,**^24^**20a**,^25^ and **21a**(26)), demonstrating the potential use of the protocol to obtain oxidative metabolites directly from drug APIs via via chemical (as opposed to biochemical) *N*-dealkylation.

**Scheme 5:**
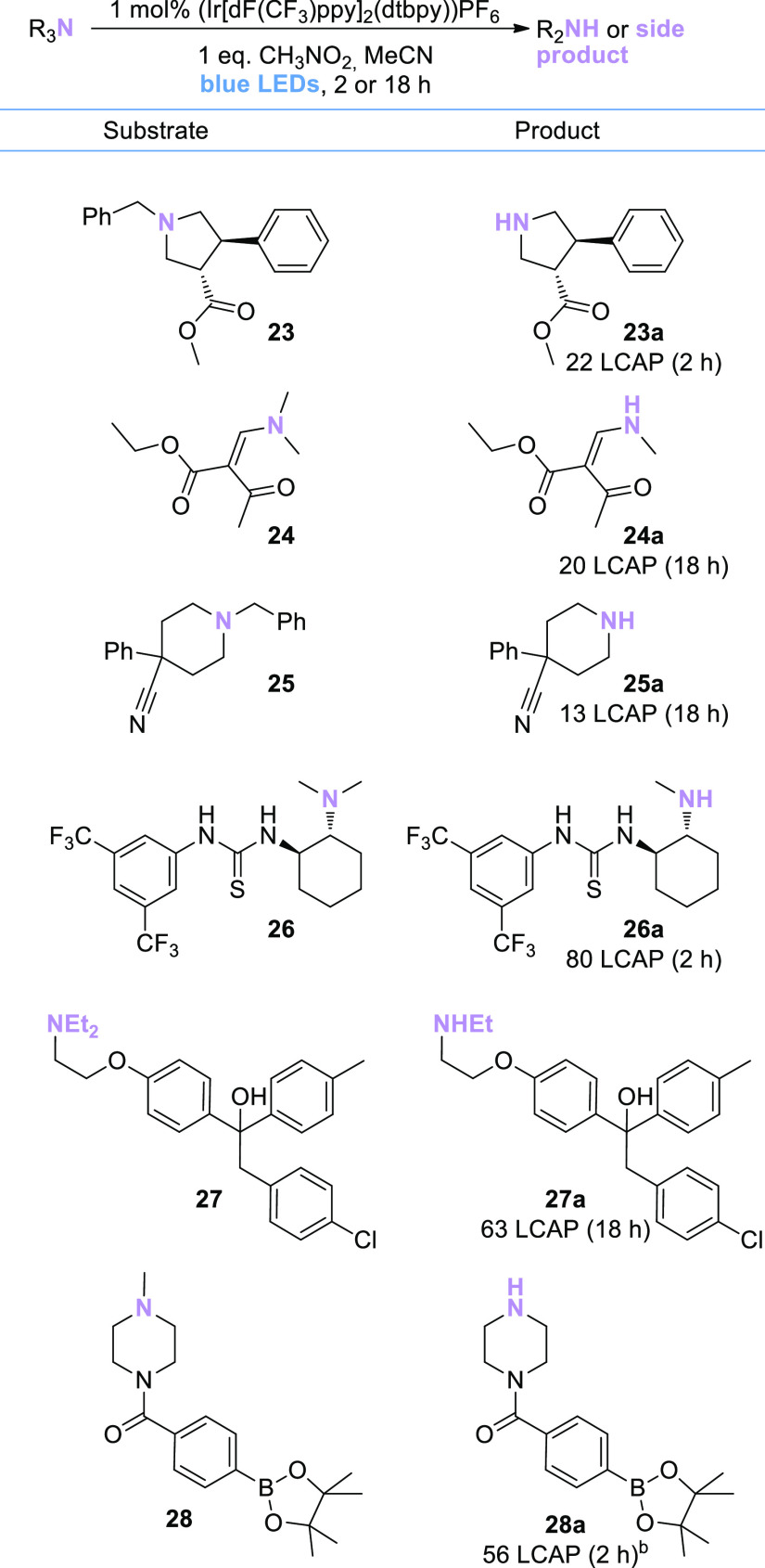
Substrate Scope of Complex Tertiary Amines The liquid chromatography area percent is determined by integration of the UV trace at 210 nm, extracted from UPLC-MS measurements. Structure assignments were confirmed by ESI-MS (for details see the Supporting Information). ^b^With MeOH as solvent instead of MeCN and 2 equiv CH_3_NO_2_.

**Scheme 6:**
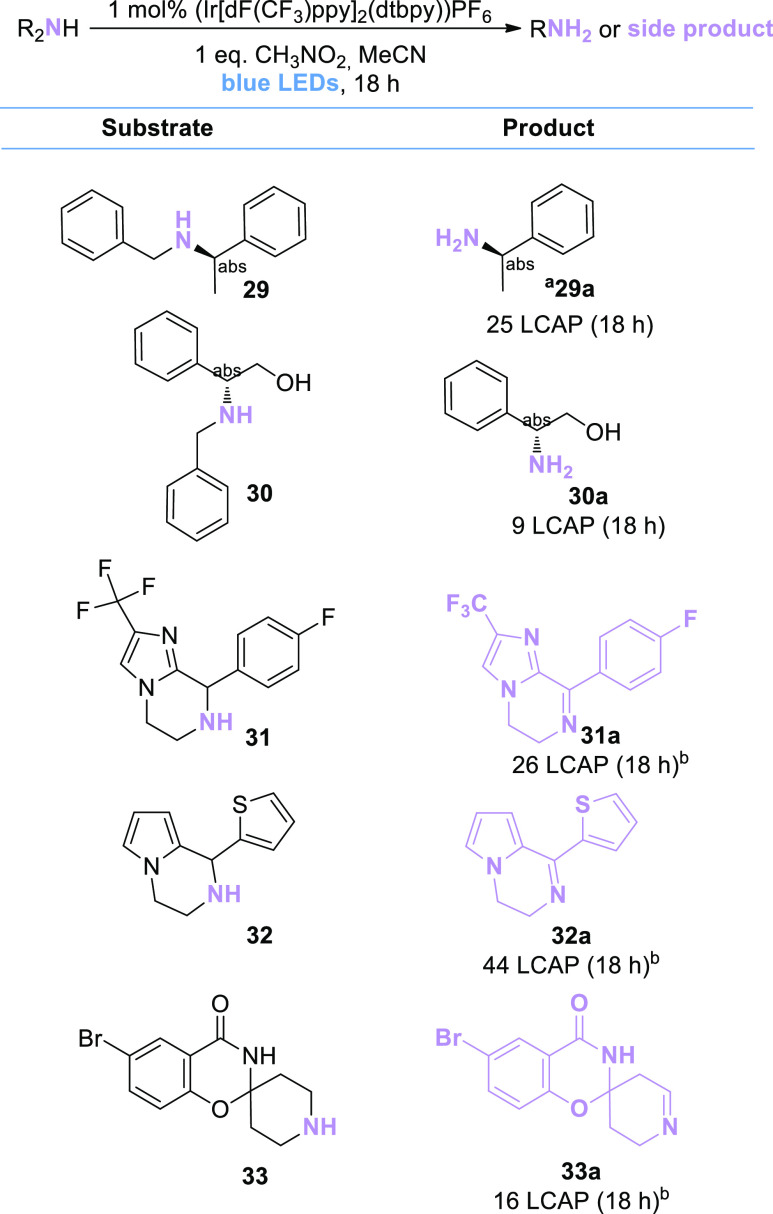
Substrate Scope of Complex Secondary Amines The LCAP is determined by integration of the UV trace at 210 nm extracted from UPLC-MS measurements. Structure assignments were confirmed by ESI-MS (for details see the Supporting Information). ^b^Since structure assignments were assigned by ESI-MS, other isomers of the shown products are possible.

In order to survey a broader scope of pharmaceutically relevant substrates, a series of tertiary and secondary amines with intermediate complexity was obtained from the Merck & Co., Inc., Rahway, NJ, USA building block collection. These substrates were subjected to the optimized reaction conditions and analyzed by UPLC-MS after 2 and 18 h. Selected results are shown as liquid chromatography area percent (LCAP) in Schemes 5 and 6; structural assignments were made based on ESI-MS data (for the complete data set, see the Supporting Information).

Even though most product LCAPs observed in these studies were relatively low, one key observation is the surprisingly broad functional group tolerance that was demonstrated. For example, the presence of an α,β-unsaturated ketone in substrate **24** or an aryl-Bpin moiety in substrate **28** (Scheme 5) did not prevent the desired *N*-dealkylation reactivity. Additional tolerated functional groups of note include esters (**23**, **24**), a thiourea (**26**), tertiary or primary alcohols (**27**, Scheme 5; **30**, Scheme 6), and an aryl chloride moiety (**27**, Scheme 5).

For the set of secondary amines tested, *N*-dealkylation proceeded well for rather simple substrates (**29**, **30**; Scheme 6). However, another reaction other than *N*-dealkylation was observed for cyclic secondary amines: the (likely oxidative) formation of imine products (**31a**, **32a**, and **33a**). Observations of these dehydrogenated products are in agreement with the working hypothesis of imine (or iminium) intermediates in the reaction mechanism (see the next section).

### Background Experiments and the Proposed Mechanism

A series of background experiments were performed to further elucidate the role of each reaction component (Table 2). As expected, no *N*-dealkylation product was obtained in the reaction without the photoredox catalyst (entry 2) or light (entry 6). A low yield (3%) was obtained without CH_3_NO_2_ (entry 3). In combination with the observation that the product yield decreased by ∼50% when using 0.5 equiv of CH_3_NO_2_ instead of 1 equiv (entry 4), these data suggest that CH_3_NO_2_ is involved in driving product formation.

**Table 2:**
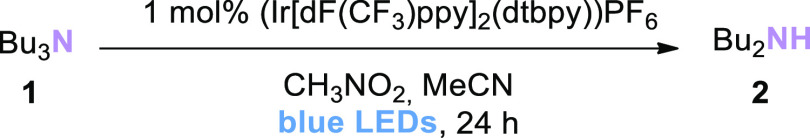
Control Studies for the Proposed Mechanism

entry	conditions	yield^a^
1	1 mol % (Ir[dF(CF_3_)ppy]_2_(dtbpy))PF_6_, 1 equiv CH_3_NO_2_, 24 h	93%
2	No (Ir[dF(CF_3_)ppy]_2_(dtbpy))PF_6_	N.R
3	No CH_3_NO_2_	3%
4	0.5 equiv CH_3_NO_2_	48%
5	50 mol % BHT^b^	37%
6	No light	N.R
7	N_2_ atmosphere (no air)	14%

a Yields were determined by quantitative, crude ^1^H NMR using 1,3-dinitrobenzene or ClH_2_CCHCl_2_ the as internal standard or by GC using decane as the internal standard.

b Butylated hydroxytoluene (2,6-di-tert-butyl-4-methylphenol).

When 50 mol % BHT (butylated hydroxytoluene or 2,6-bis(1,1-dimethylethyl)-4-methylphenol), a radical scavenger, was added to the reaction, the product yield decreased significantly (37%) (entry 5). Furthermore, performing the reaction under nitrogen (entry 7) suggests that oxygen from air is also important for turnover. These data combined suggest that radicals are important intermediates in the reaction mechanism and that the presence of oxygen is required to drive the reactivity.

**Scheme 7:**
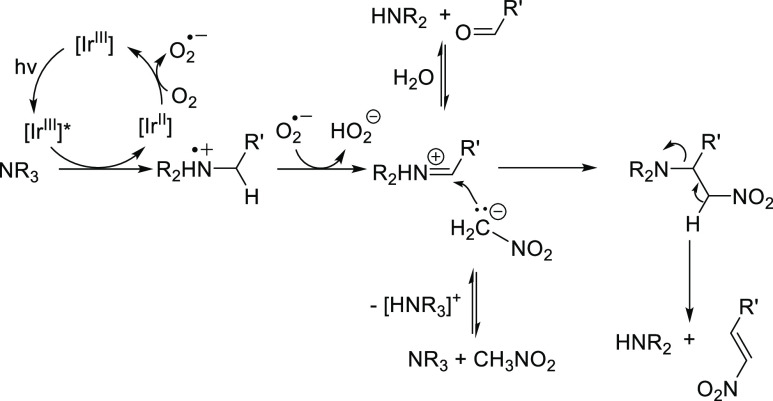
Mechanistic Hypothesis

Taken together, the importance of light, radical intermediates, the photocatalyst, and air suggests that the initial activation of the amine substrates occurs by an aerobically driven, one-electron oxidation of the amine substrate by the excited Ir photocatalyst (Scheme 7). As a result of a second oxidation, an iminium intermediate can then be formed. When traces of water are present, this iminium intermediate can be hydrolyzed; in the case of PhCHO detection in the reaction mixture of Bn_3_N-oxidation (see Scheme 2 above), this hydrolysis may be driven by the stabilization of the aldehyde functionality by conjugation with a Ph moiety. Alternatively, deprotonated nitromethane could nucleophilically attack the iminium intermediate in a Henry-type reaction, leading to the formation of the *N*-alkylated product and a nitroalkene side product. This hypothesis is supported by GCMS analysis of the crude reaction mixture of *N*-dealkylation reactions with N(n-Pr)_3_ (**3**), N(n-Hex)_3_ (**5**) and N(n-Oct)_3_ (**6**) as substrates. In all of these mixtures, the olefin R^ı^-CH=CH–NO_2_ was detected, with the R^1^ group varying according to the length of the alkyl group in the amine structure of the starting material. These results support the mechanism shown in Scheme 7. Furthermore, NMR analysis of a reaction mixture D_3_-MeCN, using tri-*n*-butylamine (**1**) as substrate shows signals indicative of the double bond structure in n-Pr-CH=CH–NO_2_ (see the Supporting Information file for details, page S105).

### Synthesis of Substituted Amides

**Scheme 8:**
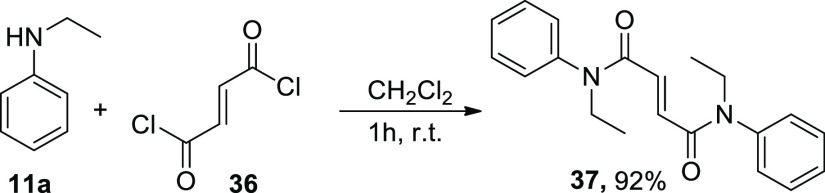
Application for the Dealkylation Product

Mixed secondary amines are generally difficult to synthesize as alkylations of ammonia or amines frequently result in non-selective alkylation,^27,28^ thus requiring the use of protecting groups to achieve synthetically useful, selective alkylation outcomes.^29−33^ Thus, the herein described protocol allows a different approach to obtain and use secondary amines with different substituents, for example, in late-stage functionalization or for the synthesis of amines or amides with three different *N*-substituents. We decided to demonstrate this concept using *N*-ethylaniline (**11a**) obtained by *N*-dealkylation of *N,N*-diethylaniline (**11**) as a nucleophile: reacting **11a** with fumaryl chloride (**36**; Scheme 8) readily produced trans-diamide **37**, a hitherto unknown compound despite its relative simplicity.

## Summary and Conclusions

Overall, this paper describes the development of a mild *N*-dealkylation protocol for simple and complex tertiary and secondary amines. Our studies show that photoredox catalysis and the presence of CH_3_NO_2_ are necessary for efficient reactivity. The value of this methodology is demonstrated by providing access to known metabolites and its applicability in substrates of medium complexity. Even though not all complex substrates employed were successful (see the Supporting Information for details), a broad study of different substructures shows encouraging functional group compatibilities, with highlights including proof of concept with substrates containing primary alcohols, enolizable ketones, and aryl-Bpin structures.

Beyond its direct synthetic impact on late-stage functionalization and metabolite synthesis, the reported method also showcases complementary approaches to accessing secondary amines with mixed substituents, which are otherwise difficult to obtain.

## Experimental Section

### General Information

All chemicals were obtained from commercial suppliers (e.g., Alfa Aesar, TCI America, and Sigma-Aldrich) and used as obtained unless otherwise specified in the procedure. Deuterated solvents used for NMR measurements were obtained from Cambridge Isotopes or Sigma-Aldrich.

The light source is taken from Solid Apollo Company. The ″Blue 5050 72W″ model is a blue-colored LED strip light with a wavelength of 465 nm. This strip was adhered to a glass container, and the vials in which the reactions were carried out were placed in this container (see the Supporting Information file for details). The 4 mL vials used in the reactions were purchased from Chemglass (CG-4904-06).

All NMR experiments were carried out on Bruker BioSpin 500, 400 MHz Avance III Digital NMR spectrometers. All quantitative ^1^H NMR measurements were performed using an adjusted method (15 s relaxation time, NS = 32) with 1,3-dinitrobenzene or *p*-xylene as the internal standard. All NMR spectra were recorded at room temperature unless otherwise noted. The signal for the non-deuterated solvent (δ 7.26 ppm for CDCl_3_) was used as an internal reference for ^1^H NMR spectra. For ^13^C NMR spectra, chemical shifts are reported relative to the solvent resonance of CDCl_3_ at δ 77.0 ppm.

High-resolution mass spectrometry was performed by the MS-service of the National Nanotechnology Research Center (UNAM) at Bilkent University, Turkey. High-resolution mass spectra were recorded by LC–MS TOF electrospray ionization. Masses are reported in *m*/*z* units and the molecule ion as [M + H]^+^ or [M + Na]^+^.

### General Procedure

A tertiary amine (0.27 mmol, 1.0 equiv) was dissolved in 3 mL of MeCN, and then, CH_3_NO_2_ (0.27 mmol, 15 μL, 1.0 equiv) [Ir(dF(CF_3_)ppy)_2_(dtbbpy)]PF_6_ (0.0027 mmol, 0.003 g, 1.0 mol %) was added to the stirred solution under an air atmosphere. The vial was then sealed tightly with a Teflon-lined vial cap, and the mixture was stirred at room temperature under irradiation with blue LEDs for 3 h. Reactions were monitored by thin-layer chromatography (TLC). After the reaction time was completed, the solvent was removed under reduced pressure.

To determine crude assay yields by GC, decane or dodecane was added to the reaction mixture. The mixture was sampled by diluting an aliquot with MeOH, followed by filtration and analysis of the filtrate by gas chromatography–flame ionization detection.

To determine crude assay yields by quantitative ^1^H NMR, the reaction mixture was evaporated. Then, CDCl_3_ and 1,1,2-trichloroethane (3.47 μL, 37.4 μmol; 0.14 equiv) or *p*-xylene (11 μL, 0.27 mmol; 1.0 equiv) as the internal standard were added. The resulting suspension was mixed well and analyzed by quantitative ^1^H NMR.

### Characterization and Spectral Data

#### Dibutylamine (1a)

Purification solvent: EtOAc, light yellow oil, yield 31.3 mg (90%). ^1^H NMR (400 MHz, CDCl_3_): δ 2.60 (t, *J* = 7.2 Hz, 4H), 1.47 (ddd, *J* = 14.4, 8.5, 6.0 Hz, 4H), 1.34 (dq, *J* = 14.3, 7.1 Hz, 4H), 0.92 (t, *J* = 7.3 Hz, 6H) ppm. The spectral data were in agreement with the literature data.^34^

#### Gram-Scale Reaction with Tri-*n*-butylamine (**1**)

NBu_3_ (1 mmol, 237 μL, 1.0 equiv), 12 mL of MeCN, Ir[dF(CF_3_)ppy]_2_(dtbpy))PF_6_ (0.01 mmol, 0.011 g, 0.01 equiv), and CH_3_NO_2_ (1 mmol, 56 μL, 1.0 equiv) were mixed into a 75 mL pressure vessel glass (Chemglass) with a stir bar and internal thread cap. The reaction mixture was stirred for 3 h at room temperature while irradiating with blue LEDs. yield 110.6 mg (86%).

#### Dipentylamine (**4a**)

Purification solvent: EtOAc, colorless oil, yield 35.6 mg (84%). ^1^H NMR (400 MHz, CDCl_3_): δ 2.65–2.58 (m, 4H), 1.57–1.47 (m, 4H), 1.37–1.24 (m, 8H), 0.90 (t, *J* = 7.0 Hz, 6H) ppm. The spectral data were in agreement with the literature data.^35^

#### Dihexylamine (**5a**)

Purification solvent: EtOAc, colorless oil, yield 38.0 mg (76%). ^1^H NMR (400 MHz, CDCl_3_): δ 2.67–2.57 (m, 4H), 1.55–1.47 (m, 4H), 1.36–1.16 (m, *J* = 3.0 Hz, 12H), 0.88 (t, *J* = 6.8 Hz, 6H) ppm. The spectral data were in agreement with the literature data.^34^

#### Dioctylamine (**6a**)

Purification solvent: EtOAc, light yellow oil, yield 50.9 mg (78%). ^1^H NMR (400 MHz, CDCl_3_): δ 2.67–2.57 (m, 4H), 1.55–1.47 (m, 4H), 1.36–1.16 (m, *J* = 3.0 Hz, 20H), 0.88 (t, *J* = 6.8 Hz, 6H) ppm. The spectral data were in agreement with the literature data.^36^

#### Mopholine (**7a**)

^1^H NMR (400 MHz, CDCl_3_): δ 3.72–3.64 (m, 4H), 2.92–2.84 (m, 4H), 1.69 (s, 1H) ppm. The spectral data were in agreement with the literature data.^37^

#### Dibenzylamine (**9a**)

Purification solvent: 1:3 EtOAc/hexane, light yellow oil, yield 40.7 mg (76%). ^1^H NMR (400 MHz, CDCl_3_): δ 7.44–7.18 (m, 10H), 3.78 (s, 4H), 1.61 (s, 1H) ppm. The spectral data were in agreement with the literature data.^34^

#### *N*-Methylaniline (**10a**)

Purification solvent: 1:9 EtOAc/hexane, light yellow oil, yield 12.4 mg (45%). ^1^H NMR (400 MHz, CDCl_3_): δ 7.25 (dd, *J* = 6.4, 2.5 Hz, 2H), 6.76–6.70 (m, 3H), 2.94 (s, 3H) ppm. The spectral data were in agreement with the literature data.^36^

#### *N*-Ethylaniline (**11a**)

Purification solvent: 1:9 EtOAc/hexane, light yellow oil, yield 11.7 mg (35%). ^1^H NMR (400 MHz, CDCl_3_): δ 7.20–7.15 (m, 2H), 6.70 (tt, *J* = 7.4, 1.0 Hz, 1H), 6.61 (dd, *J* = 8.6, 1.0 Hz, 2H), 3.16 (q, *J* = 7.1 Hz, 2H), 1.26 (t, *J* = 7.1 Hz, 3H) ppm. The spectral data were in agreement with the literature data.^38^

#### *N*-Propylaniline (**12a**)

Purification solvent: 1:9 EtOAc/hexane, viscous oil, yield 12.1 mg (33%). ^1^H NMR (400 MHz, CDCl_3_): δ 7.20–7.14 (m, 2H), 6.69 (tt, *J* = 7.4, 1.0 Hz, 1H), 6.61 (dt, *J* = 8.8, 1.7 Hz, 2H), 3.08 (t, *J* = 7.1 Hz, 2H), 1.65 (dq, *J* = 14.6, 7.4 Hz, 2H), 1.00 (t, *J* = 7.4 Hz, 3H) ppm. The spectral data were in agreement with the literature data.^38^

#### *N*,3-Dimethylaniline (**15a**)

Purification solvent: 1:9 EtOAc/hexane, light yellow oil, yield 19.5 mg (60%). ^1^H NMR (400 MHz, CDCl_3_): δ 7.11–7.05 (m, 1H), 6.54 (d, *J* = 7.2 Hz, 1H), 6.47–6.39 (m, 2H), 2.83 (s, 3H), 2.29 (s, 3H) ppm. The spectral data were in agreement with the literature data.^39^

#### *N*,4-Dimethylaniline (**16a**)

Purification solvent: 1:9 EtOAc/hexane, light yellow oil, yield 17.2 mg (53%). ^1^H NMR (400 MHz, CDCl_3_): δ 7.01 (d, *J* = 8.2 Hz, 2H), 6.56 (d, *J* = 8.1 Hz, 2H), 2.82 (s, 3H), 2.24 (s, 3H) ppm. The spectral data were in agreement with the literature data.^39^

#### 1H-Indole-3-carbaldehyde (**17a**)

Purification solvent: 1:1 EtOAc/hexane, viscous oil, yield 22.8 mg (58%). ^1^H NMR (400 MHz, CDCl_3_): δ 10.08 (s, 1H), 8.35–8.30 (m, 1H), 7.85 (d, *J* = 3.1 Hz, 1H), 7.46–7.43 (m, 1H), 7.35–7.31 (m, 2H) ppm. The spectral data were in agreement with the literature data.^40^

#### (*E*)-6,6-Dimethyl-*N*-(naphthalen-1-ylmethyl)hept-2-en-4-yn-1-amine (**18a**)

Purification solvent: 1:11 MeOH/CH2Cl2, viscous oil, yield 30.1 mg (40%). ^1^H NMR (400 MHz, CDCl_3_): δ 7.31–7.25 (m, 5H), 7.17 (dt, *J* = 10.3, 3.4 Hz, 2H), 7.07 (dd, *J* = 6.3, 2.3 Hz, 1H), 6.77 (dd, *J* = 7.4, 6.5 Hz, 1H), 3.98 (dt, *J* = 6.2, 1.7 Hz, 1H), 2.90 (s, 2H), 2.39 (dd, *J* = 5.5, 2.0 Hz, 1H), 1.03 (s, 9H) ppm. The spectral data were in agreement with the literature data.^41^

#### 2-(Benzhydryloxy)-*N*-methylethanamine (**19a**)

Purification solvent: 1:1 EtOAc/hexane, viscous oil, yield 21.5 mg (33%). ^1^H NMR (400 MHz, CDCl_3_): δ 7.42–7.29 (m, 10H), 5.36 (s, 1H), 3.62 (t, *J* = 5.1 Hz, 2H), 2.91 (t, *J* = 5.1 Hz, 2H), 2.48 (s, 3H) ppm. The spectral data were in agreement with the literature data.^42^

#### 10,11-Dihydro-5H-dibenzo[b,f]azepine (**20a**)

Purification solvent: 1:3 EtOAc/hexane, light yellow oil, yield 29.0 mg (51%). ^1^H NMR (500 MHz, CDCl_3_): δ 7.00 (td, *J* = 6.82, 1.57 Hz, 2H), 6.97 (dd, *J* = 7.45, 1.24 Hz, 2H), 6.70 (td, *J* = 7.39, 1.14 Hz, 2H), 6.66 (dd, *J* = 7.94, 1.01 Hz, 2H), 5.91 (br s, 1H), 3.01 (s, 4H) ppm. The spectral data were in agreement with the literature data.^20^

#### 3-(2,6-Dimethylphenyl)-1-ethyl-2-methylimidazolidin-4-one (**21a**)

Purification solvent: EtOAc, yield 34.4 mg (55%). ^1^H NMR (500 MHz, CDCl_3_): δ 7.15–7.04 (m, 3H), 4.39 (qt, *J* = 5.7, 1.5 Hz, 1H), 3.71 (dd, *J* = 14.7, 1.2 Hz, 1H), 3.12 (dd, *J* = 14.6, 1.8 Hz 1H), 2.86 (dq, *J* = 11.7, 7.4 Hz, 1H), 2.40 (dq, *J* = 11.7, 7.0 Hz, 1H), 2.20 (s, 3H), 2.15 (s, 3H), 1.12 (t, *J* = 7.2 Hz, 3H), 1.07 (d, J = 5.7 Hz, 3H) ppm. The spectral data were in agreement with the literature data.^20^

#### *N*^1^,*N*^4^-Diethyl-*N*^1^,*N*^4^-diphenylfumaramide (**37**)

Fumaryl chloride (**36**) (0.13 mmol, 20 mg, 0.5 equiv) was added to a solution of secondary amine **11a** (0.26 mmol, 31.5 mg, 1 equiv) in CH_2_Cl_2_ (3 mL). The resulting solution was stirred at room temperature until the starting material was consumed, as determined by TLC analysis. The solid product that had precipitated was filtered. The solid was washed with CH_2_Cl_2_ and dried under high vacuum to afford amide **37**.

Yield 77.1 mg (92%). ^1^H NMR (400 MHz): δ 7.46–7.39 (m, 1H), 7.37 (d, *J* = 7.1 Hz, 1H), 7.12 (t, *J* = 6.5 Hz, 1H), 6.77 (s, 1H), 3.78 (dd, *J* = 14.3, 7.1 Hz, 1H), 1.09 (t, *J* = 7.1 Hz, 1H) ppm. ^13^C{^1^H} NMR (101 MHz, CDCl_3_): δ 164.1, 141.2, 131.8, 130.0, 129.8, 128.1, 53.4, 12.8 ppm. HRMS (ESI), *m*/*z*: [M + H]^+^ calcd for C_20_H_22_N_2_O_2_, 322.1681; found 322.1675. FT-IR (KBr) ν = 3085, 2916, 1681, 1614, 1518, 1441, 1259, 1131, 746 cm^–1^.

## Data Availability

The data underlying this study are available in the published article and its Supporting Information

## Supporting Information Available

Reaction optimization details, synthetic procedures, and characterization of new and known compounds (PDF)
