# Supplementary material for: Mild and Functional Group-Tolerant Aerobic N-Dealkylation of Tertiary Amines Promoted by Photoredox Catalysis
Source: J Org Chem. 2023 Jun 9;88(13):8874–81. doi: 10.1021/acs.joc.3c00656 (PMC10336922; doi:10.1021/acs.joc.3c00656)
Supplement: Supplementary file 1 — jo3c00656_si_001.pdf [file jo3c00656_si_001.pdf]

Supporting Information

Mild and Functional Group Tolerant Aerobic *N*-Dealkylation of Tertiary Amines Promoted by Photoredox Catalysis

Ozgur Yilmaz*‡, Marion H. Emmert*§

‡Department of Chemistry, Faculty of Sciences, Mersin University, 33343 Mersin, Turkey.
§Process Research & Development, MRL, Merck & Co., Inc., 126 E Lincoln Ave, Rahway, NJ 07065, USA.

Email: marion.emmert@merck.com
Email: yilmazozgur@mersin.edu.tr

Table of Contents

Optimization of Conditions S4

General Procedure for *N*-dealkylation after optimization (Table S5, Entry 2) S4

NMR and GC spectra for dealkylated products S21

Dibutylamine ($C_8H_{19}N$, 1a) S21

Dietylamine (2a) S22

Dipropylamine (3a) S23

Dipentylamine ($C_{10}H_{23}N$, 4a) S25

Dihexylamine ($C_{12}H_{27}N$, 5a) S26

Dioctylamine ($C_{16}H_{35}N$, 6a) S27

Mopholine (C_4H_9NO, 7a) S28

Piperidine (8a) S29

Dibenzylamine ($C_{14}H_{15}N$, 9a) S30

N-Methylaniline (C_7H_9N, 10a) S31

N-Ethylaniline ($C_8H_{11}N$, 11a) S32

N-Propylaniline ($C_9H_{13}N$, 12a) S33

N,3-Dimethylaniline ($C_8H_{11}N$, 15a) S34

N,4-Dimethylaniline ($C_8H_{11}N$, 16a) S35

1*H*-indole-3-carbaldehyde (C_9H_7NO, 17a) S36

(E)-6,6-dimethyl-*N*-(naphthalen-1-ylmethyl)hept-2-en-4-yn-1-amine ($C_{20}H_{23}N$, 18a) S37

2-(benzhydryloxy)-N-methylethanamine ($C_{16}H_{19}NO$, 19a) S38

10,11-Dihydro-5H-dibenzo[b,f]azepine ($C_{14}H_{13}N$, 20a) S39

3-(2,6-Dimethylphenyl)-1-ethyl-2-methylimidazolidin -4-one ($C_{14}H_{20}NO_2$, 21a) S40

Data of reactions examined by LCAP (liquid chromatography area percent) S41

Reaction with (3S,4R)-methyl 1-benzyl-4-phenylpyrrolidine-3-carboxylate (23) S41

Reaction with (*E*)-ethyl 2-((dimethylamino)methylene)-3-oxobutanoate (24) S43

Reaction with 1-benzyl-4-phenylpiperidine-4-carbonitrile (25) S45

Reaction with 1-(3,5-bis(trifluoromethyl)phenyl)-3-((1R,2R)-2-(dimethylamino)cyclohexyl)thiourea (26) S48

Reaction with 2-(4-chlorophenyl)-1-(4-(2-(diethylamino)ethoxy)phenyl)-1-(p-tolyl)ethanol (27) .. S50

Reaction with (4-methylpiperazin-1-yl)(4-(4,4,5,5-tetramethyl-1,3,2-dioxaborolan-2-yl)phenyl)methanone (28) S53

Reaction with (R)-N-benzyl-1-phenylethanamine (29) S60

Reaction with (R)-2-(benzylamino)-2-phenylethanol (30) S63

Reaction with 8-(4-fluorophenyl)-2-(trifluoromethyl)-5,6,7,8-tetrahydroimidazo[1,2-a]pyrazine (31) S67

Reaction with 1-(thiophen-2-yl)-1,2,3,4-tetrahydropyrrolo[1,2-a]pyrazine (32) S70

Reaction with 6-bromospiro[benzo[e][1,3]oxazine-2,4'-piperidin]-4(3H)-one (33) S75

Reaction with *N,N*-dimethyl-3-((5-(4,4,5,5-tetramethyl-1,3,2-dioxaborolan-2-yl)pyridin-2-yl)oxy)propan-1-amine (34) S80

Reaction with 5-((3,4-dimethoxyphenethyl)(methyl)amino)-2-(3,4-dimethoxyphenyl)-2-isopropylpentanenitrile (35) S82

Reaction with 2-(benzylamino)-4-(4-cyclohexylphenyl)-4-oxobutanoic acid (36) S85

Reaction with 1-benzyl-N-(4-(trifluoromethyl)benzyl)piperidin-4-amine (37) S88

Reaction with *N*-phenylpyrrolidine-2-carboxamide (38) S91

Reaction with 3-bromo-5-(piperidin-2-yl)pyridine (39) S95

Reaction with (S)-2-(diphenyl((trimethylsilyl)oxy)methyl)pyrrolidine (40) S98

Reaction with 2,3,4,9-tetrahydro-1H-pyrido[3,4-b]indole-3-carboxylic acid (41) S100

Reaction with 2-(diethylamino)ethyl 4-amino-3-butoxybenzoate (86) S102

GC-MS and NMR results for mechanistic studies S105

Optimization of Conditions

Initially, the *N*-dealkylation of tributylamine was selected as the model reaction to optimize the reaction conditions. The starting conditions for reaction development were chosen as follows:

NBu_3 (0.27 mmol, 64 µL, 1.0 eq.), 3 mL MeOH, and $Ru(bpy)_3$ (0.0054 mmol, 0.004 g, 0.02 eq.) were weighed into a 4 mL glass vial equipped with a stir bar. The vial was sealed and the reaction mixture was stirred for 24 h at room temperature under an air atmosphere while irradiating with blue LEDs (465nm).

In follow-up experiments, the reaction conditions were changed in order to increase the yield; detailed changes and results are tabulated in table S1.

General Procedure for *N*-dealkylation after optimization (Table S5, Entry 2)

NBu_3 (0.27 mmol, 64 µL, 1.0 eq.), 3 mL MeCN, $Ir[dF(CF_3)ppy]_2(dtbpy))PF_6$[1] (0.0027 mmol, 0.003 g, 0.01 eq.) and CH_3NO_2 (0.27 mmol, 15 µL, 1.0 eq.) were mixed in a 4 mL glass vial equipped with a stir bar. The vial was sealed and the reaction mixture was stirred for 3 h at room temperature while irradiating with blue LEDs.

To determine crude assay yields by GC, decane or dodecane was added to the reaction mixture. The mixture was sampled by diluting an aliquot with MeCN or EtOAc, followed by filtration, and analysis of the filtrate by GC-FID. Yields were determined by use of a previously established calibration curve.

To determine crude assay yields by quantitative ^1H NMR, the reaction mixture was evaporated. Then, $CDCl_3$ and 1,1,2-trichloroethane (3.47 uL, 37.4 umol; 0.14 equiv.) as internal standard were added; the resulting suspension was mixed well, filtered, and analyzed by quantitative ^1H NMR.

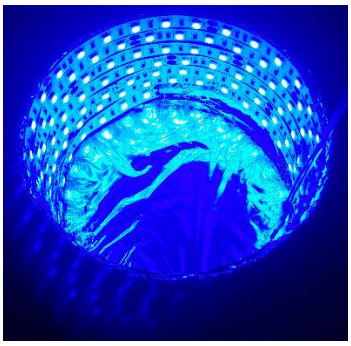
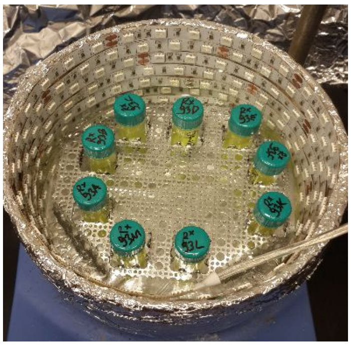
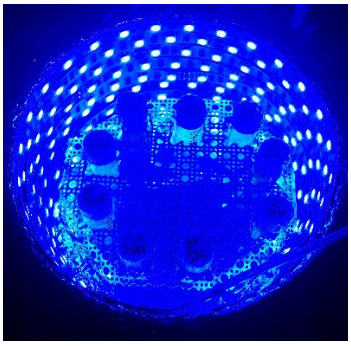

[1] $(Ir[dF(CF_3)ppy]_2(dtbpy))PF_6$ (CAS Number 870987-63-6) used in this study was obtained from Sigma Aldrich (SKU 747793-1G).

Table S1. Optimization of N-dealkylation of NBu_3. Starting conditions for development (entry 1), unless otherwise indicated: NBu_3 (0.27 mmol, 64 µL, 1.0 eq.), 3 mL MeOH, $Ru(bpy)_3$ (0.0054 mmol, 0.004 g, 0.02 eq.), blue leds, r.t., 24 h.

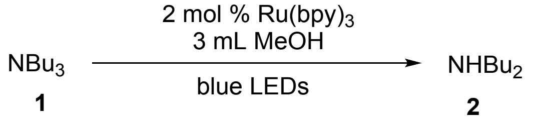

Entry	Changes Compared to Starting Conditions (Entry 1)	Yield (%)[a]
1	--	<5%
2	4 mol% $Ru(bpy)_3$	<5%
3	1 eq. AcOH	<5%
4	1 eq. K_3PO_4	<5%
5	1 eq. K_2CO_3	<5%
6	4mol% $[Ir(dtbbpy)(ppy)_2]PF_6$ instead of $Ru(bpy)_3$	6%
7	4mol% $(Ir[dF(CF_3)ppy]_2(dtbpy))PF_6$ instead of $Ru(bpy)_3$	11%
**8**	**4mol% $(Ir[dF(CF_3)ppy]_2(dtbpy))PF_6$ instead of $Ru(bpy)_3$ and 1 eq. CH_3NO_2**	**88%**
[a]Yields were determined by GC. [b]N.R.= No Reaction		

Table S2. Solvent optimization of N-dealkylation of NBu_3. Starting conditions for development (Table1/entry 8), unless otherwise indicated: NBu_3 (0.27 mmol, 64 µL, 1.0 eq.), 3 mL MeOH, $(Ir[dF(CF_3)ppy]_2(dtbpy))PF_6$ (0.0108 mmol, 0.012 g, 0.04 eq.), CH_3NO_2 (0.27 mmol, 15 µL , 1.0 eq), blue leds, r.t., 24 h.

	Changes Compared to Conditions	Yield (%)[a]
**1**	**with MeCN as solvent**	**94%**
2	with DMSO as solvent	85%
3	with DMF as solvent	82%
4	with IPA as solvent	84%
[a]Yields were determined by GC. [b]N.R.= No Reaction		

Table S3. Amount of catalyst optimization of N-dealkylation of NBu_3. Starting conditions for development (Table2/entry 1), unless otherwise indicated: NBu_3 (0.27 mmol, 64 µL, 1.0 eq.), 3 mL MeCN, $(Ir[dF(CF_3)ppy]_2(dtbpy))PF_6$ (0.0108 mmol, 0.012 g, 0.04 eq.), CH_3NO_2 (0.27 mmol, 15 µL , 1.0 eq), blue leds, r.t., 24 h.

	Changes Compared to Conditions	Yield (%)[a]
1	2mol% $(Ir[dF(CF_3)ppy]_2(dtbpy))PF_6$	94%
**2**	**1mol% $(Ir[dF(CF_3)ppy]_2(dtbpy))PF_6$**	**93%**
3	0.5mol% $(Ir[dF(CF_3)ppy]_2(dtbpy))PF_6$	77%
[a]Yields were determined by GC. [b]N.R.= No Reaction		

Table S4. Amount of CH_3NO_2 optimization of N-dealkylation of NBu_3. Starting conditions for development (Table3/entry 2), unless otherwise indicated: NBu_3 (0.27 mmol, 64 µL, 1.0 eq.), 3 mL MeCN, $(Ir[dF(CF_3)ppy]_2(dtbpy))PF_6$ (0.0027 mmol, 0.003 g, 0.01 eq.), CH_3NO_2 (0.27 mmol, 15 µL , 1.0 eq), blue leds, r.t., 24 h.

	Changes Compared to Conditions	Yield (%)[a]
1	2 eq. CH_3NO_2	88%
2	0.5 eq. CH_3NO_2	49%
3	0.25 eq. CH_3NO_2	17%
[a]Yields were determined by GC. [b]N.R.= No Reaction		

Table S5. Reaction time optimization of N-dealkylation of NBu_3. Starting conditions for development (Table3/entry 2), unless otherwise indicated: NBu_3 (0.27 mmol, 64 µL, 1.0 eq.), 3 mL MeCN, (Ir[dF(CF_3)ppy]$_2$(dtbpy))PF_6 (0.0027 mmol, 0.003 g, 0.01 eq.), CH_3NO_2 (0.27 mmol, 15 µL , 1.0 eq), blue leds, r.t., 24 h.

	Changes Compared to Conditions	Yield (%)[a]
1	12h	93%
2	6h	93%
**3**	**3h**	**93%**
4	1h	79%
[a]Yields were determined by GC. [b]N.R.= No Reaction		

Table S6. Control reactions for mechanistic studies of N-dealkylation of NBu_3. For the best conditions, unless otherwise indicated: NBu_3 (0.27 mmol, 64 µL, 1.0 eq.), 3 mL MeCN, (Ir[dF(CF_3)ppy]$_2$(dtbpy))PF_6 (0.0027 mmol, 0.003 g, 0.01 eq.), CH_3NO_2 (0.27 mmol, 15 µL , 1.0 eq), blue leds, r.t., 3 h.

	Changes Compared to Conditions	Yield (%)[a]
1	Without O_2, with N_2 atmosphere	14%
2	Without Blue Leds	N.R.
3	Without (Ir[dF(CF_3)ppy]$_2$(dtbpy))PF_6	N.R.
4	Without CH_3NO_2	3%
5	With 50mol% BHT	37%
[a]Yields were determined by GC. [b]N.R.= No Reaction		

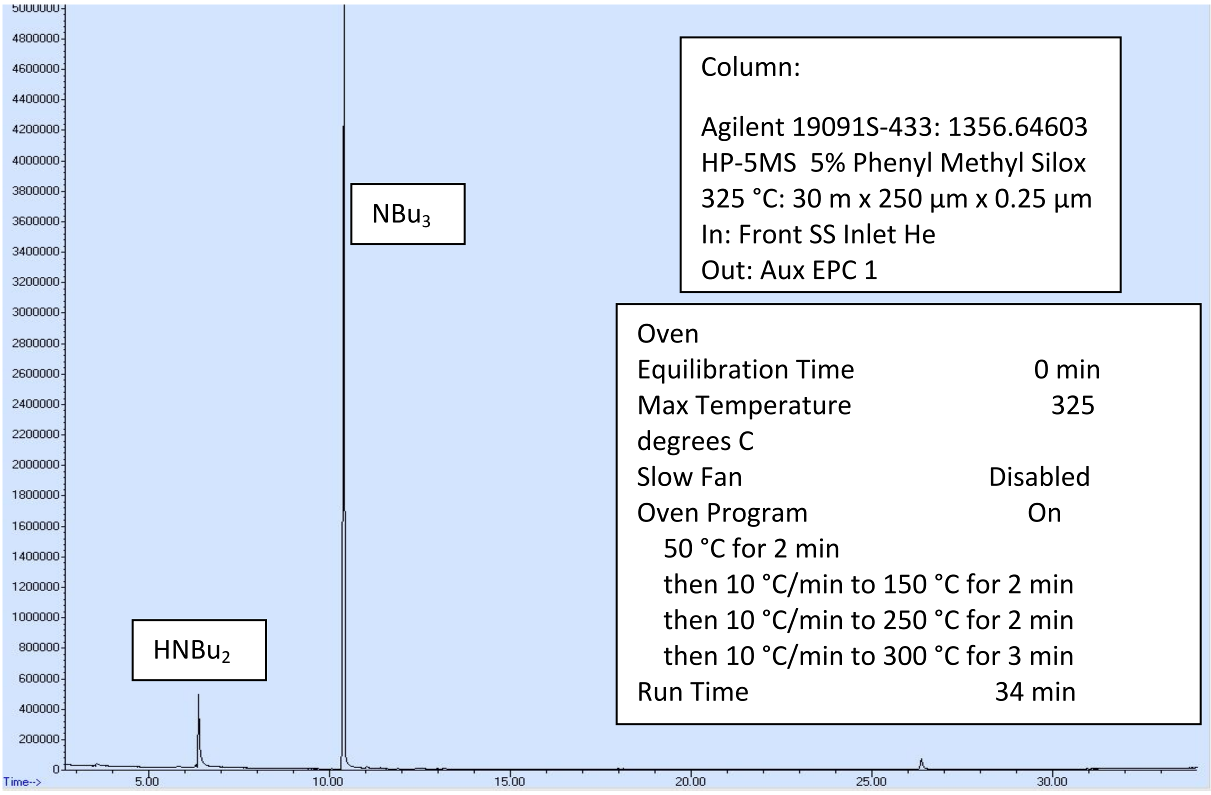

Figure S1. GC-MS result for reaction in TableS1/Entry7 (MS trace).

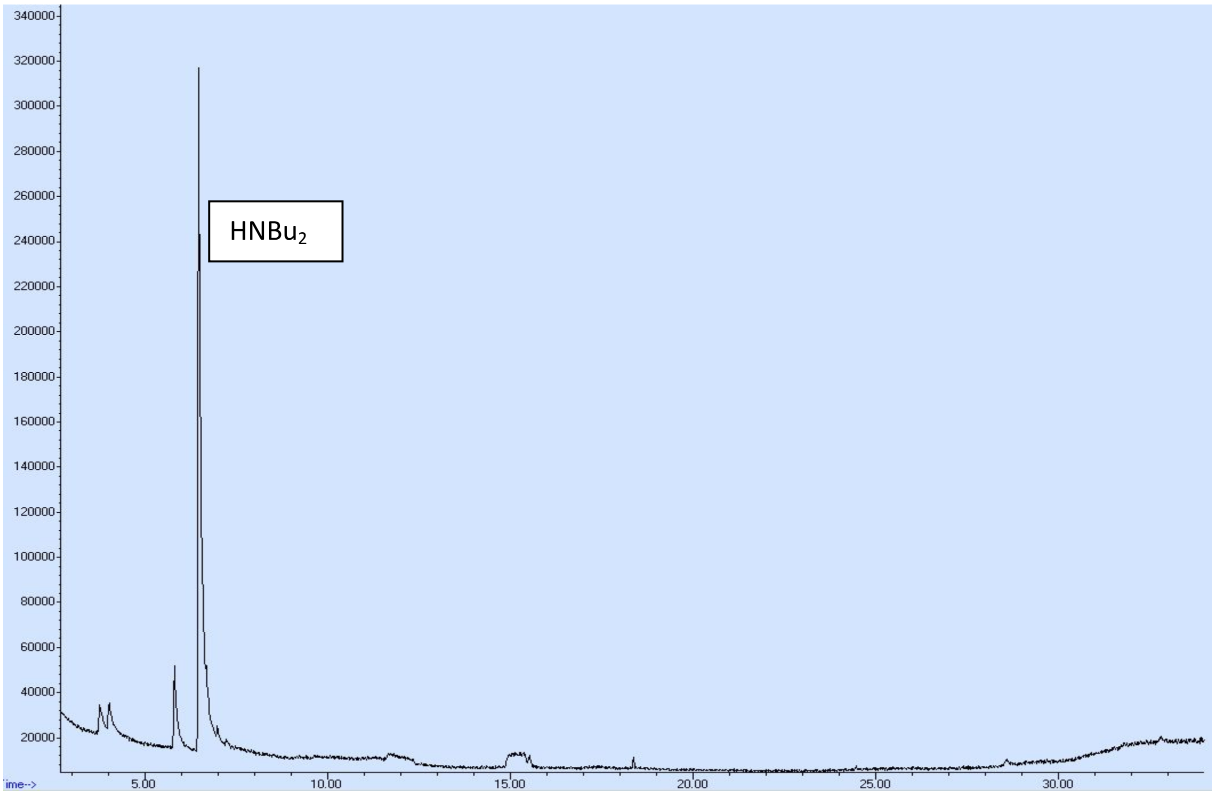

Figure S2. GC-MS result for reaction in TableS1/Entry8 (MS trace).

Table S7. Substrate scope for tertiary amines (simple, benzilic and complex were given respectively). General Conditions: Amine (0.27 mmol), MeCN (3 mL), Ir[dF(CF_3)ppy]$_2$(dtbpy))PF_6 (0.01 eq.) CH_3NO_2 (1.0 eq.), 3h, r.t., blue leds.

NR_3 —[%1mol (Ir[dF(CF_3)ppy]$_2$(dtbpy))PF_6; 1 eq. CH_3NO_2, 3 mL MeCN, blue Leds, 3 h]→ NHR_2 yield (isolated)

Substrate (Simple Tertiary Amines)	Product(s)	Yield[a] (Isolated Yield)
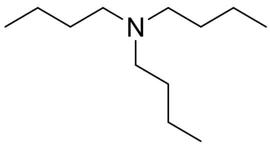 **1**	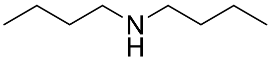 **1a**	93% (90%)
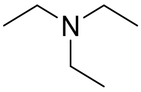 **2**	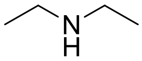 **2a**	96%
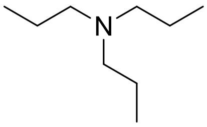 **3**	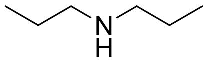 **3a**	94%
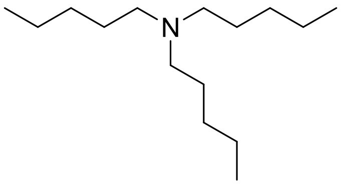 **4**	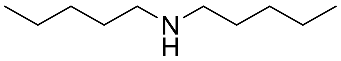 **4a**	87% (84%)
$NHex_3$ **5**	$HNHex_2$ **5a**	84% (76%)
N(n-octyl)$_3$ **6**	HN(n-octyl)$_2$ **6a**	83% (78%)

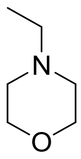 **7**	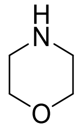 **7a**	61%
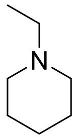 **8**	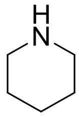 **8a**	35%<br><br>mainly remaining starting material
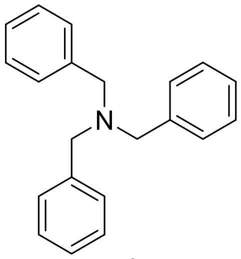 **9**	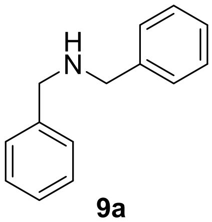 **9a** 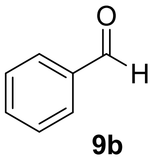 **9b**	**9a**<br>78% (76%)<br><br>**9b**<br>57%
**Substrate (Benzylic Tertiary Amines)**	**Product(s)**	**Yield[a] (Isolated Yield)**
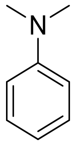 **10**	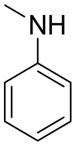 **10a**	48% (45%)<br><br>51% remaining starting material
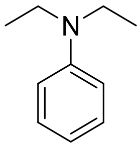 **11**	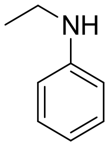 **11a**	42% (35%)<br><br>57% remaining starting material<br><br>49%[b], 42%[c], 50%[d], 19%[e]
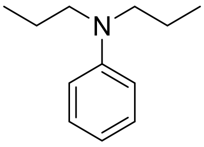 **12**	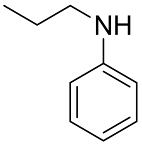 **12a**	36% (33%)<br><br>62% remaining starting material

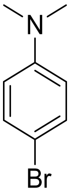<br>**13**	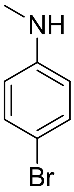<br>**13a**	N.R.<br><br>98% remaining starting material
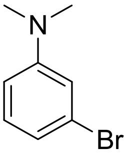<br>**14**	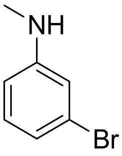<br>**14a**	N.R.<br><br>97% remaining starting
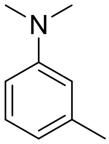<br>**15**	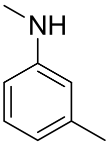<br>**15a**	63% (60%)<br><br>35% remaining starting material
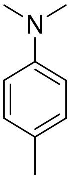<br>**16**	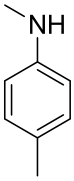<br>**16a**	55% (53%)<br><br>44% remaining starting material
**Substrate (Complex Tertiary Amines)**	**Product(s)**	**Yield[a] (Isolated Yield)**
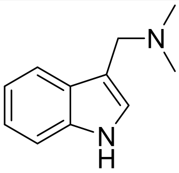<br>**17**	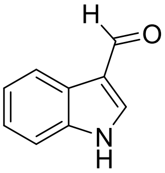<br>**17a**	67% (58%)
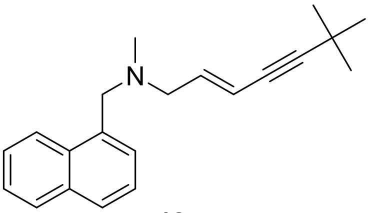<br>**18**	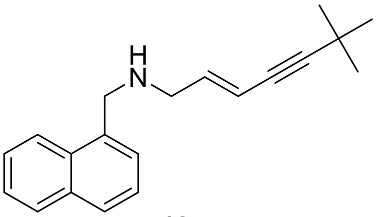<br>**18a**	44% (40%)

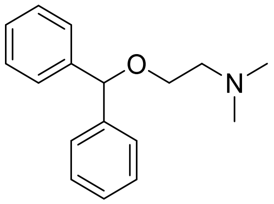<br>**19**	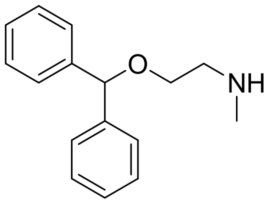<br>**19a**	74%
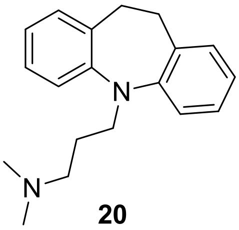<br>**20**	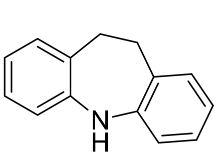<br>**20a**	59% (51%)
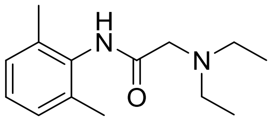<br>**21**	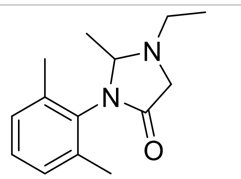<br>**21a**	62% (55%)
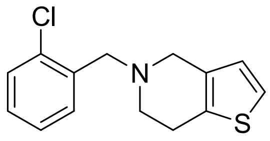<br>**22**		N.R.

[a]Yields were determined by quantitative, crude ^{1}H NMR using 1,1,2-trichloroethane as internal standard or by GC. [b]48 h. [c]2 mol% (Ir[dF(CF3)ppy]2(dtbpy))PF6, [d]2eq. CH_3NO_2, [e]MeOH as solvent.

Table S8. Substrate scope for tertiary and secondary amines for which dealkylation product can be obtained investigated via UPLCMS analysis. Results are documented in liquid chromatography area percent (LCAP), using the extracted UV trace at 210 nm. General Conditions: Amine (0.27 mmol), MeCN (3 mL), Ir[dF(CF_3)ppy]$_2$(dtbpy))PF_6 (0.01 eq.) CH_3NO_2 (1.0 eq.), 3h, r.t., blue leds.

NR_3 → NHR_2 (%1mol (Ir[dF(CF_3)ppy]$_2$(dtbpy))PF_6; 1 eq. CH_3NO_2, 3 mL MeCN; blue Leds, 3 h)				
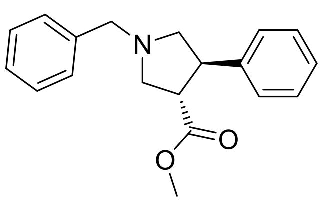<br>**23**<br>**2 h: 22 LCAP -Bn**<br>**18 h: 13 LCAP -Bn**	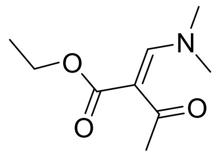<br>**24**<br>**2 h: 5 LCAP -Me**<br>**18 h: 20 LCAP -Me, no rsm**	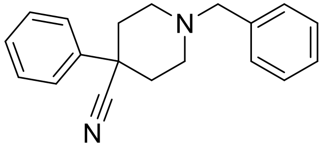<br>**25**<br>**2 h: <5 LCAP -Bn**<br>**18 h: 13 LCAP -Bn**	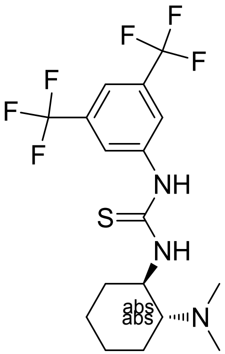<br>**26**<br>**2 h : 80 LCAP -Me**<br>**18 h: 23 LCAP -Me**	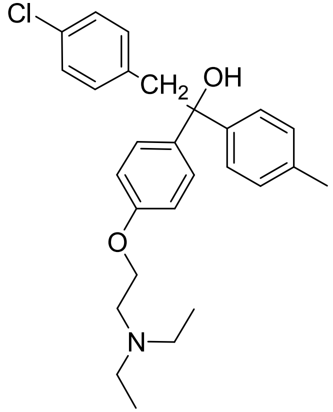<br>**27**<br>**2 h: 58 LCAP -Et**<br>**18 h: 63 LCAP -Et**
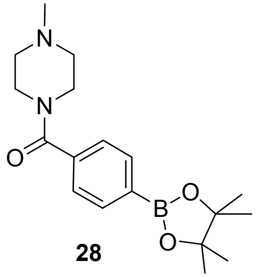<br>**28**<br>**2 h: ~10 LCAP -Me**<br>**18 h: 15 LCAP -Me**<br>**no rsm**	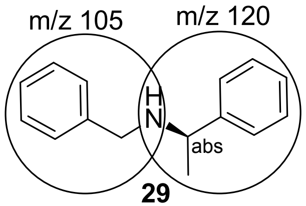<br><br>**29**<br>**2 h: 13 LCAP m/z 120; 7 LCAP m/z 105**<br>**18 h: 25 LCAP m/z 120; 15 LCAP m/z 105**	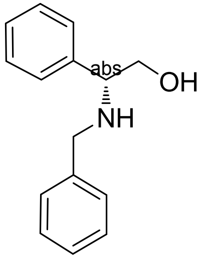<br>**30**<br>**2 h: 3 LCAP m/z 138 (-Bn)**<br>**18 h: 8 LCAP m/z 138 (-Bn)**	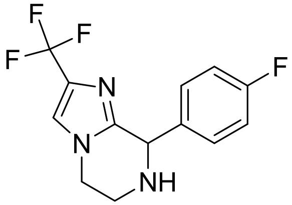<br>**31**<br>**2 h: m/z 183, 17 LCAP**<br>**18 h: m/z 183, 26 LCAP**<br>**183= enamine with *N*-deprotonation**	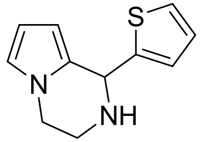<br>**32**<br>**2 h: m/z 202, 18 LCAP**<br>**18 h: m/z 202, 44 LCAP**<br>**202= enamine with *N*-deprotonation**

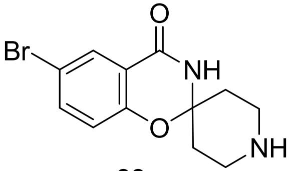 **33** 2 h: m/z 294, no LCAP<br>18 h: m/z 294, 16 LCAP<br>294= enamine with *N*-deprotonation	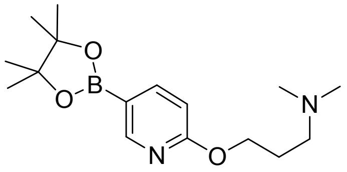 **34** 2 h: 20 LCAP -Me<br>18 h: no rsm, no pdt	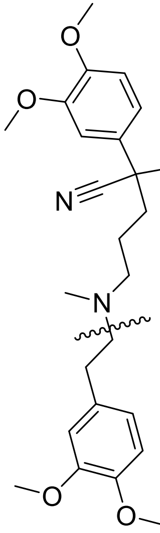 **35** dealkylation (as indicated)<br>2 h: 15 LCAP<br>18 h: 27 LCAP	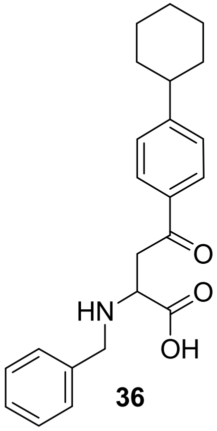 **36** 2 h: 1 LCAP -Bn<br>18 h: 5 LCAP $BnNH_2$<br>mostly rsm	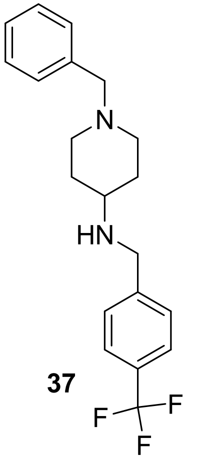 **37** 2 h: 8 LCAP -CF_3Bn<br>18 h: 30 LCAP -CF_3Bn
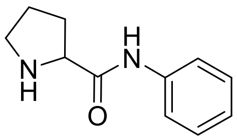 **38** 2 h: m/z 189, 4 LCAP<br>18 h: m/z 189, 12 LCAP<br>189= enamine with *N*-deprotonation	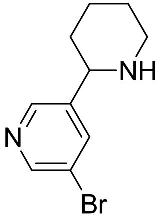 **39** 2 h: m/z 239, 9 LCAP<br>18 h: m/z 239, 20 LCAP<br>239= enamine with *N*-deprotonation	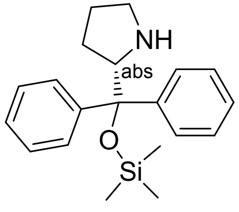 **40** rsm: 23 LCAP -TBS<br>2 h: 44 LCAP m/z 183<br>18 h: 51 LCAP m/z 183<br>183 = benzophenone	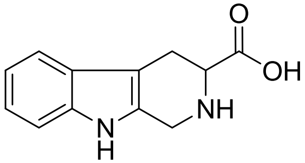 **41** 2 h: m/z 168, 24 LCAP<br>18 h: m/z 168, 35 LCAP	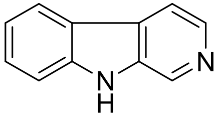 m/z: 168.07 (100.0%), 169.07 (12.6%)

Table S9. Substrate scope for tertiary and secondary amines for which dealkylation product can be obtained investigated via UPLCMS analysis. Results are documented in liquid chromatography area percent (LCAP), using the extracted UV trace at 210 nm. General Conditions: Amine (0.27 mmol), MeCN (3 mL), Ir[dF(CF$_3$)ppy]$_2$(dtbpy))PF$_6$ (0.01 eq.) CH_3NO_2 (1.0 eq.), 3h, r.t., blue leds. Some reactions were repeated using MeOH instead of MeCN or without CH_3NO_2 to compare results. Obtained results are shown in blue font.

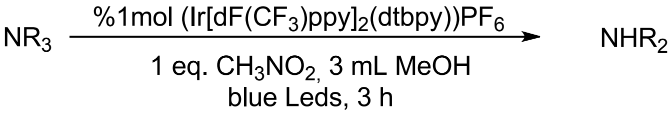

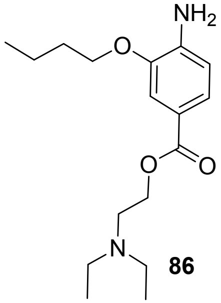 **86**	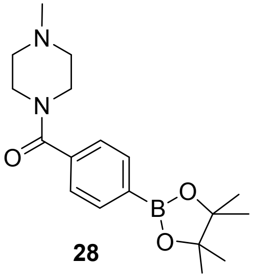 **28**	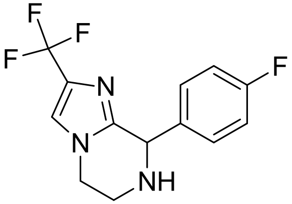 **31**	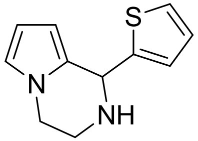 **32**	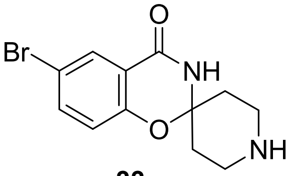 **33**
**18 h: 13 LCAP -Et With MeOH as solvent instead of MeCN and 2 eq. CH_3NO_2**	**18 h: 56 LCAP -Me With MeOH as solvent instead of MeCN and 2 eq. CH_3NO_2**	**2 h: m/z 183, 11 LCAP without CH_3NO_2**	**2 h: m/z 202, 14 LCAP without CH_3NO_2**	**18 h: m/z 294, no LCAP without CH_3NO_2**

Table S10. Substrate scope for tertiary and secondary amines for which no dealkylation product can be obtained investigated via UPLCMS analysis. Results are documented in liquid chromatography area percent (LCAP), using the extracted UV trace at 210 nm. General Conditions: Amine (0.27 mmol), MeCN (3 mL), Ir[dF(CF$_3$)ppy]$_2$(dtbpy))PF$_6$ (0.01 eq.) CH_3NO_2 (1.0 eq.), 3h, r.t., blue leds. Undefined side reactions (e.g., decomposition) are noted as "other side reaction" in the table. Rsm = remaining starting material.

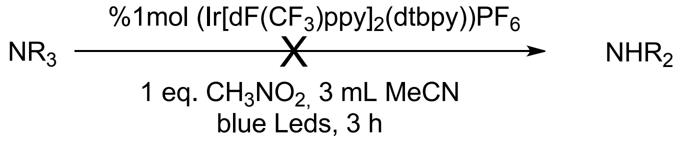

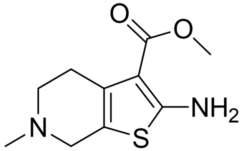<br>**42**<br>No dealkylation; other side reaction and rsm	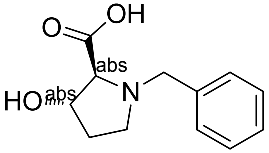<br>**43**<br>No dealkylation; other side reaction and rsm	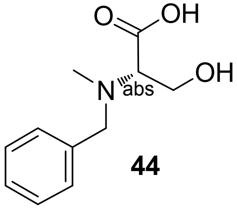<br>**44**<br>No dealkylation; other side reaction and rsm	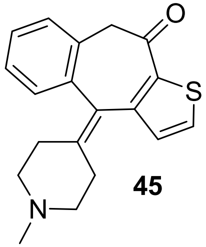<br>**45**<br>No dealkylation; other side reaction and rsm	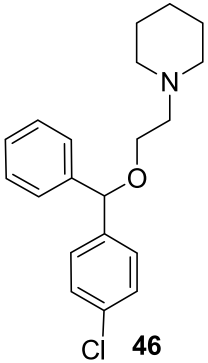<br>**46**<br>No dealkylation; other side reaction and rsm
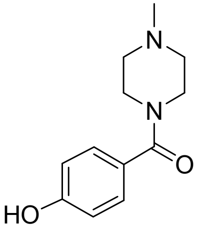<br>**47**<br>No dealkylation; mostly rsm	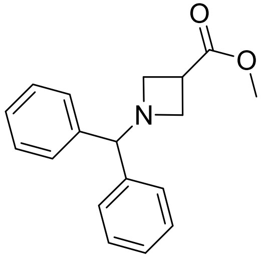<br>**48**<br>No dealkylation; other side reaction and rsm	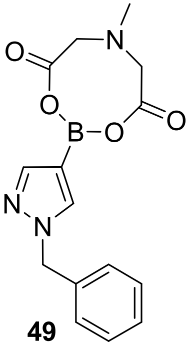<br>**49**<br>No dealkylation; mostly rsm	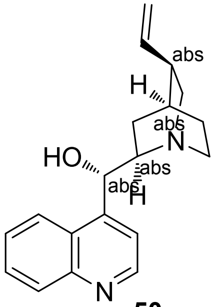<br>**50**<br>No dealkylation; mostly rsm	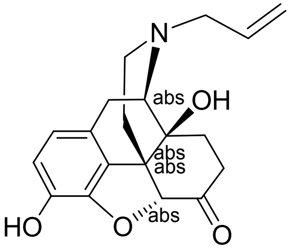<br>**51**<br>No dealkylation; other side reaction and rsm

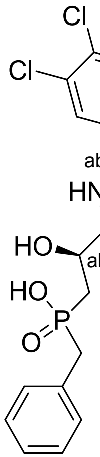 **52** No dealkylation; mostly rsm	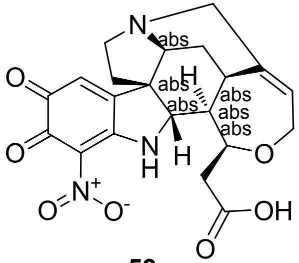 **53** No dealkylation; mostly rsm	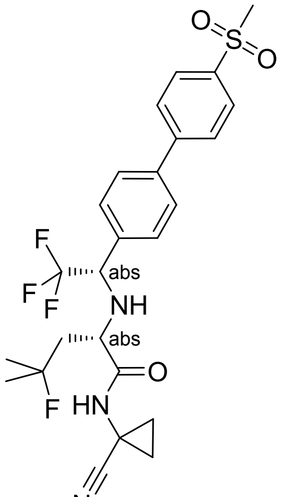 **54** No dealkylation; mostly rsm	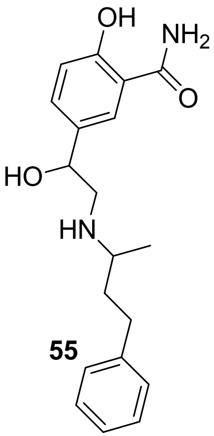 **55** No dealkylation; mostly rsm	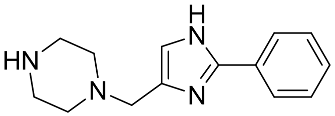 **56** No clear product, mostly rsm
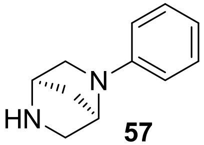 **57** No dealkylation, no rsm after 2 h	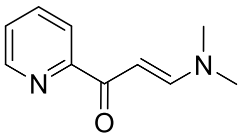 **58** No dealkylation; mostly rsm	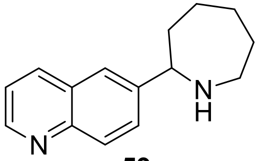 **59** No clear product, mostly rsm	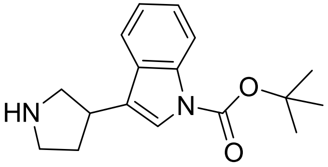 **60** No clear product, mostly rsm	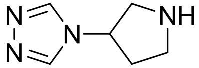 **61** No clear product, mostly rsm

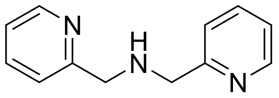<br>**62**<br>2/18 h: clear product, overlaps with solvent peak & not quantifyable<br>18 h: 22 LCAP rsm	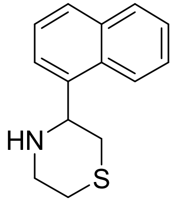<br>**63**<br>18 h: 2 new products m/z 245 (+ O)<br>6 LCAP ^ 14 LCAP	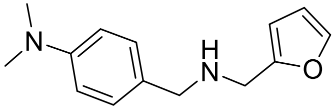<br>**64**<br>46% rsm and products mixture	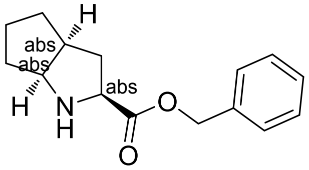<br>**65**<br>No clear product, mostly rsm	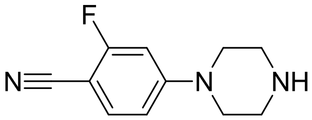<br>**66**<br>No clear product, 58 LCAP rsm
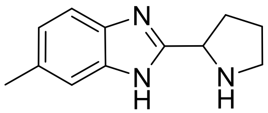<br>**67**<br>No clear product, mostly rsm	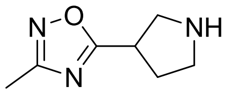<br>**68**<br>No clear product, mostly rsm	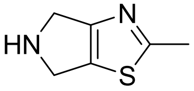<br>**69**<br>No clear product, mostly rsm	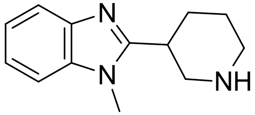<br>**70**<br>No clear product, mostly rsm	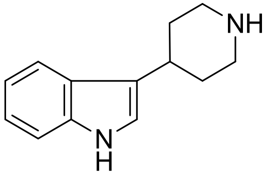<br>**71**<br>No clear product, mostly rsm
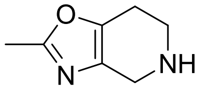<br>**72**<br>No clear product, mostly rsm	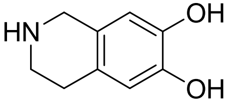<br>**73**<br>No clear product, mostly rsm	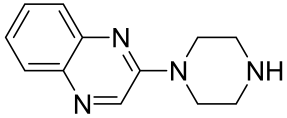<br>**74**<br>No clear product, mostly rsm	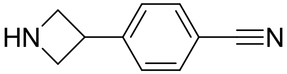<br>**75**<br>No clear product, mostly rsm	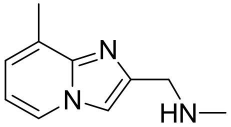<br>**76**<br>No clear product, mostly rsm

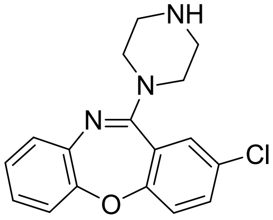<br>**77**<br>No clear product, mostly rsm	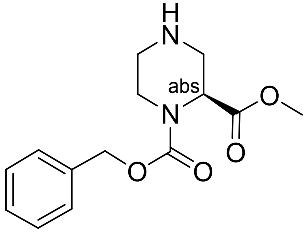<br>**78**<br>No clear product, mostly rsm	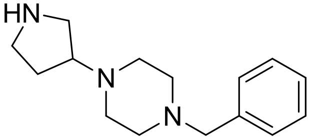<br>**79**<br>No clear product, mostly rsm	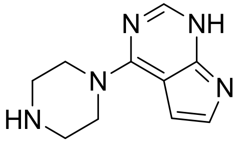<br>**80**<br>No clear product, mostly rsm	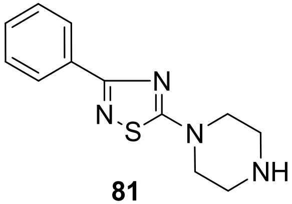<br>**81**<br>No clear product, mostly rsm
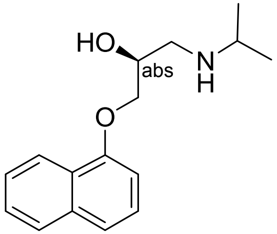<br>**82**<br>No clear product, mostly rsm	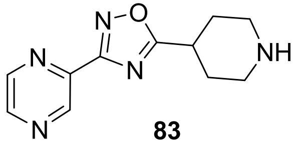<br>**83**<br>No clear product, mostly rsm	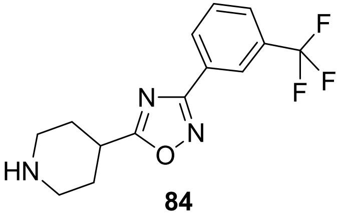<br>**84**<br>No clear product, mostly rsm	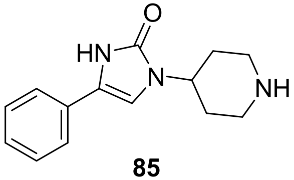<br>**85**<br>No clear product, mostly rsm	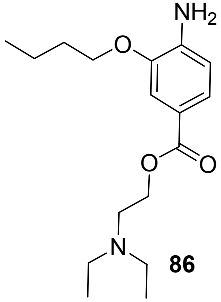<br>**86**<br>No dealkylation; other side reaction and rsm

Synthesis of non-symmetrically substituted amides by employing the dealkylated amine product

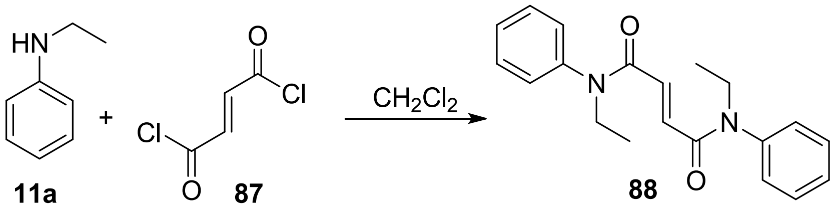

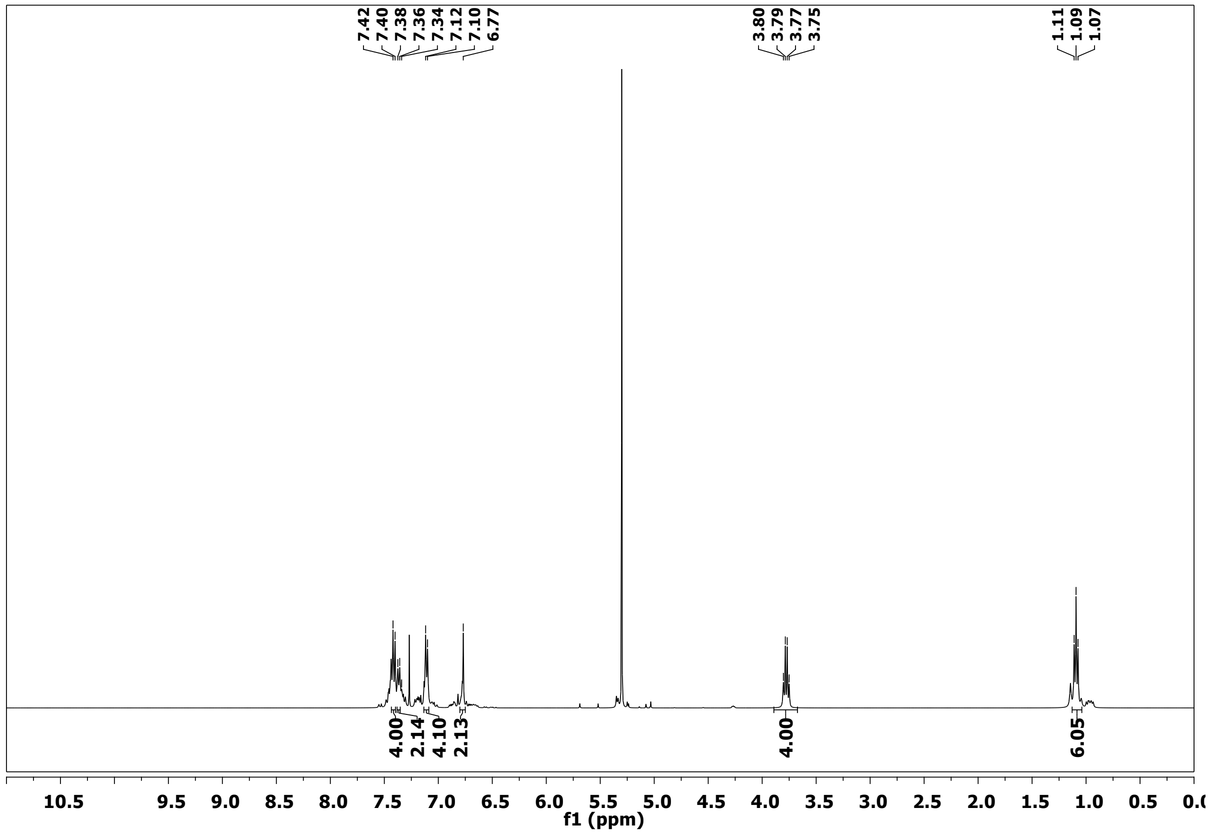

Figure S3. ^{1}H NMR of compound ***(88)*** *($CDCl_3$), 400 MHz.*

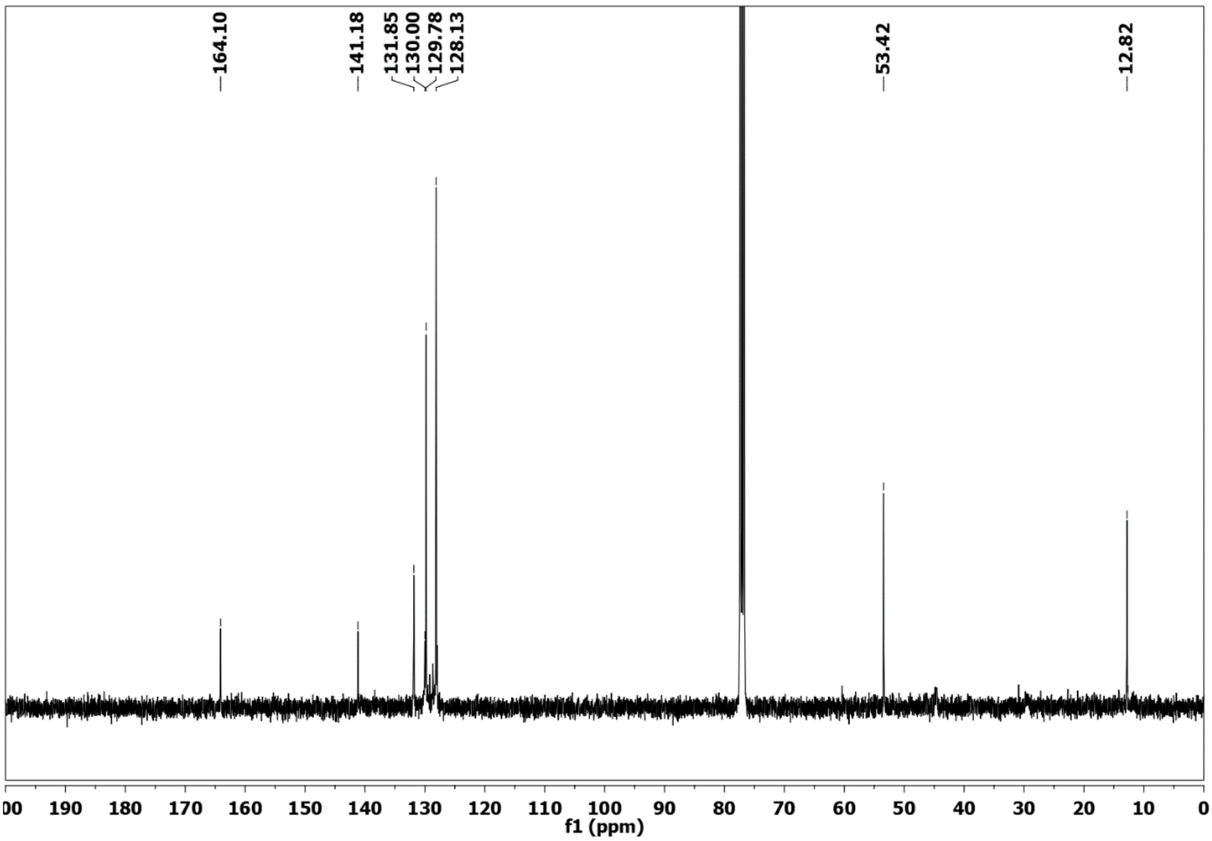

*Figure S4. ^{13}C NMR of compound **(37)** ($CDCl_3$), 101 MHz.*

NMR and GC spectra for dealkylated products

Dibutylamine ($C_8H_{19}N$, 1a)

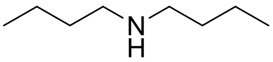

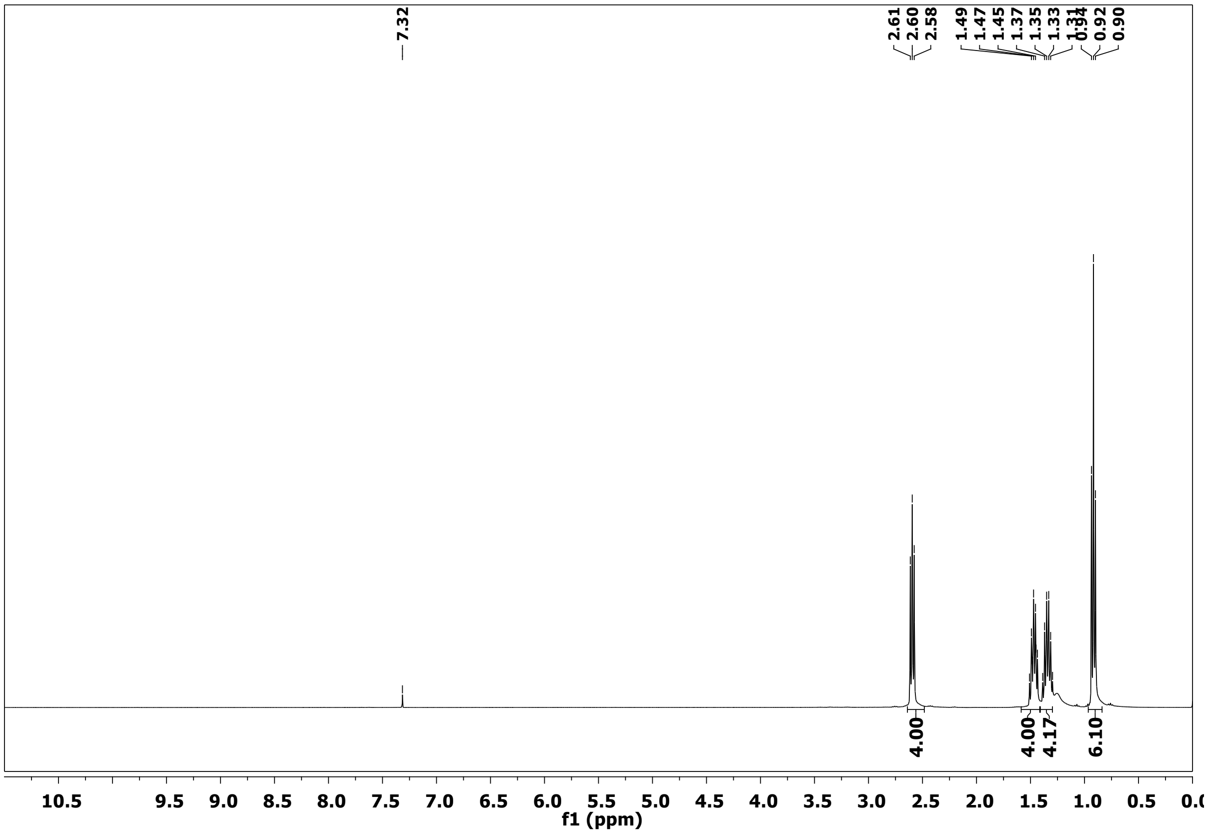

*Figure S5. ^{1}H NMR of Dibutylamine **(1a)** ($CDCl_3$), 400 MHz.*

Dietylamine (2a)

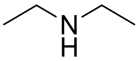

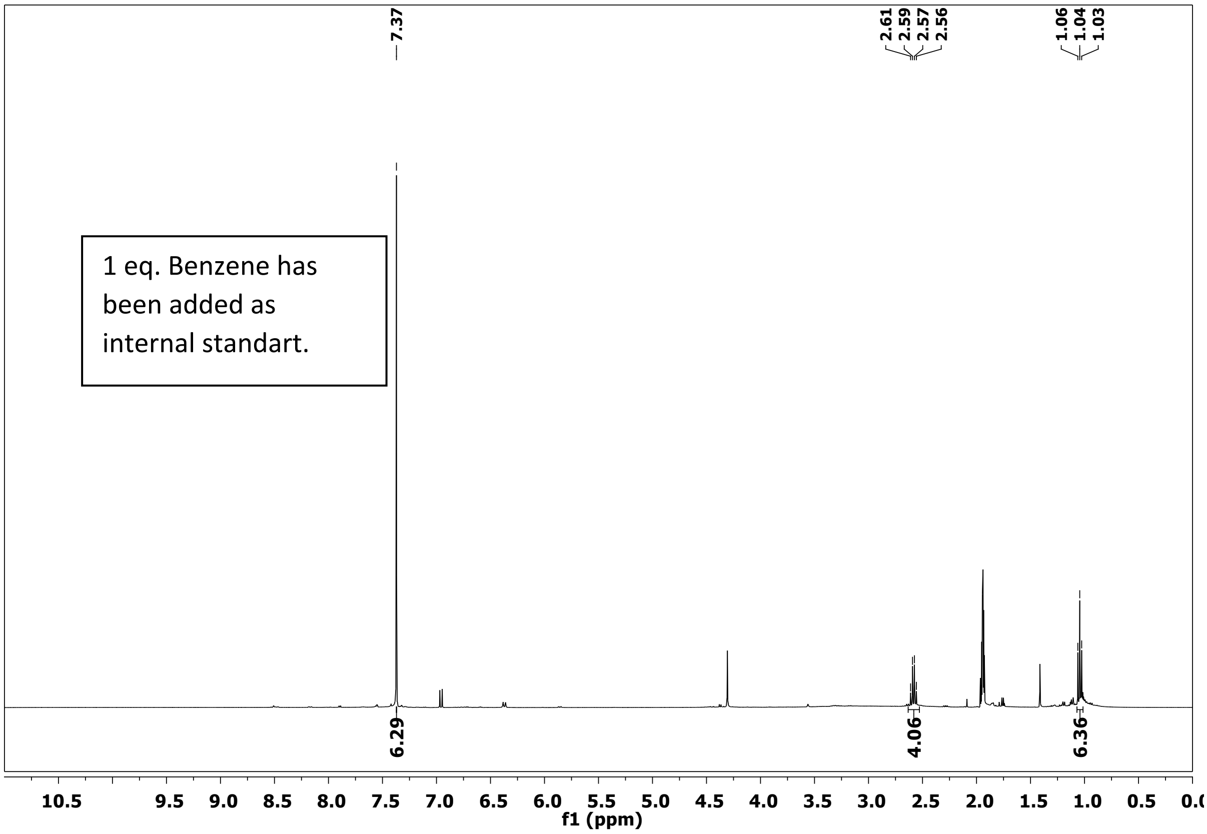

Figure S6. [1]*H-NMR spectrum of crude mixture after N-dealkylation reaction of triethylamine. The signal of the internal standard (benzene) can be seen at 7.37 ppm (CD_3CN). The reaciton set up in CD_3CN and the NMR spectrum measured directly after the reactian time end, 400 MHz.*

Dipropylamine (3a)

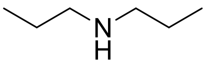

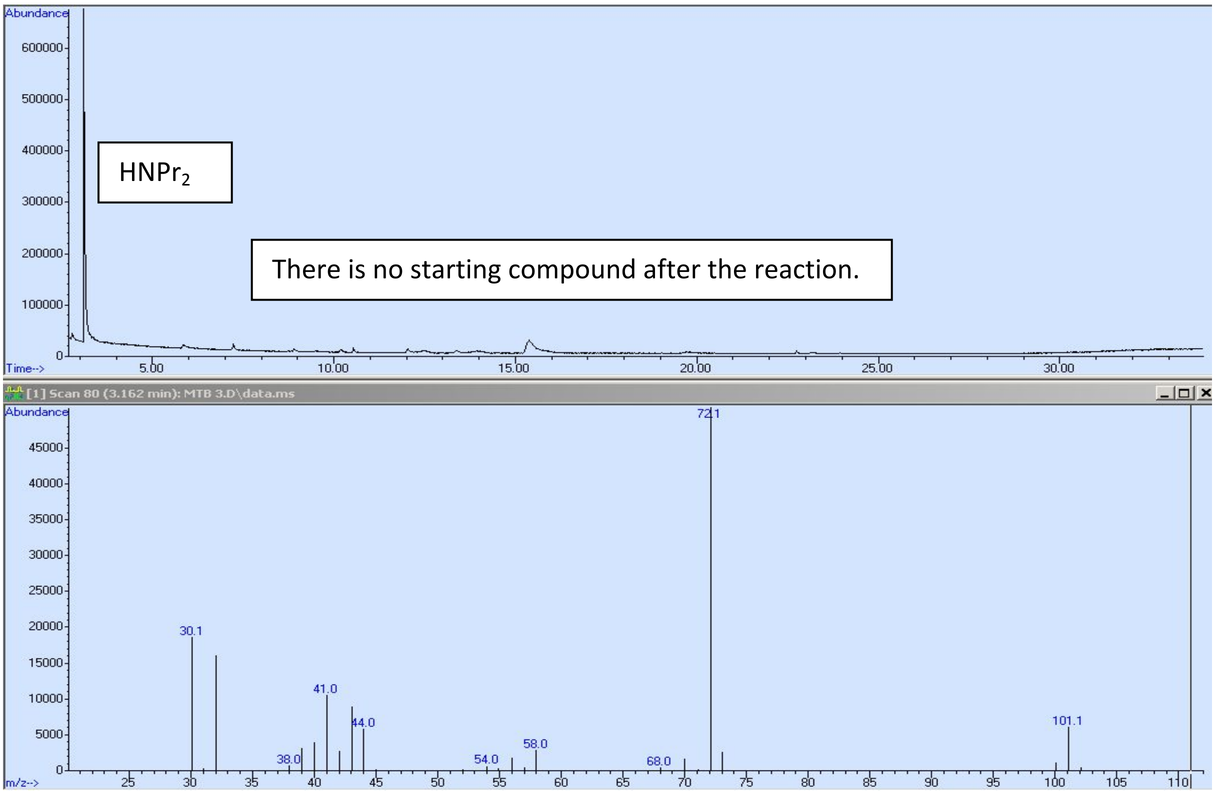

*Figure S7. GC-MS result for reaction with NPr$_3$ (**3**).*

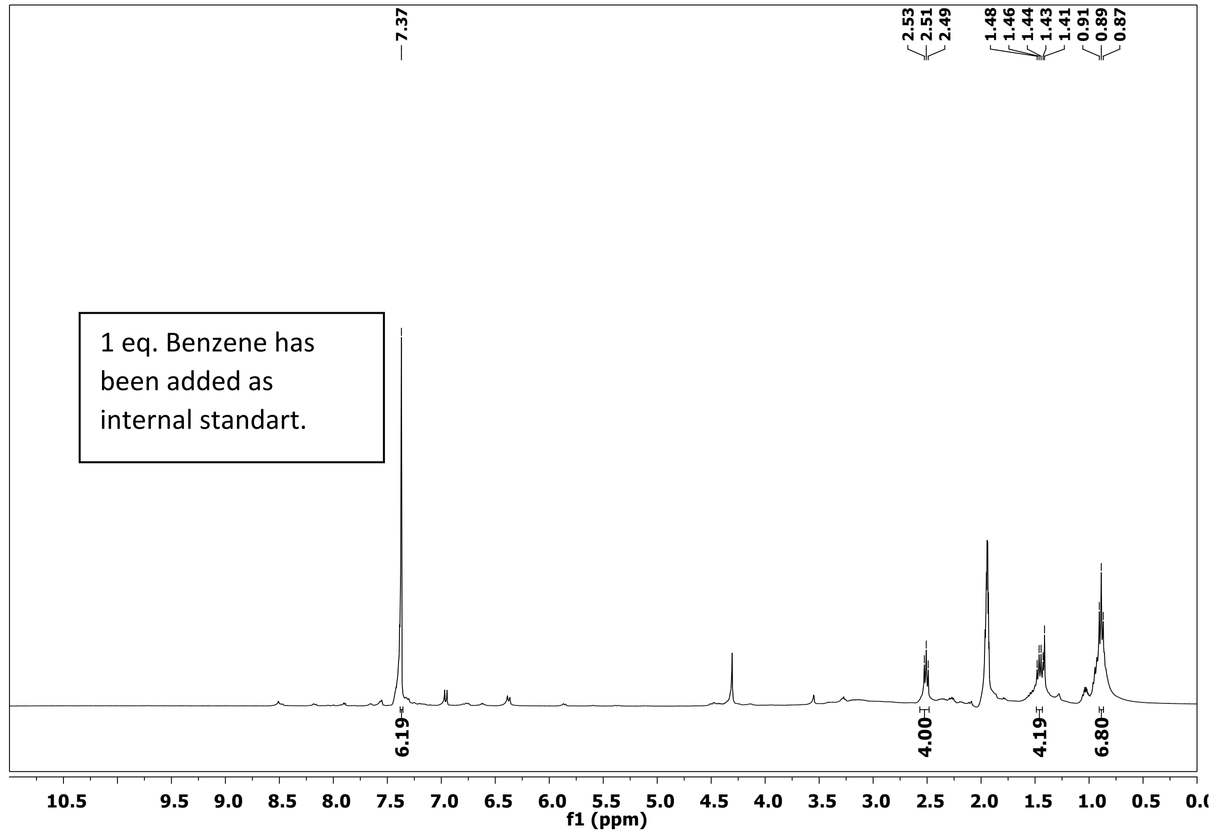

Figure S8. ¹H-NMR spectrum of crude mixture after N-dealkylation reaction of tripropylamine. The signal of the internal standard (benzene) can be seen at 7.37 ppm (CD_3CN). The reaciton set up in CD_3CN and the NMR spectrum measured directly after the reactian time end, 400 MHz.

Dipentylamine ($C_{10}H_{23}N$, 4a)

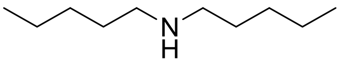

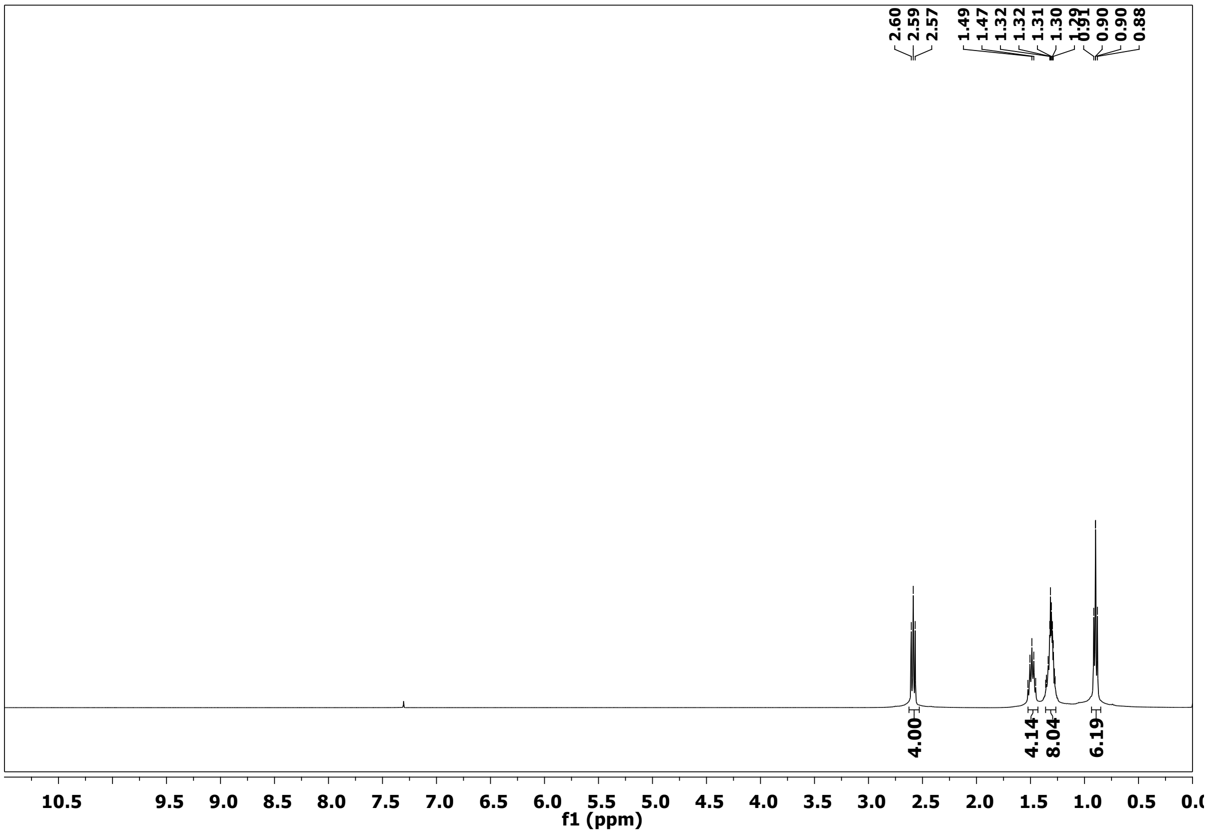

Figure S9. ^{1}H NMR of Dipentylamine ***(4a)*** *($CDCl_3$), 400 MHz.*

Dihexylamine ($C_{12}H_{27}N$, 5a)

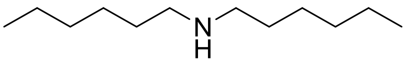

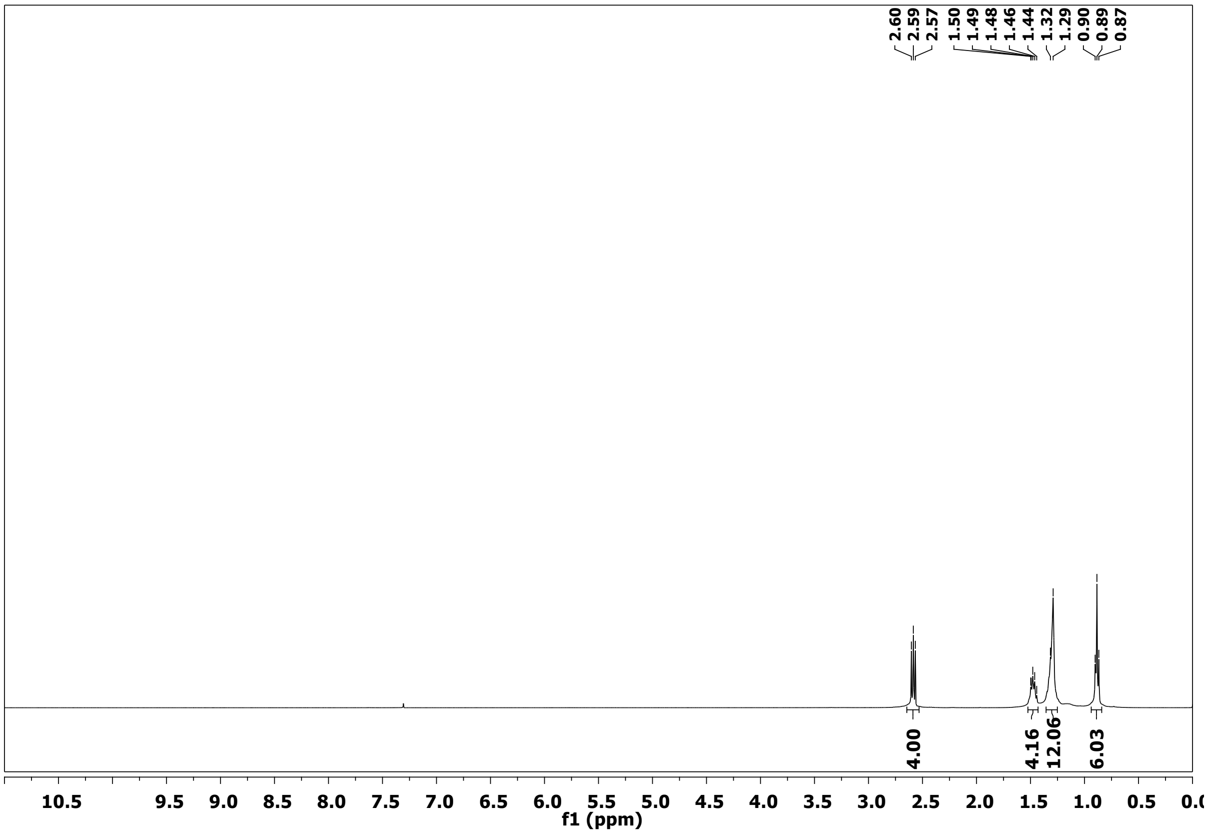

*Figure S10. ^{1}H NMR of Dihexylamine **(5a)** ($CDCl_3$), 400 MHz.*

Dioctylamine ($C_{16}H_{35}N$, 6a)

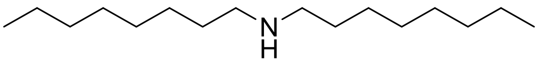

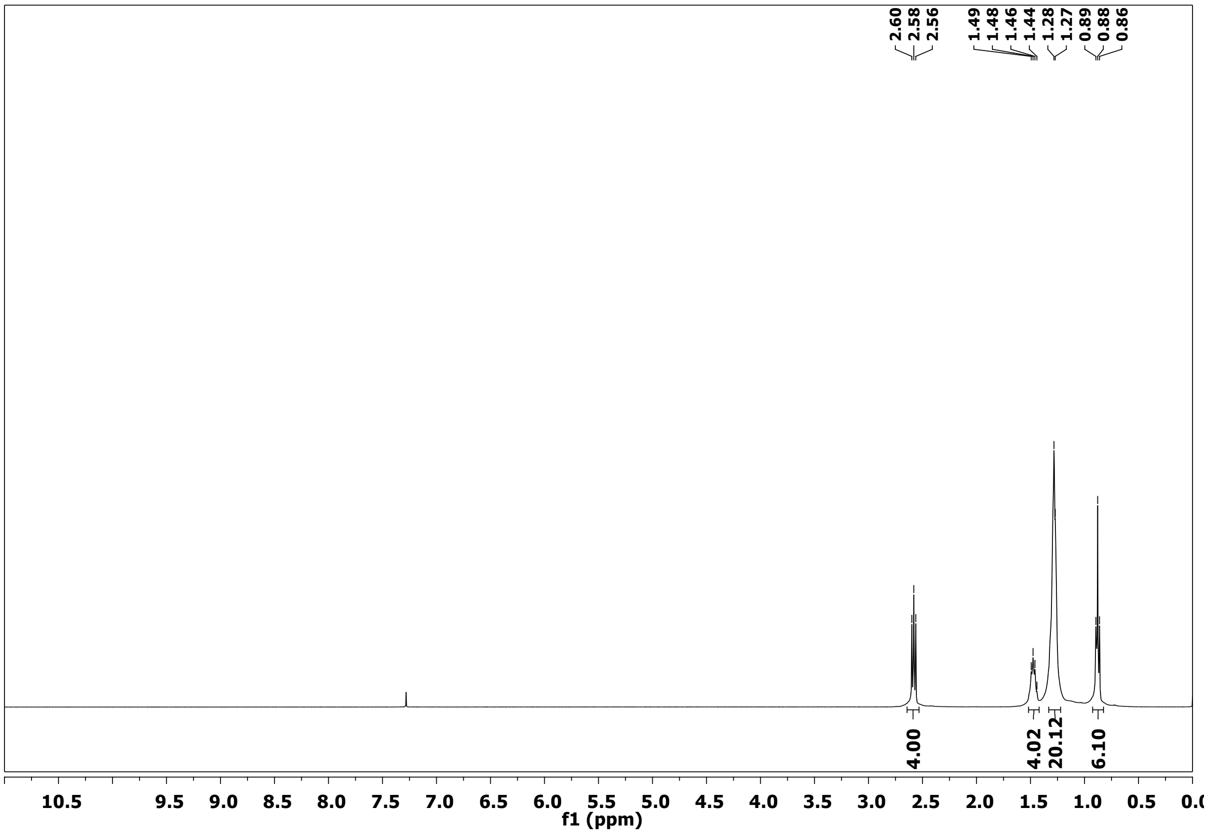

*Figure S11. ^{1}H NMR of Dioctylamine **(6a)** ($CDCl_3$), 400 MHz.*

Mopholine (C_4H_9NO, 7a)

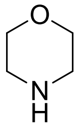

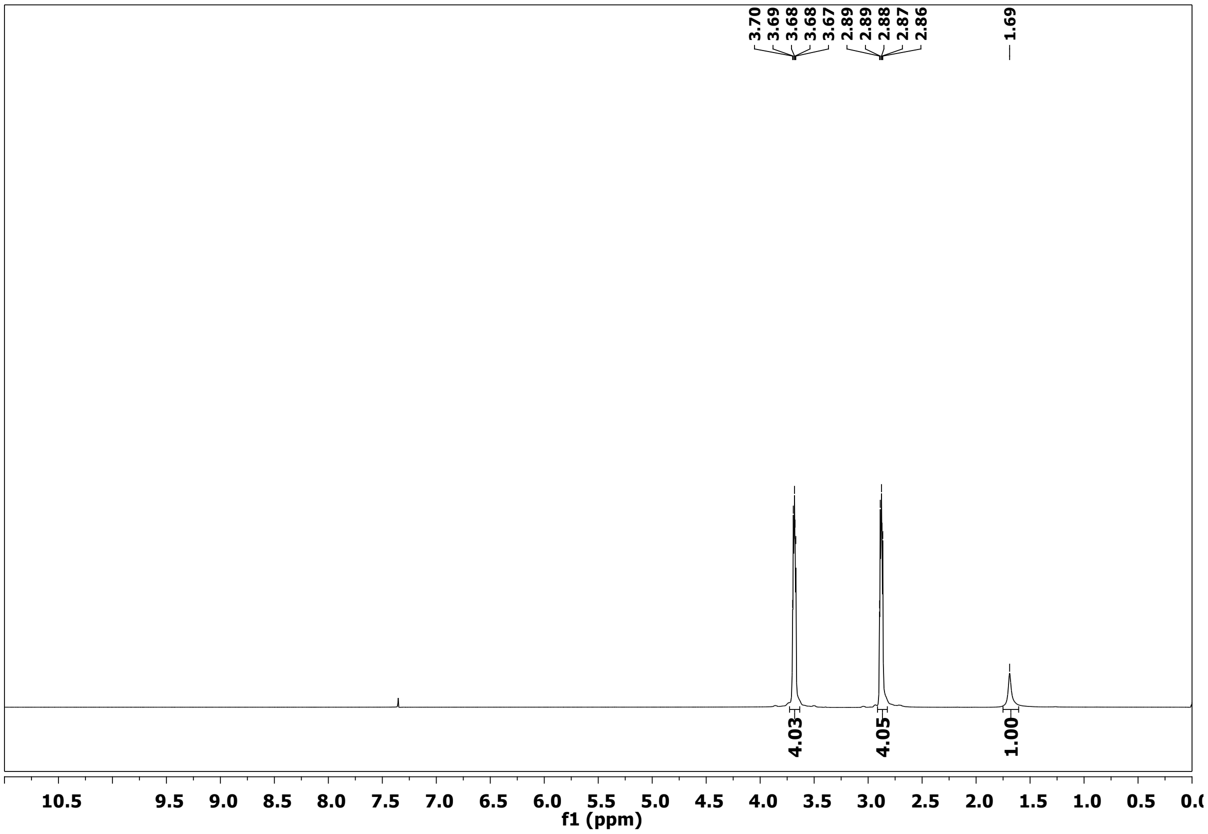

*Figure S12. ^{1}H NMR of Morpholine **(7a)** ($CDCl_3$), 400 MHz.*

Piperidine (8a)

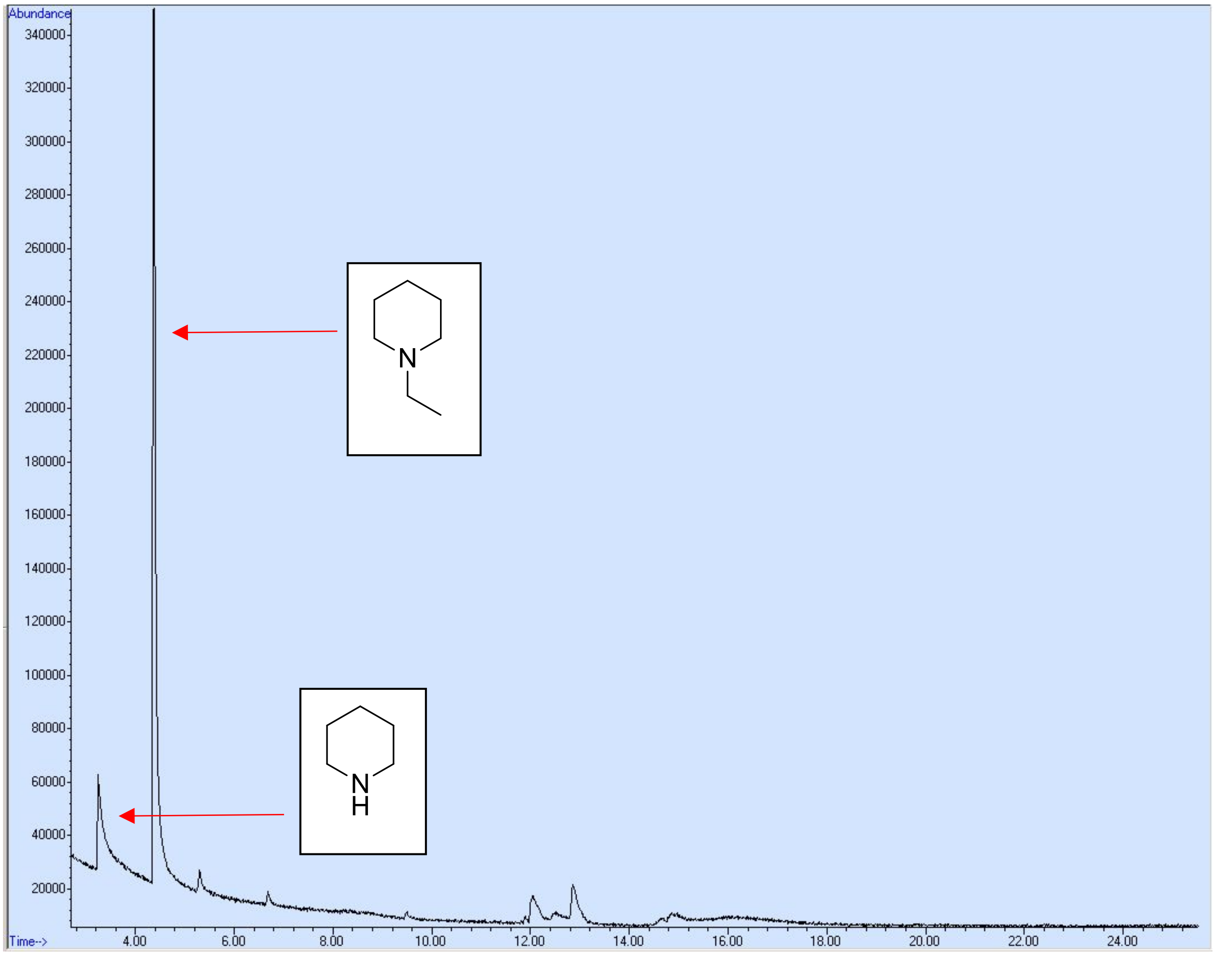

*Figure S13. GC-MS result for reaction with N-ethylpiperidine (**8**).*

Dibenzylamine ($C_{14}H_{15}N$, 9a)

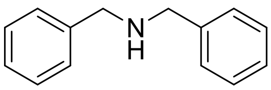

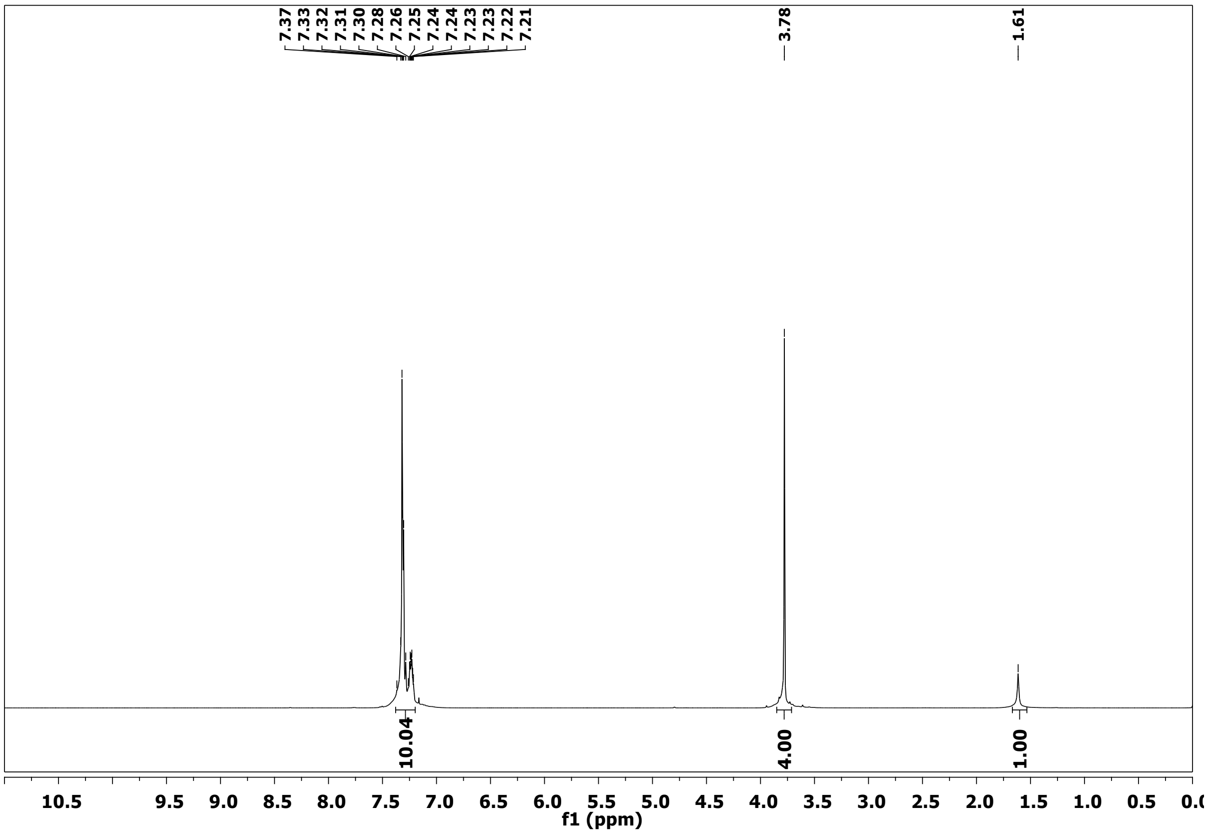

*Figure S14. ^{1}H NMR of dibenzylamine **(9a)** ($CDCl_3$), 400 MHz.*

N-Methylaniline (C_7H_9N, 10a)

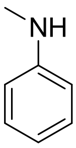

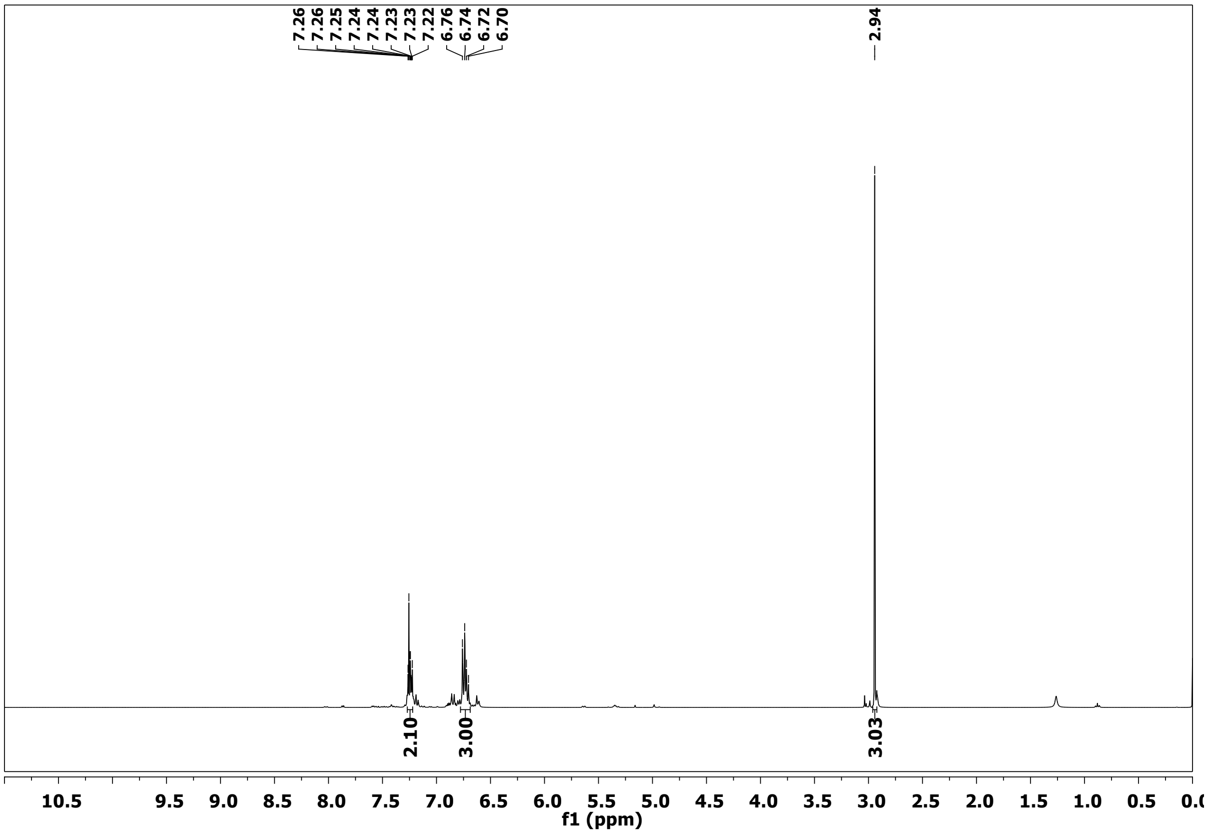

*Figure S15. ^1H NMR of N-methylaniline **(10a)** ($CDCl_3$), 400 MHz.*

N-Ethylaniline ($C_8H_{11}N$, 11a)

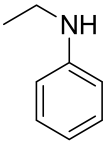

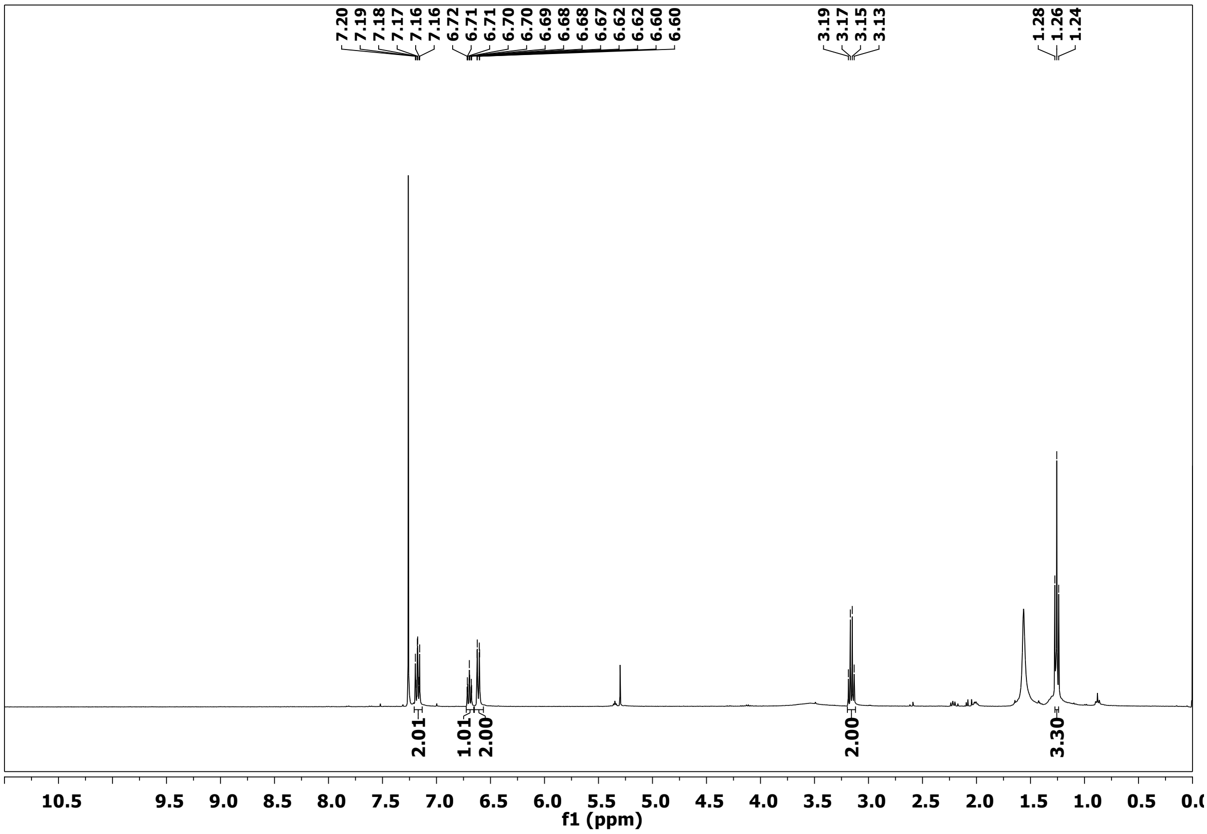

Figure S16. [1]*H NMR of N-ethylaniline* ***(11a)*** *($CDCl_3$), 400 MHz.*

N-Propylaniline ($C_9H_{13}N$, 12a)

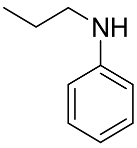

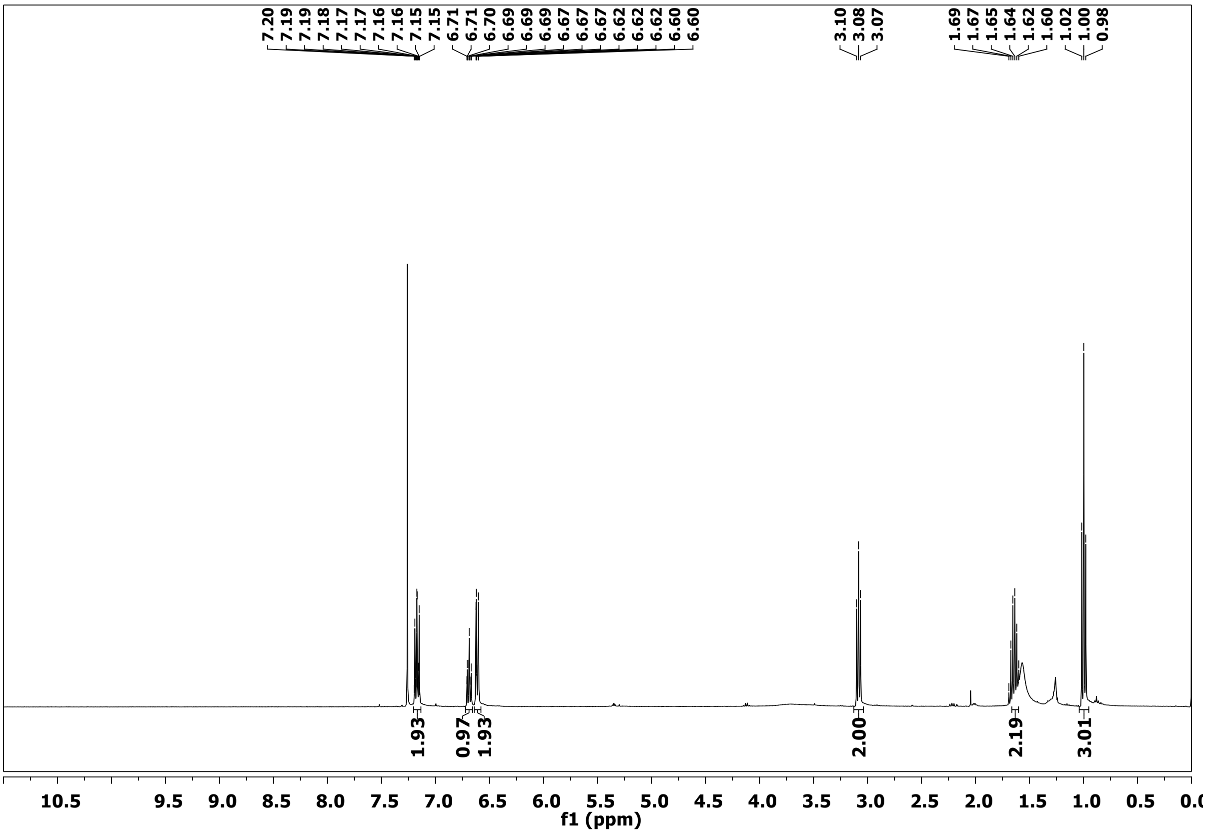

*Figure S17. ^{1}H NMR of N-propylaniline **(12a)** ($CDCl_3$), 400 MHz.*

N,3-Dimethylaniline ($C_8H_{11}N$, 15a)

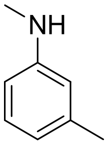

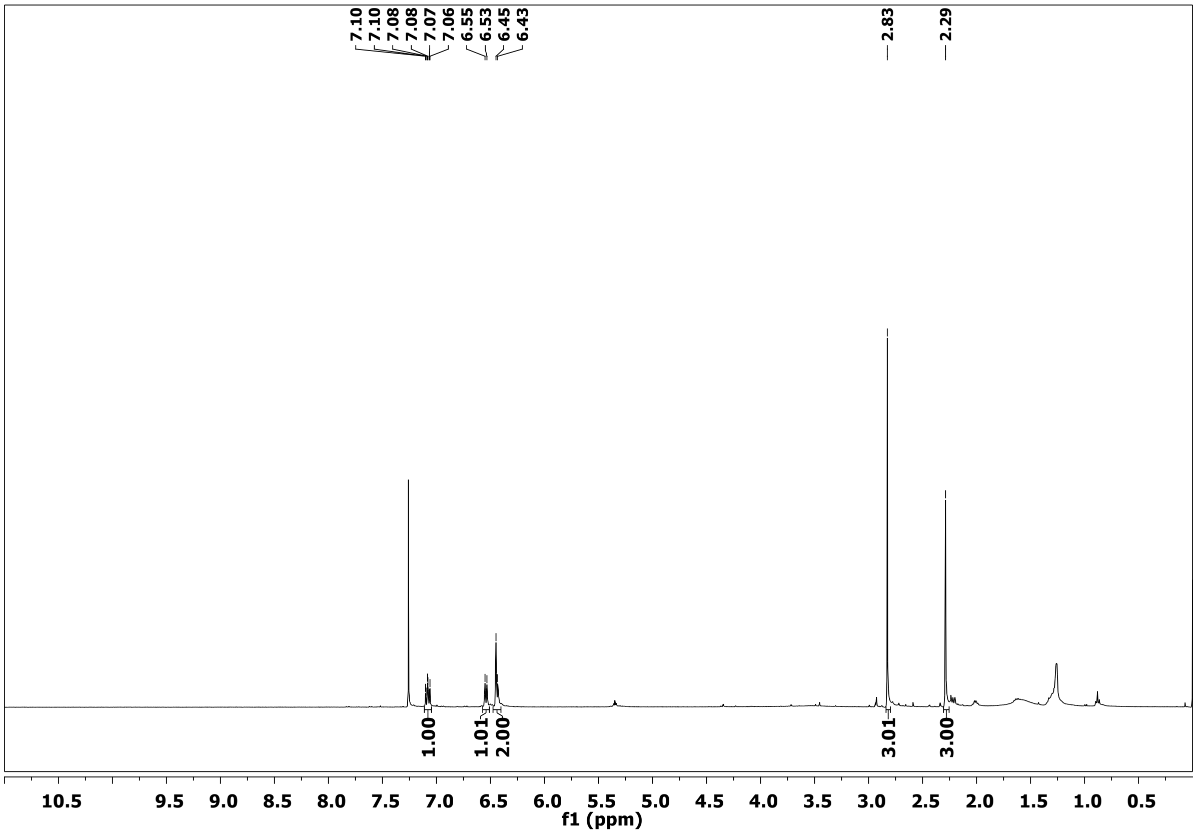

*Figure S18. ^{1}H NMR of N,3-dimethylaniline **(13a)** ($CDCl_3$), 400 MHz.*

N,4-Dimethylaniline ($C_8H_{11}N$, 16a)

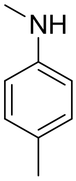

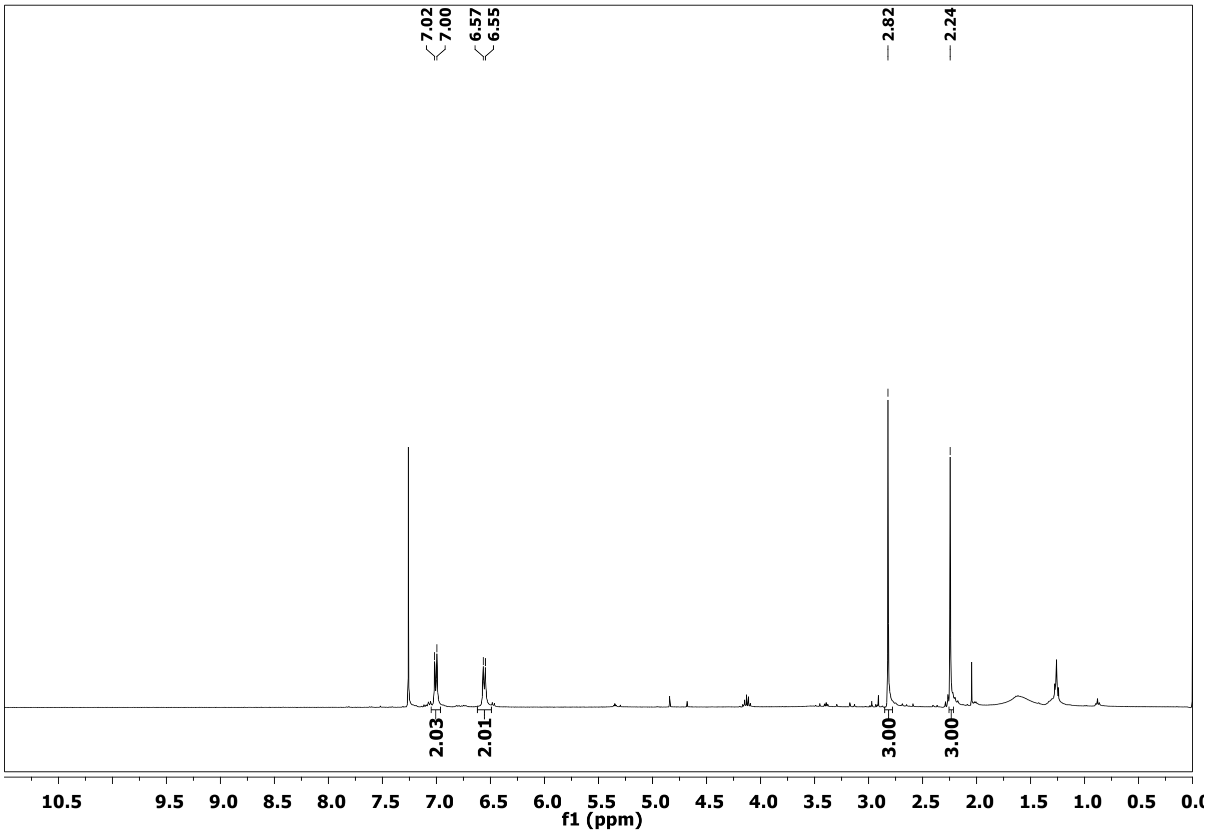

Figure S19. ^{1}H NMR of N,4-dimethylaniline ***(14a)*** *($CDCl_3$), 400 MHz.*

1*H*-indole-3-carbaldehyde (C_9H_7NO, 17a)

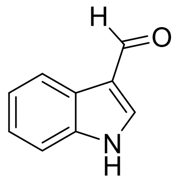

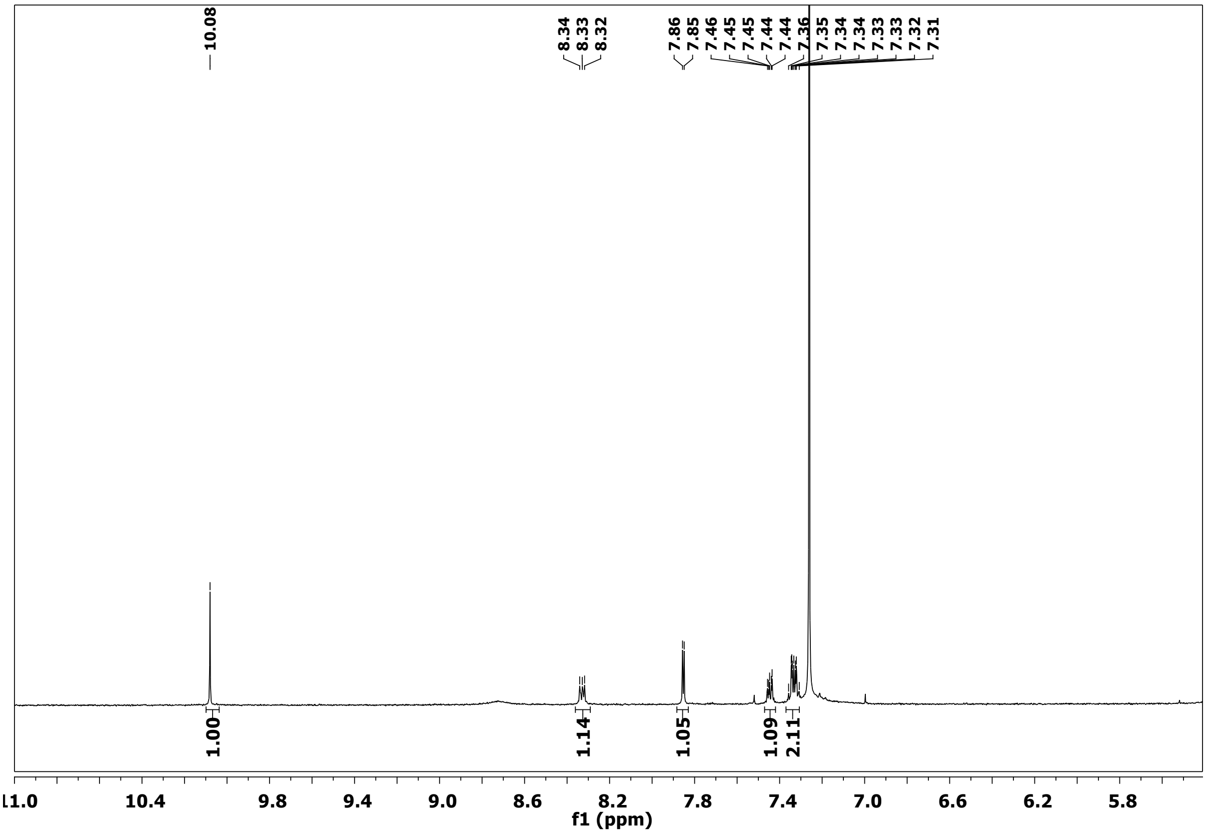

*Figure S20. ^{1}H NMR of 1H-indole-3-carbaldehyde **(17a)** (CDCl$_3$), 400 MHz.*

(E)-6,6-dimethyl-*N*-(naphthalen-1-ylmethyl)hept-2-en-4-yn-1-amine ($C_{20}H_{23}N$, 18a)

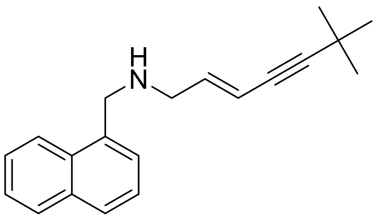

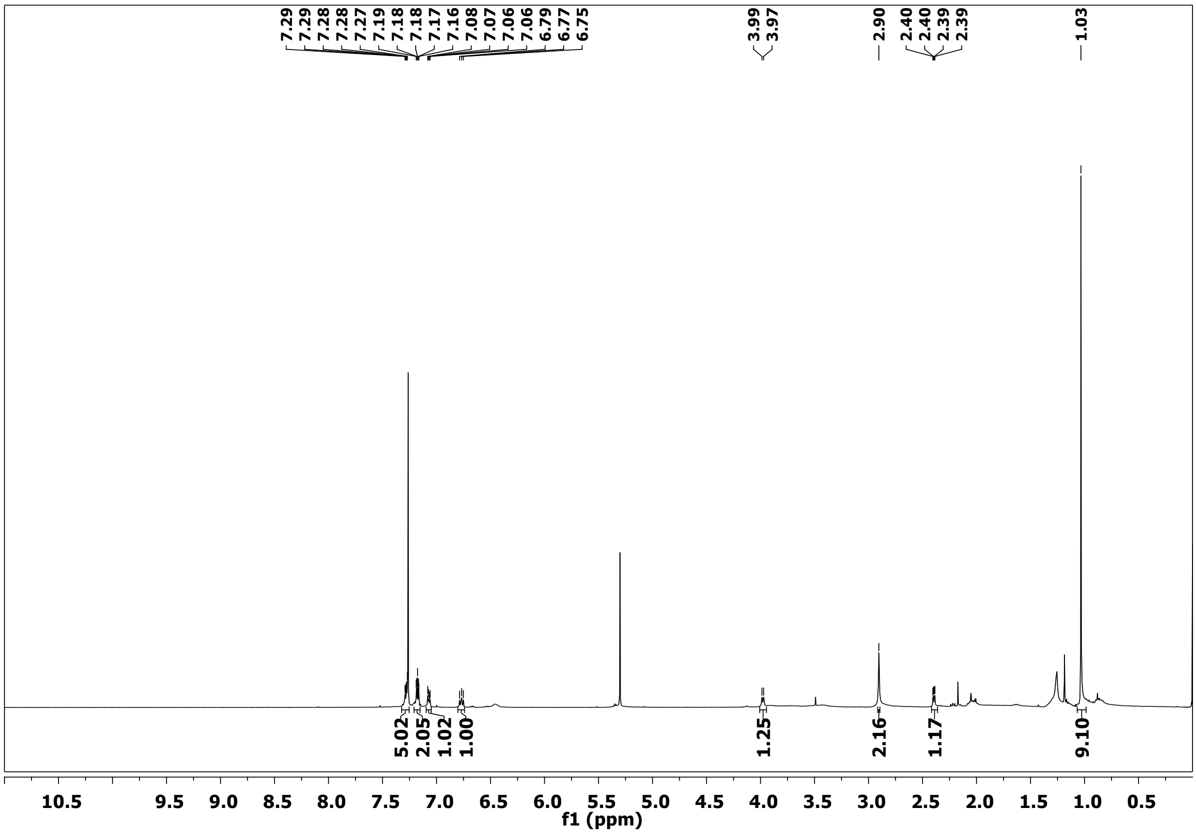

*Figure S21. ^{1}H NMR of (E)-6,6-dimethyl-N-(naphthalen-1-ylmethyl)hept-2-en-4-yn-1-amine **(18a)** ($CDCl_3$), 400 MHz.*

2-(benzhydryloxy)-N-methylethanamine ($C_{16}H_{19}NO$, 19a)

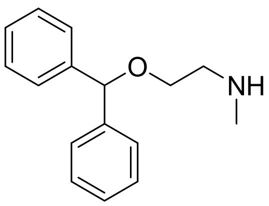

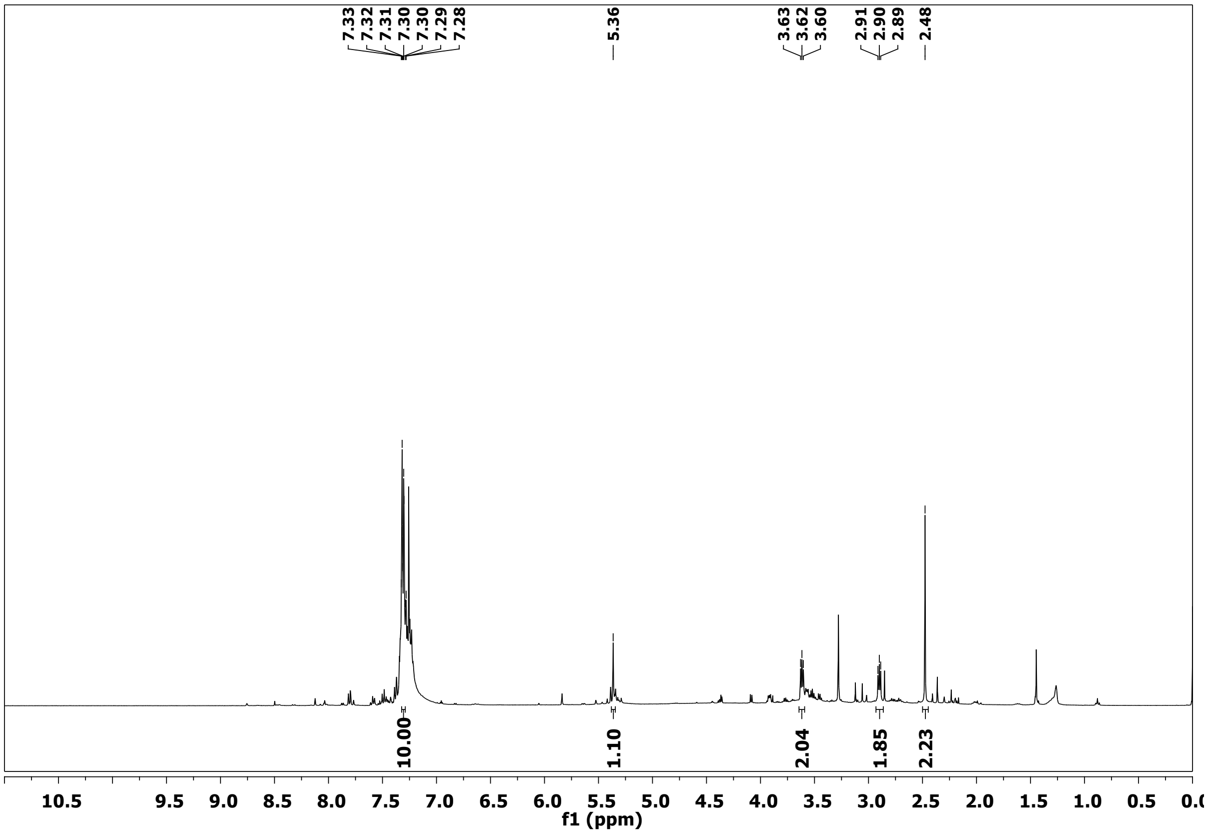

*Figure S22. . ^{1}H-NMR spectrum of crude mixture after N-dealkylation reaction of 2-(benzhydryloxy)-N,N-dimethylethanamine (**19**) ($CDCl_3$). The spectral data were in agreement with literature data, 400 MHz.*

10,11-Dihydro-5H-dibenzo[b,f]azepine ($C_{14}H_{13}N$, 20a)

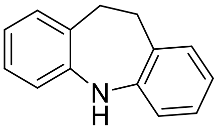

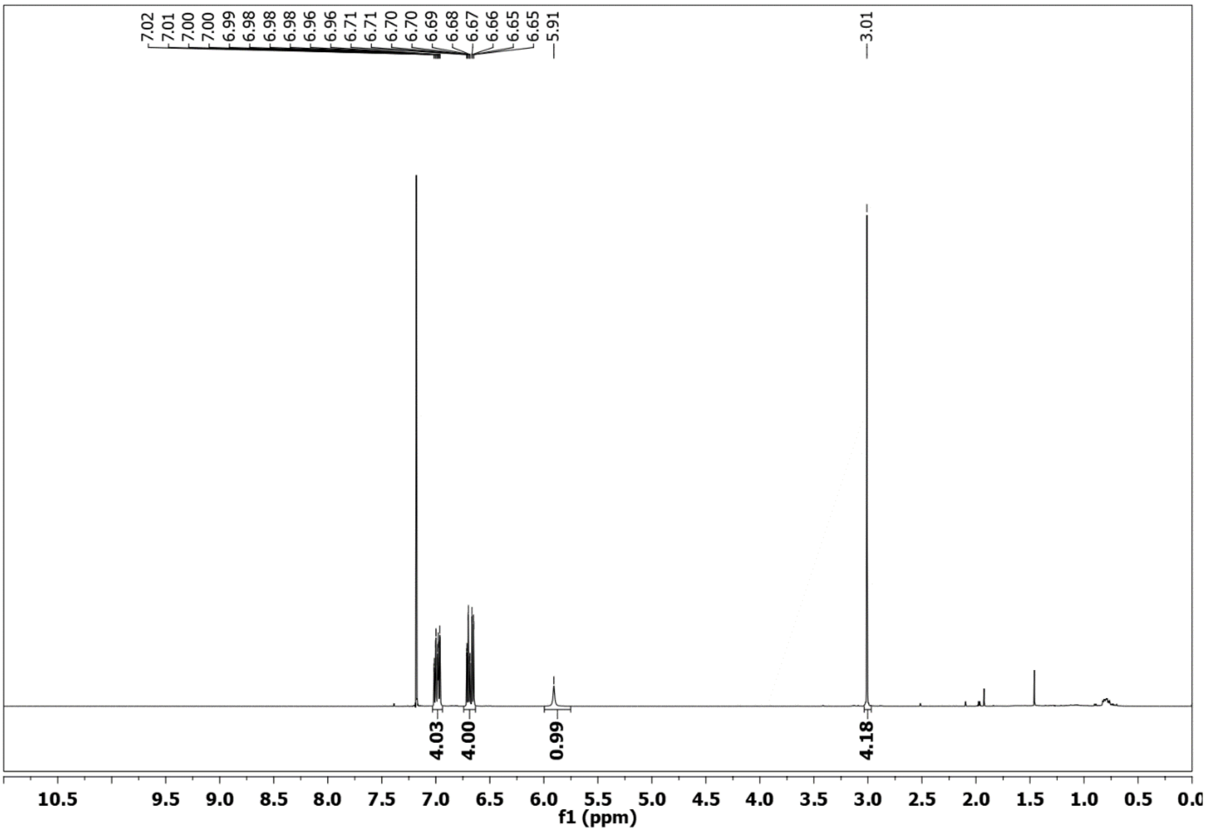

*Figure S23. ^{1}H NMR of 10,11-Dihydro-5H-dibenzo[b,f]azepine **(20a)** ($CDCl_3$), 400 MHz.*

3-(2,6-Dimethylphenyl)-1-ethyl-2-methylimidazolidin -4-one ($C_{14}H_{20}NO_2$, 21a)

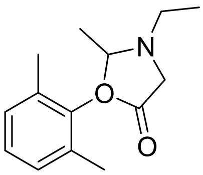

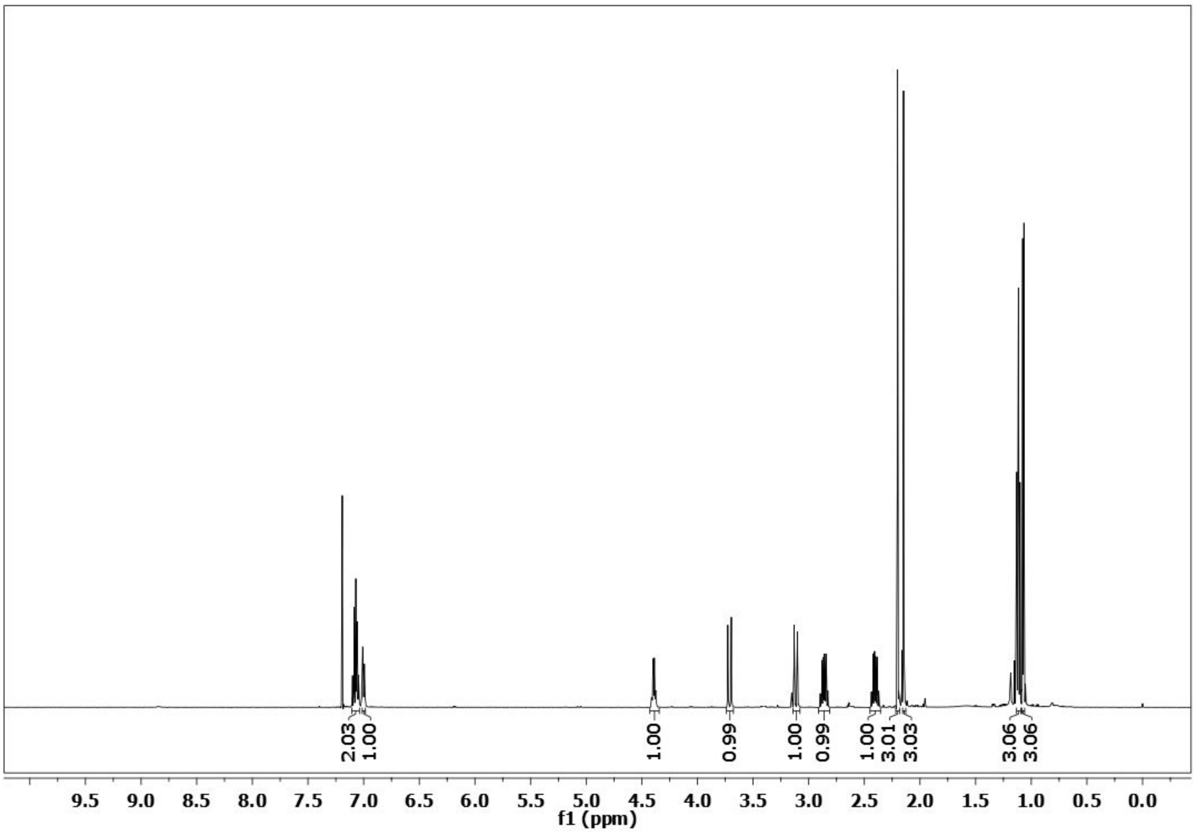

*Figure S24. ^{1}H NMR spectrum of 3-(2,6-dimethylphenyl)-1-ethyl-2-methylimidazolidin-4-one **(21a)** ($CDCl_3$), 400 MHz.*

Data of reactions examined by LCAP (liquid chromatography area percent)

Reaction with (3S,4R)-methyl 1-benzyl-4-phenylpyrrolidine-3-carboxylate (23)

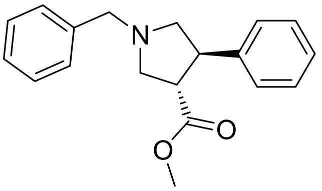

23

m/z: 295.16 (100.0%), 296.16 (20.9%), 297.16 (2.5%)

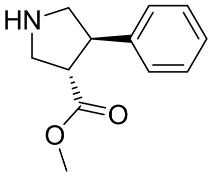

23a

m/z: 205.11 (100.0%), 206.11 (13.4%)

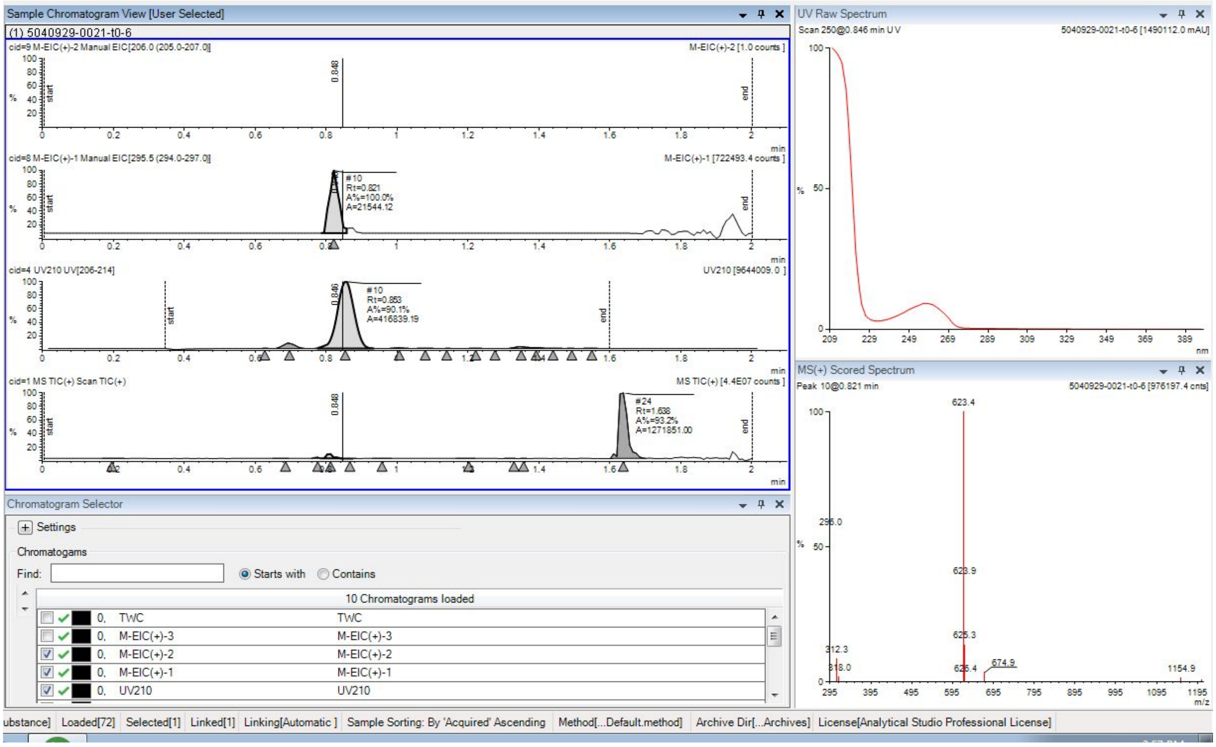

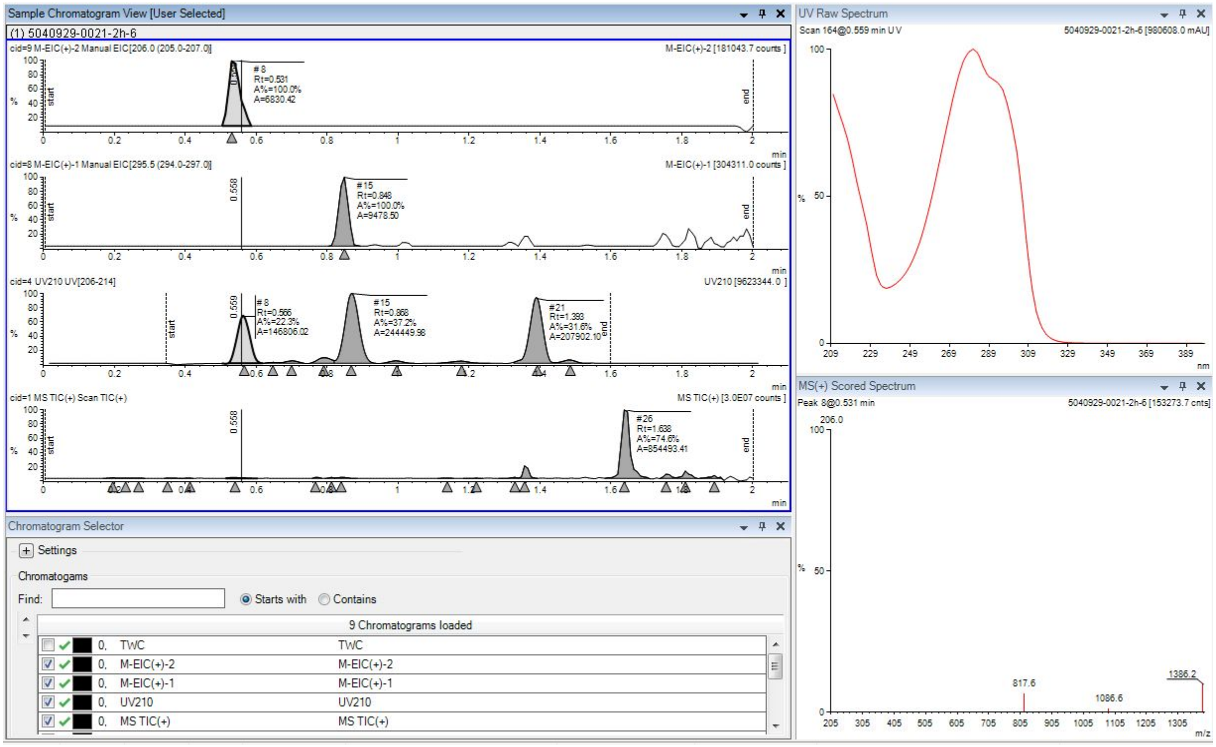
Sample Chromatogram View [User Selected]
(1) 5040929-0021-2h-6
cid=9 M-EIC(+)-2 Manual EIC[206.0 (205.0-207.0)]
M-EIC(+)-2 [181043.7 counts]
cid=8 M-EIC(+)-1 Manual EIC[295.5 (294.0-297.0)]
M-EIC(+)-1 [304311.0 counts]
cid=4 UV210 UV[206-214]
UV210 [9623344.0]
cid=1 MS TIC(+) Scan TIC(+)
MS TIC(+) [3.0E07 counts]
UV Raw Spectrum
Scan 164@0.559 min UV
5040929-0021-2h-6 [980608.0 mAU]
MS(+) Scored Spectrum
Peak 8@0.531 min
5040929-0021-2h-6 [153273.7 cnts]
206.0
817.6
1086.6
1386.2
Chromatogram Selector
Settings
Chromatogams
Find:
Starts with
Contains
9 Chromatograms loaded
0, TWC TWC
0, M-EIC(+)-2 M-EIC(+)-2
0, M-EIC(+)-1 M-EIC(+)-1
0, UV210 UV210
0, MS TIC(+) MS TIC(+)

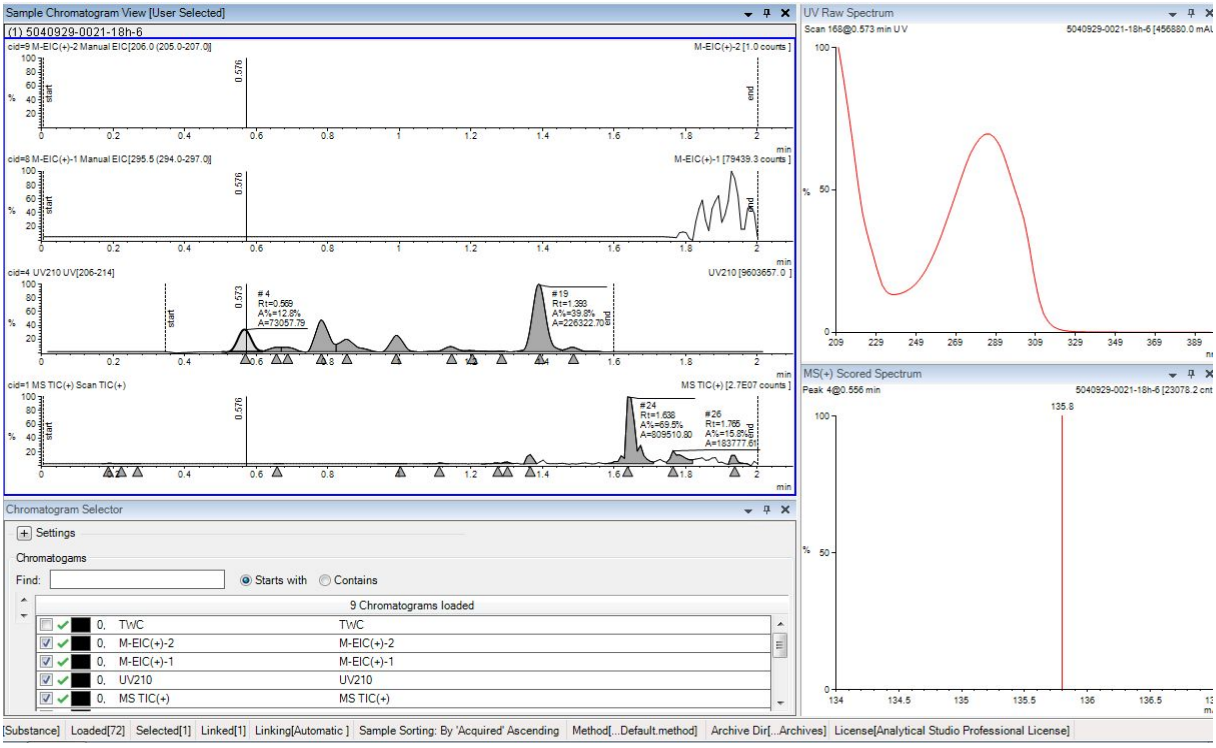
Sample Chromatogram View [User Selected]
(1) 5040929-0021-18h-6
cid=9 M-EIC(+)-2 Manual EIC[206.0 (205.0-207.0)]
M-EIC(+)-2 [1.0 counts]
cid=8 M-EIC(+)-1 Manual EIC[295.5 (294.0-297.0)]
M-EIC(+)-1 [79439.3 counts]
cid=4 UV210 UV[206-214]
UV210 [9603657.0]
cid=1 MS TIC(+) Scan TIC(+)
MS TIC(+) [2.7E07 counts]
UV Raw Spectrum
Scan 168@0.573 min UV
MS(+) Scored Spectrum
Peak 4@0.556 min
135.8
Chromatogram Selector
Settings
Chromatogams
Find:
Starts with
Contains
9 Chromatograms loaded
0, TWC TWC
0, M-EIC(+)-2 M-EIC(+)-2
0, M-EIC(+)-1 M-EIC(+)-1
0, UV210 UV210
0, MS TIC(+) MS TIC(+)
[Substance] Loaded[72] Selected[1] Linked[1] Linking[Automatic] Sample Sorting: By 'Acquired' Ascending Method[...Default.method] Archive Dir[...Archives] License[Analytical Studio Professional License]

Reaction with (*E*)-ethyl 2-((dimethylamino)methylene)-3-oxobutanoate (24)

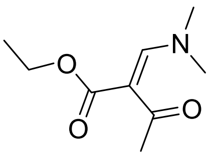

24

m/z: 185.11 (100.0%), 186.11 (10.0%), 187.11 (1.1%)

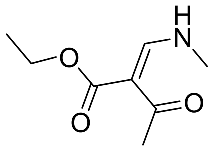

24a

m/z: 171.09 (100.0%), 172.09 (9.1%)

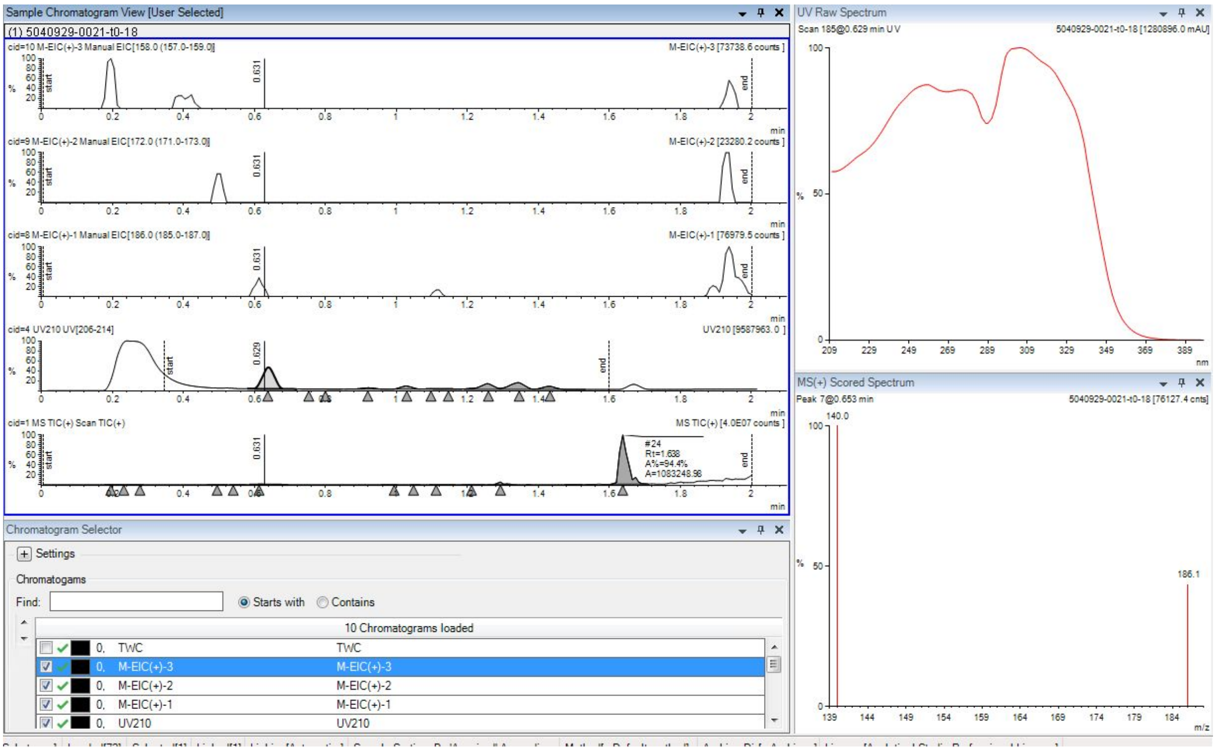

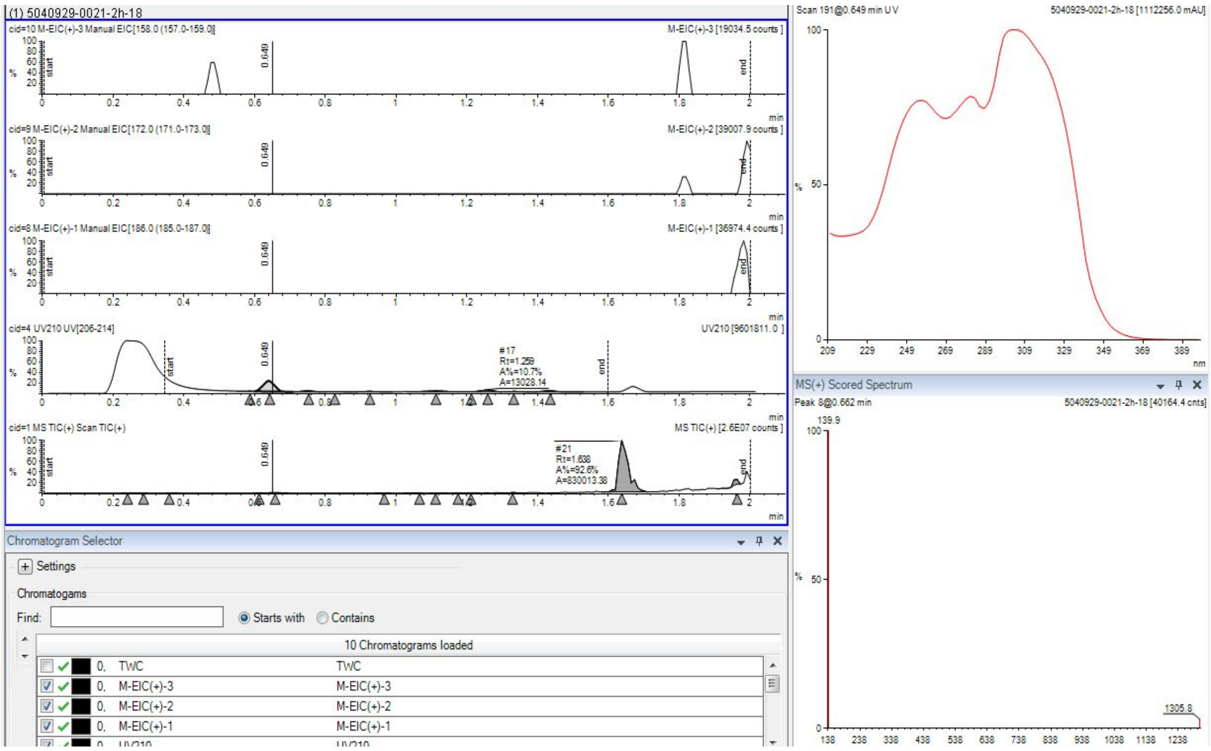
(1) 5040929-0021-2h-18
Scan 191@0.649 min UV
5040929-0021-2h-18 [1112256.0 mAU]
MS(+) Scored Spectrum
Peak 8@0.662 min
5040929-0021-2h-18 [40164.4 cnts]
139.9
1305.8
Chromatogram Selector
Settings
Chromatogams
Find:
Starts with
Contains
10 Chromatograms loaded

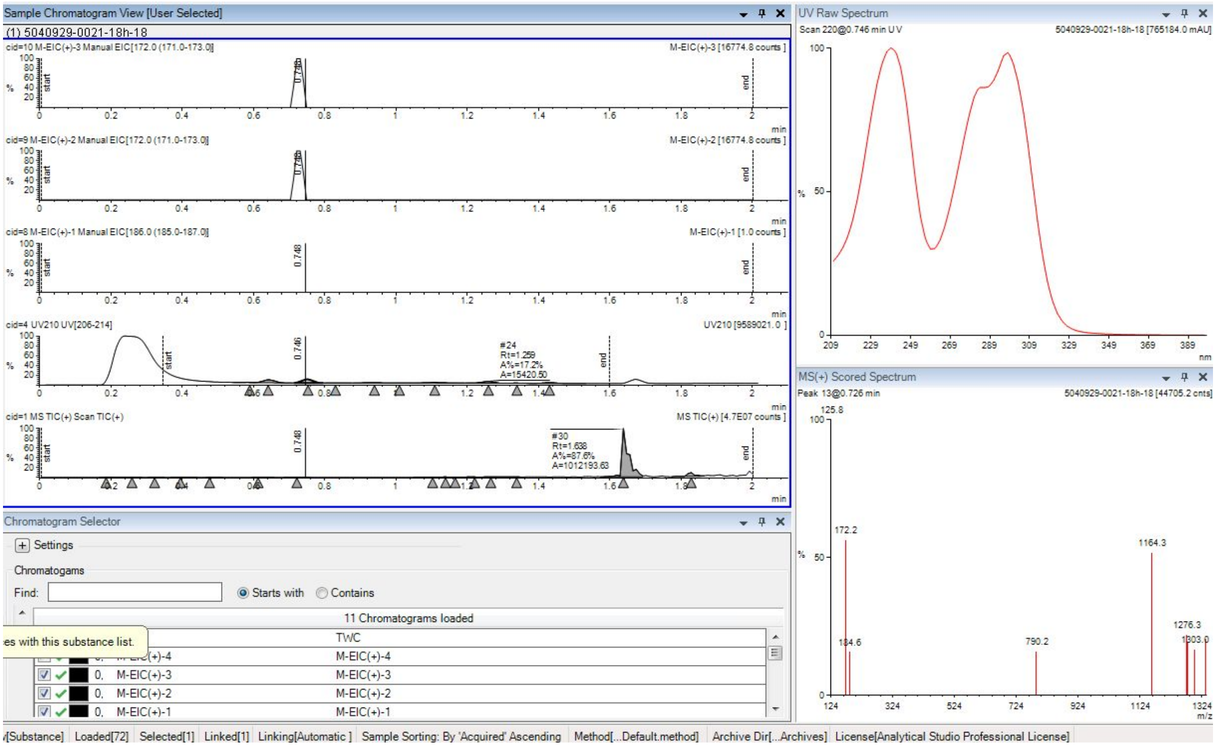
Sample Chromatogram View [User Selected]
(1) 5040929-0021-18h-18
UV Raw Spectrum
Scan 220@0.746 min UV
5040929-0021-18h-18 [765184.0 mAU]
MS(+) Scored Spectrum
Peak 13@0.726 min
5040929-0021-18h-18 [44705.2 cnts]
125.8
172.2
184.6
790.2
1164.3
1276.3
1303.0
Chromatogram Selector
Settings
Chromatogams
Find:
Starts with
Contains
11 Chromatograms loaded

Reaction with 1-benzyl-4-phenylpiperidine-4-carbonitrile (25)

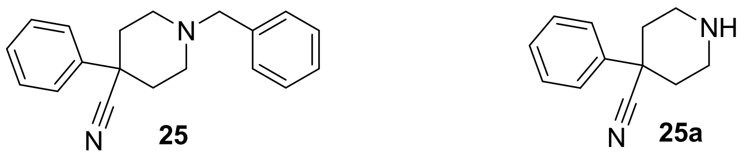

m/z: 276.16 (100.0%), 277.17 (20.8%), 278.17 (2.0%)

m/z: 186.12 (100.0%), 187.12 (13.1%)

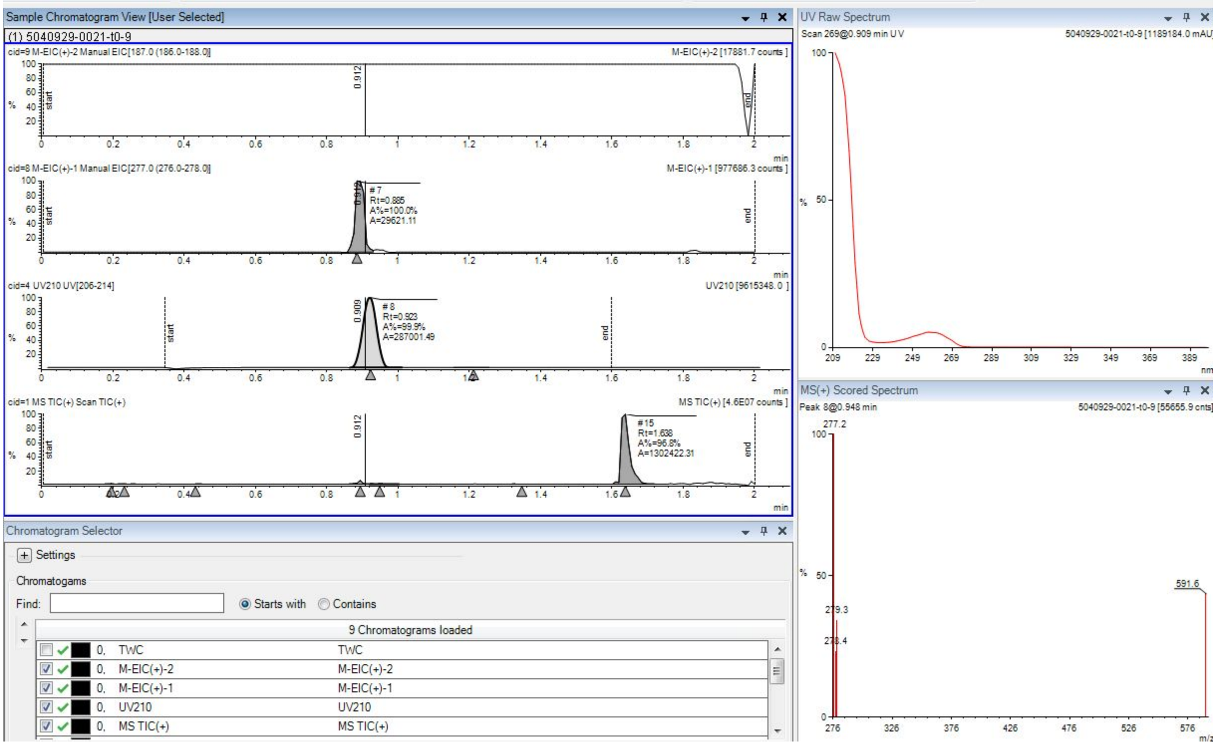

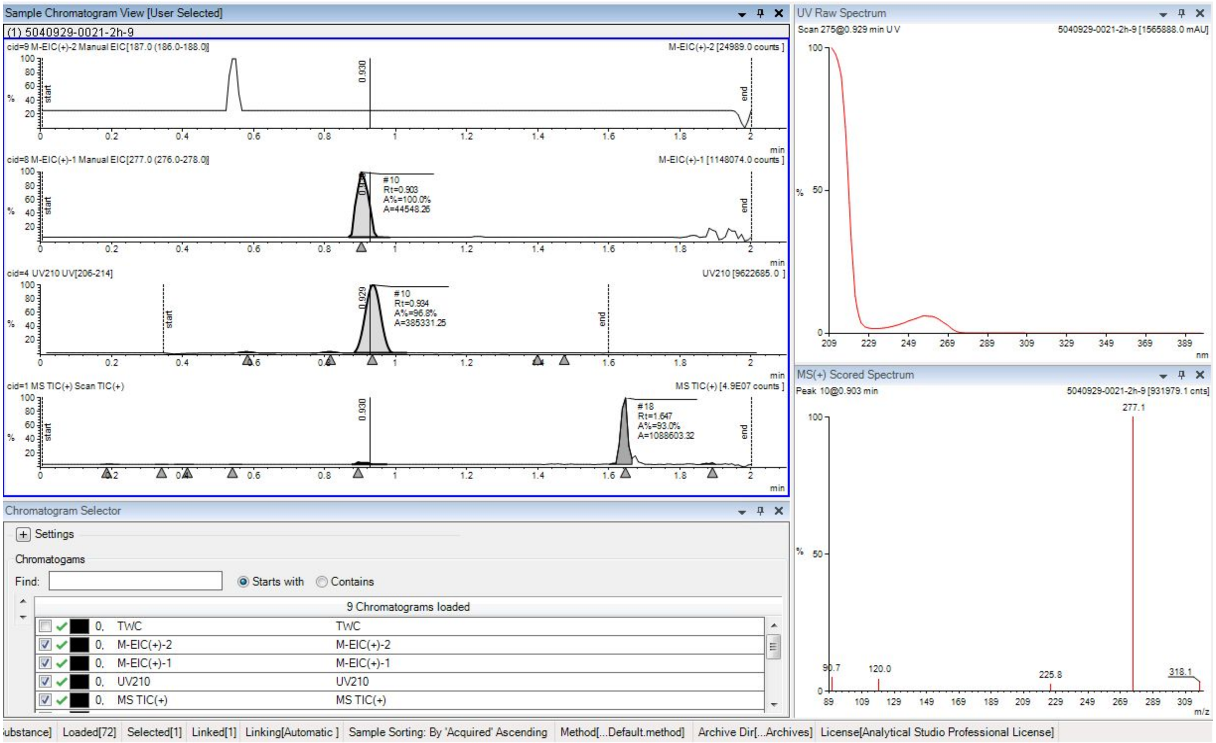

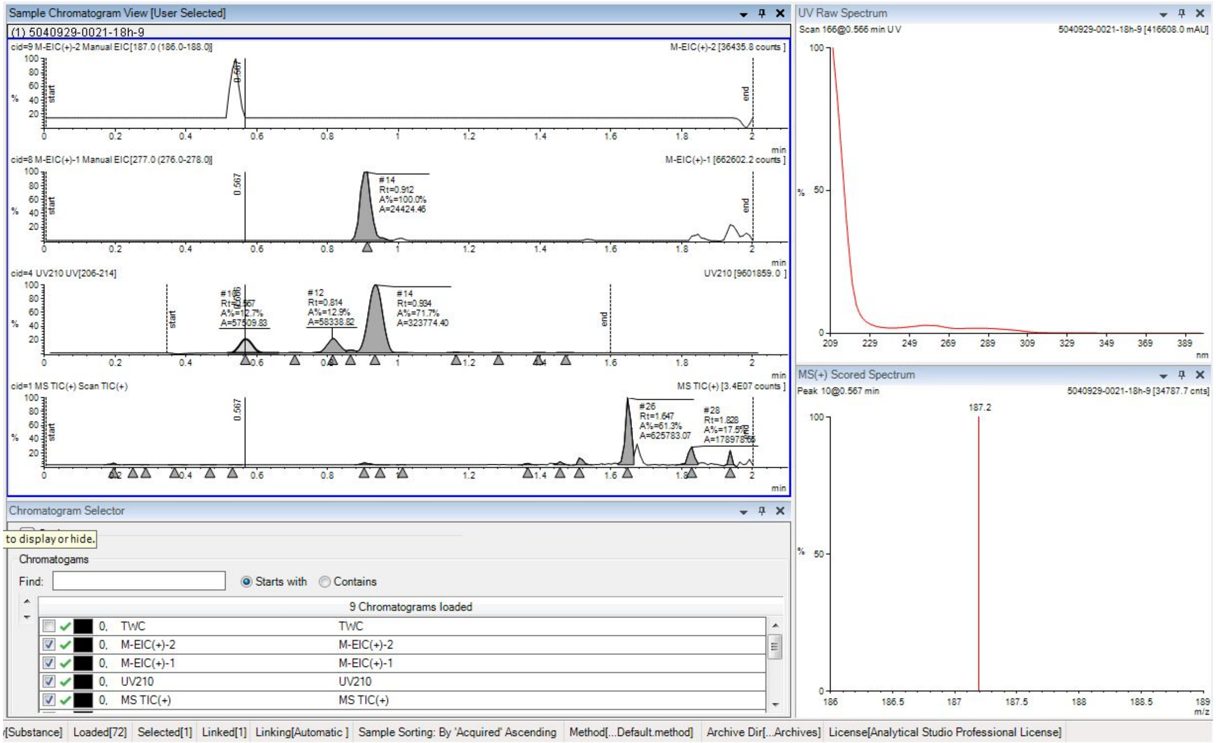

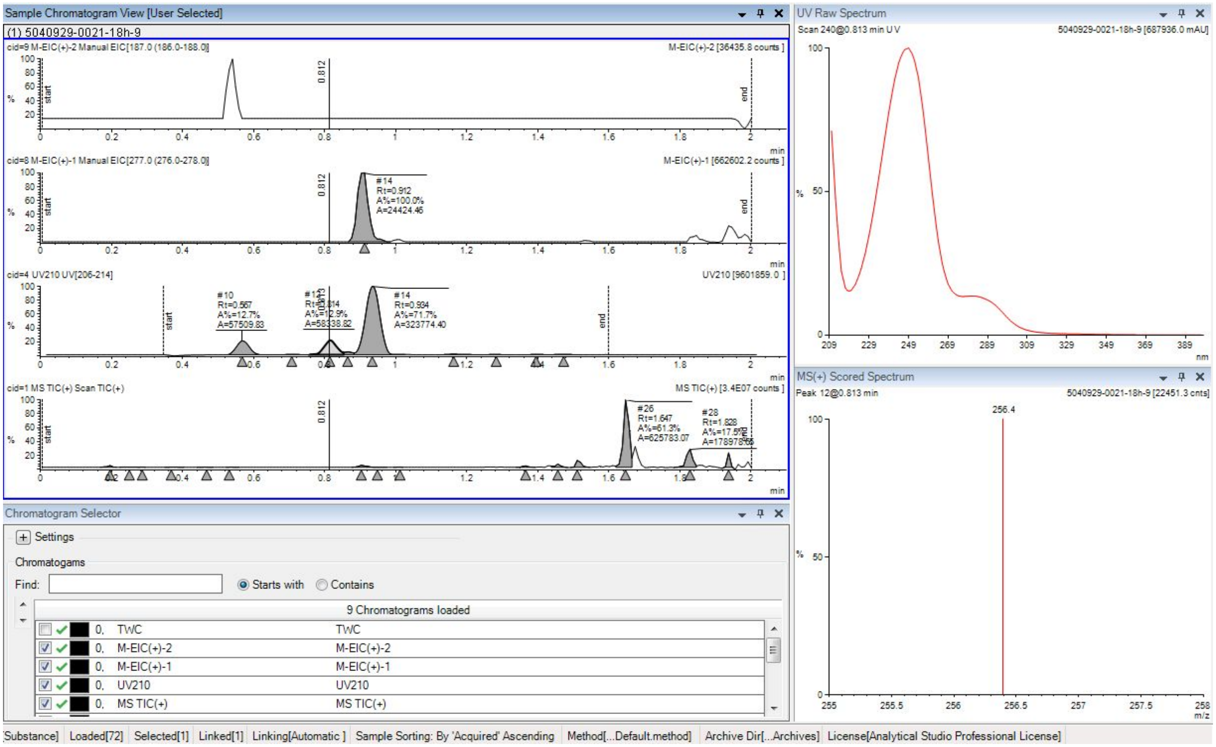

Reaction with 1-(3,5-bis(trifluoromethyl)phenyl)-3-((1R,2R)-2-(dimethylamino)cyclohexyl)thiourea (26)

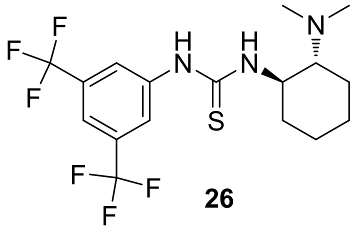

26

m/z: 413.14 (100.0%), 414.14 (19.4%), 415.13 (4.5%), 415.14 (1.9%), 414.13 (1.1%)

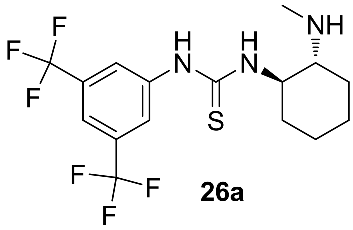

26a

m/z: 399.12 (100.0%), 400.12 (19.2%), 401.12 (4.9%), 401.13 (1.4%)

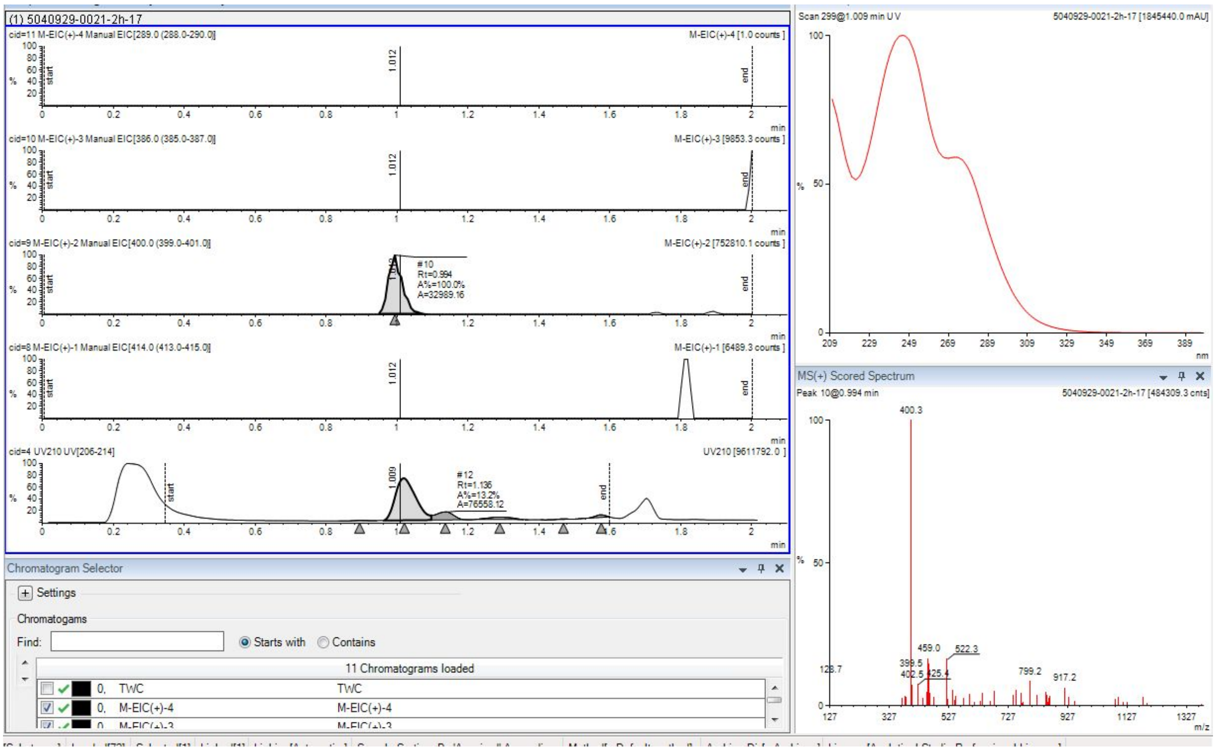

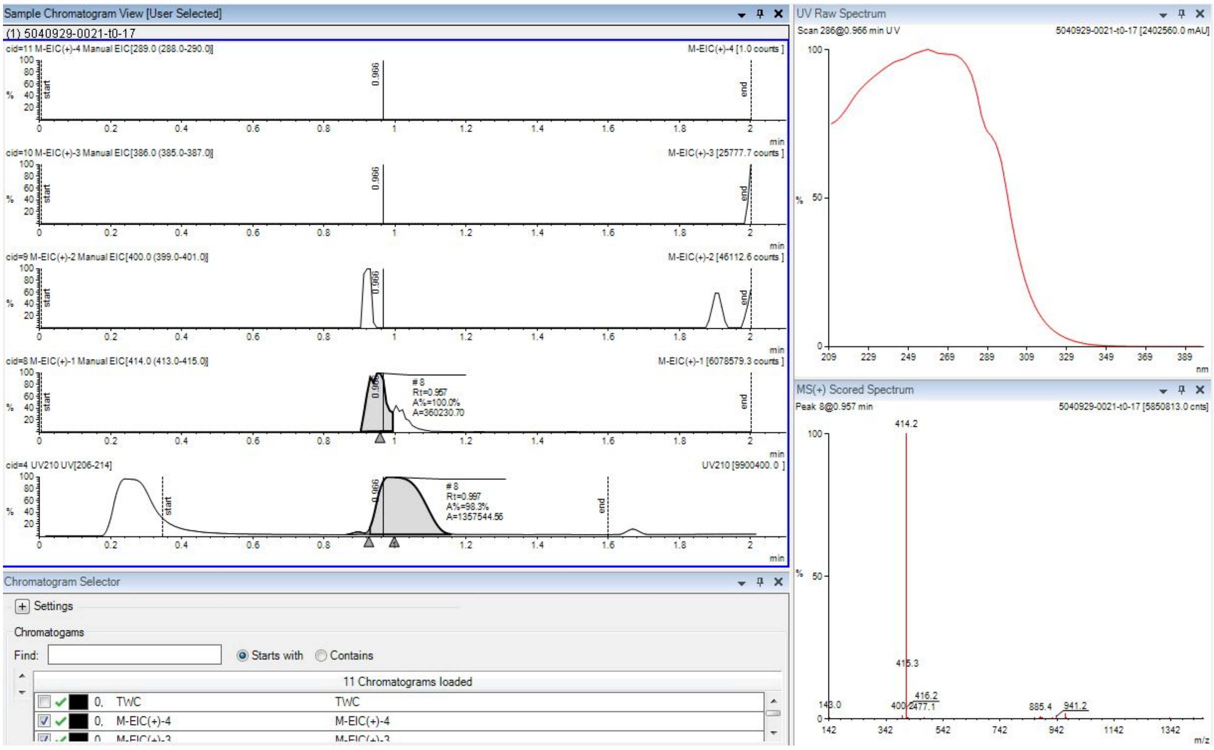
Sample Chromatogram View [User Selected]
(1) 5040929-0021-t0-17
cid=11 M-EIC(+)-4 Manual EIC[289.0 (288.0-290.0)]
cid=10 M-EIC(+)-3 Manual EIC[386.0 (385.0-387.0)]
cid=9 M-EIC(+)-2 Manual EIC[400.0 (399.0-401.0)]
cid=8 M-EIC(+)-1 Manual EIC[414.0 (413.0-415.0)]
cid=4 UV210 UV[206-214]
UV Raw Spectrum
MS(+) Scored Spectrum
414.2
Chromatogram Selector
11 Chromatograms loaded

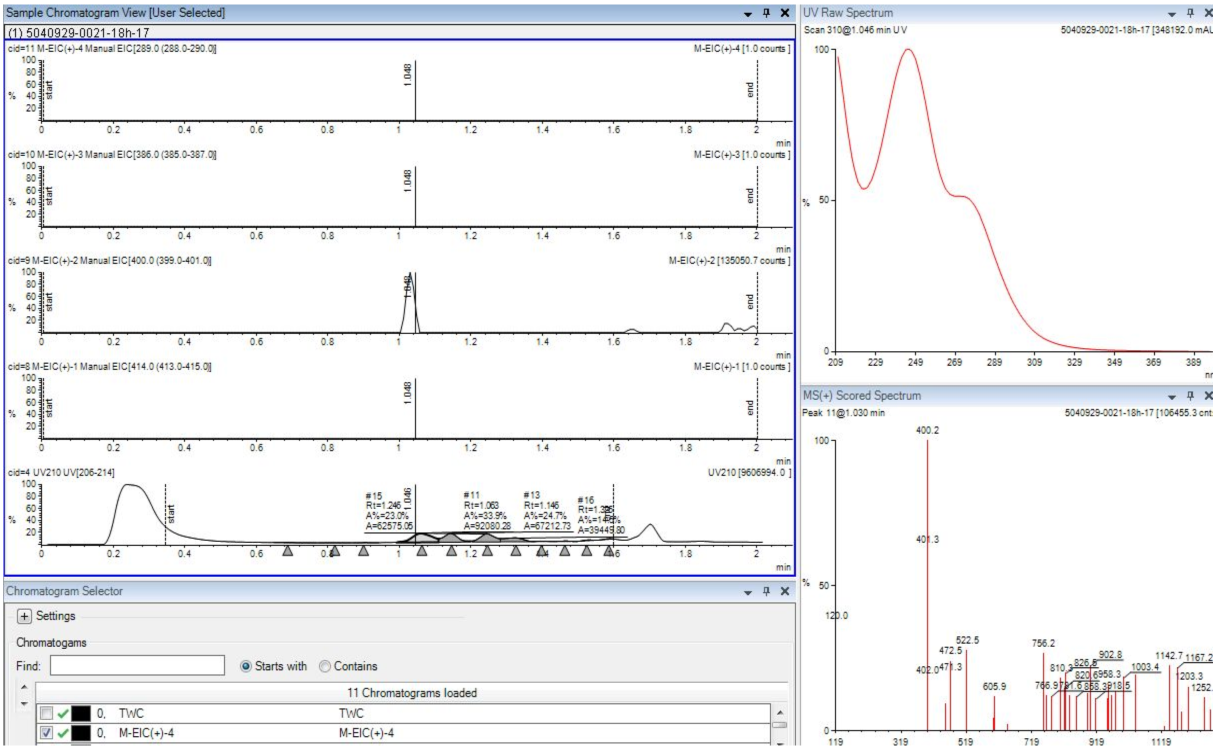
Sample Chromatogram View [User Selected]
(1) 5040929-0021-18h-17
cid=11 M-EIC(+)-4 Manual EIC[289.0 (288.0-290.0)]
cid=10 M-EIC(+)-3 Manual EIC[386.0 (385.0-387.0)]
cid=9 M-EIC(+)-2 Manual EIC[400.0 (399.0-401.0)]
cid=8 M-EIC(+)-1 Manual EIC[414.0 (413.0-415.0)]
cid=4 UV210 UV[206-214]
UV Raw Spectrum
MS(+) Scored Spectrum
400.2
Chromatogram Selector
11 Chromatograms loaded

Reaction with 2-(4-chlorophenyl)-1-(4-(2-(diethylamino)ethoxy)phenyl)-1-(p-tolyl)ethanol (27)

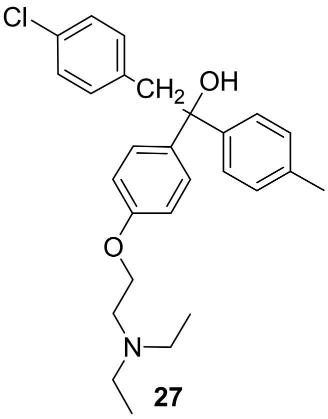

27

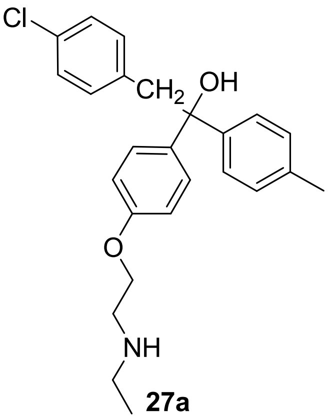

27a

m/z: 437.21 (100.0%), 439.21 (32.1%), 438.22 (29.6%), 440.21 (9.5%), 439.22 (4.6%), 441.22 (1.4%)

m/z: 409.18 (100.0%), 411.18 (32.1%), 410.18 (27.4%), 412.18 (8.8%), 411.19 (4.0%), 413.18 (1.3%)

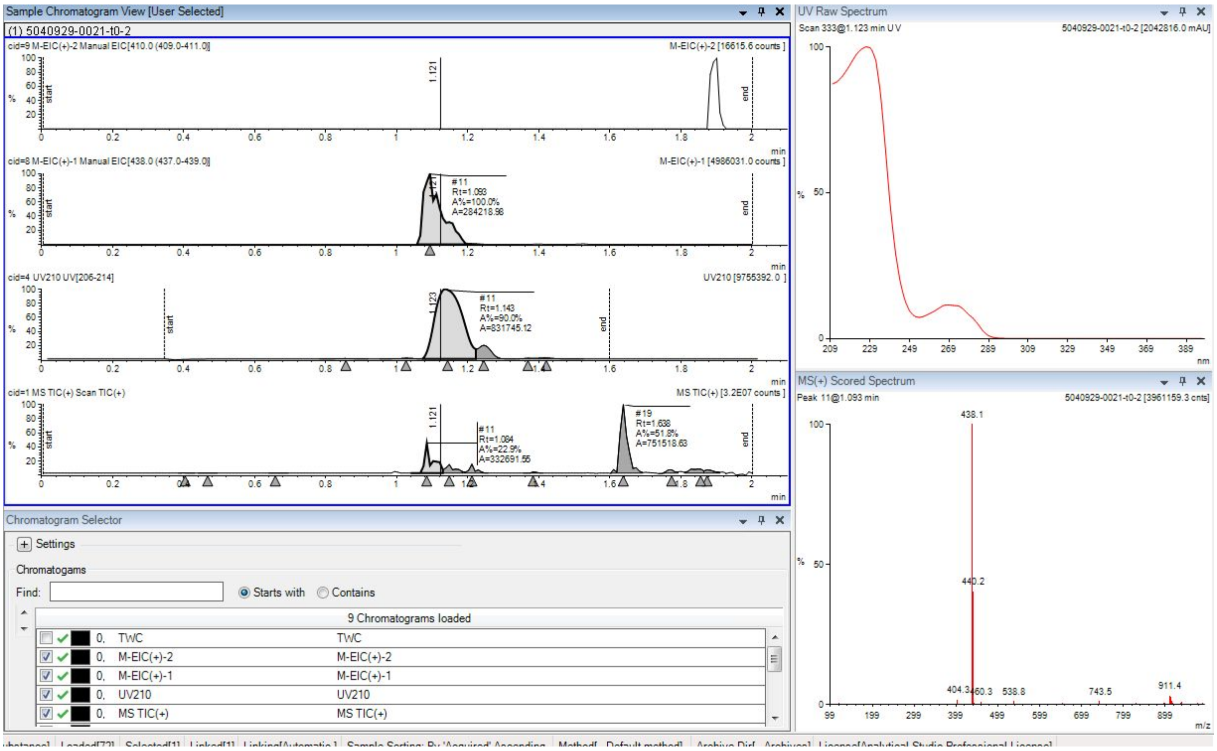

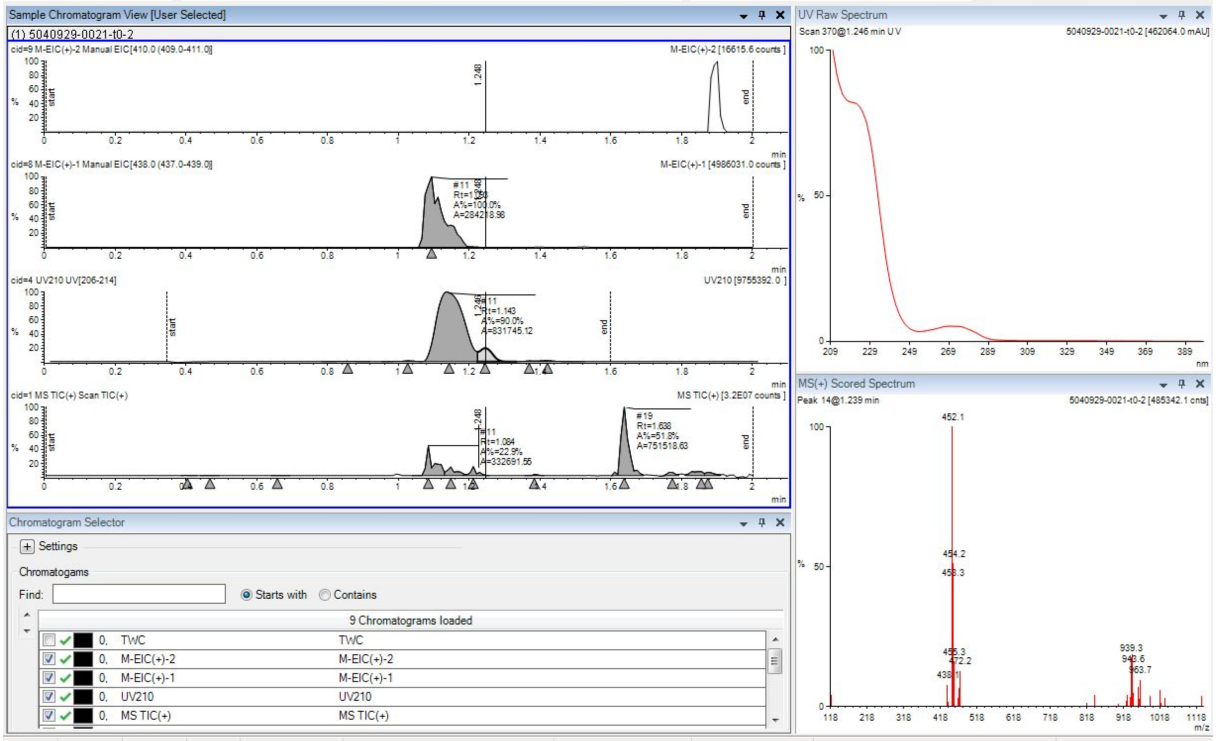
Sample Chromatogram View [User Selected]
(1) 5040929-0021-t0-2
cid=9 M-EIC(+)-2 Manual EIC[410.0 (409.0-411.0)]
M-EIC(+)-2 [16615.6 counts]
cid=8 M-EIC(+)-1 Manual EIC[438.0 (437.0-439.0)]
M-EIC(+)-1 [4986031.0 counts]
cid=4 UV210 UV[206-214]
UV210 [9755392.0]
cid=1 MS TIC(+) Scan TIC(+)
MS TIC(+) [3.2E07 counts]
#19
Rt=1.638
A%=51.8%
A=751518.63
UV Raw Spectrum
Scan 370@1.246 min UV
5040929-0021-t0-2 [462064.0 mAU]
MS(+) Scored Spectrum
Peak 14@1.239 min
5040929-0021-t0-2 [485342.1 cnts]
452.1
939.3
Chromatogram Selector
Settings
Chromatogams
Find:
Starts with
Contains
9 Chromatograms loaded
TWC
M-EIC(+)-2
M-EIC(+)-1
UV210
MS TIC(+)

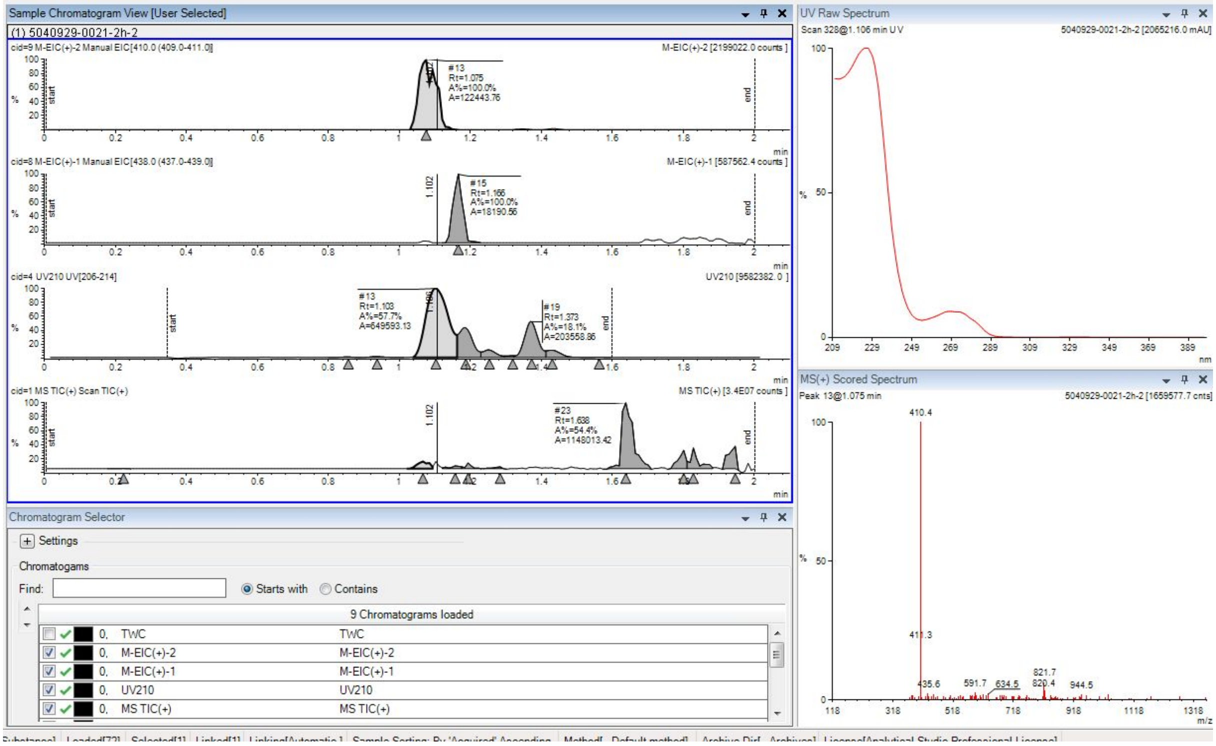
Sample Chromatogram View [User Selected]
(1) 5040929-0021-2h-2
cid=9 M-EIC(+)-2 Manual EIC[410.0 (409.0-411.0)]
M-EIC(+)-2 [2199022.0 counts]
#13
Rt=1.075
A%=100.0%
A=122443.76
cid=8 M-EIC(+)-1 Manual EIC[438.0 (437.0-439.0)]
M-EIC(+)-1 [587562.4 counts]
#15
Rt=1.166
A%=100.0%
A=18190.56
cid=4 UV210 UV[206-214]
UV210 [9582382.0]
#13
Rt=1.103
A%=57.7%
A=649593.13
#19
Rt=1.373
A%=18.1%
A=203558.86
cid=1 MS TIC(+) Scan TIC(+)
MS TIC(+) [3.4E07 counts]
#23
Rt=1.638
A%=54.4%
A=1148013.42
UV Raw Spectrum
Scan 328@1.106 min UV
5040929-0021-2h-2 [2065216.0 mAU]
MS(+) Scored Spectrum
Peak 13@1.075 min
5040929-0021-2h-2 [1659577.7 cnts]
410.4
Chromatogram Selector
Settings
Chromatogams
Find:
Starts with
Contains
9 Chromatograms loaded
TWC
M-EIC(+)-2
M-EIC(+)-1
UV210
MS TIC(+)

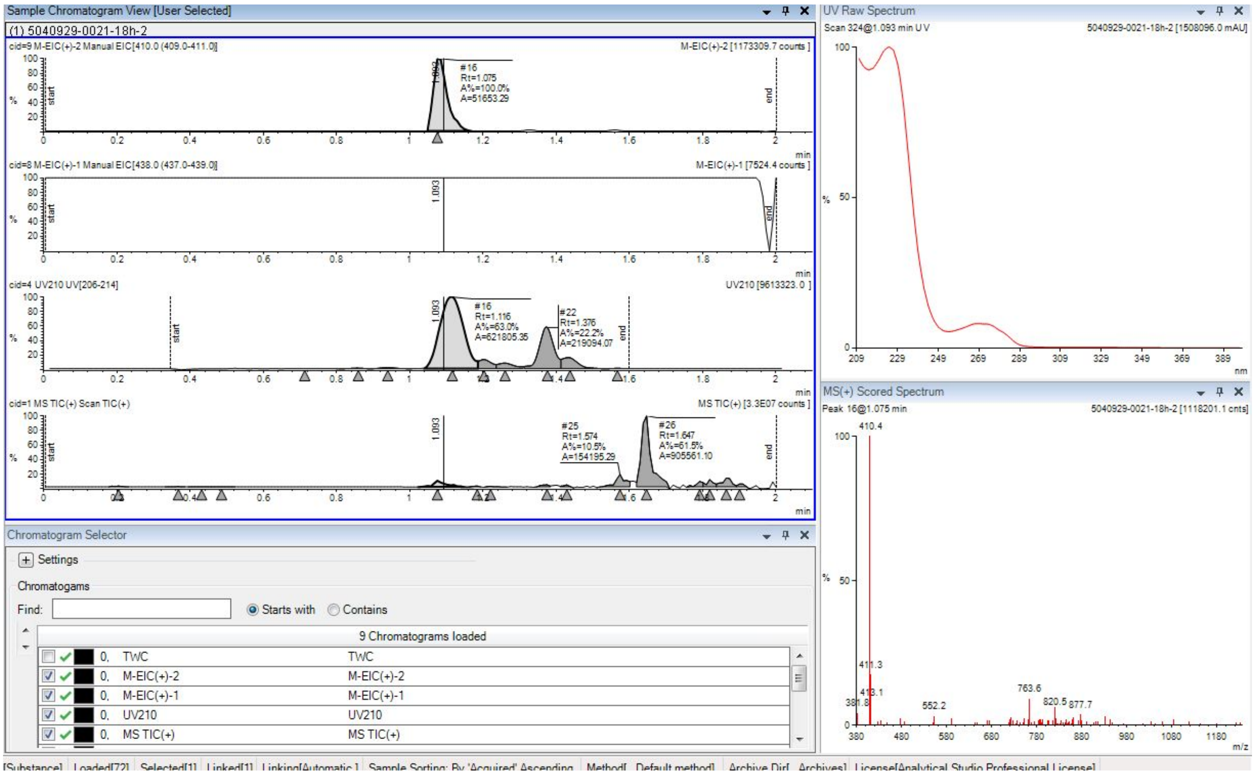

Sample Chromatogram View [User Selected]
(1) 5040929-0021-18h-2
cid=9 M-EIC(+)-2 Manual EIC[410.0 (409.0-411.0)]
M-EIC(+)-2 [1173309.7 counts]
#16
Rt=1.075
A%=100.0%
A=51653.29
cid=8 M-EIC(+)-1 Manual EIC[438.0 (437.0-439.0)]
M-EIC(+)-1 [7524.4 counts]
cid=4 UV210 UV[206-214]
UV210 [9613323.0]
#16
Rt=1.116
A%=63.0%
A=621805.35
#22
Rt=1.376
A%=22.2%
A=219094.07
cid=1 MS TIC(+) Scan TIC(+)
MS TIC(+) [3.3E07 counts]
#25
Rt=1.574
A%=10.5%
A=154195.29
#26
Rt=1.647
A%=61.5%
A=905561.10
UV Raw Spectrum
Scan 324@1.093 min UV
5040929-0021-18h-2 [1508096.0 mAU]
MS(+) Scored Spectrum
Peak 16@1.075 min
5040929-0021-18h-2 [1118201.1 cnts]
410.4
411.3
413.1
381.8
552.2
763.6
820.5
877.7
Chromatogram Selector
Settings
Chromatogams
Find:
Starts with
Contains
9 Chromatograms loaded
0, TWC TWC
0, M-EIC(+)-2 M-EIC(+)-2
0, M-EIC(+)-1 M-EIC(+)-1
0, UV210 UV210
0, MS TIC(+) MS TIC(+)

Reaction with (4-methylpiperazin-1-yl)(4-(4,4,5,5-tetramethyl-1,3,2-dioxaborolan-2-yl)phenyl)methanone (28)

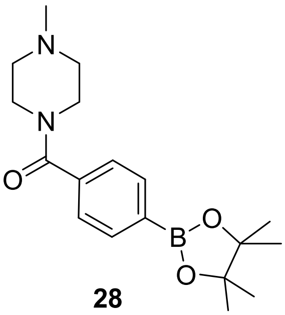

28

m/z: 330.21 (100.0%), 329.22 (24.8%), 331.21 (20.2%), 330.22 (4.9%), 332.22 (2.5%), 331.22 (1.1%)

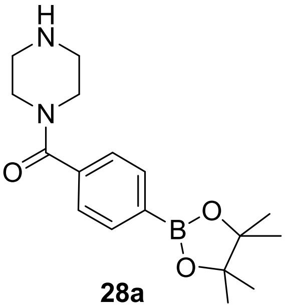

28a

m/z: 316.20 (100.0%), 315.20 (23.7%), 317.20 (18.1%), 318.20 (2.3%)

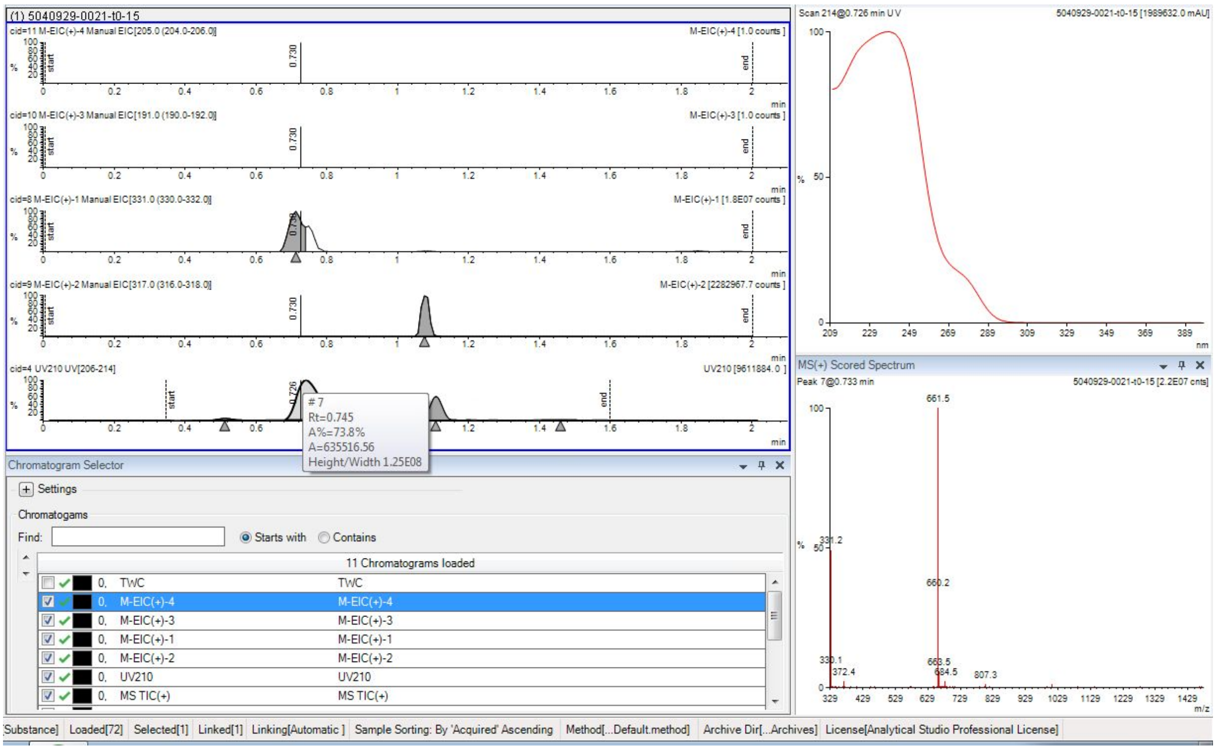

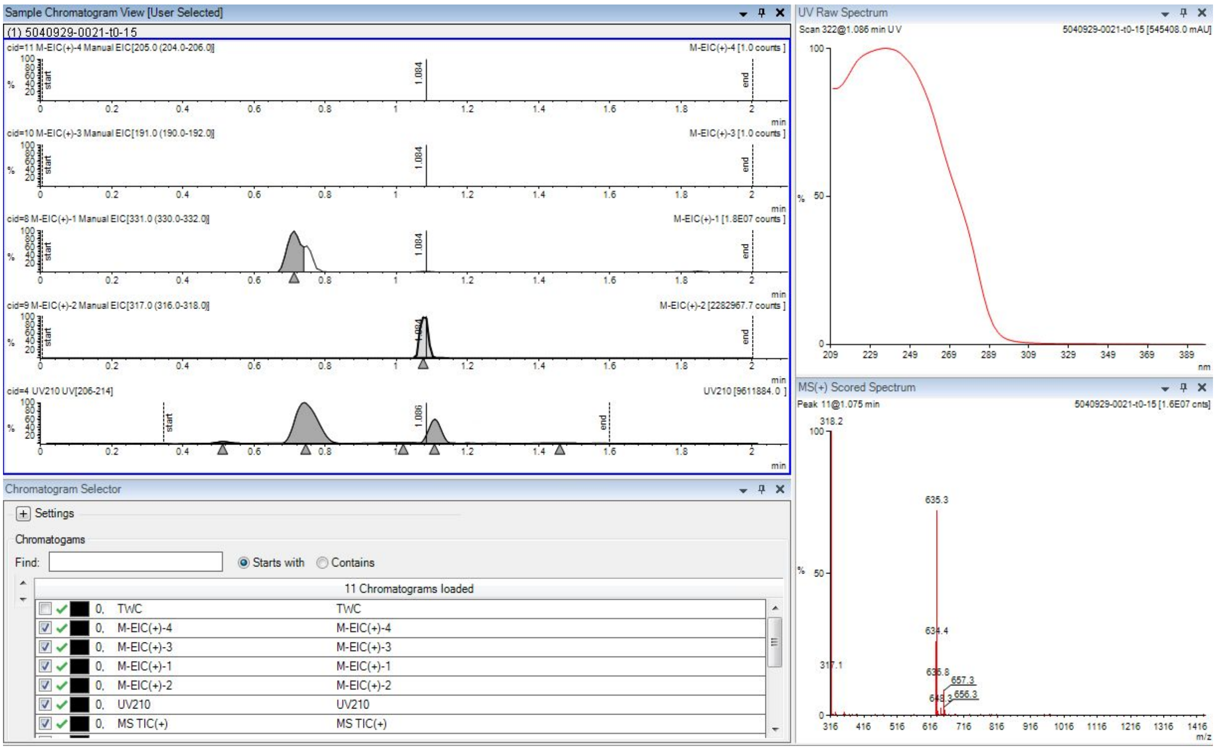
Sample Chromatogram View [User Selected]
(1) 5040929-0021-t0-15
cid=11 M-EIC(+)-4 Manual EIC[205.0 (204.0-206.0)]
M-EIC(+)-4 [1.0 counts]
cid=10 M-EIC(+)-3 Manual EIC[191.0 (190.0-192.0)]
M-EIC(+)-3 [1.0 counts]
cid=8 M-EIC(+)-1 Manual EIC[331.0 (330.0-332.0)]
M-EIC(+)-1 [1.8E07 counts]
cid=9 M-EIC(+)-2 Manual EIC[317.0 (316.0-318.0)]
M-EIC(+)-2 [2282967.7 counts]
cid=4 UV210 UV[206-214]
UV210 [9611884.0]
UV Raw Spectrum
Scan 322@1.086 min UV
5040929-0021-t0-15 [545408.0 mAU]
MS(+) Scored Spectrum
Peak 11@1.075 min
5040929-0021-t0-15 [1.6E07 cnts]
318.2
635.3
634.4
317.1
636.8
657.3
656.3
Chromatogram Selector
Settings
Chromatogams
Find:
Starts with
Contains
11 Chromatograms loaded
TWC
M-EIC(+)-4
M-EIC(+)-3
M-EIC(+)-1
M-EIC(+)-2
UV210
MS TIC(+)

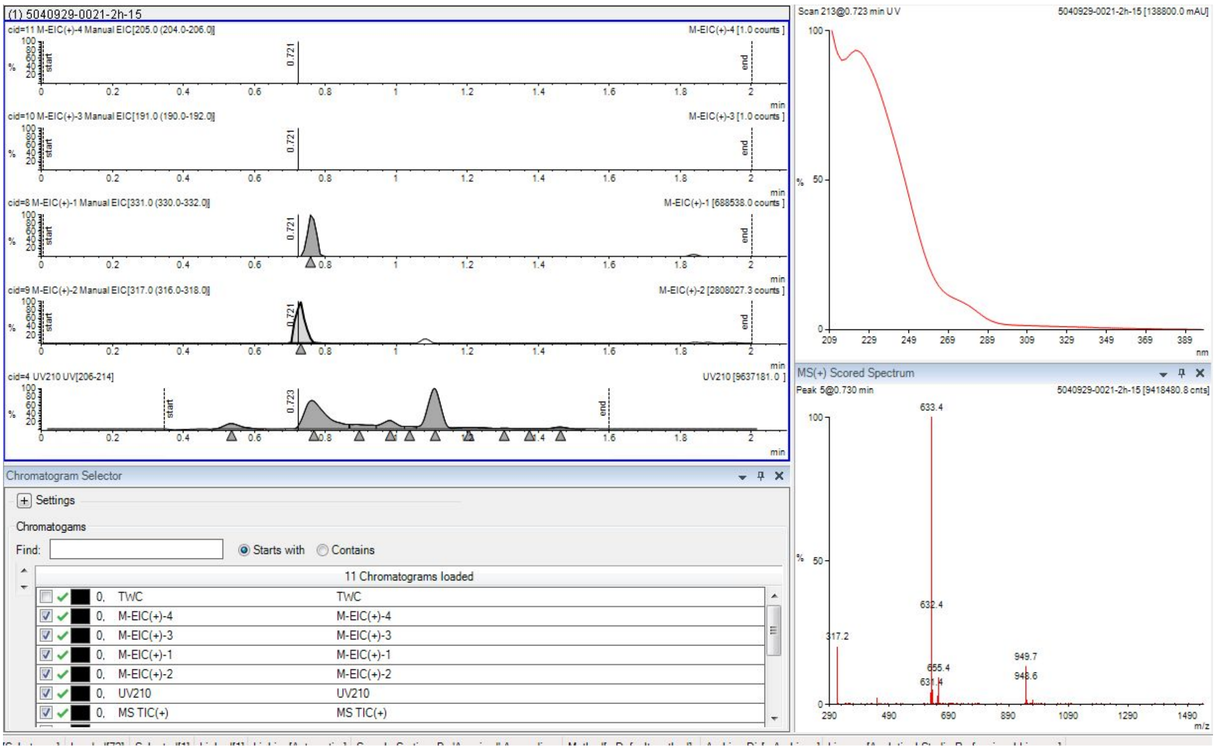
(1) 5040929-0021-2h-15
cid=11 M-EIC(+)-4 Manual EIC[205.0 (204.0-206.0)]
M-EIC(+)-4 [1.0 counts]
cid=10 M-EIC(+)-3 Manual EIC[191.0 (190.0-192.0)]
M-EIC(+)-3 [1.0 counts]
cid=8 M-EIC(+)-1 Manual EIC[331.0 (330.0-332.0)]
M-EIC(+)-1 [688538.0 counts]
cid=9 M-EIC(+)-2 Manual EIC[317.0 (316.0-318.0)]
M-EIC(+)-2 [2808027.3 counts]
cid=4 UV210 UV[206-214]
UV210 [9637181.0]
Scan 213@0.723 min UV
5040929-0021-2h-15 [138800.0 mAU]
MS(+) Scored Spectrum
Peak 5@0.730 min
5040929-0021-2h-15 [9418480.8 cnts]
633.4
632.4
317.2
655.4
949.7
948.6
Chromatogram Selector
Settings
Chromatogams
Find:
Starts with
Contains
11 Chromatograms loaded
TWC
M-EIC(+)-4
M-EIC(+)-3
M-EIC(+)-1
M-EIC(+)-2
UV210
MS TIC(+)

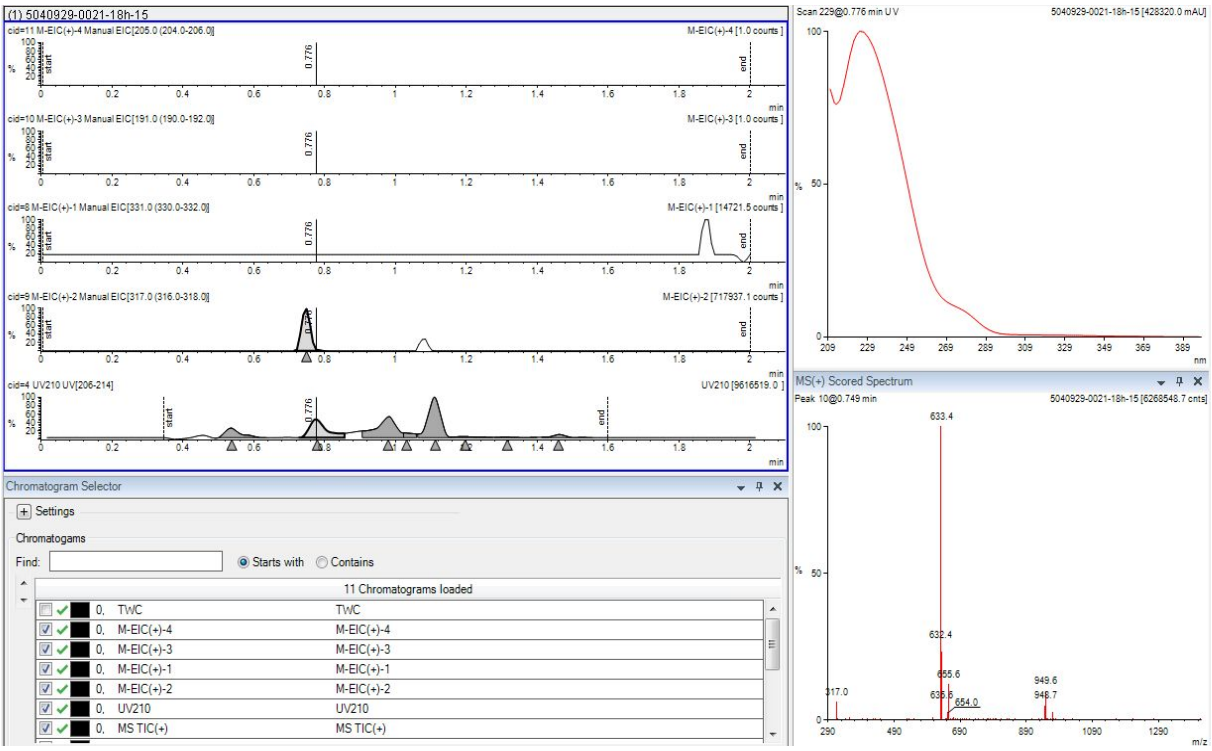

When the same reaction set up with MeOH as solvent instead of MeCN and with 2 eq. CH_3NO_2

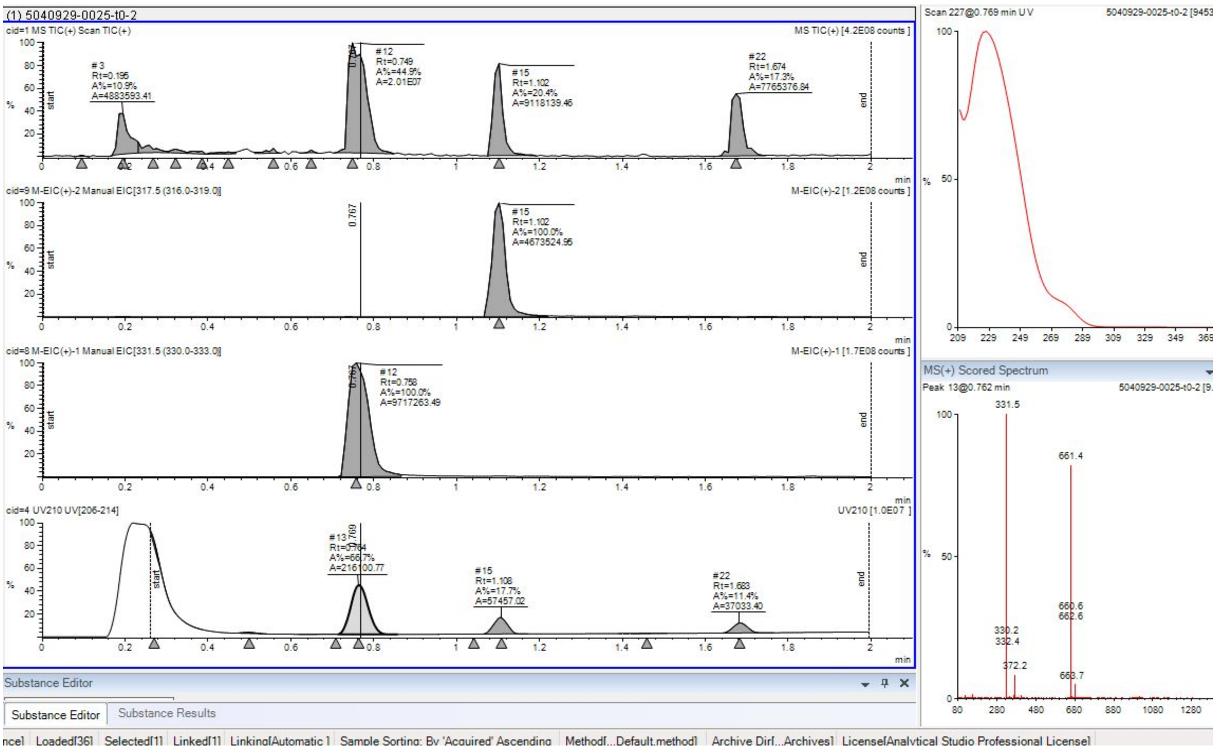

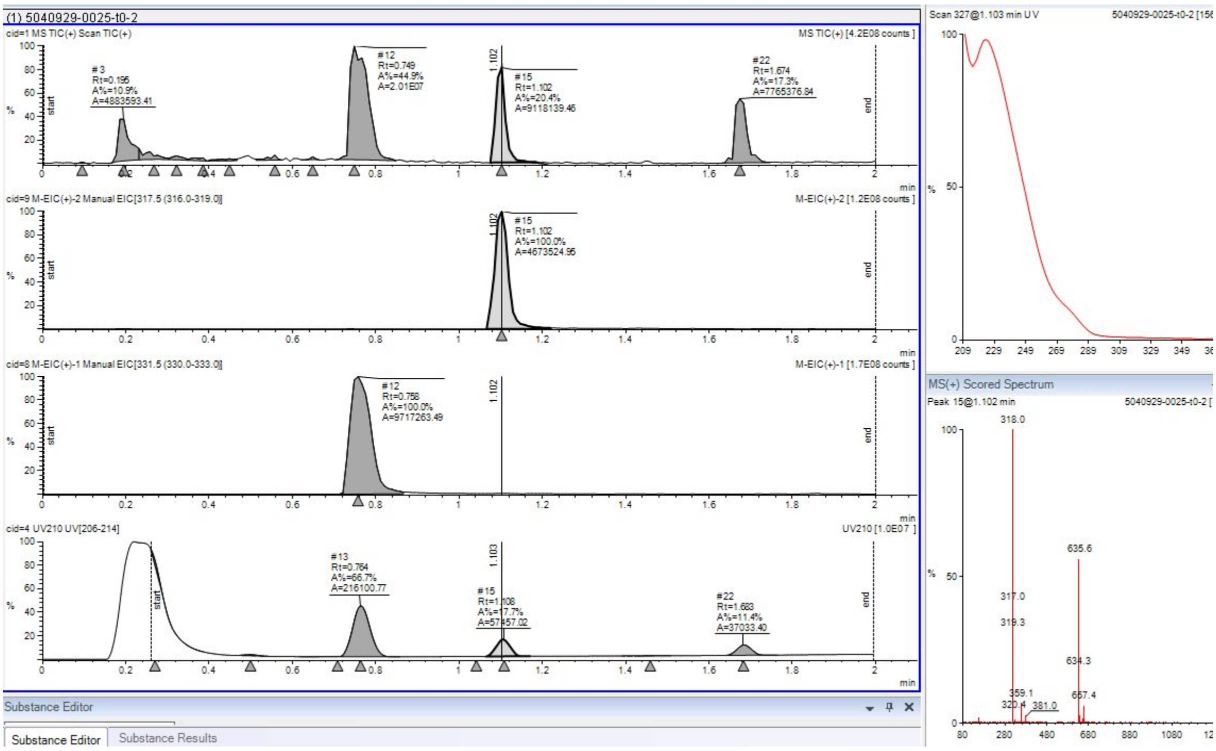

(1) 5040929-0025-t0-2
cid=1 MS TIC(+) Scan TIC(+)
MS TIC(+) [4.2E08 counts]
#3 Rt=0.195 A%=10.9% A=4883593.41
#12 Rt=0.749 A%=44.9% A=2.01E07
#15 Rt=1.102 A%=20.4% A=9118139.46
#22 Rt=1.674 A%=17.3% A=7765376.84
cid=9 M-EIC(+)-2 Manual EIC[317.5 (316.0-319.0)]
M-EIC(+)-2 [1.2E08 counts]
#15 Rt=1.102 A%=100.0% A=4673524.95
cid=8 M-EIC(+)-1 Manual EIC[331.5 (330.0-333.0)]
M-EIC(+)-1 [1.7E08 counts]
#12 Rt=0.758 A%=100.0% A=9717263.49
cid=4 UV210 UV[206-214]
UV210 [1.0E07]
#13 Rt=0.764 A%=66.7% A=216100.77
#22 Rt=1.683 A%=11.4% A=37033.40
Substance Editor
Substance Results
Scan 327@1.103 min UV
MS(+) Scored Spectrum
Peak 15@1.102 min
318.0
635.6
317.0
319.3
634.3
359.1
320.4
381.0
657.4

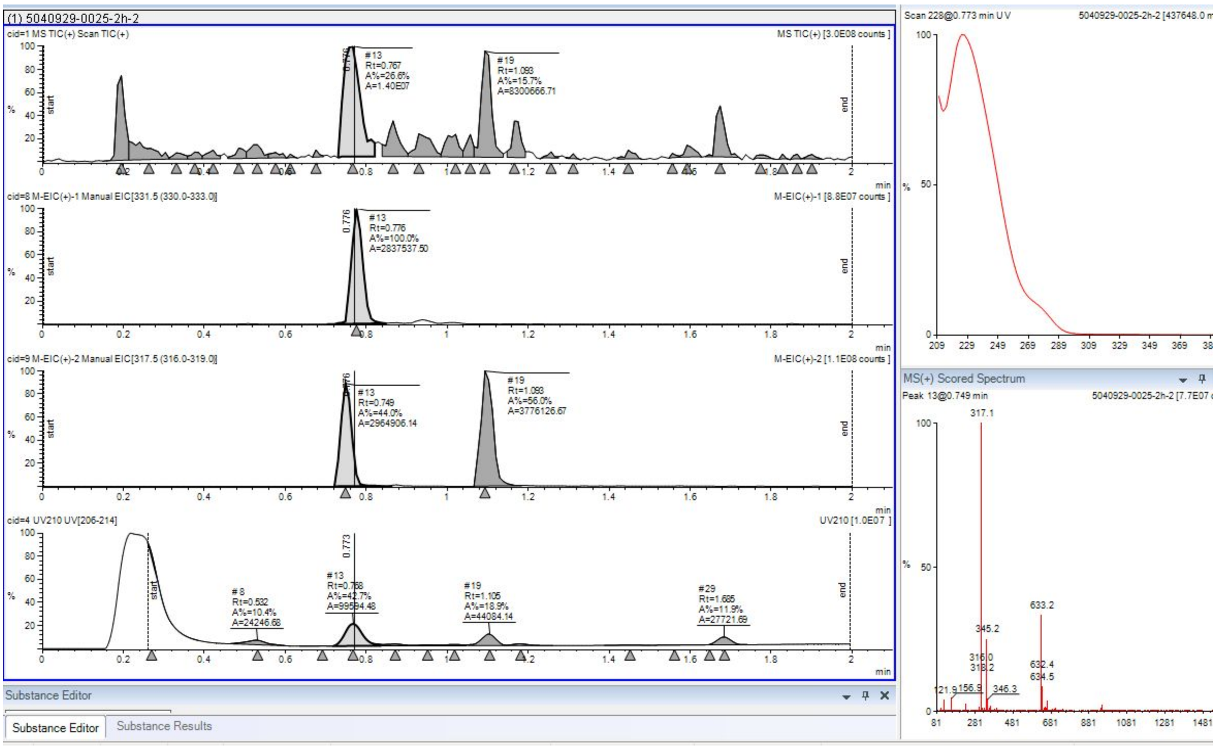

(1) 5040929-0025-2h-2
cid=1 MS TIC(+) Scan TIC(+)
MS TIC(+) [3.0E08 counts]
#13 Rt=0.767 A%=26.6% A=1.40E07
#19 Rt=1.093 A%=15.7% A=8300666.71
cid=8 M-EIC(+)-1 Manual EIC[331.5 (330.0-333.0)]
M-EIC(+)-1 [8.8E07 counts]
#13 Rt=0.776 A%=100.0% A=2837537.50
cid=9 M-EIC(+)-2 Manual EIC[317.5 (316.0-319.0)]
M-EIC(+)-2 [1.1E08 counts]
#13 Rt=0.749 A%=44.0% A=2964906.14
#19 Rt=1.093 A%=56.0% A=3776126.67
cid=4 UV210 UV[206-214]
UV210 [1.0E07]
#8 Rt=0.532 A%=10.4% A=24246.68
#19 Rt=1.105 A%=18.9% A=44084.14
#29 Rt=1.685 A%=11.9% A=27721.69
Substance Editor
Substance Results
Scan 228@0.773 min UV
MS(+) Scored Spectrum
Peak 13@0.749 min
317.1
633.2
345.2
632.4
634.5
346.3

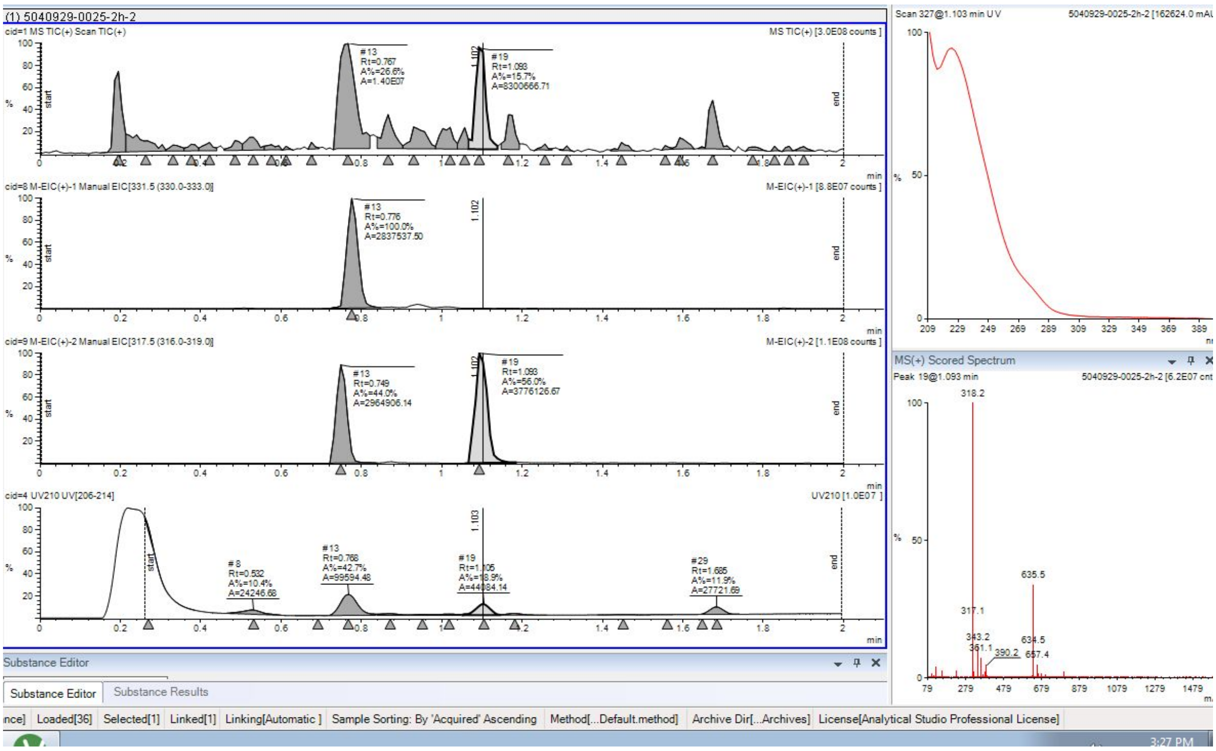
(1) 5040929-0025-2h-2
MS(+) Scored Spectrum
Substance Editor
Substance Results

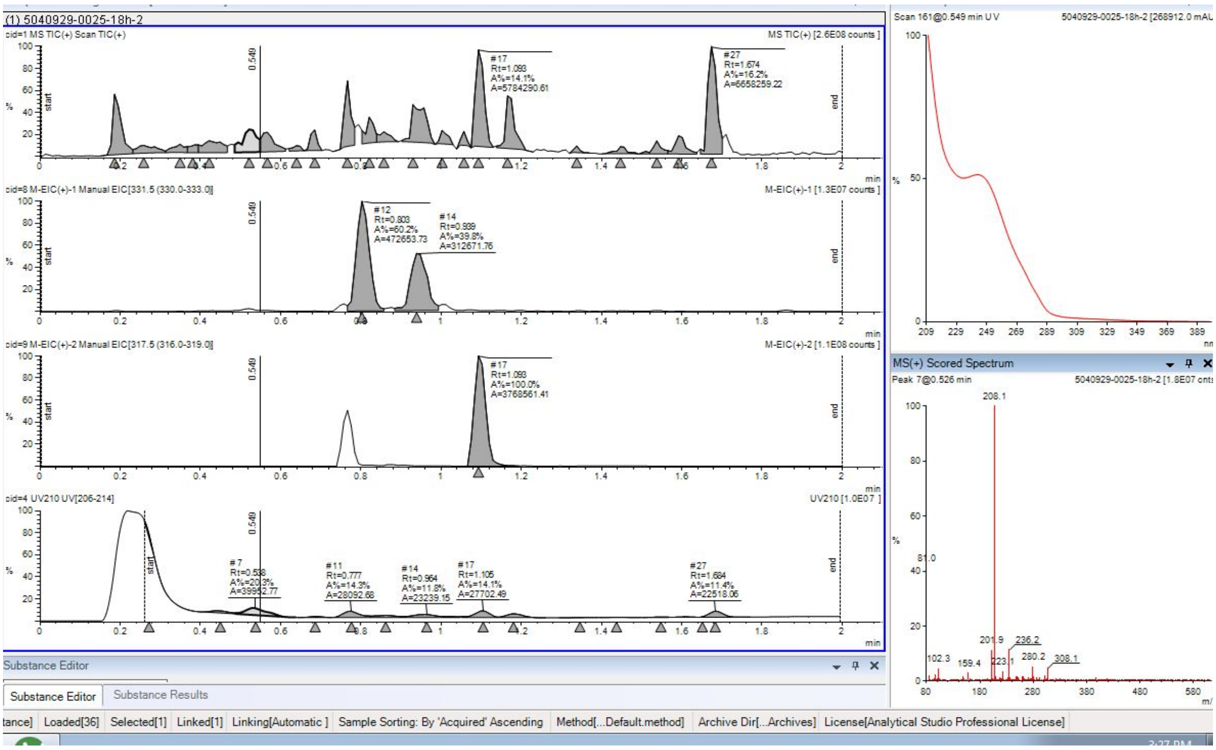
(1) 5040929-0025-18h-2
MS(+) Scored Spectrum
Substance Editor
Substance Results

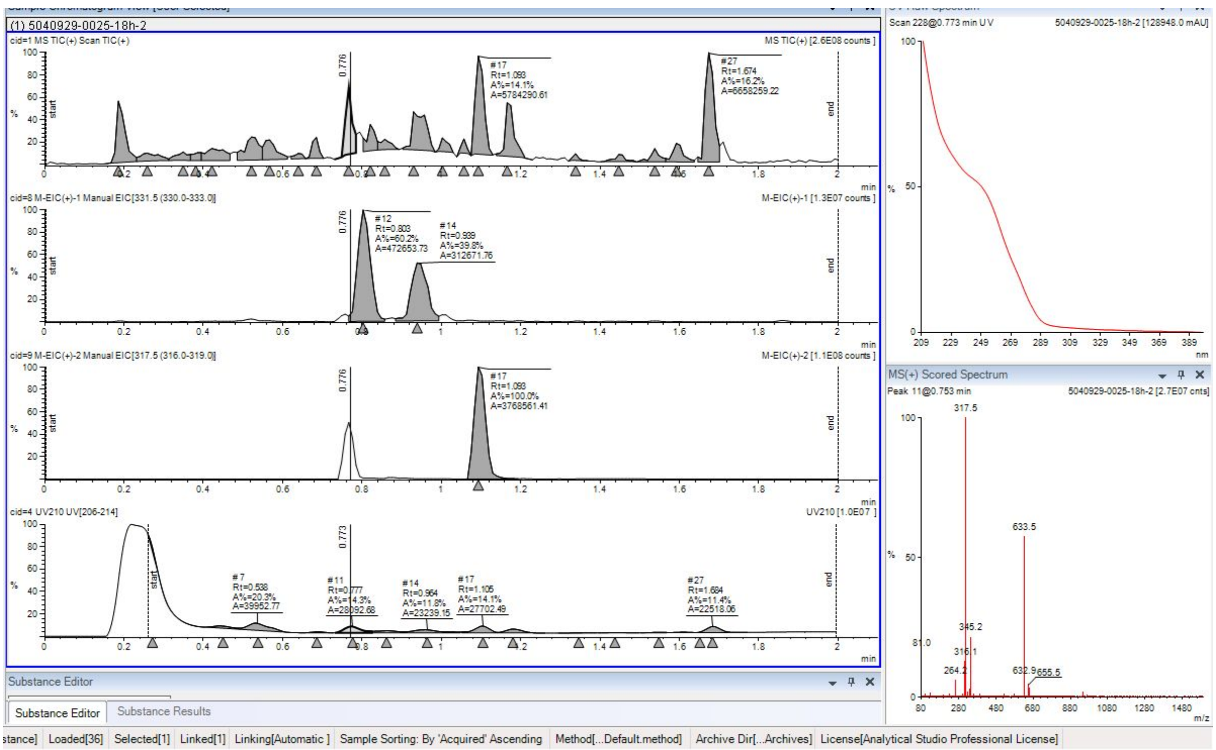

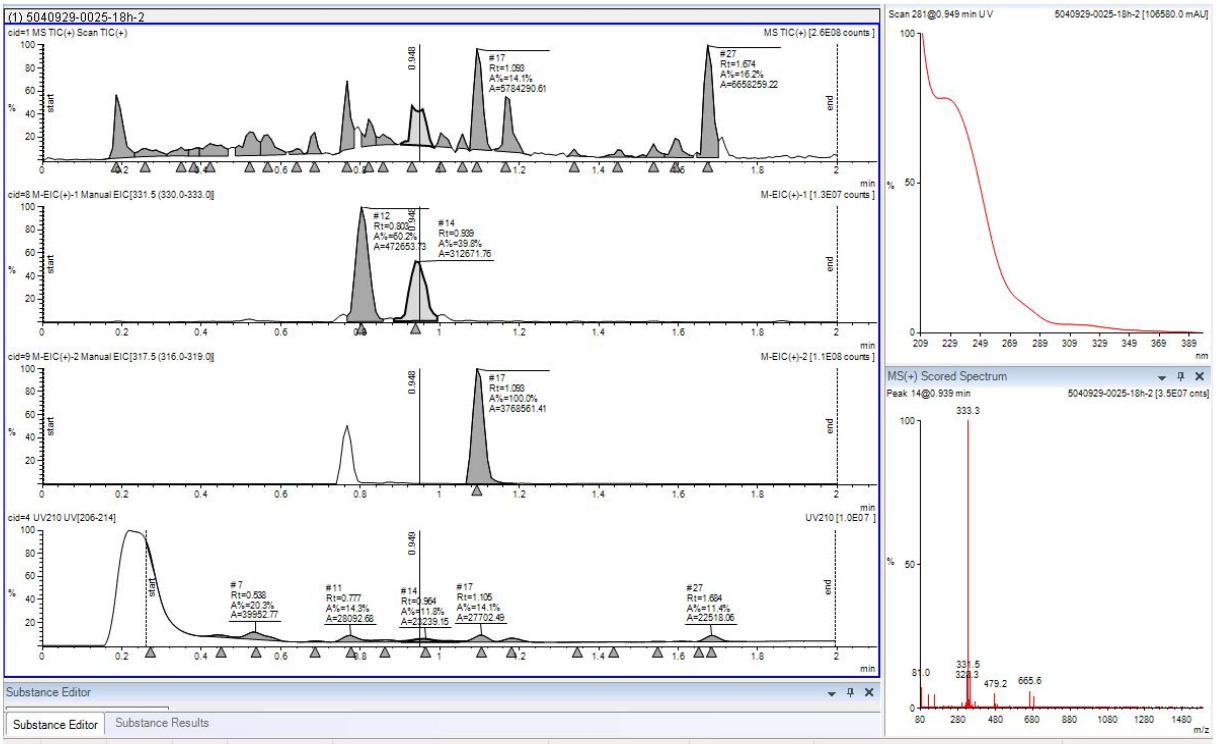

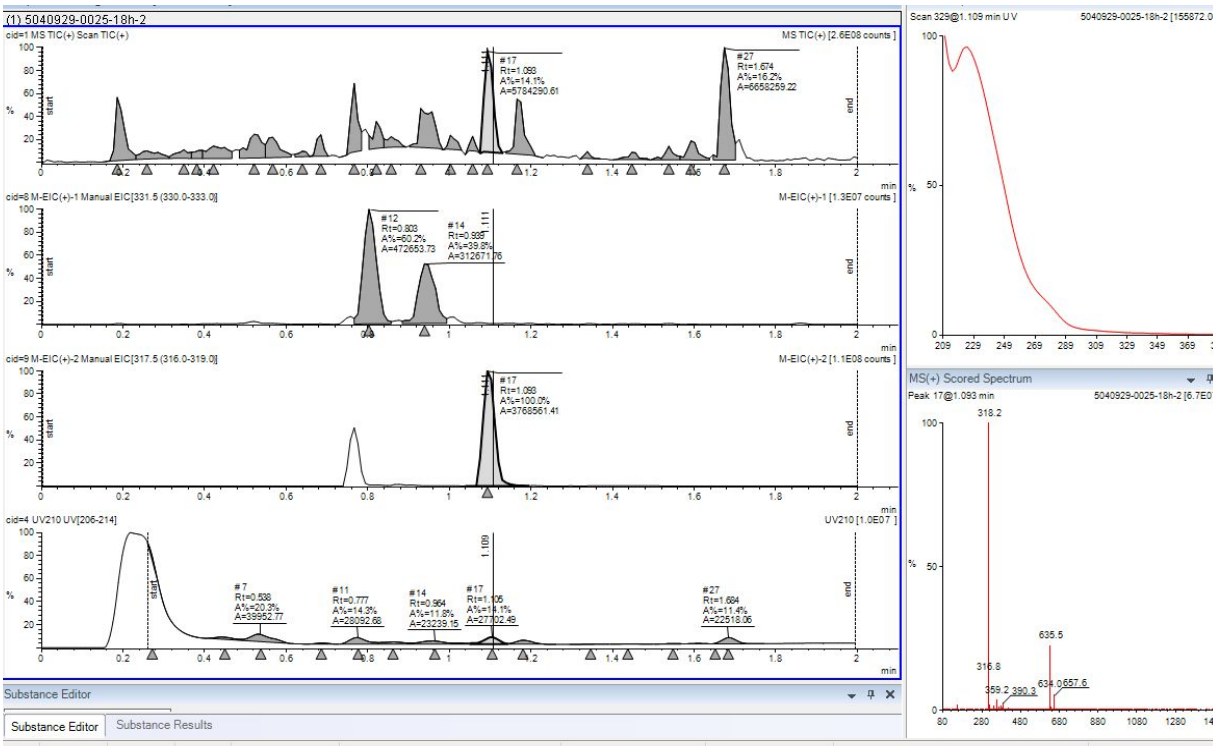

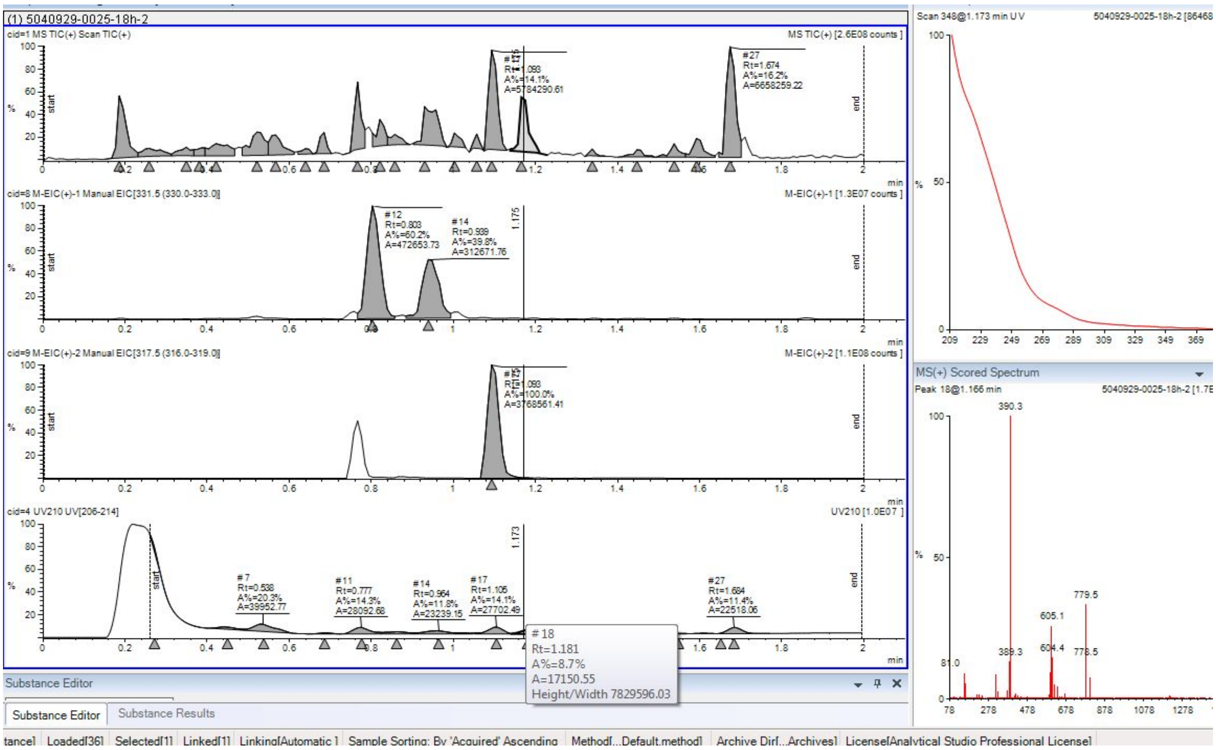

Reaction with (R)-N-benzyl-1-phenylethanamine (29)

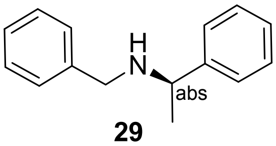

29

m/z: 211.14 (100.0%), 212.14 (16.4%), 213.14 (1.3%)

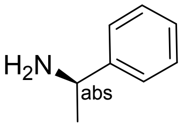

29a

m/z: 121.09 (100.0%), 122.09 (9.0%)

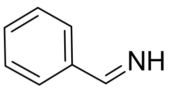

29b

m/z: 105.06 (100.0%), 106.06 (7.7%)

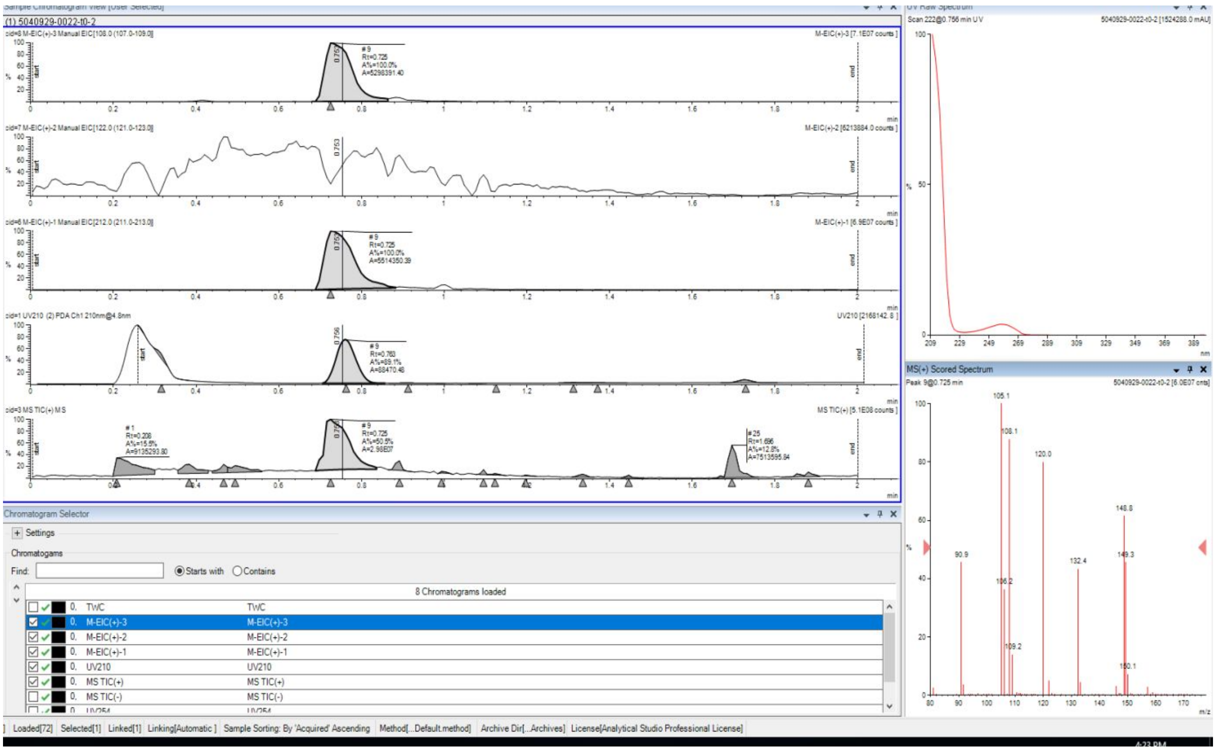

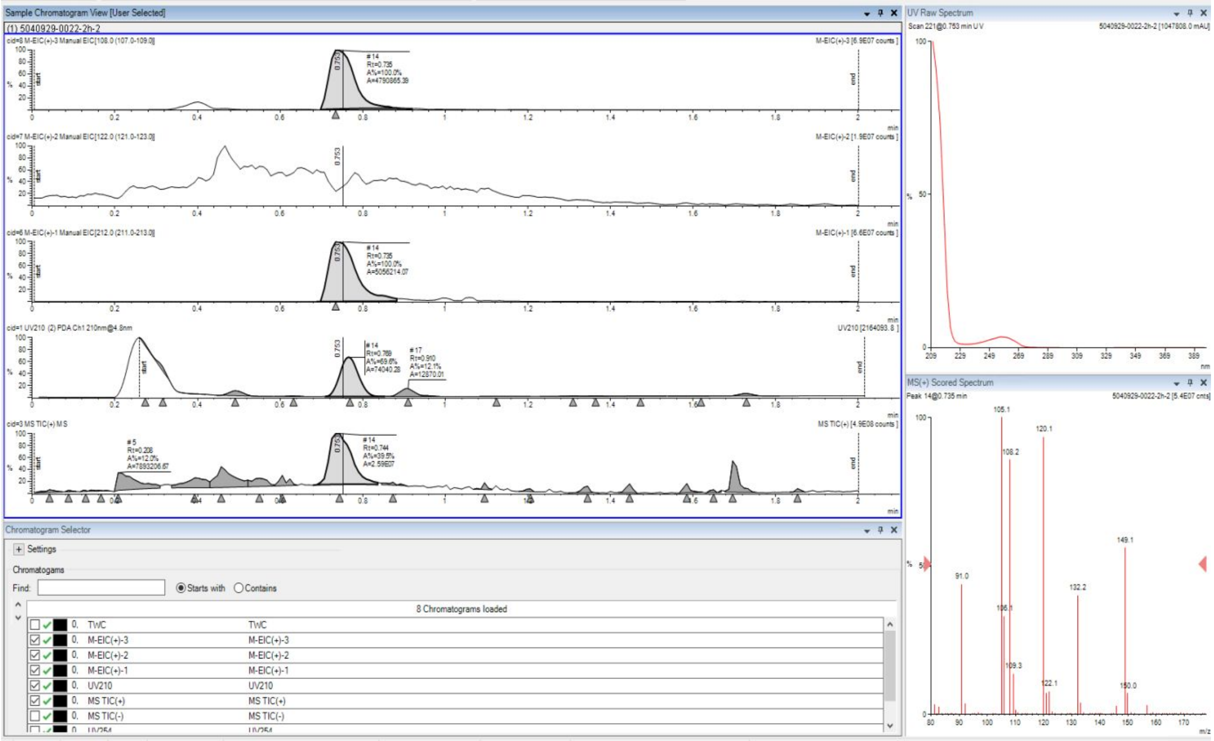
Sample Chromatogram View [User Selected]
(1) 5040929-0022-2h-2
UV Raw Spectrum
MS(+) Scored Spectrum
Chromatogram Selector
Settings
Chromatograms
Find:
Starts with
Contains
8 Chromatograms loaded
TWC
M-EIC(+)-3
M-EIC(+)-2
M-EIC(+)-1
UV210
MS TIC(+)
MS TIC(-)

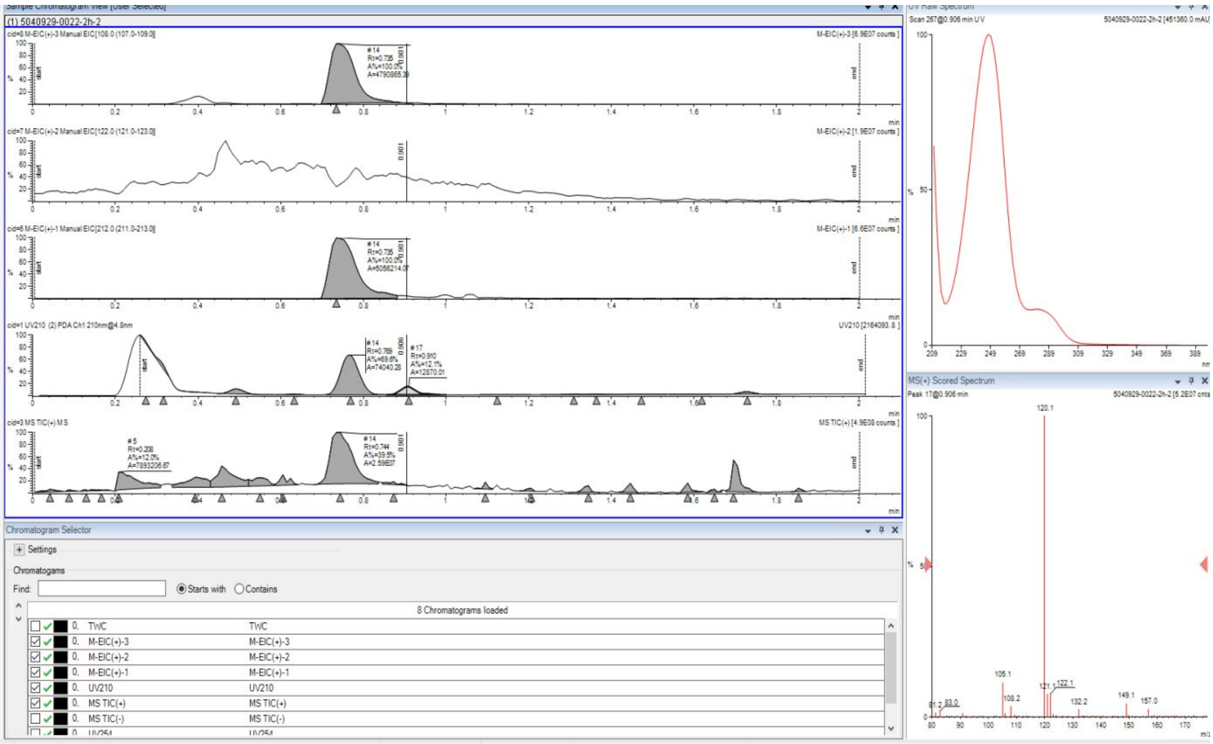
(1) 5040929-0022-2h-2
MS(+) Scored Spectrum
Chromatogram Selector
Settings
Chromatograms
Find:
Starts with
Contains
8 Chromatograms loaded
TWC
M-EIC(+)-3
M-EIC(+)-2
M-EIC(+)-1
UV210
MS TIC(+)
MS TIC(-)

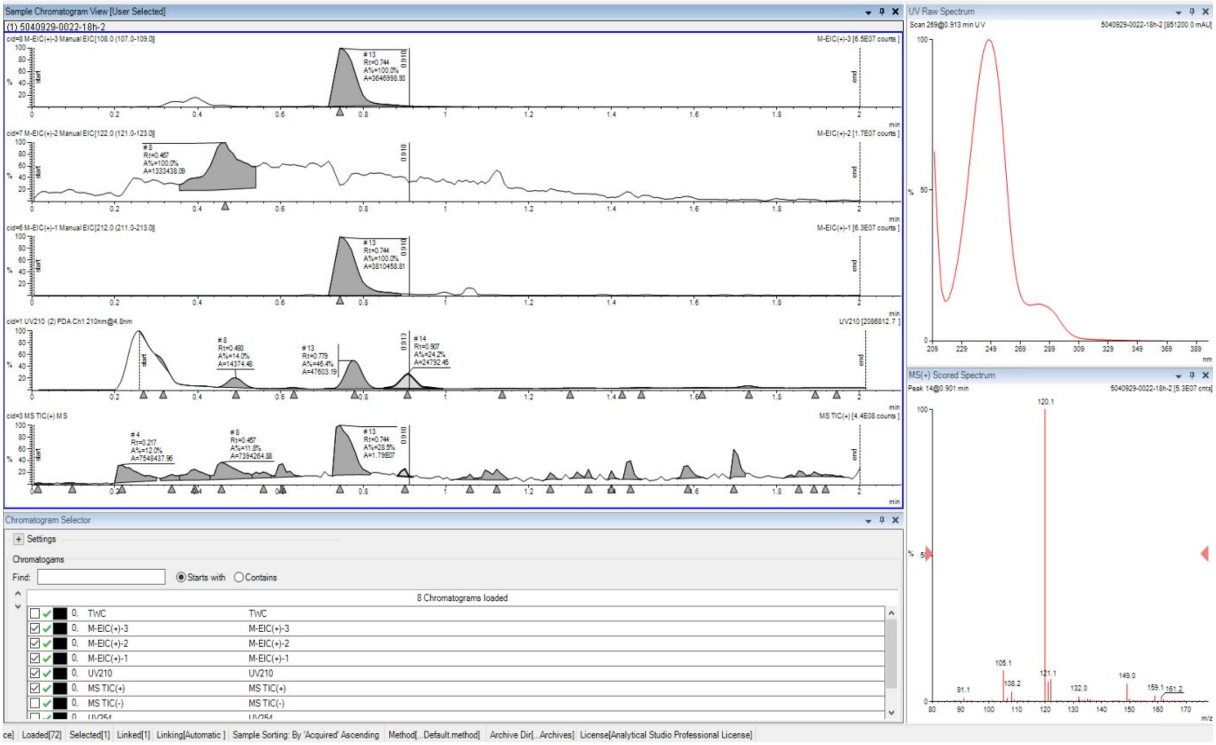
Sample Chromatogram View [User Selected]
(1) 5040929-0022-18h-2
UV Raw Spectrum
MS(+) Scored Spectrum
Chromatogram Selector
8 Chromatograms loaded

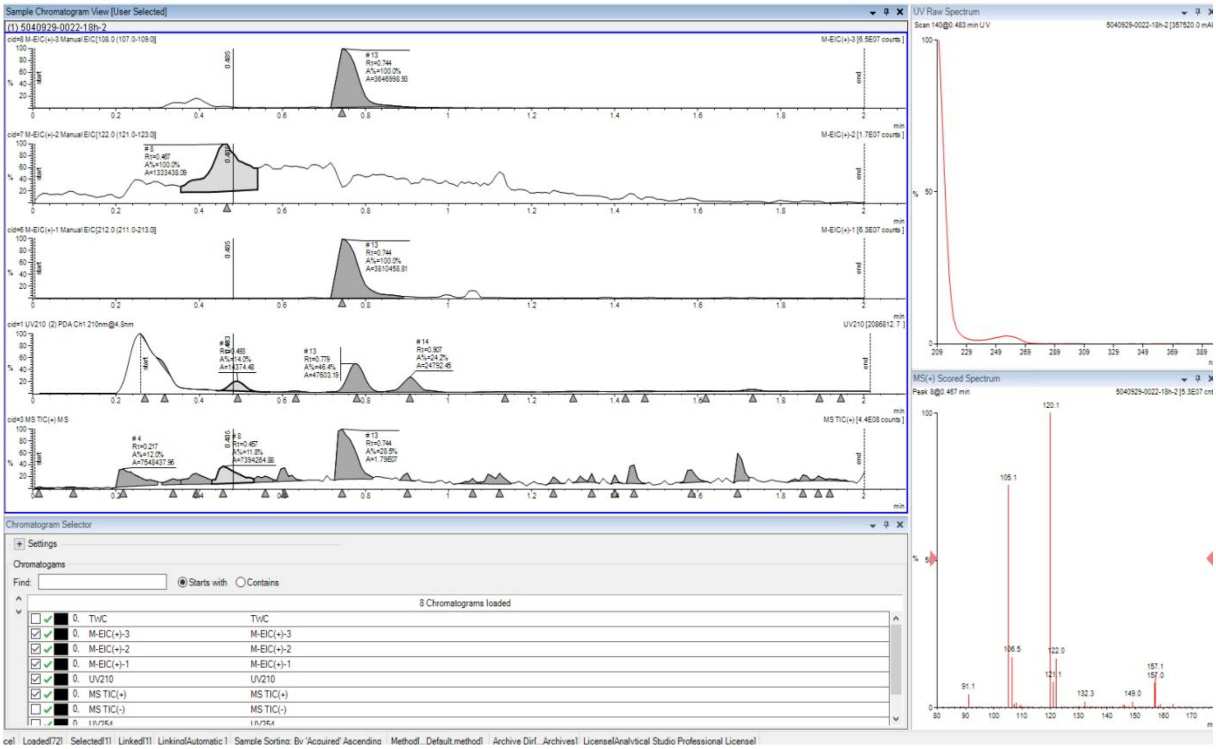
Sample Chromatogram View [User Selected]
(1) 5040929-0022-18h-2
UV Raw Spectrum
MS(+) Scored Spectrum
Chromatogram Selector
8 Chromatograms loaded

Reaction with (R)-2-(benzylamino)-2-phenylethanol (30)

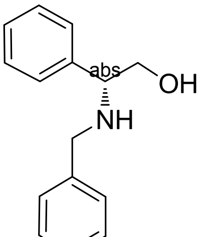

30

m/z: 227.13 (100.0%), 228.13 (16.6%), 229.14 (1.5%)

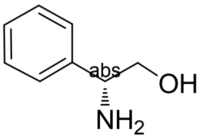

30a

m/z: 137.08 (100.0%), 138.09 (8.8%)

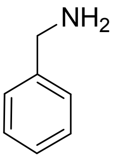

30b

m/z: 107.07 (100.0%), 108.08 (7.7%)

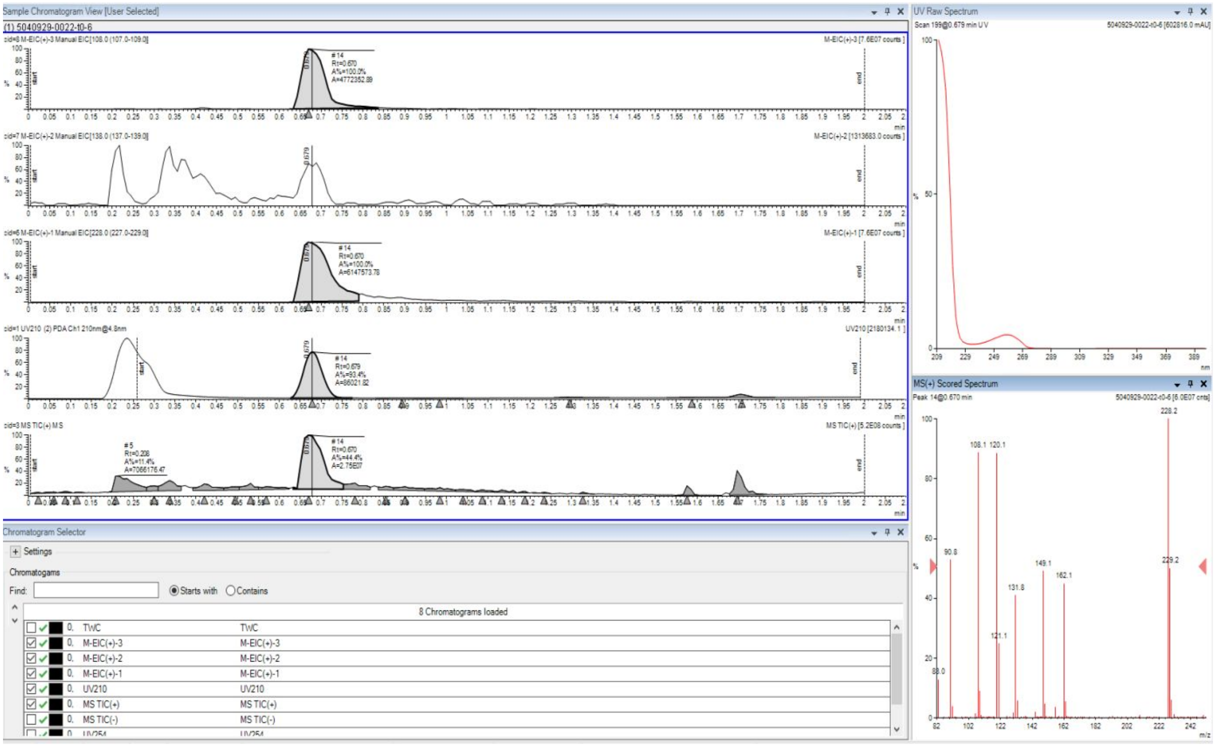

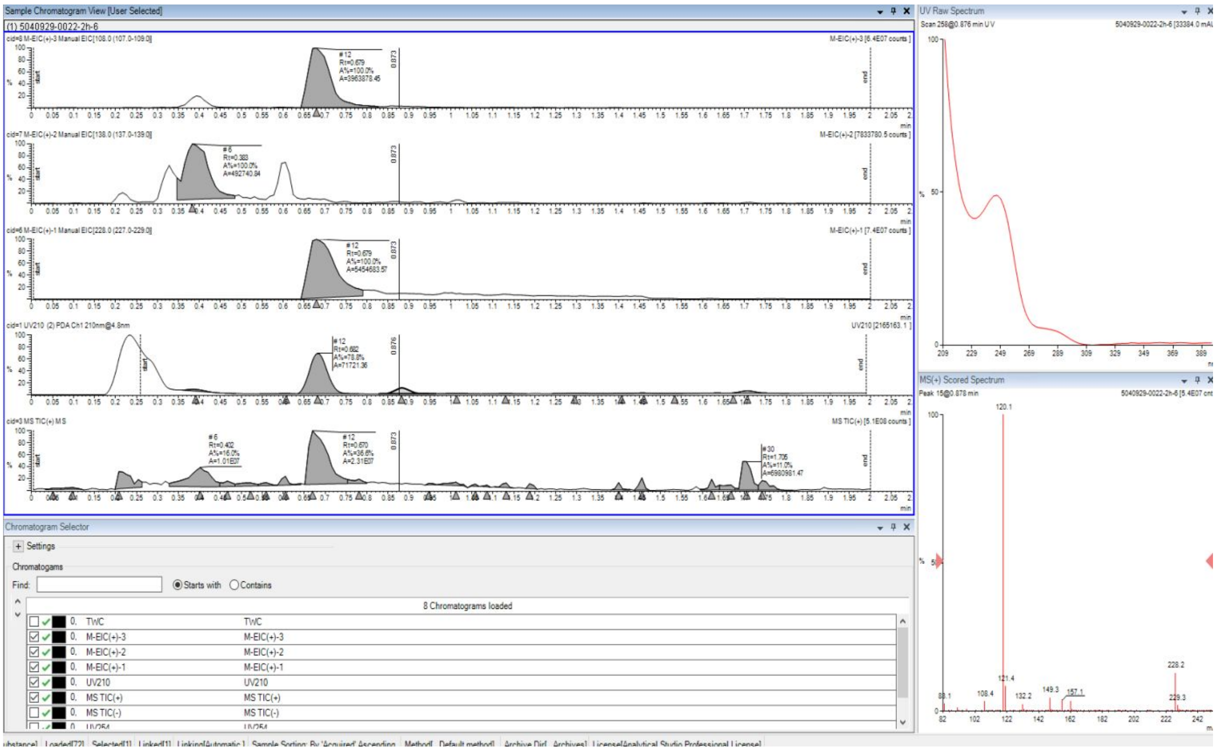

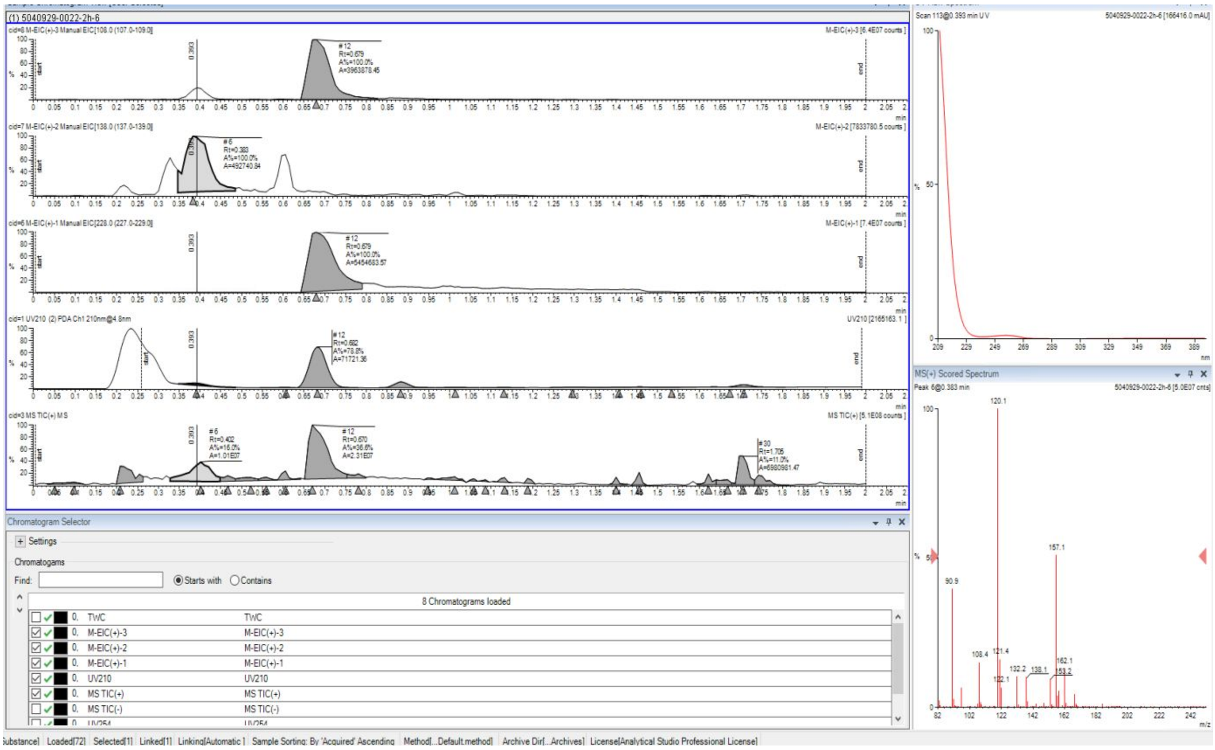

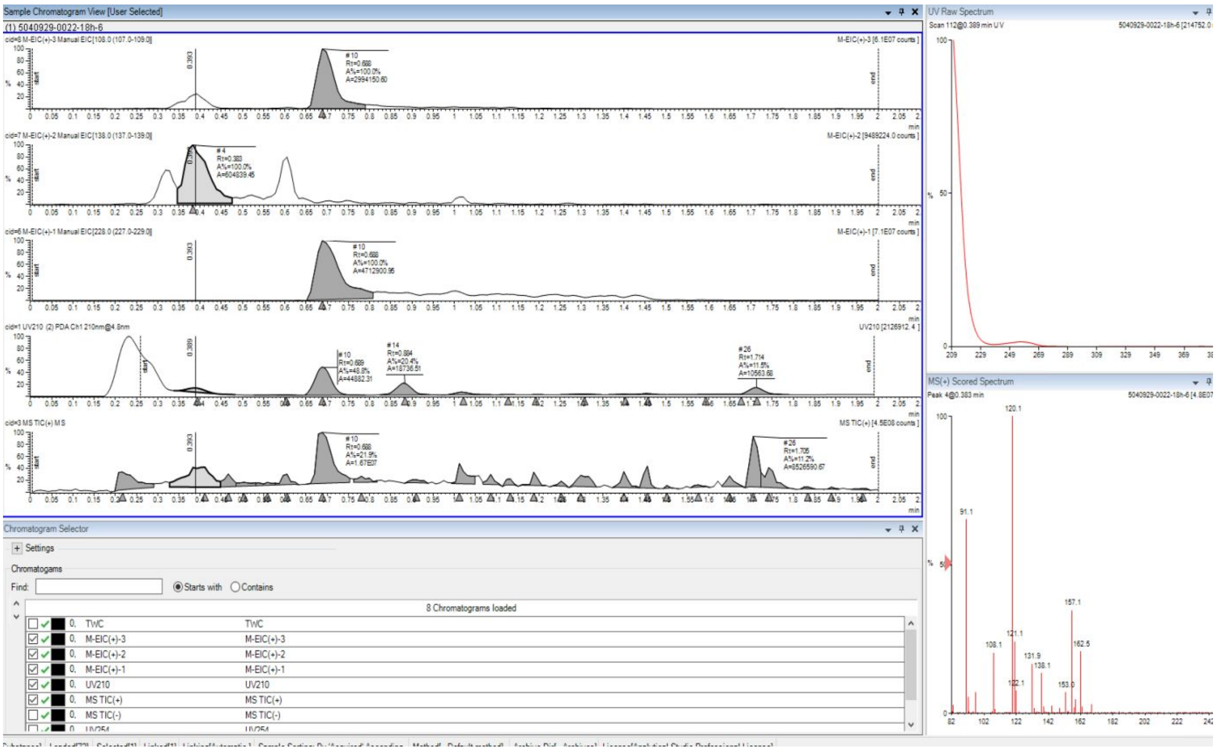
Sample Chromatogram View [User Selected]
(1) 5040929-0022-18h-6
UV Raw Spectrum
MS(+) Scored Spectrum
Chromatogram Selector
Settings
Chromatograms
Find:
Starts with
Contains
8 Chromatograms loaded
TWC
M-EIC(+)-3
M-EIC(+)-2
M-EIC(+)-1
UV210
MS TIC(+)
MS TIC(-)

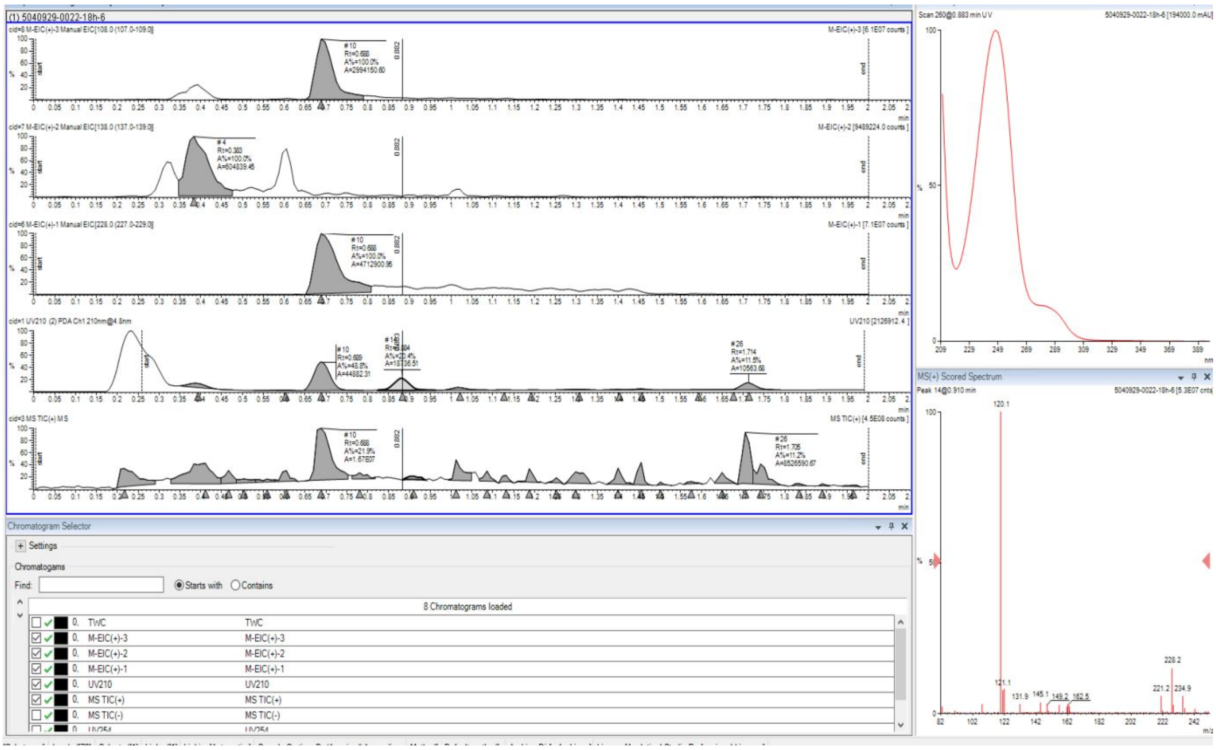
(1) 5040929-0022-18h-6
MS(+) Scored Spectrum
Chromatogram Selector
Settings
Chromatograms
Find:
Starts with
Contains
8 Chromatograms loaded
TWC
M-EIC(+)-3
M-EIC(+)-2
M-EIC(+)-1
UV210
MS TIC(+)
MS TIC(-)

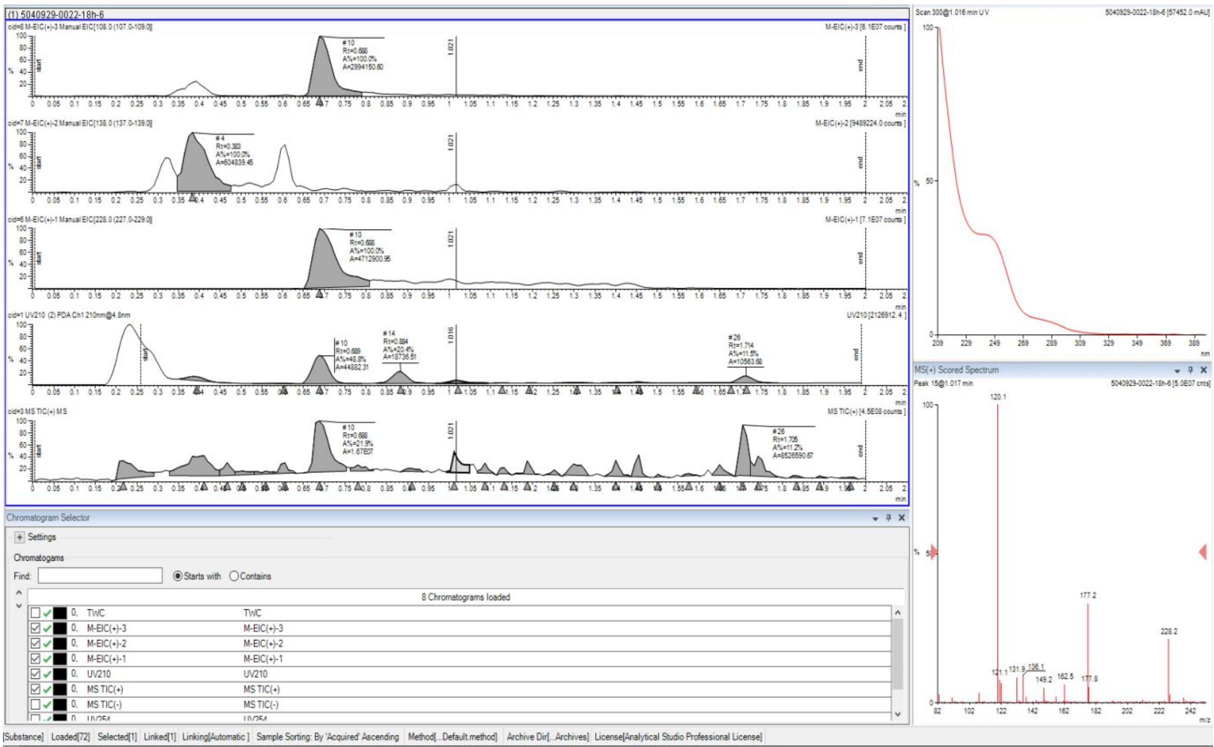

Reaction with 8-(4-fluorophenyl)-2-(trifluoromethyl)-5,6,7,8-tetrahydroimidazo[1,2-a]pyrazine (31)

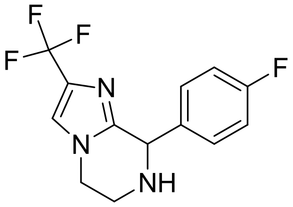

31

m/z: 285.09 (100.0%), 286.09 (15.2%)

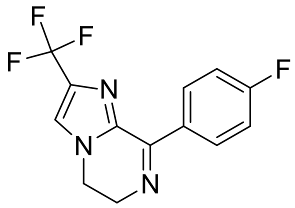

31a

m/z: 283.07 (100.0%), 284.08 (14.2%), 284.07 (1.1%)

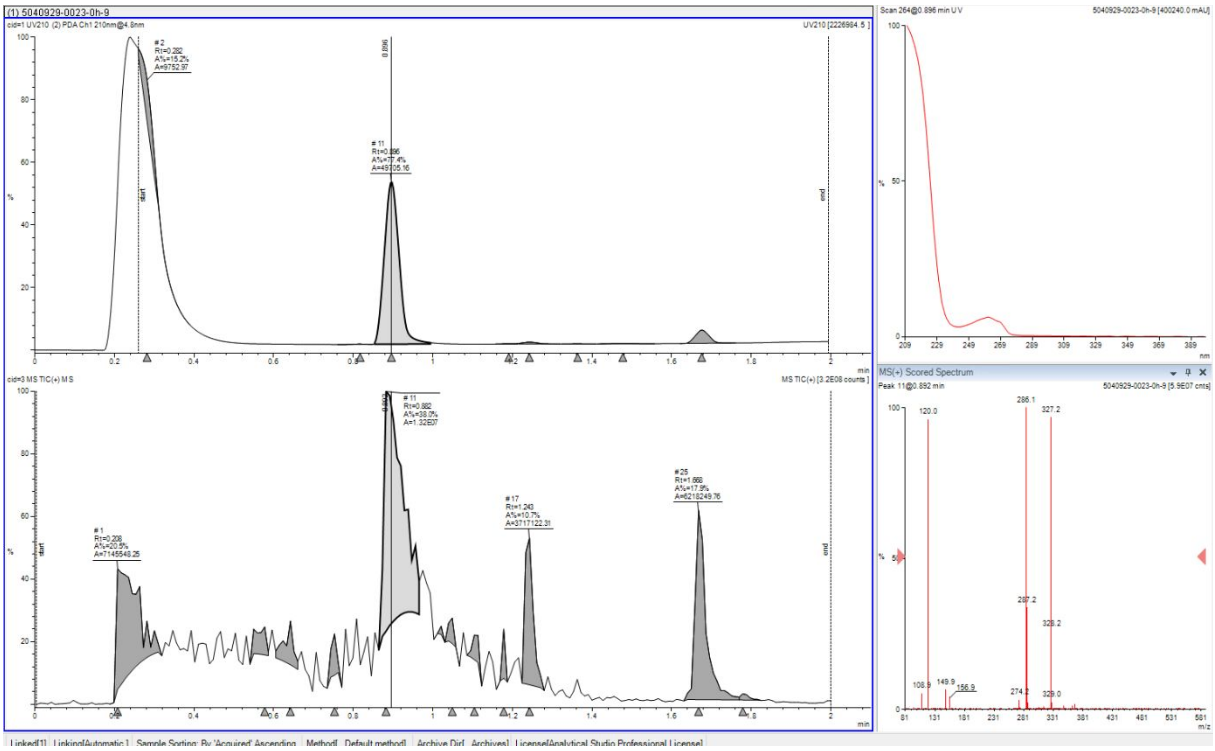

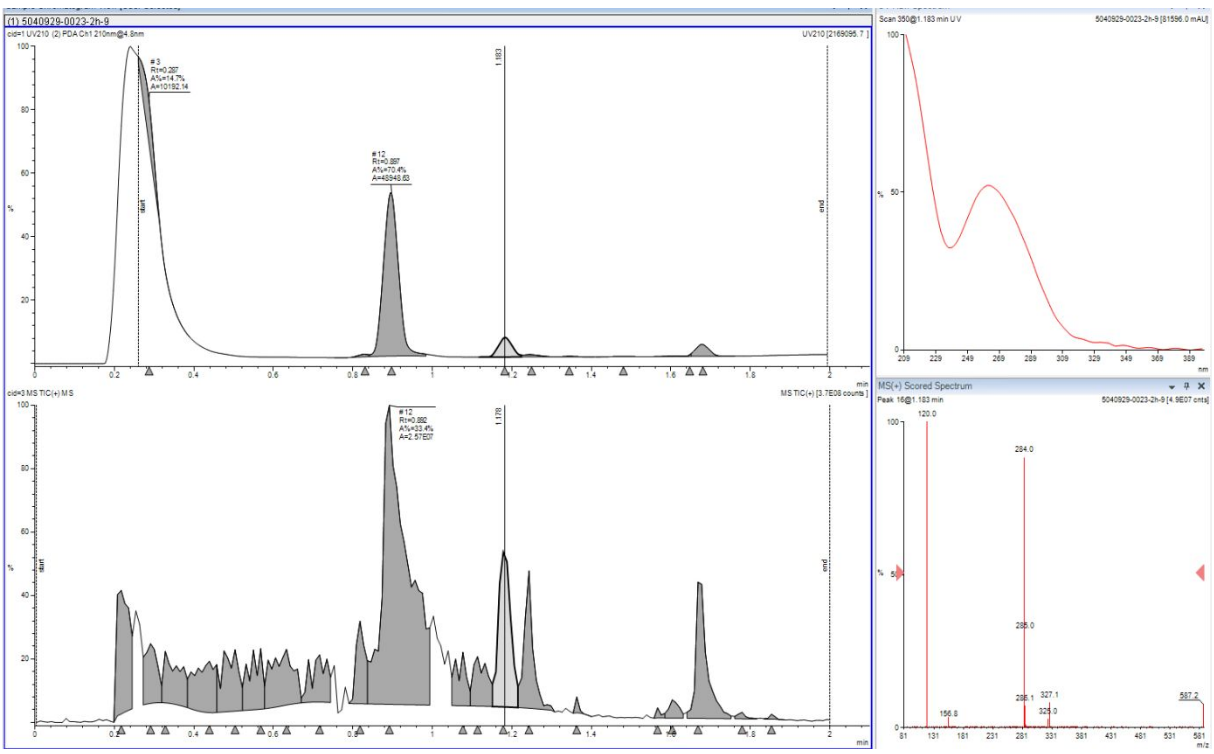
(1) 5040929-0023-2h-9
cid=1 UV210 (2) PDA Ch1 210nm@4.8nm
cid=3 MS TIC(+) MS
MS(+) Scored Spectrum
120.0
284.0
587.2

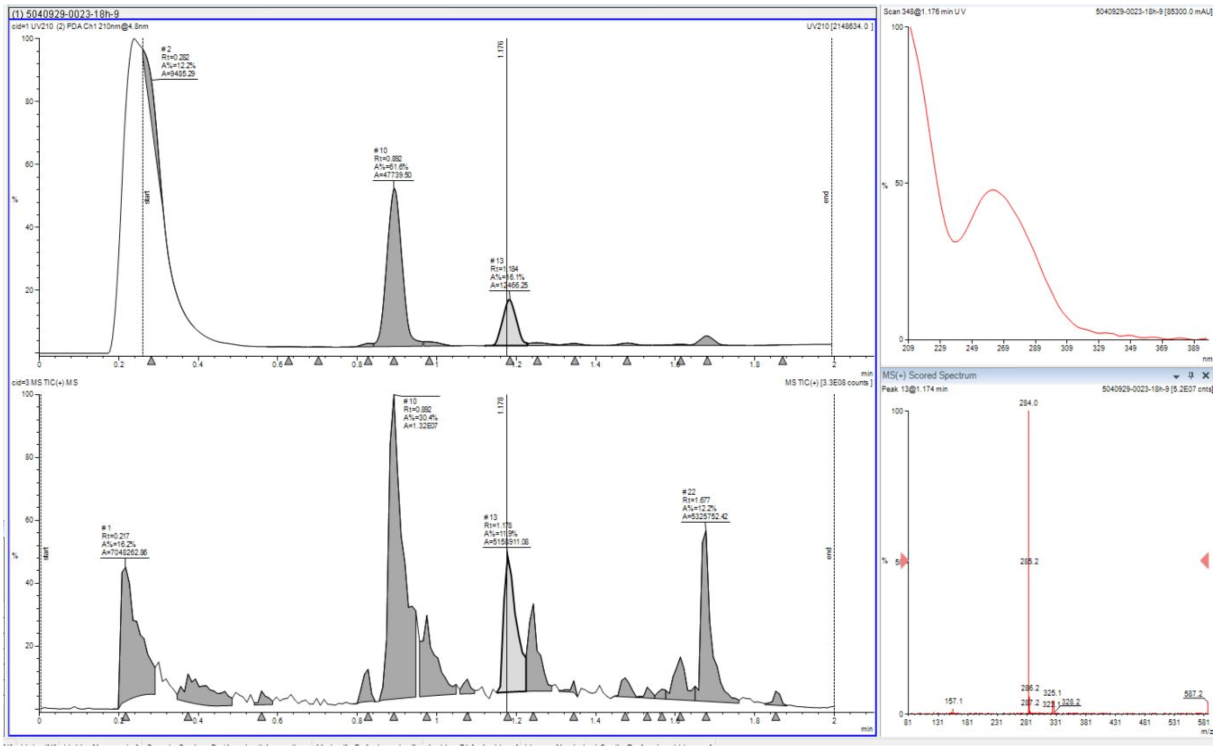
(1) 5040929-0023-18h-9
cid=1 UV210 (2) PDA Ch1 210nm@4.8nm
cid=3 MS TIC(+) MS
MS(+) Scored Spectrum
284.0
587.2

When reaction set up without CH_3NO_2

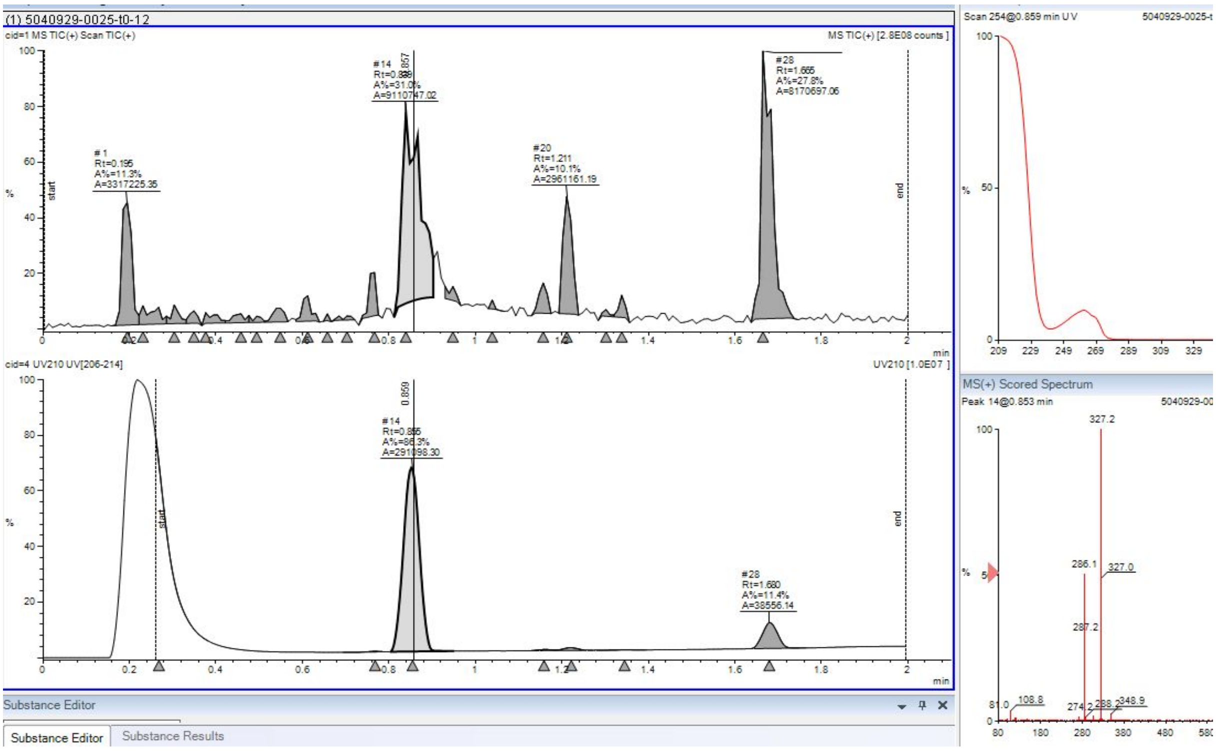

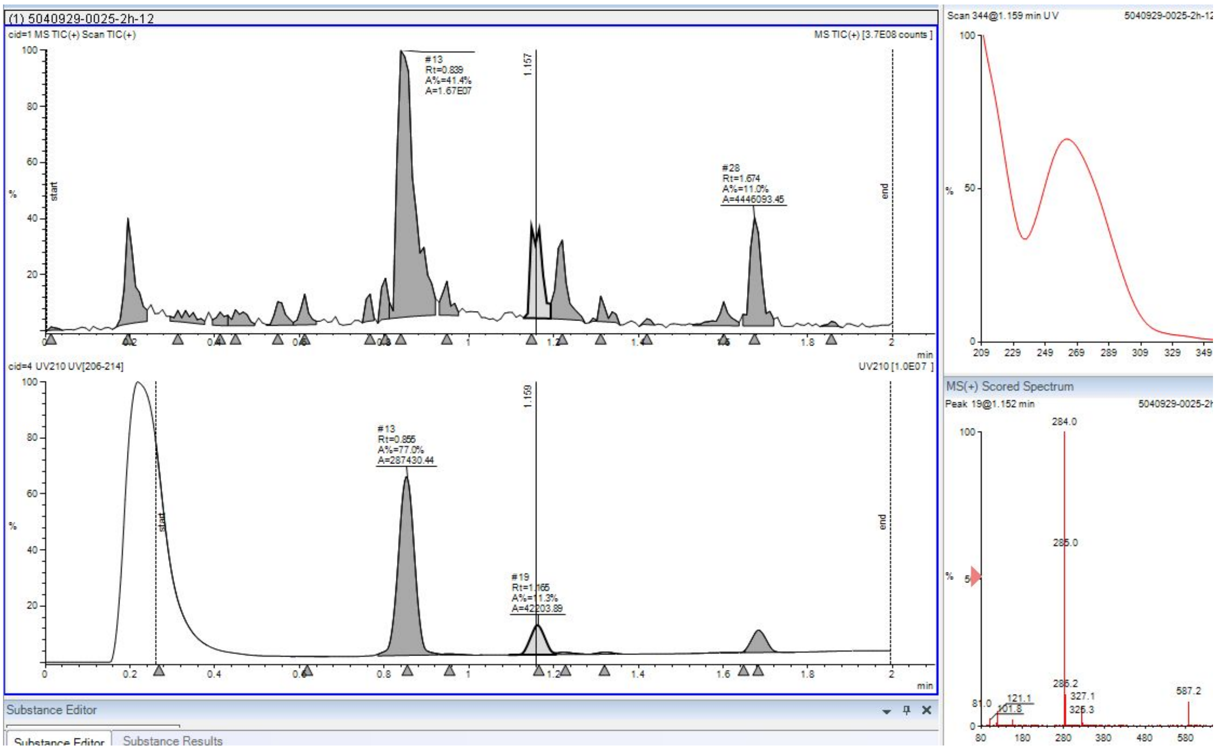

Reaction with 1-(thiophen-2-yl)-1,2,3,4-tetrahydropyrrolo[1,2-a]pyrazine (32)

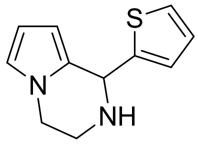

32

m/z: 204.07 (100.0%), 205.08 (12.0%), 206.07 (4.7%), 205.07 (1.5%)

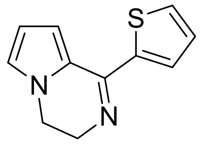

32a

m/z: 202.06 (100.0%), 203.06 (12.8%), 204.05 (4.5%)

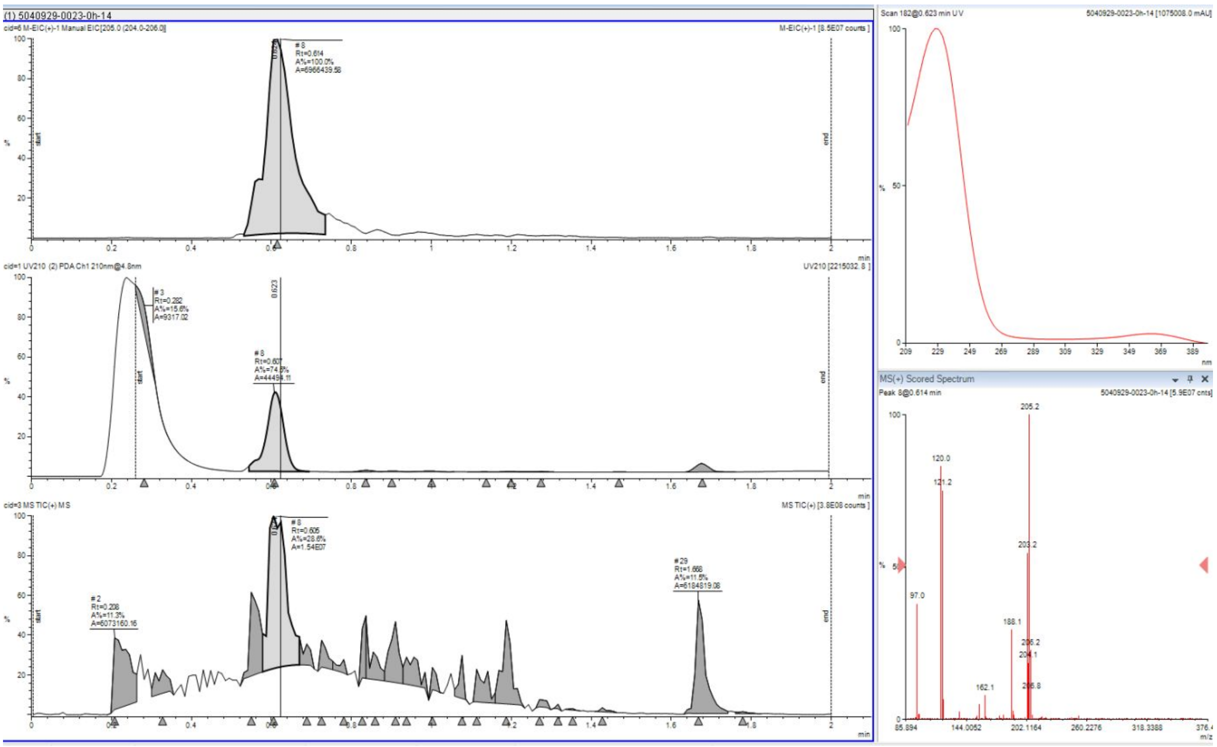

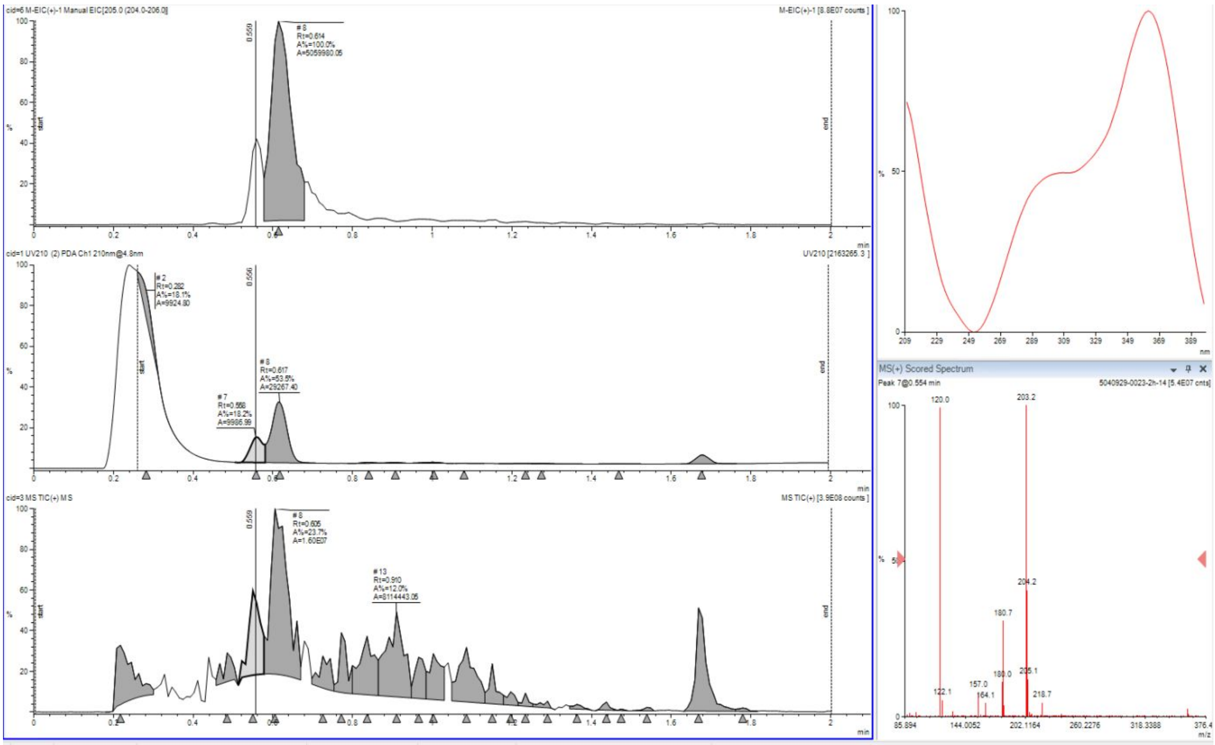
cid=6 M-EIC(+)-1 Manual EIC[205.0 (204.0-206.0)]
M-EIC(+)-1 [8.8E07 counts]
# 8
Rt=0.614
A%=100.0%
A=5059980.05
cid=1 UV210 (2) PDA Ch1 210nm@4.8nm
UV210 [2163265.3]
# 2
Rt=0.282
A%=18.1%
A=9924.80
# 7
Rt=0.558
A%=18.2%
A=9986.99
# 8
Rt=0.617
A%=53.5%
A=29267.40
cid=3 MS TIC(+) MS
MS TIC(+) [3.9E08 counts]
# 8
Rt=0.605
A%=23.7%
A=1.60E07
# 13
Rt=0.910
A%=12.0%
A=8114443.05
MS(+) Scored Spectrum
Peak 7@0.554 min
5040929-0023-2h-14 [5.4E07 cnts]
120.0
203.2
204.2
180.7
122.1
157.0
180.0
205.1
218.7

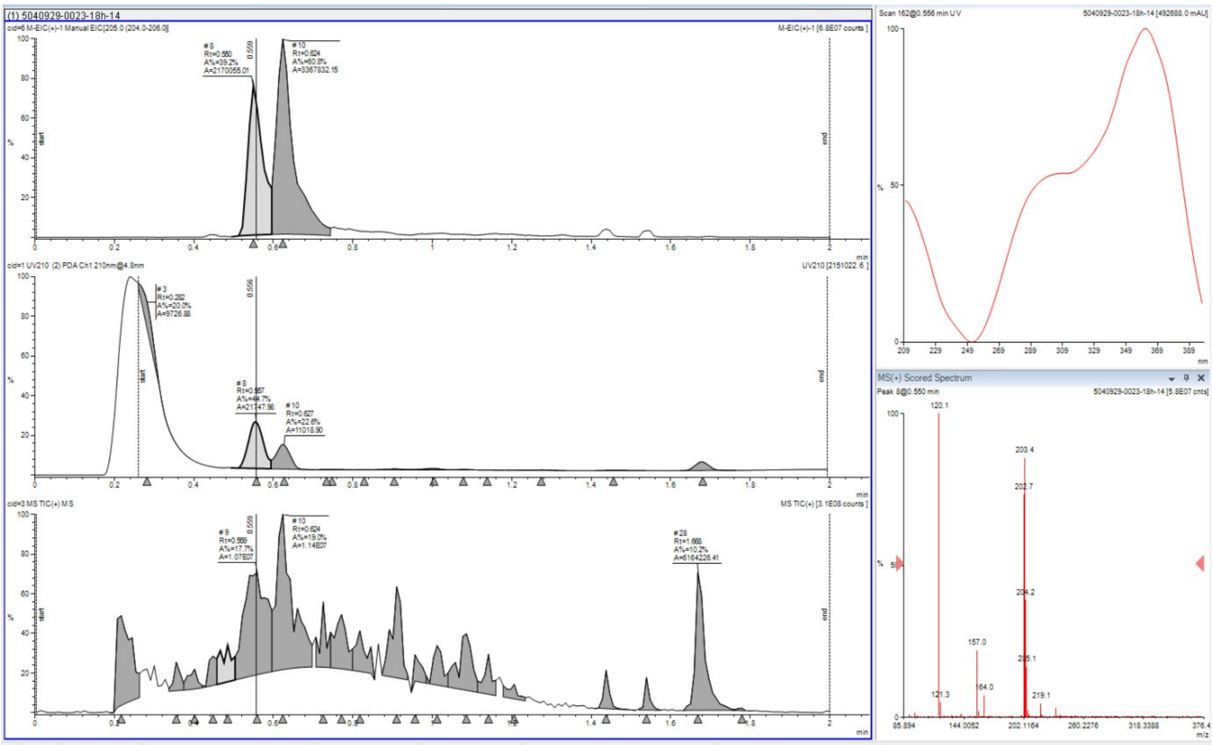
(1) 5040929-0023-18h-14
cid=6 M-EIC(+)-1 Manual EIC[205.0 (204.0-206.0)]
M-EIC(+)-1 [6.8E07 counts]
# 8
Rt=0.550
A%=39.2%
A=2170055.01
# 10
Rt=0.624
A%=60.8%
A=3367832.15
cid=1 UV210 (2) PDA Ch1 210nm@4.8nm
UV210 [2151022.6]
# 3
Rt=0.282
A%=20.0%
A=9726.88
# 8
Rt=0.557
A%=44.7%
A=21747.98
# 10
Rt=0.627
A%=22.6%
A=11018.90
cid=3 MS TIC(+) MS
MS TIC(+) [3.1E08 counts]
# 9
Rt=0.559
A%=17.7%
A=1.07E07
# 10
Rt=0.624
A%=19.0%
A=1.14E07
# 28
Rt=1.666
A%=10.2%
A=6164226.41
Scan 162@0.556 min UV
5040929-0023-18h-14 [492688.0 mAU]
MS(+) Scored Spectrum
Peak 8@0.550 min
5040929-0023-18h-14 [5.8E07 cnts]
120.1
203.4
202.7
204.2
157.0
205.1
121.3
164.0
219.1

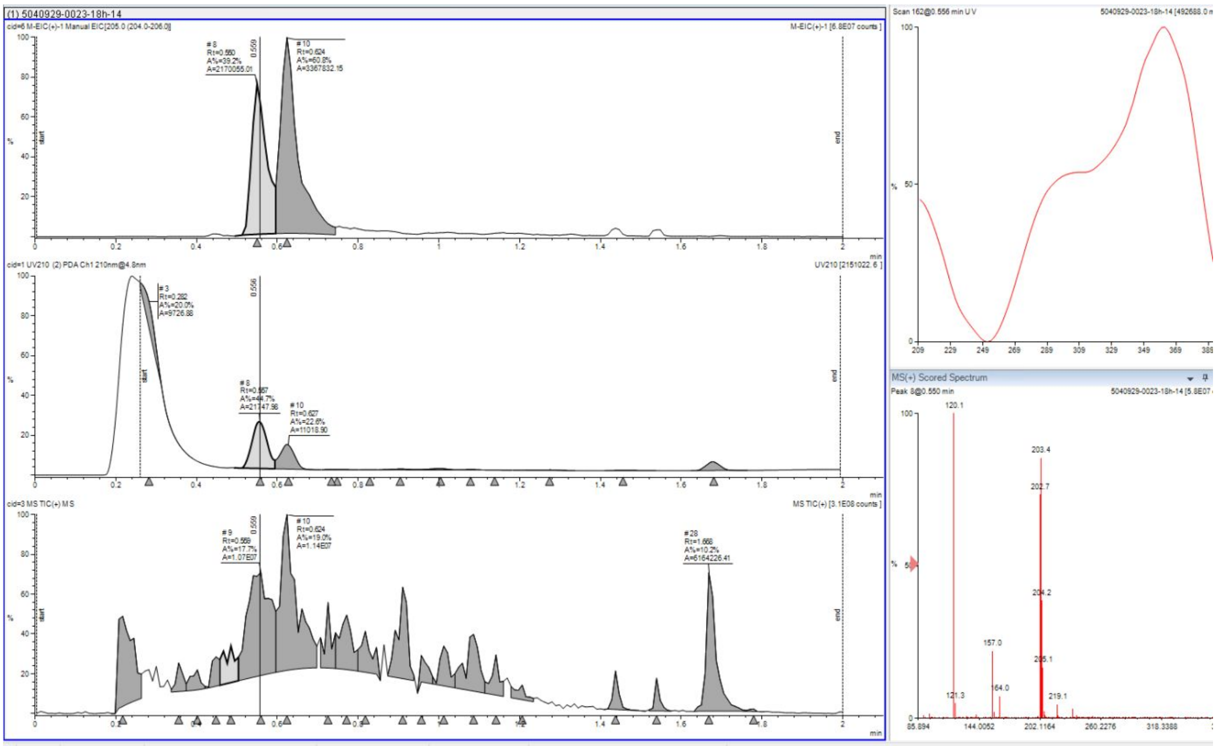

When reaction set up without CH_3NO_2

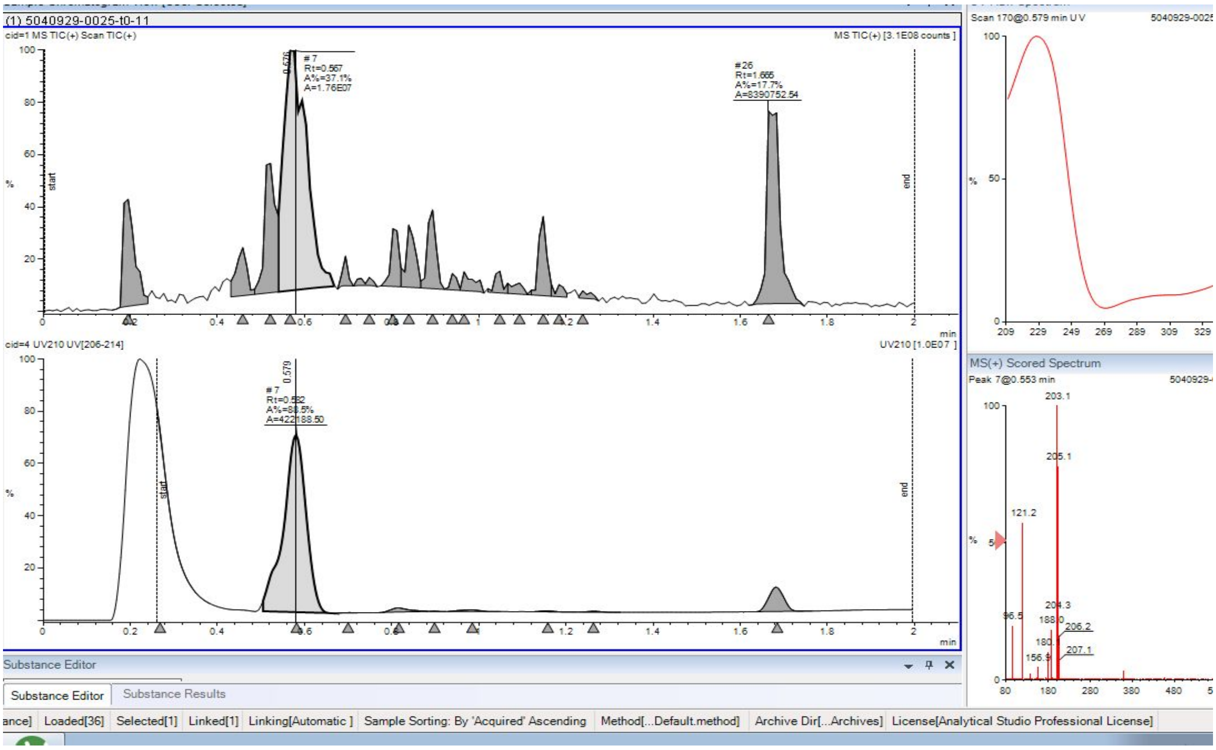

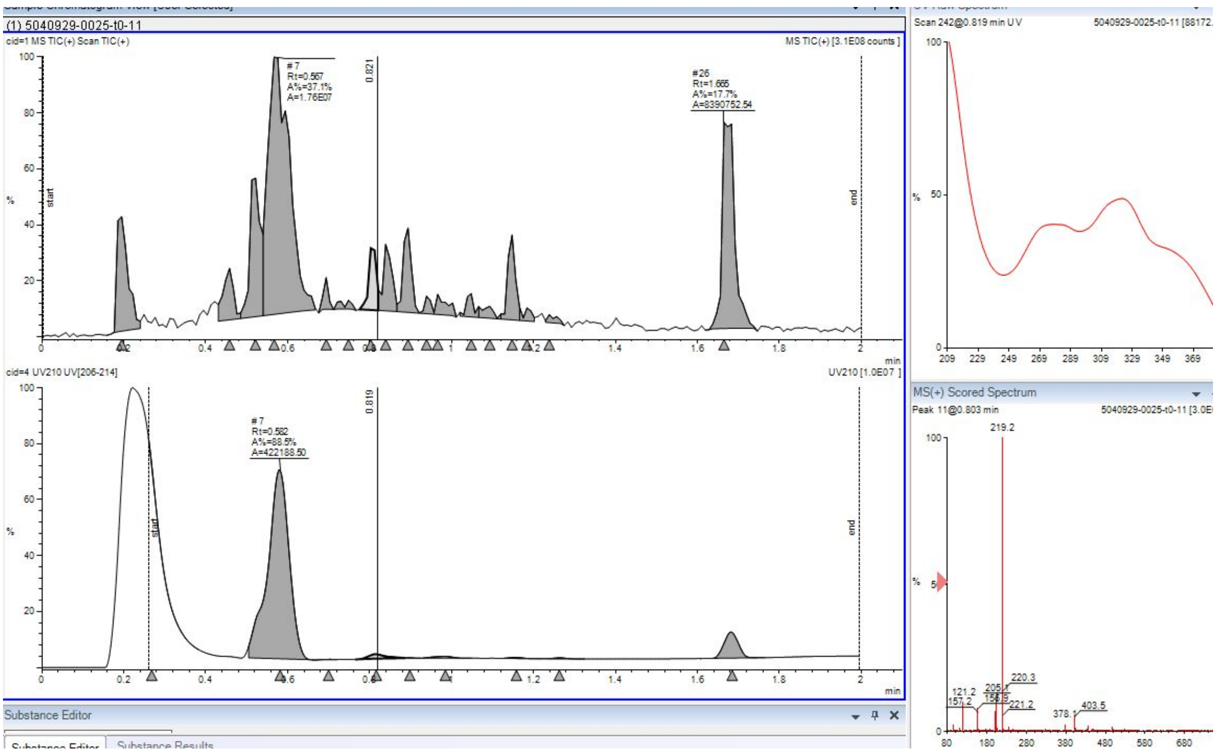
(1) 5040929-0025-t0-11
cid=1 MS TIC(+) Scan TIC(+)
MS TIC(+) [3.1E08 counts]
# 7
Rt=0.567
A%=37.1%
A=1.76E07
0.821
#26
Rt=1.665
A%=17.7%
A=8390752.54
start
end
cid=4 UV210 UV[206-214]
UV210 [1.0E07]
# 7
Rt=0.582
A%=88.5%
A=422188.50
0.819
Scan 242@0.819 min UV
5040929-0025-t0-11
MS(+) Scored Spectrum
Peak 11@0.803 min
219.2
121.2
157.2
205.1
220.3
221.2
378.1
403.5
Substance Editor
Substance Editor
Substance Results

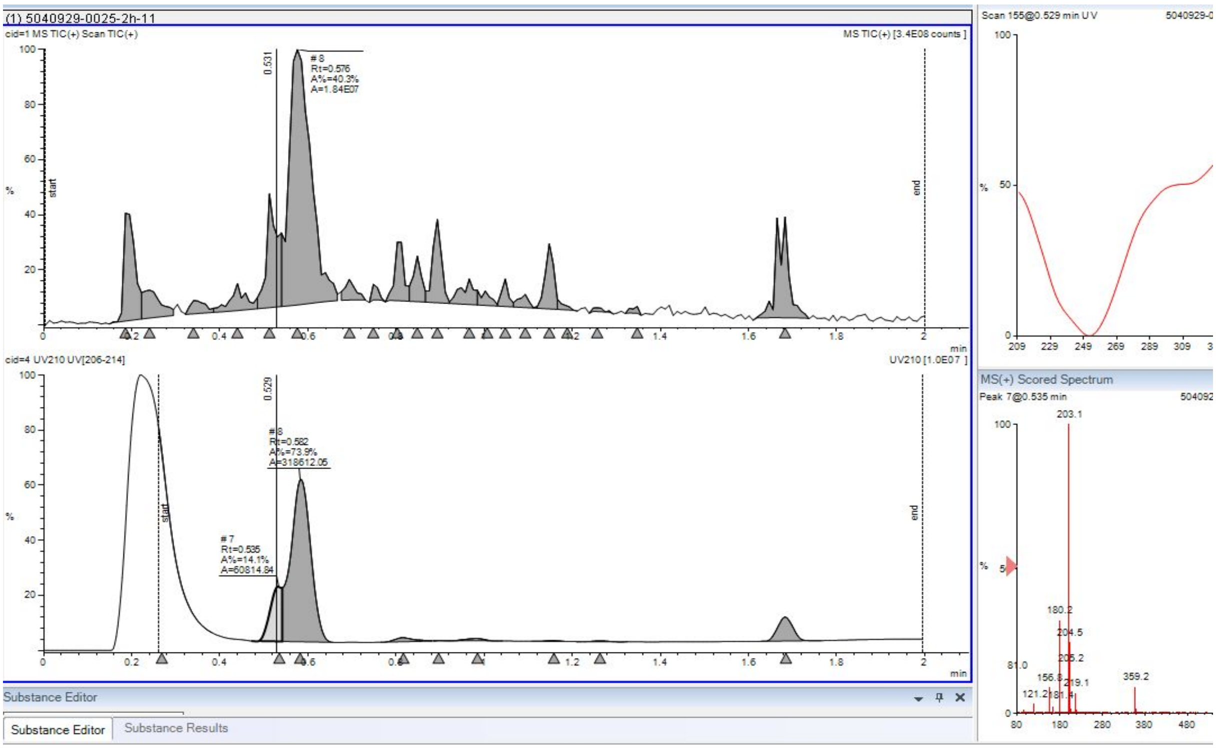
(1) 5040929-0025-2h-11
cid=1 MS TIC(+) Scan TIC(+)
MS TIC(+) [3.4E08 counts]
0.531
# 8
Rt=0.576
A%=40.3%
A=1.84E07
start
end
cid=4 UV210 UV[206-214]
UV210 [1.0E07]
0.529
# 8
Rt=0.582
A%=73.9%
A=318612.05
# 7
Rt=0.535
A%=14.1%
A=60814.84
Scan 155@0.529 min UV
MS(+) Scored Spectrum
Peak 7@0.535 min
203.1
180.2
204.5
205.2
81.0
156.8
219.1
121.2
359.2
Substance Editor
Substance Editor
Substance Results

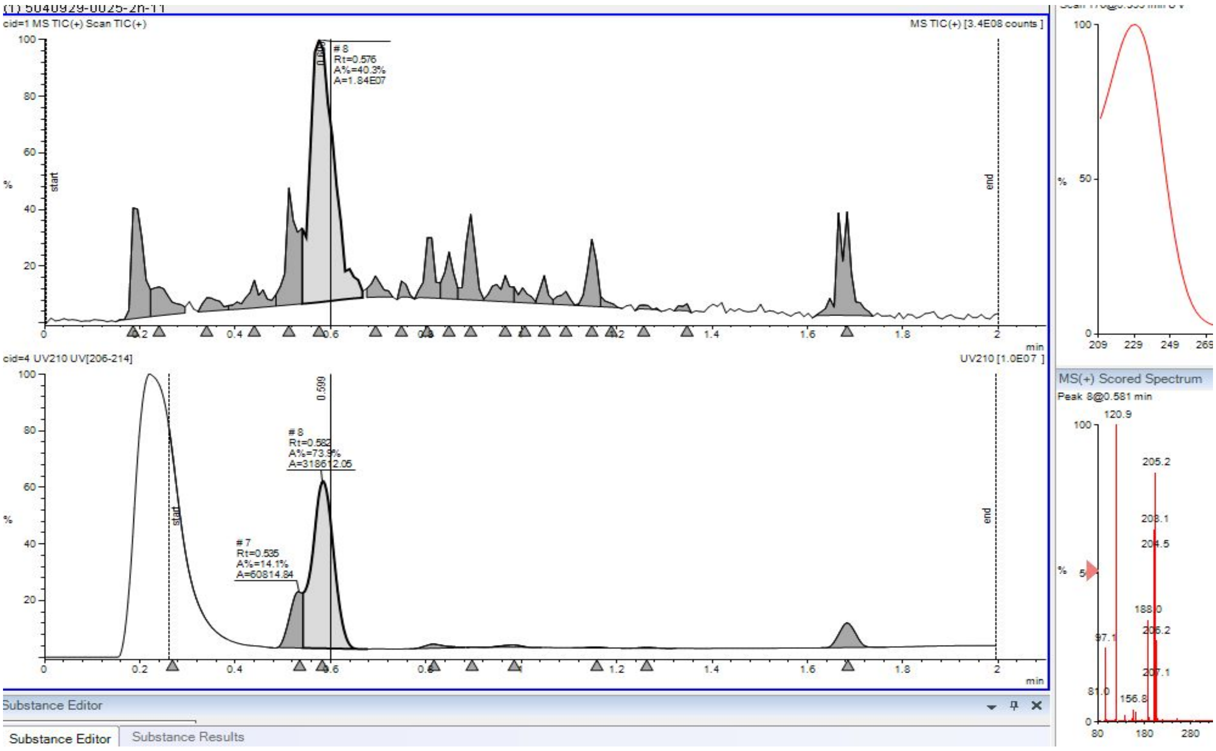

cid=1 MS TIC(+) Scan TIC(+)
MS TIC(+) [3.4E08 counts]
# 8
Rt=0.576
A%=40.3%
A=1.84E07
cid=4 UV210 UV[206-214]
UV210 [1.0E07]
# 8
Rt=0.582
A%=73.9%
A=318612.05
# 7
Rt=0.535
A%=14.1%
A=60814.84
Substance Editor
Substance Results
MS(+) Scored Spectrum
Peak 8@0.581 min
120.9
205.2

Reaction with 6-bromospiro[benzo[e][1,3]oxazine-2,4'-piperidin]-4(3H)-one (33)

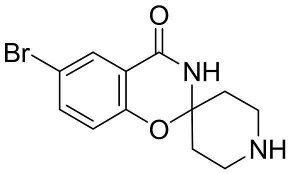

33

m/z: 296.02 (100.0%), 298.01 (97.3%), 297.02 (13.2%), 299.02 (12.9%), 298.02 (1.3%), 300.02 (1.2%)

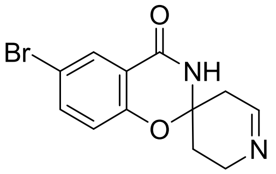

33a

m/z: 294.00 (100.0%), 296.00 (97.8%), 295.00 (13.8%), 297.00 (13.6%)

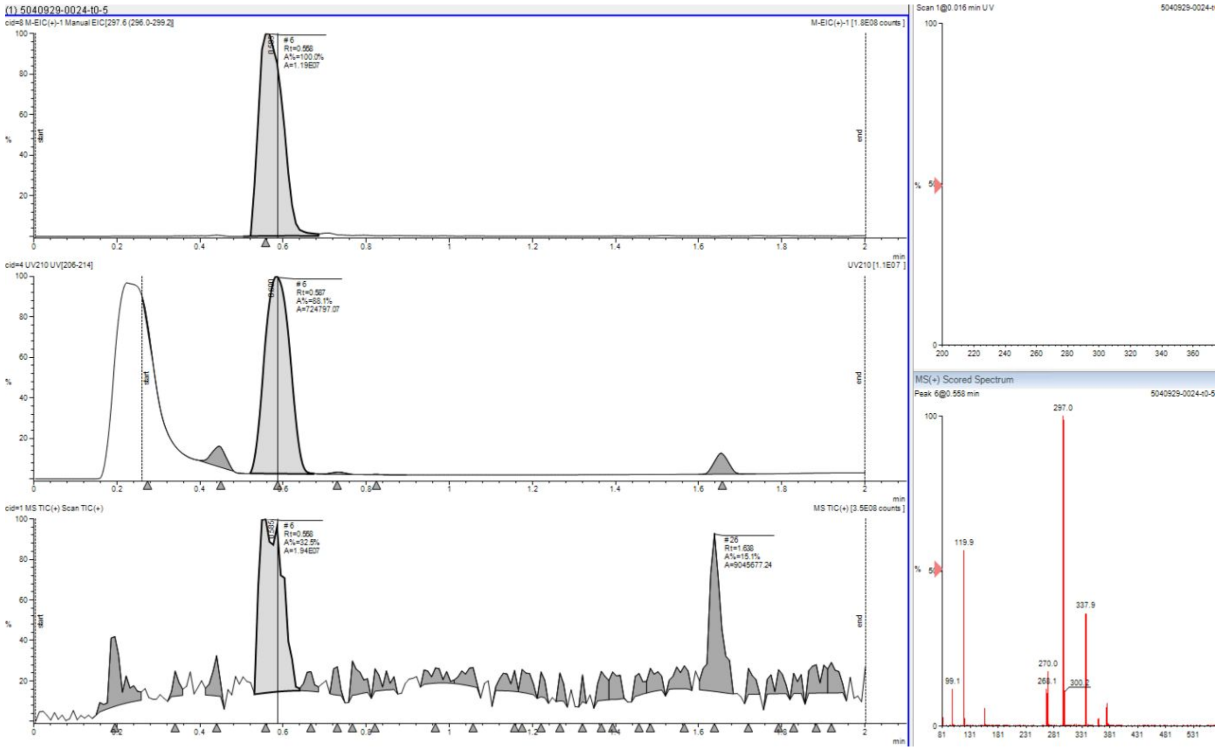

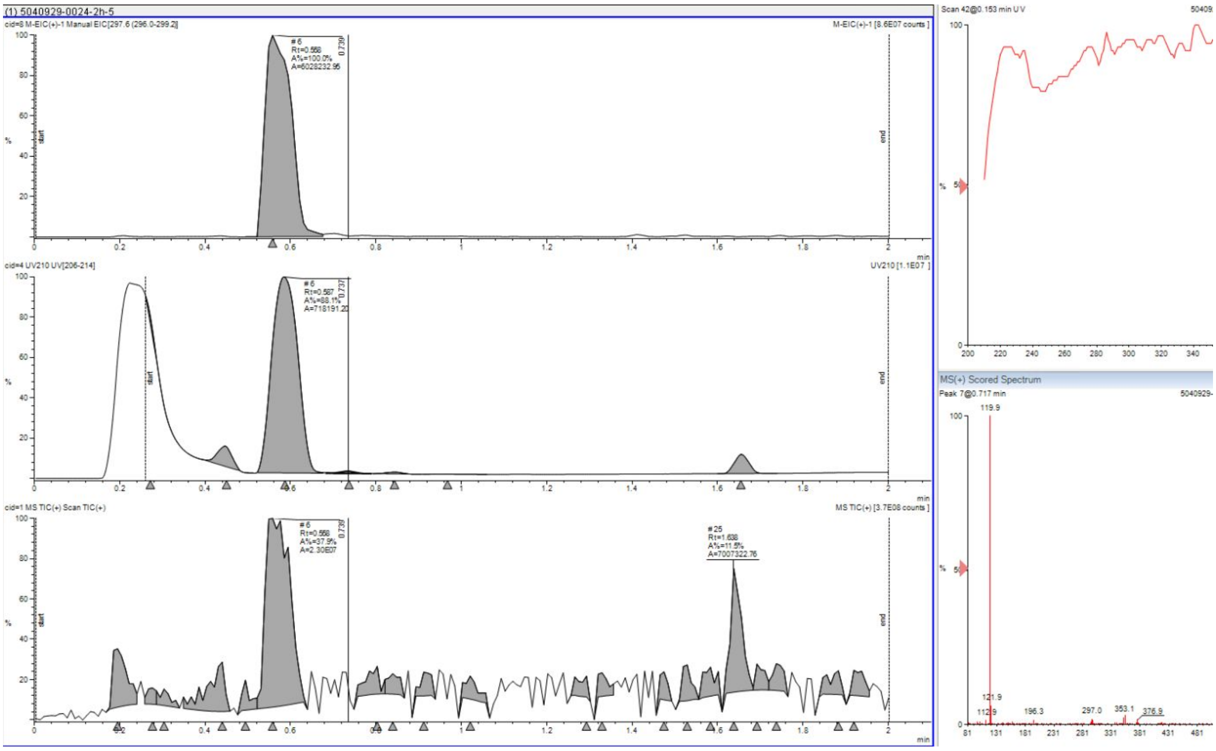

(1) 5040929-0024-2h-5
cid=8 M-EIC(+)-1 Manual EIC[297.6 (296.0-299.2)]
M-EIC(+)-1 [8.6E07 counts]
cid=4 UV210 UV[206-214]
UV210 [1.1E07]
cid=1 MS TIC(+) Scan TIC(+)
MS TIC(+) [3.7E08 counts]
Scan 42@0.153 min UV
MS(+) Scored Spectrum
Peak 7@0.717 min
119.9

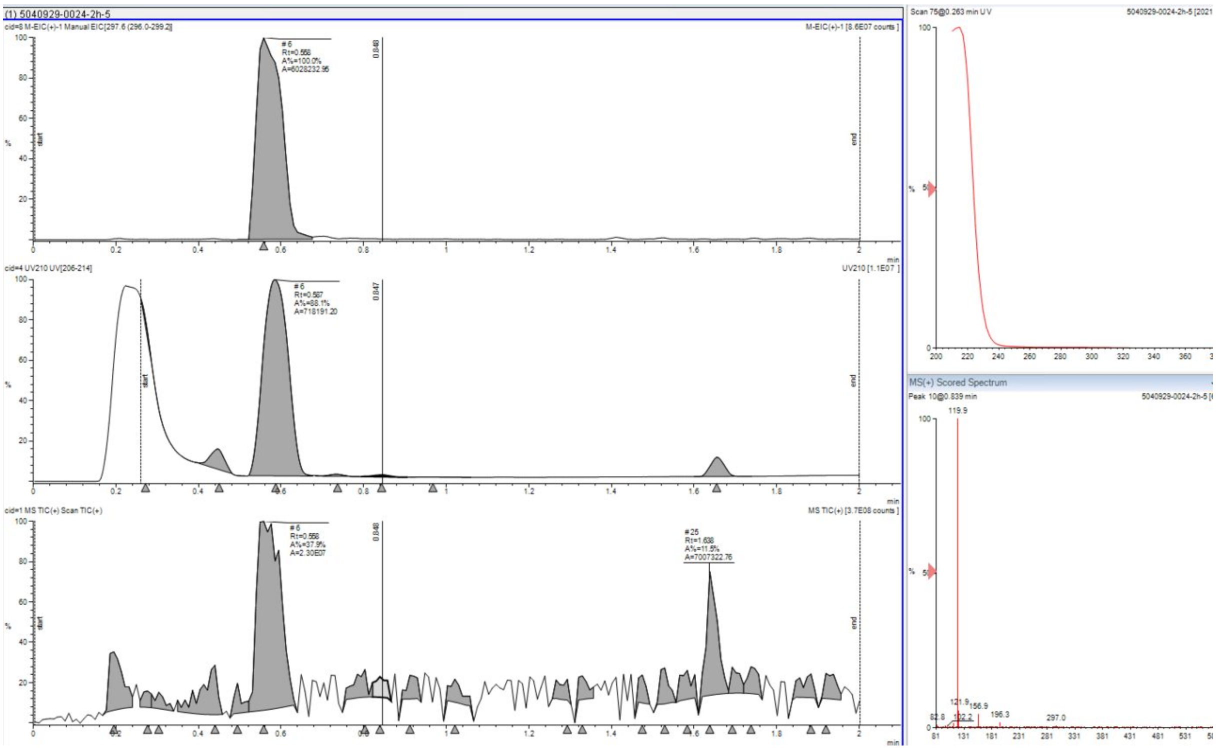

(1) 5040929-0024-2h-5
cid=8 M-EIC(+)-1 Manual EIC[297.6 (296.0-299.2)]
M-EIC(+)-1 [8.6E07 counts]
cid=4 UV210 UV[206-214]
UV210 [1.1E07]
cid=1 MS TIC(+) Scan TIC(+)
MS TIC(+) [3.7E08 counts]
Scan 75@0.263 min UV
MS(+) Scored Spectrum
Peak 10@0.839 min
119.9

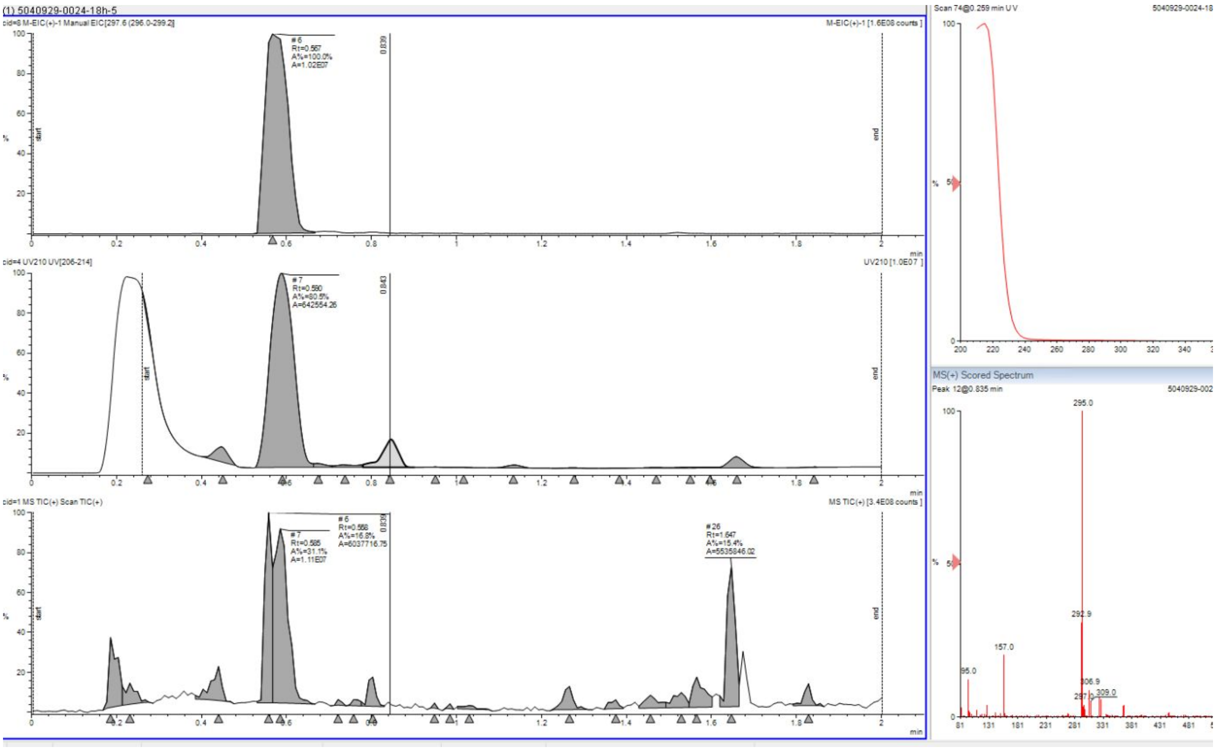

When reaction set up without CH_3NO_2

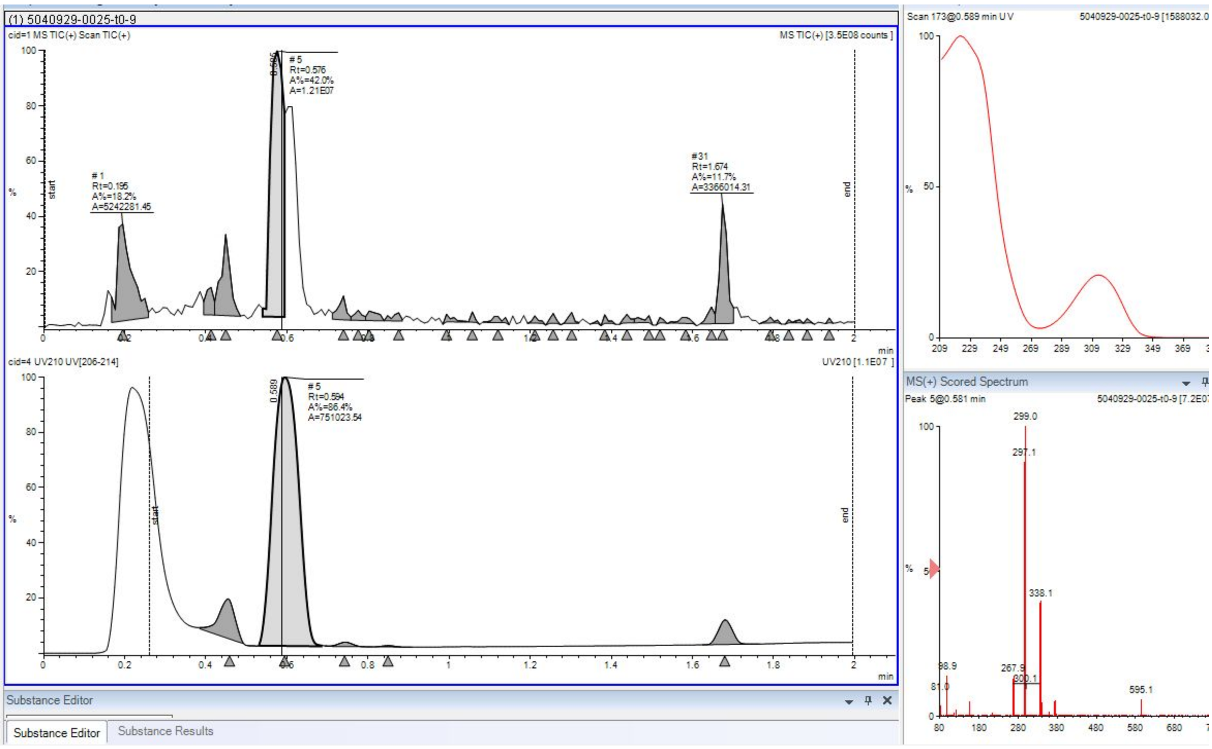

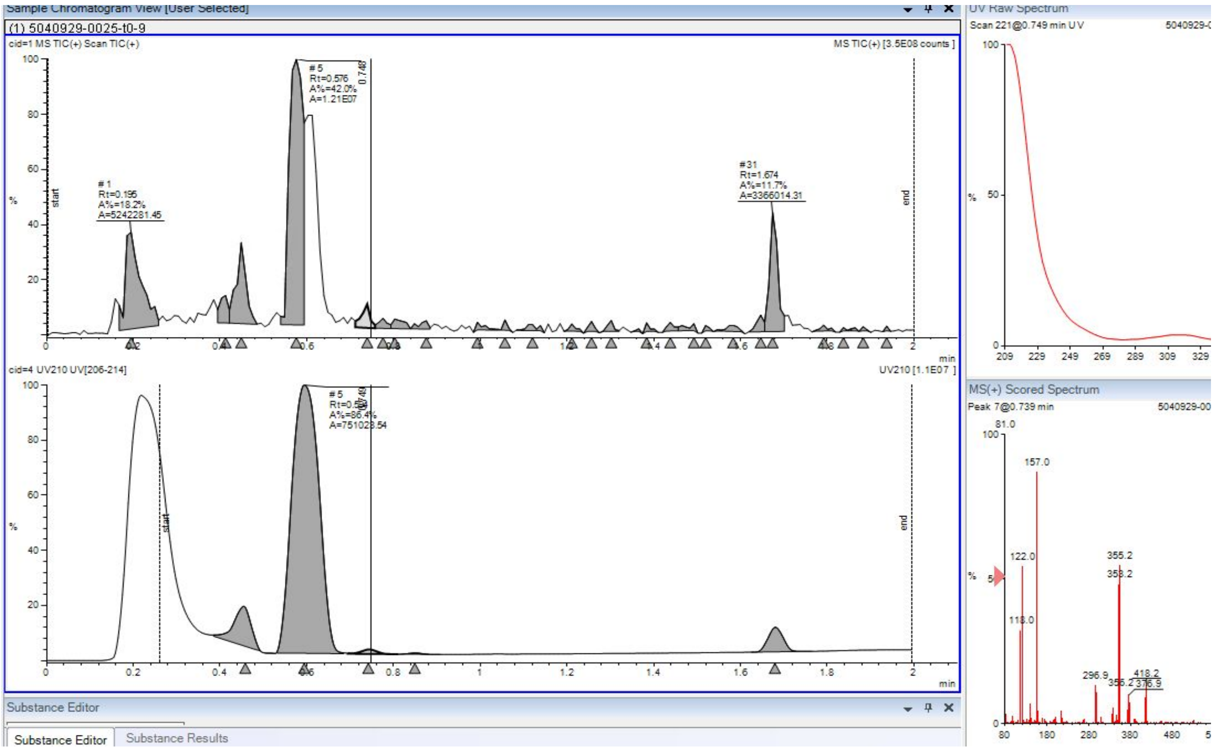
Sample Chromatogram View [User Selected]
(1) 5040929-0025-t0-9
cid=1 MS TIC(+) Scan TIC(+)
MS TIC(+) [3.5E08 counts]
# 1
Rt=0.195
A%=18.2%
A=5242281.45
# 5
Rt=0.576
A%=42.0%
A=1.21E07
0.749
#31
Rt=1.674
A%=11.7%
A=3366014.31
start
end
min
cid=4 UV210 UV[206-214]
UV210 [1.1E07]
5
A%=86.4%
A=751023.54
UV Raw Spectrum
Scan 221@0.749 min UV
MS(+) Scored Spectrum
Peak 7@0.739 min
81.0
157.0
122.0
118.0
355.2
353.2
296.9
356.2
418.2
376.9
Substance Editor
Substance Results

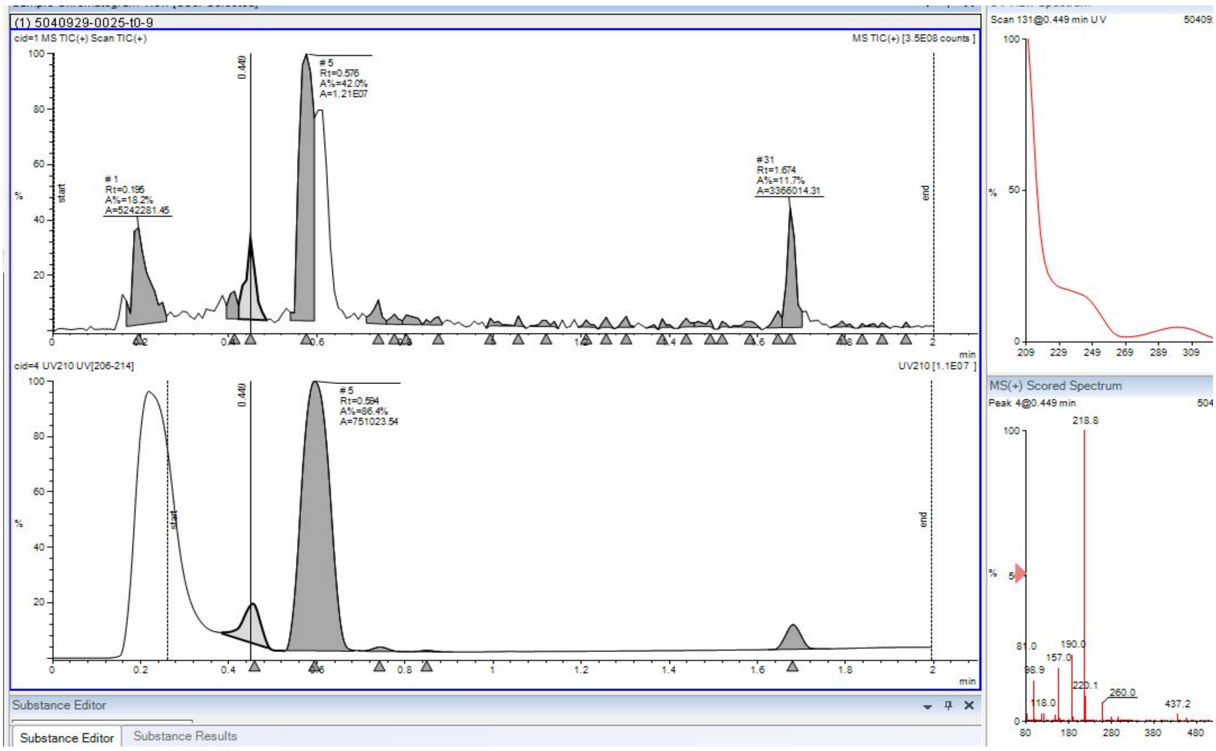
(1) 5040929-0025-t0-9
cid=1 MS TIC(+) Scan TIC(+)
MS TIC(+) [3.5E08 counts]
0.449
# 1
Rt=0.195
A%=18.2%
A=5242281.45
# 5
Rt=0.576
A%=42.0%
A=1.21E07
#31
Rt=1.674
A%=11.7%
A=3366014.31
start
end
min
cid=4 UV210 UV[206-214]
UV210 [1.1E07]
# 5
Rt=0.594
A%=86.4%
A=751023.54
Scan 131@0.449 min UV
MS(+) Scored Spectrum
Peak 4@0.449 min
218.8
81.0
98.9
157.0
190.0
118.0
220.1
260.0
437.2
Substance Editor
Substance Results

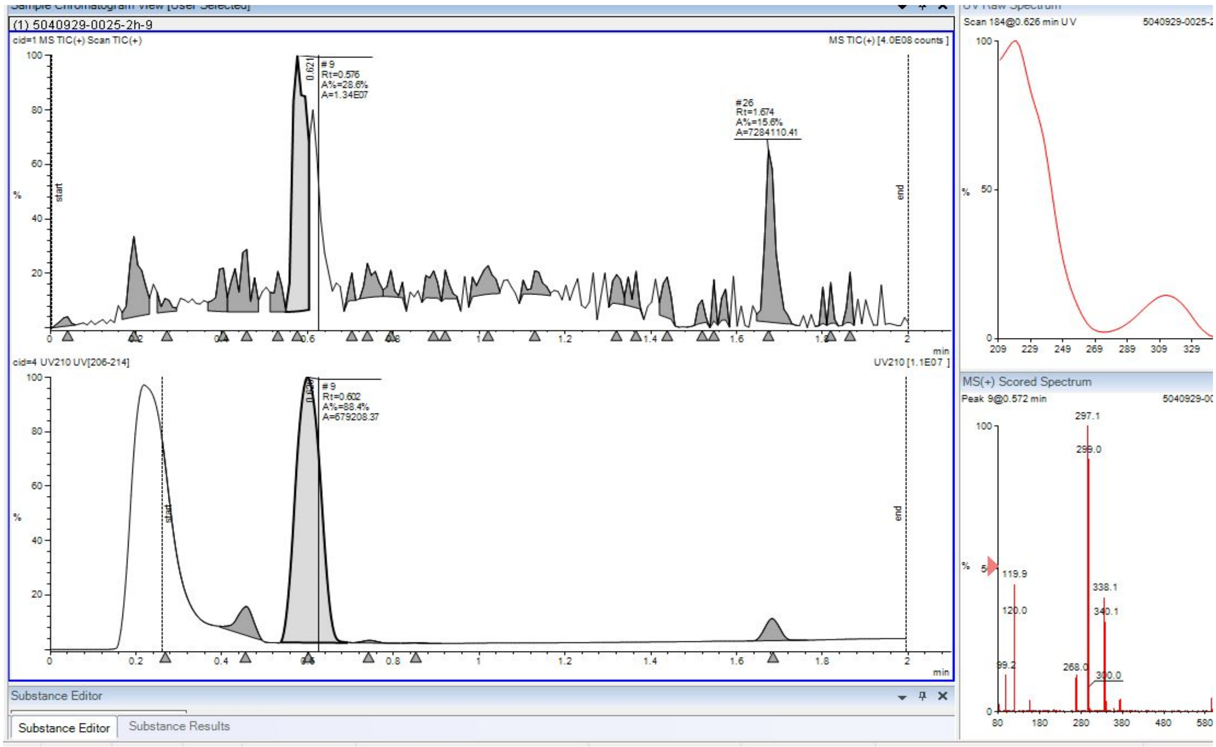
(1) 5040929-0025-2h-9
cid=1 MS TIC(+) Scan TIC(+)
MS TIC(+) [4.0E08 counts]
#9
Rt=0.576
A%=28.6%
A=1.34E07
#26
Rt=1.674
A%=15.6%
A=7284110.41
cid=4 UV210 UV[206-214]
UV210 [1.1E07]
#9
Rt=0.602
A%=88.4%
A=679208.37
Substance Editor
Substance Results
Scan 184@0.626 min UV
MS(+) Scored Spectrum
Peak 9@0.572 min
297.1
299.0
119.9
120.0
338.1
340.1
99.2
268.0
300.0

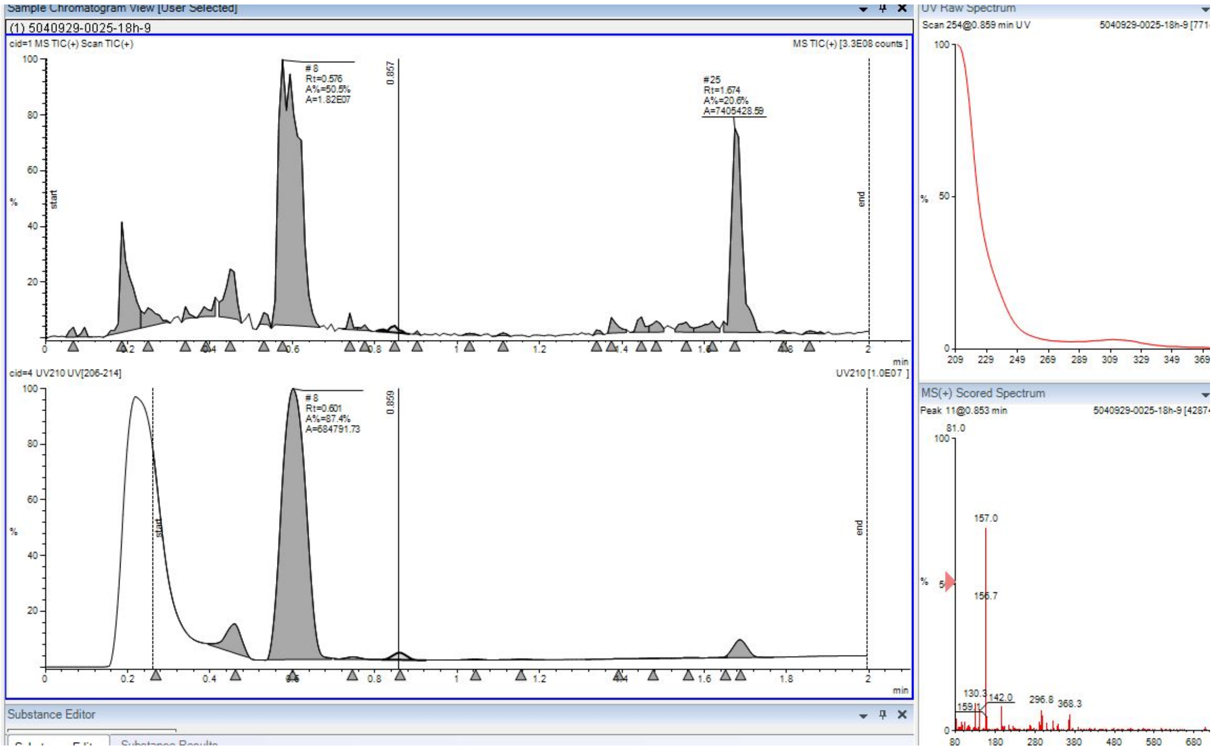
Sample Chromatogram View [User Selected]
(1) 5040929-0025-18h-9
cid=1 MS TIC(+) Scan TIC(+)
MS TIC(+) [3.3E08 counts]
#8
Rt=0.576
A%=50.5%
A=1.82E07
#25
Rt=1.674
A%=20.6%
A=7405428.59
cid=4 UV210 UV[206-214]
UV210 [1.0E07]
#8
Rt=0.601
A%=87.4%
A=684791.73
Substance Editor
Scan 254@0.859 min UV
5040929-0025-18h-9
MS(+) Scored Spectrum
Peak 11@0.853 min
81.0
157.0
156.7
130.3
142.0
296.8
368.3

Reaction with *N,N*-dimethyl-3-((5-(4,4,5,5-tetramethyl-1,3,2-dioxaborolan-2-yl)pyridin-2-yl)oxy)propan-1-amine (34)

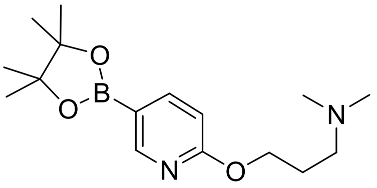

34

m/z: 306.21 (100.0%), 305.22 (24.8%), 307.21 (18.0%), 306.22 (4.4%), 308.22 (2.1%)

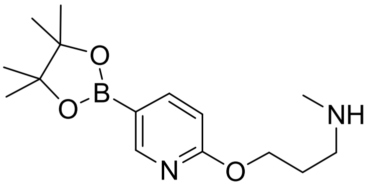

34a

m/z: 292.20 (100.0%), 291.20 (23.8%), 293.20 (16.1%), 294.20 (1.9%)

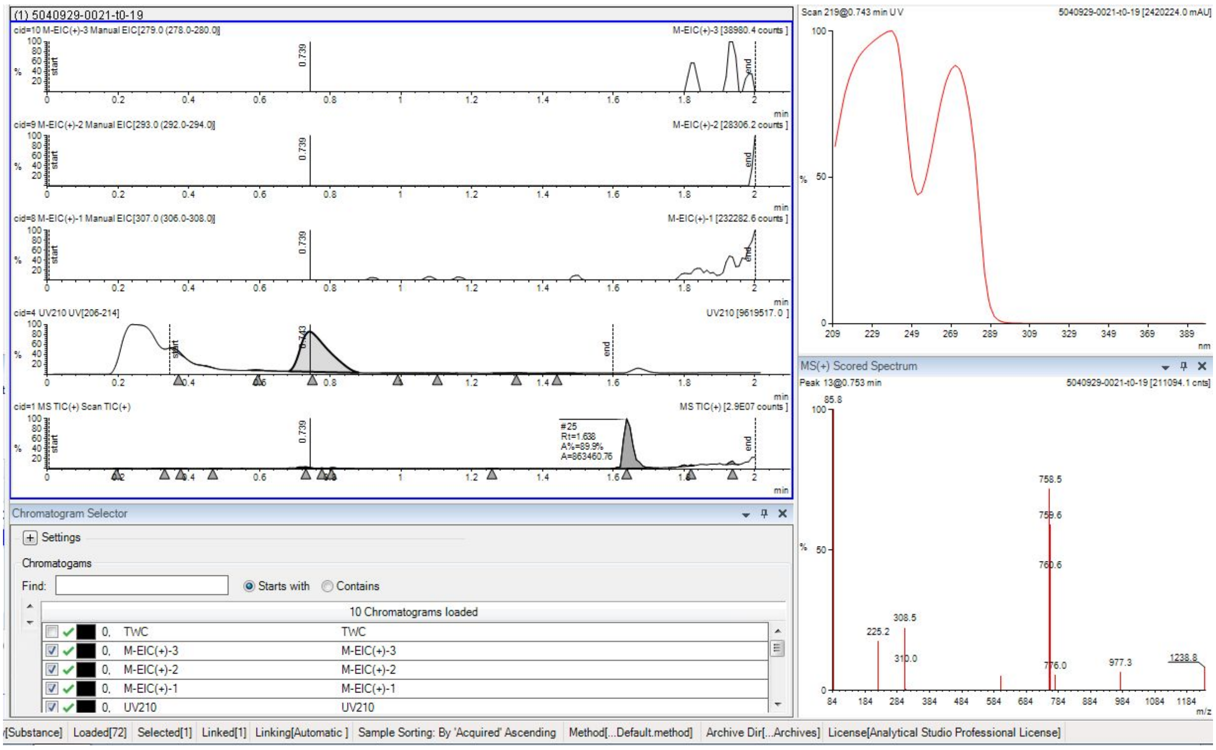

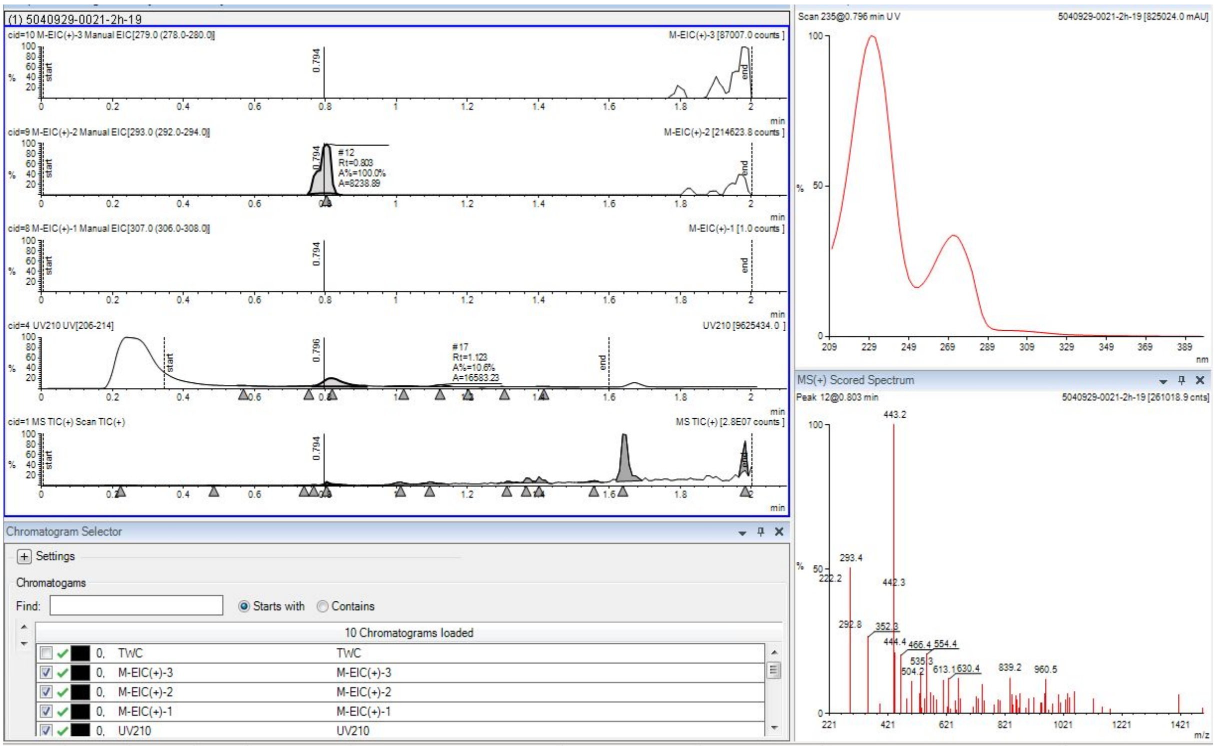

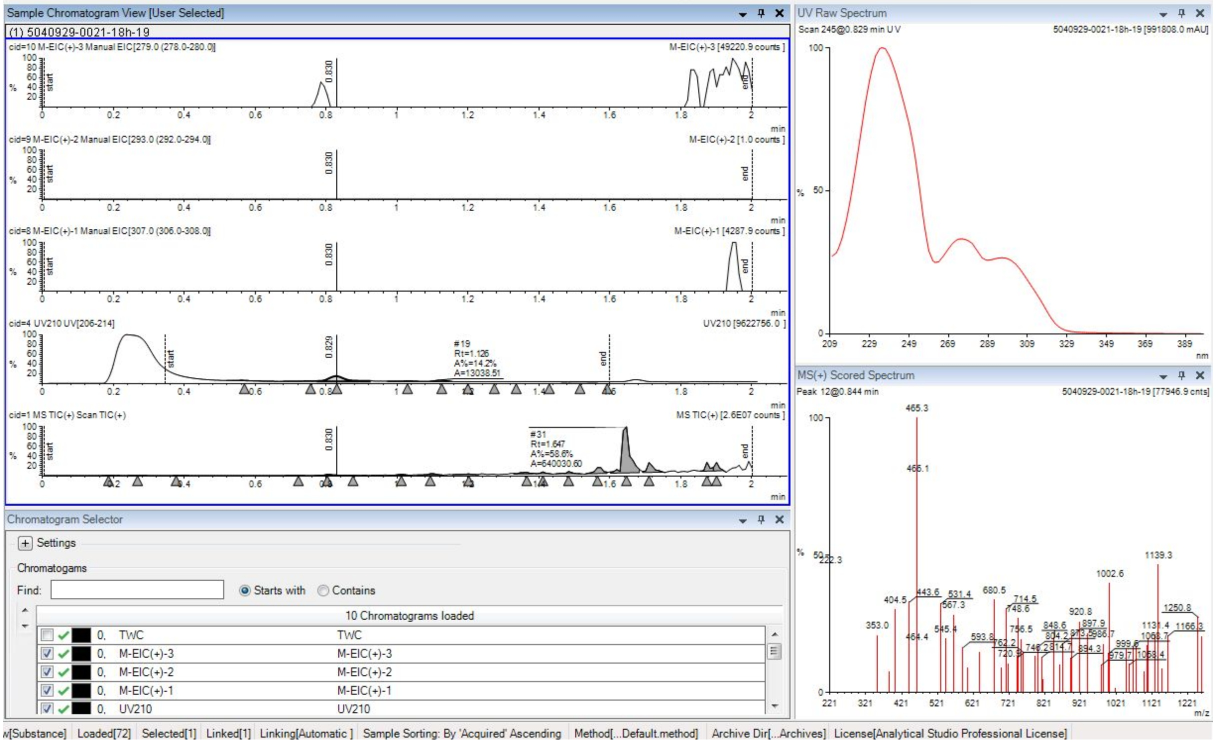

Reaction with 5-((3,4-dimethoxyphenethyl)(methyl)amino)-2-(3,4-dimethoxyphenyl)-2-isopropylpentanenitrile (35)

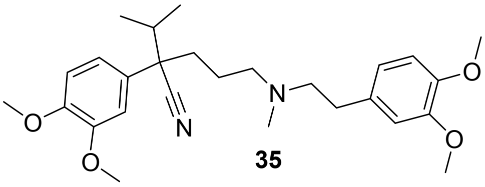

35

m/z: 454.28 (100.0%), 455.29 (29.8%), 456.29 (5.1%)

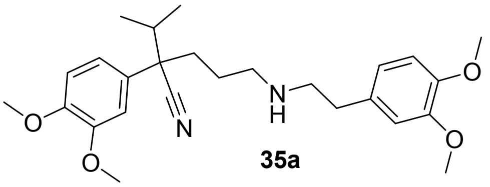

35a

m/z: 440.27 (100.0%), 441.27 (28.7%), 442.27 (4.8%)

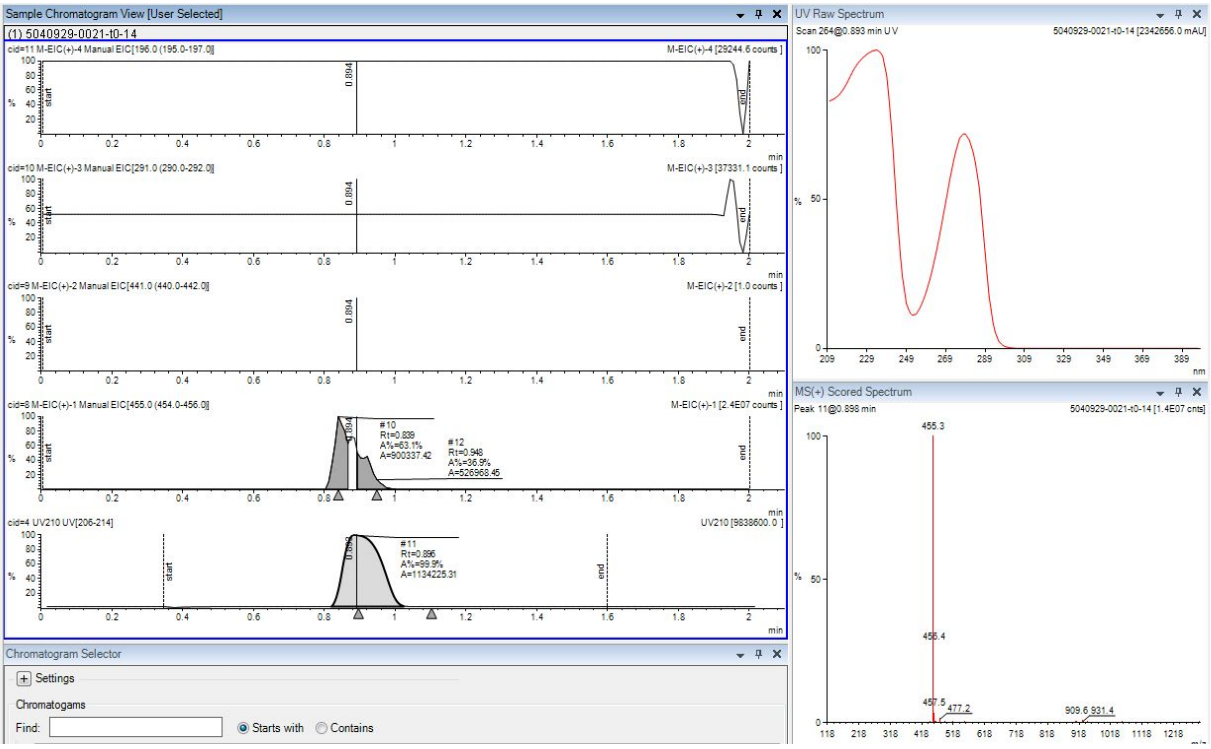

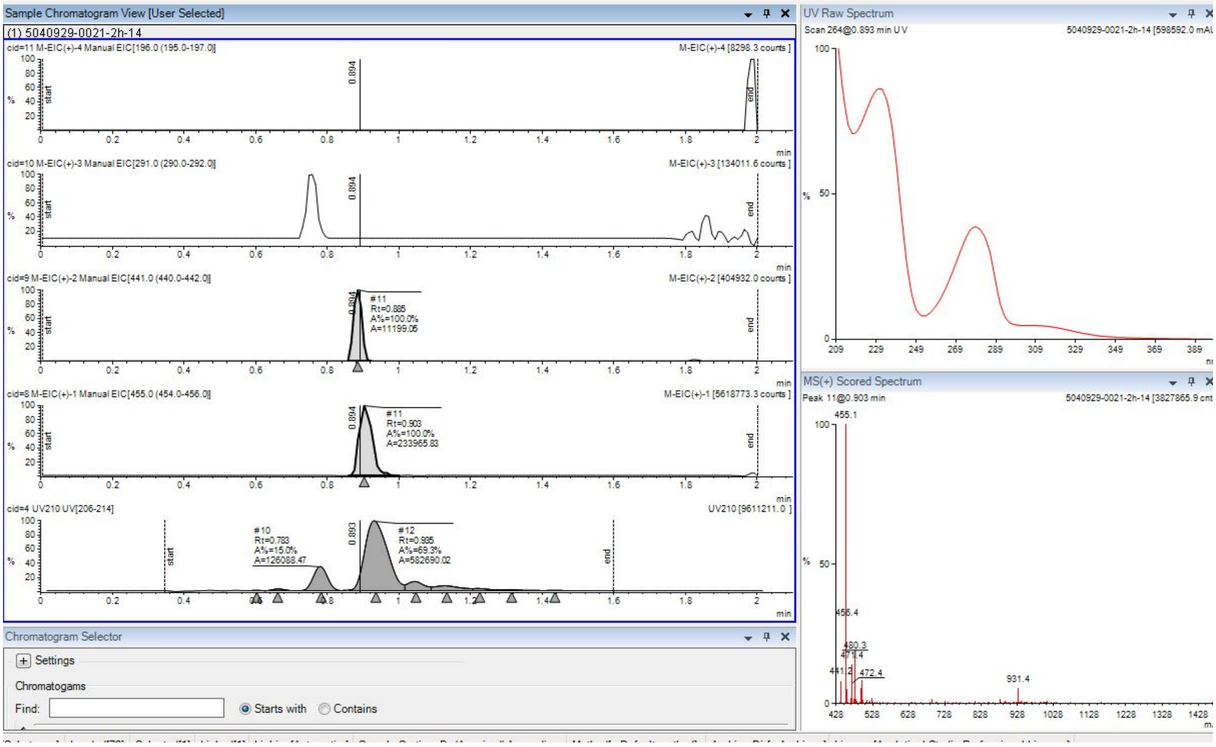
Sample Chromatogram View [User Selected]
(1) 5040929-0021-2h-14
cid=11 M-EIC(+)-4 Manual EIC[196.0 (195.0-197.0)]
M-EIC(+)-4 [8298.3 counts]
cid=10 M-EIC(+)-3 Manual EIC[291.0 (290.0-292.0)]
M-EIC(+)-3 [134011.6 counts]
cid=9 M-EIC(+)-2 Manual EIC[441.0 (440.0-442.0)]
M-EIC(+)-2 [404932.0 counts]
#11 Rt=0.885 A%=100.0% A=11199.05
cid=8 M-EIC(+)-1 Manual EIC[455.0 (454.0-456.0)]
M-EIC(+)-1 [5618773.3 counts]
#11 Rt=0.903 A%=100.0% A=233965.83
cid=4 UV210 UV[206-214]
UV210 [9611211.0]
#10 Rt=0.783 A%=15.0% A=126088.47
#12 Rt=0.935 A%=69.3% A=582690.02
Chromatogram Selector
Settings
Chromatogams
Find:
Starts with
Contains
UV Raw Spectrum
Scan 264@0.893 min UV
MS(+) Scored Spectrum
Peak 11@0.903 min
455.1
456.4
480.3
441.2
472.4
931.4

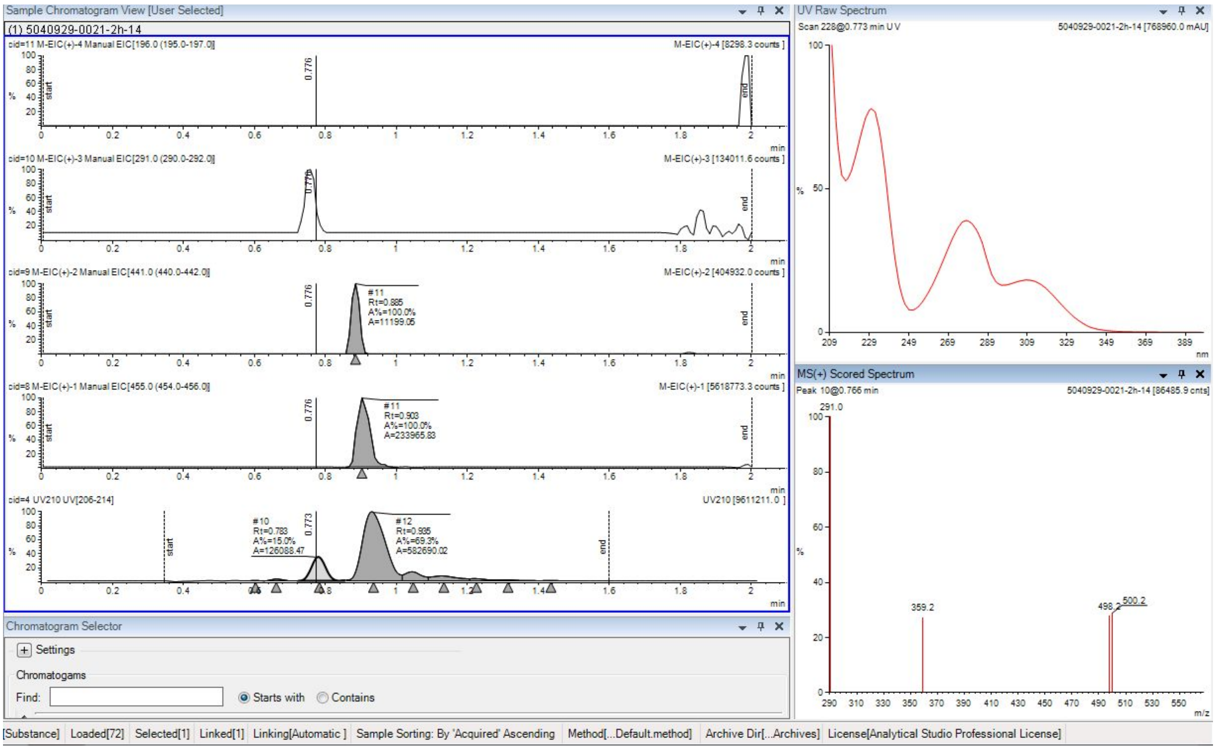
Sample Chromatogram View [User Selected]
(1) 5040929-0021-2h-14
cid=11 M-EIC(+)-4 Manual EIC[196.0 (195.0-197.0)]
M-EIC(+)-4 [8298.3 counts]
cid=10 M-EIC(+)-3 Manual EIC[291.0 (290.0-292.0)]
M-EIC(+)-3 [134011.6 counts]
cid=9 M-EIC(+)-2 Manual EIC[441.0 (440.0-442.0)]
M-EIC(+)-2 [404932.0 counts]
#11 Rt=0.885 A%=100.0% A=11199.05
cid=8 M-EIC(+)-1 Manual EIC[455.0 (454.0-456.0)]
M-EIC(+)-1 [5618773.3 counts]
#11 Rt=0.903 A%=100.0% A=233965.83
cid=4 UV210 UV[206-214]
UV210 [9611211.0]
#10 Rt=0.783 A%=15.0% A=126088.47
#12 Rt=0.935 A%=69.3% A=582690.02
Chromatogram Selector
Settings
Chromatogams
Find:
Starts with
Contains
UV Raw Spectrum
Scan 228@0.773 min UV
5040929-0021-2h-14 [768960.0 mAU]
MS(+) Scored Spectrum
Peak 10@0.766 min
5040929-0021-2h-14 [86485.9 cnts]
291.0
359.2
498.2
500.2
[Substance] Loaded[72] Selected[1] Linked[1] Linking[Automatic] Sample Sorting: By 'Acquired' Ascending Method[...Default.method] Archive Dir[...Archives] License[Analytical Studio Professional License]

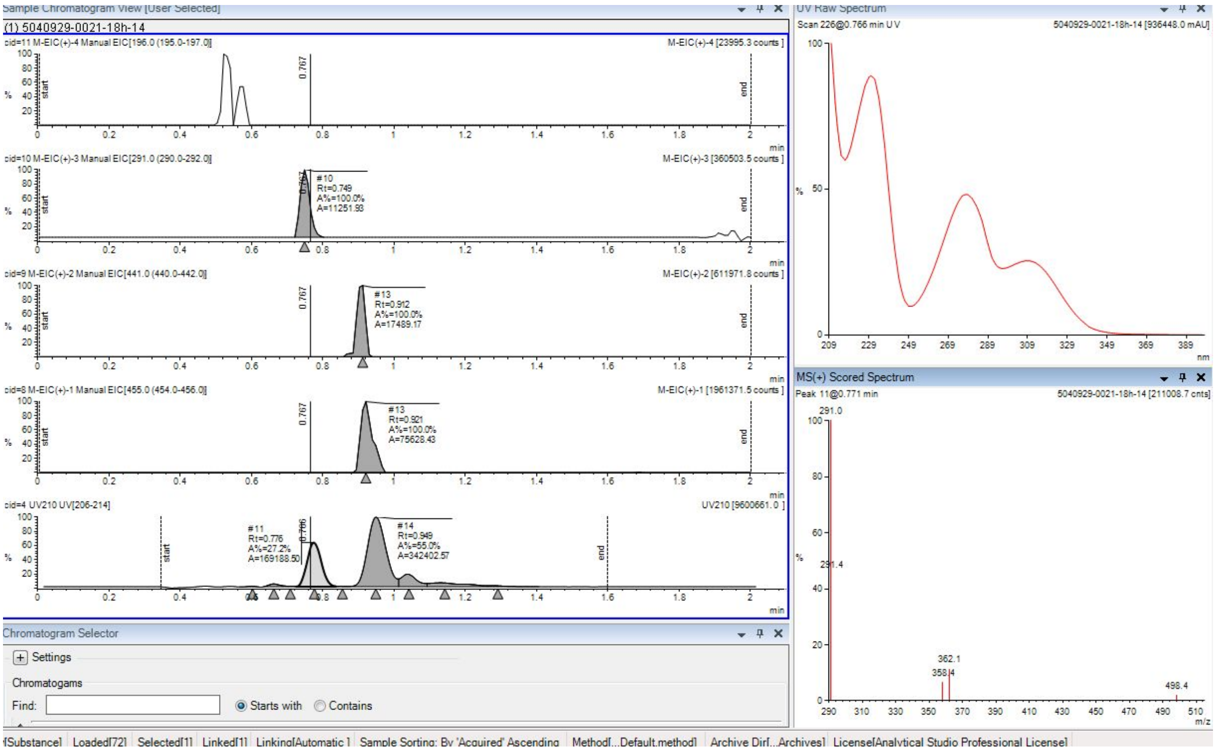
Sample Chromatogram View [User Selected]
(1) 5040929-0021-18h-14
UV Raw Spectrum
Scan 226@0.766 min UV
5040929-0021-18h-14 [936448.0 mAU]
MS(+) Scored Spectrum
Peak 11@0.771 min
5040929-0021-18h-14 [211008.7 cnts]
Chromatogram Selector
Settings
Chromatogams
Find:
Starts with
Contains

Reaction with 2-(benzylamino)-4-(4-cyclohexylphenyl)-4-oxobutanoic acid (36)

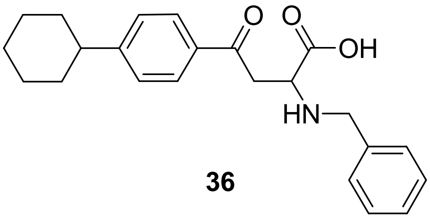

36

m/z: 365.20 (100.0%), 366.20 (25.4%), 367.21 (3.1%)

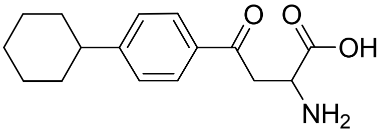

36a

m/z: 275.15 (100.0%), 276.16 (17.7%), 277.16 (2.1%)

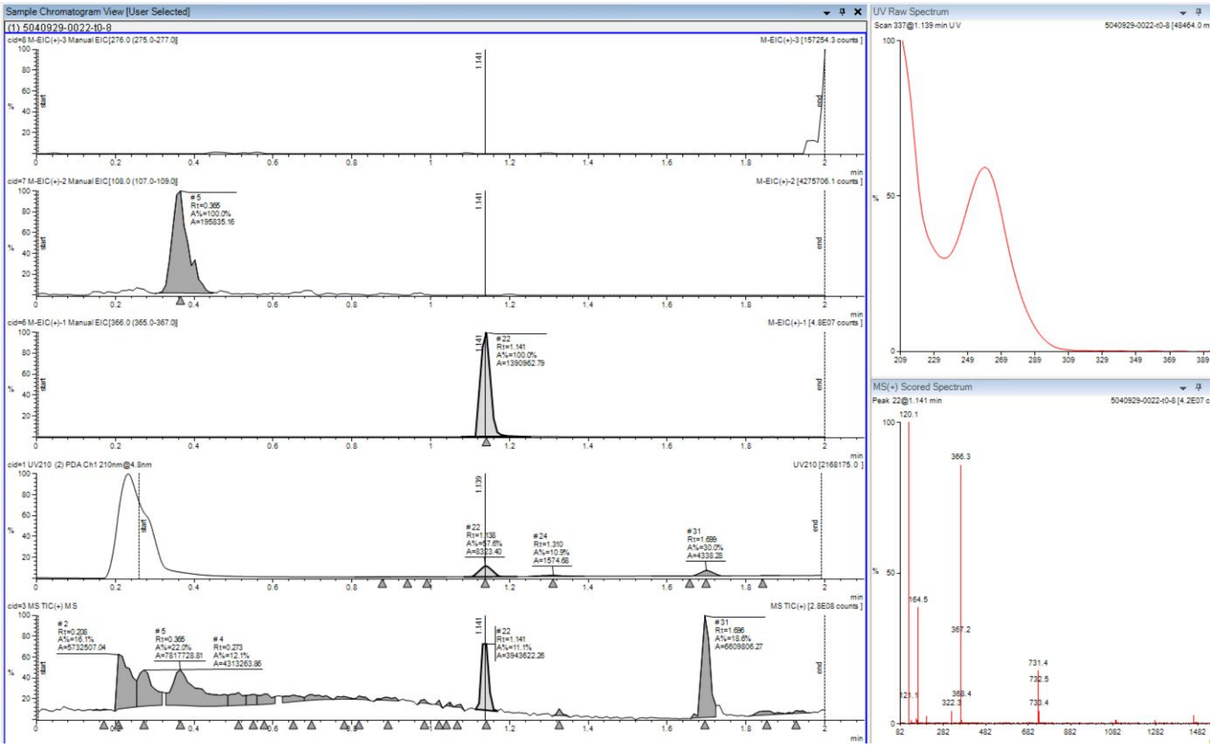

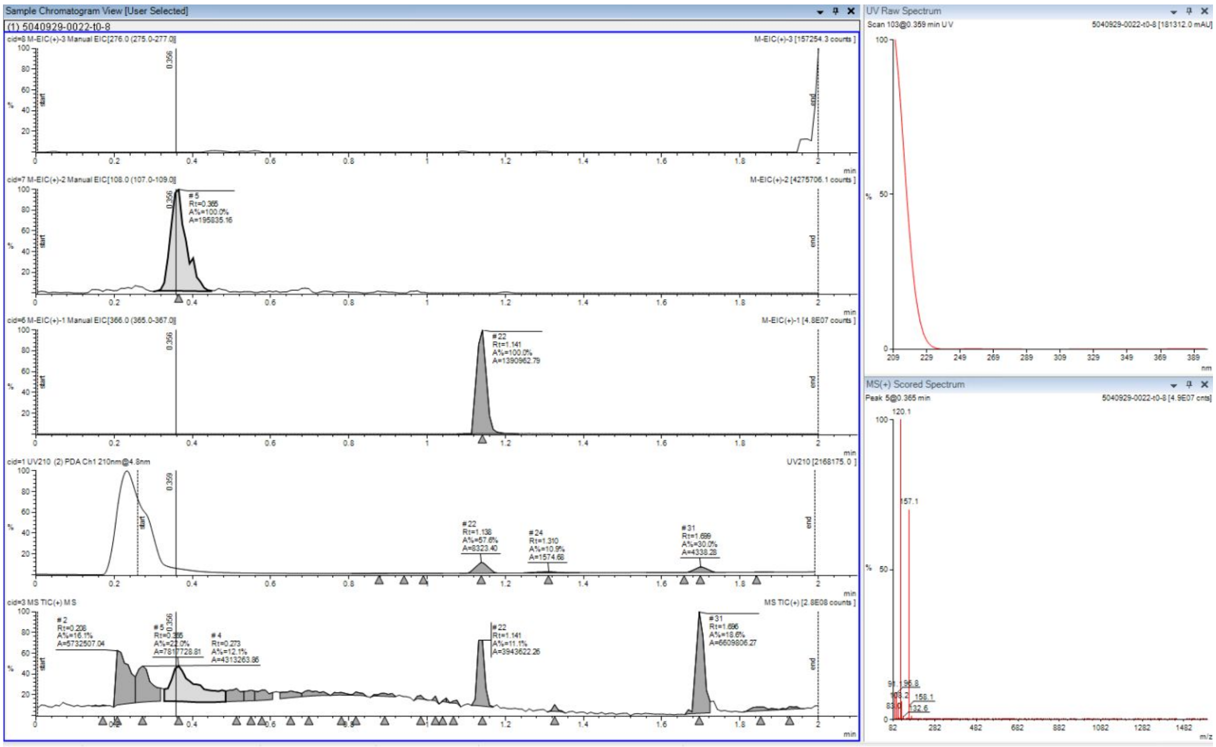

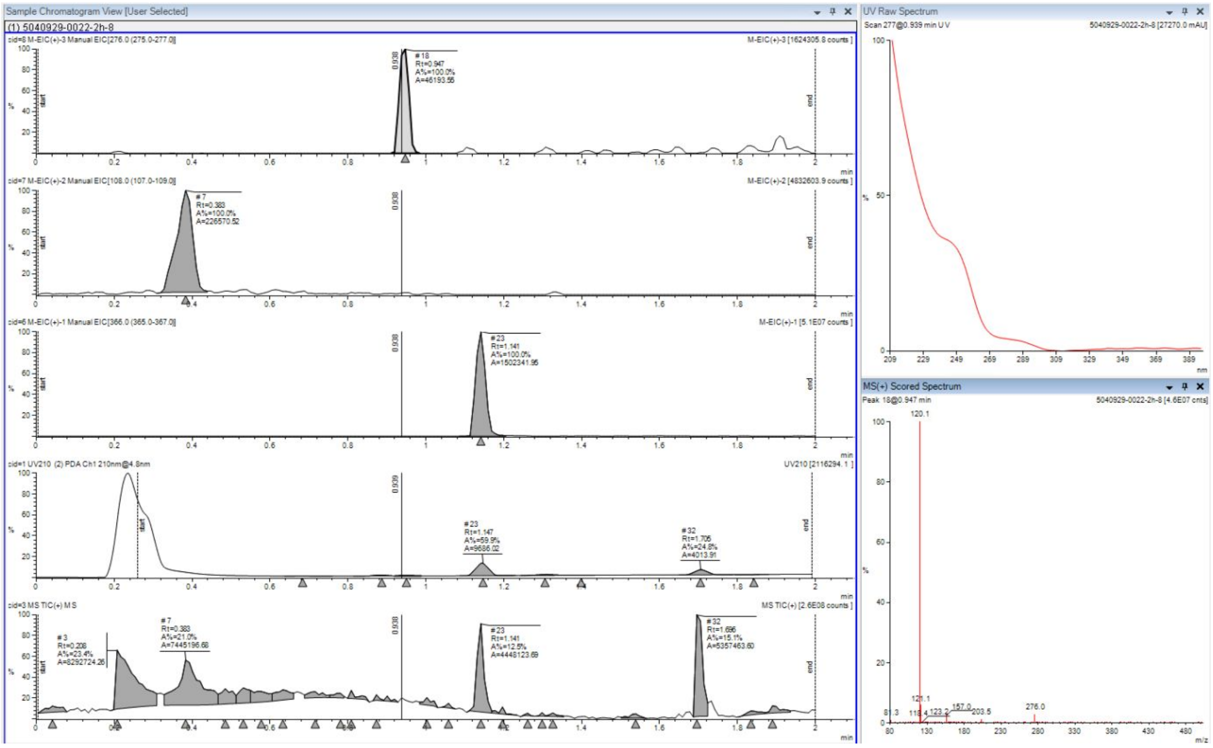

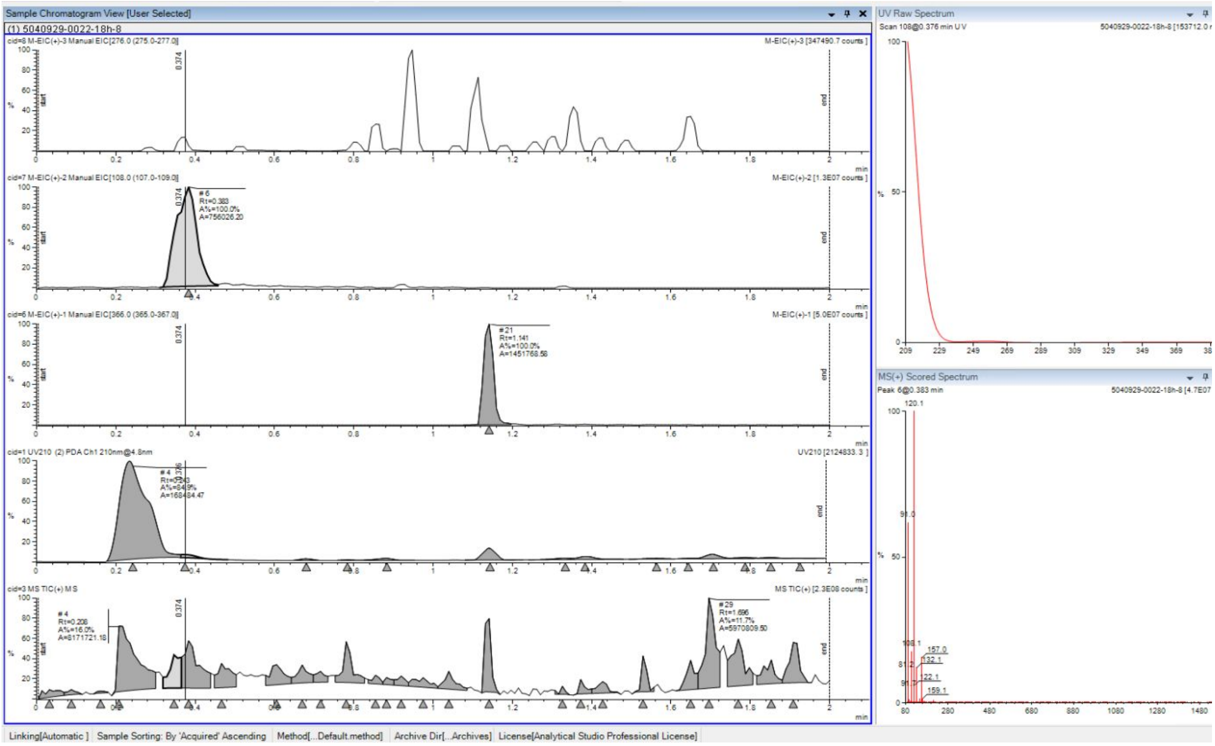

Reaction with 1-benzyl-N-(4-(trifluoromethyl)benzyl)piperidin-4-amine (37)

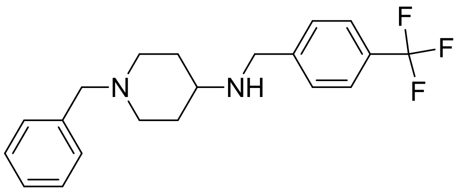

m/z: 348.18 (100.0%), 349.18 (22.4%), 350.19 (2.3%)

37

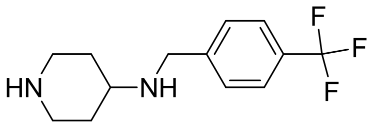

m/z: 258.13 (100.0%), 259.14 (14.3%)

37a

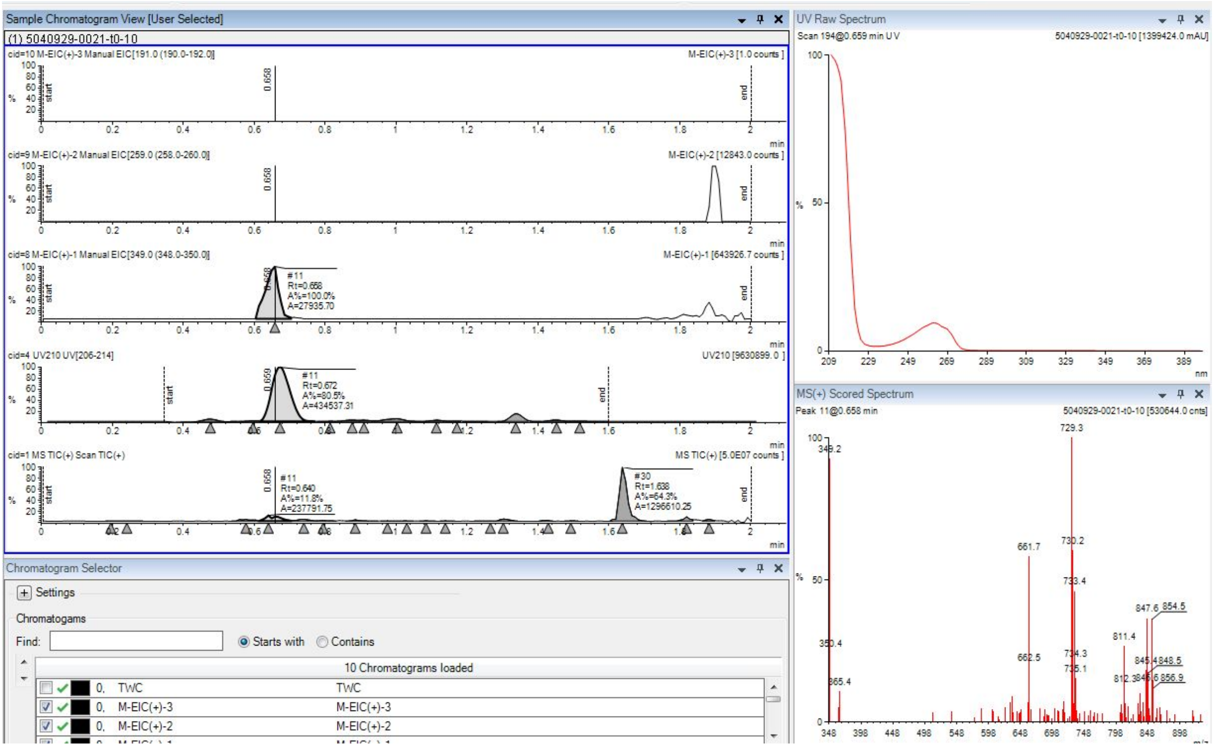

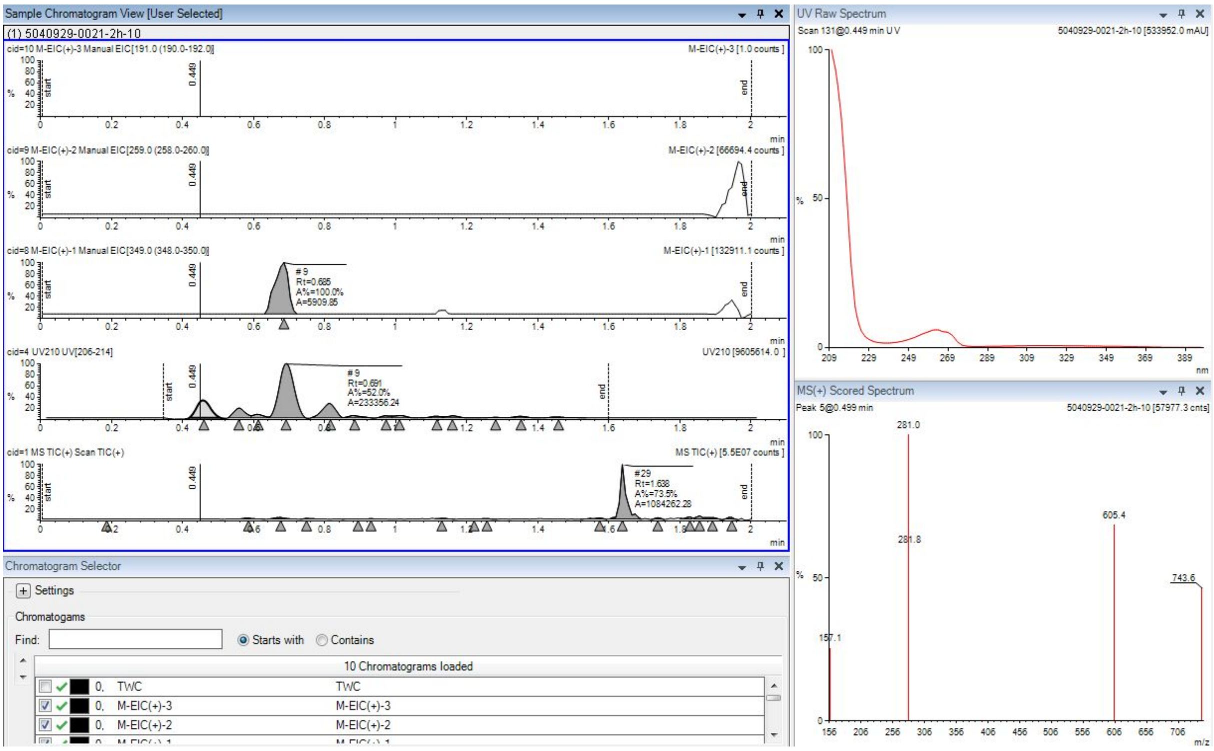
Sample Chromatogram View [User Selected]
(1) 5040929-0021-2h-10
cid=10 M-EIC(+)-3 Manual EIC[191.0 (190.0-192.0)]
M-EIC(+)-3 [1.0 counts]
cid=9 M-EIC(+)-2 Manual EIC[259.0 (258.0-260.0)]
M-EIC(+)-2 [66694.4 counts]
cid=8 M-EIC(+)-1 Manual EIC[349.0 (348.0-350.0)]
M-EIC(+)-1 [132911.1 counts]
#9
Rt=0.685
A%=100.0%
A=5909.85
cid=4 UV210 UV[206-214]
UV210 [9605614.0]
#9
Rt=0.691
A%=52.0%
A=233356.24
cid=1 MS TIC(+) Scan TIC(+)
MS TIC(+) [5.5E07 counts]
#29
Rt=1.638
A%=73.5%
A=1084262.28
UV Raw Spectrum
Scan 131@0.449 min UV
5040929-0021-2h-10 [533952.0 mAU]
MS(+) Scored Spectrum
Peak 5@0.499 min
5040929-0021-2h-10 [57977.3 cnts]
281.0
281.8
605.4
743.6
157.1
Chromatogram Selector
Settings
Chromatogams
Find:
Starts with
Contains
10 Chromatograms loaded
0, TWC
TWC
0, M-EIC(+)-3
M-EIC(+)-3
0, M-EIC(+)-2
M-EIC(+)-2

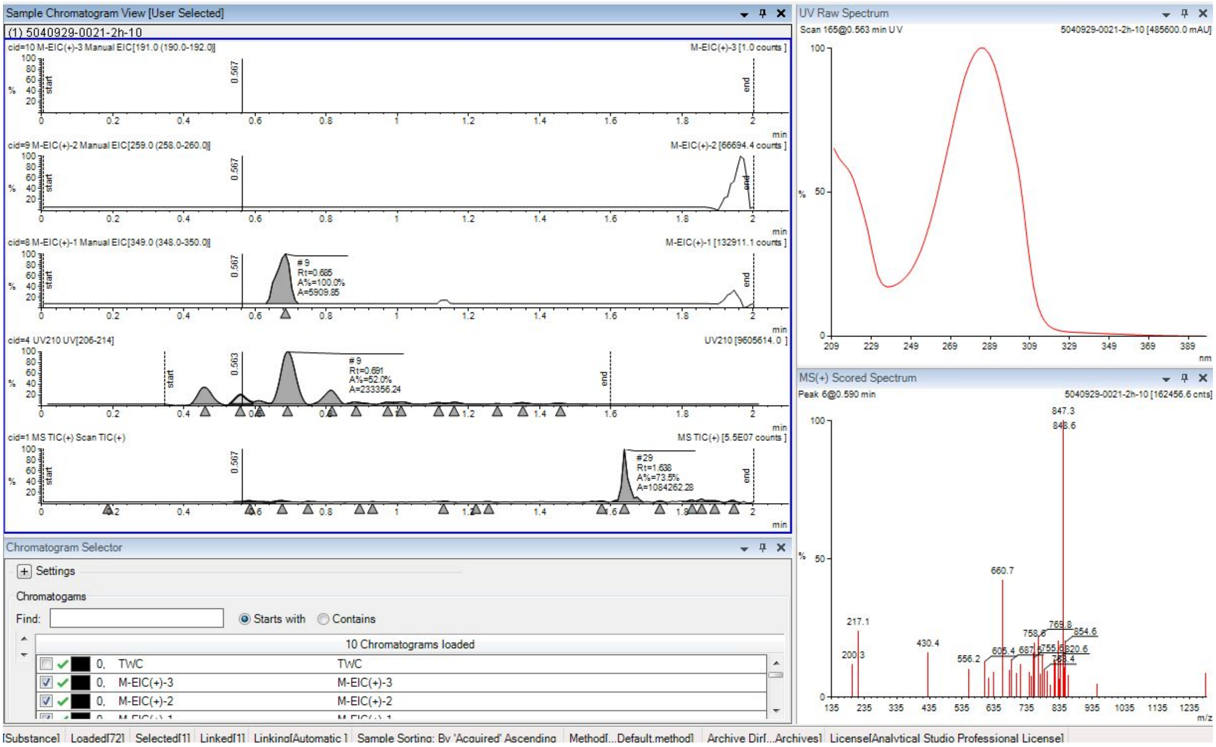
Sample Chromatogram View [User Selected]
(1) 5040929-0021-2h-10
cid=10 M-EIC(+)-3 Manual EIC[191.0 (190.0-192.0)]
M-EIC(+)-3 [1.0 counts]
cid=9 M-EIC(+)-2 Manual EIC[259.0 (258.0-260.0)]
M-EIC(+)-2 [66694.4 counts]
cid=8 M-EIC(+)-1 Manual EIC[349.0 (348.0-350.0)]
M-EIC(+)-1 [132911.1 counts]
#9
Rt=0.685
A%=100.0%
A=5909.85
cid=4 UV210 UV[206-214]
UV210 [9605614.0]
#9
Rt=0.691
A%=52.0%
A=233356.24
cid=1 MS TIC(+) Scan TIC(+)
MS TIC(+) [5.5E07 counts]
#29
Rt=1.638
A%=73.5%
A=1084262.28
UV Raw Spectrum
Scan 165@0.563 min UV
5040929-0021-2h-10 [485600.0 mAU]
MS(+) Scored Spectrum
Peak 6@0.590 min
5040929-0021-2h-10 [162456.6 cnts]
847.3
848.6
660.7
217.1
200.3
430.4
556.2
758.6
769.8
854.6
820.6
Chromatogram Selector
Settings
Chromatogams
Find:
Starts with
Contains
10 Chromatograms loaded
0, TWC
TWC
0, M-EIC(+)-3
M-EIC(+)-3
0, M-EIC(+)-2
M-EIC(+)-2
[Substance] Loaded[72] Selected[1] Linked[1] Linking[Automatic] Sample Sorting: By 'Acquired' Ascending Method[...Default.method] Archive Dir[...Archives] License[Analytical Studio Professional License]

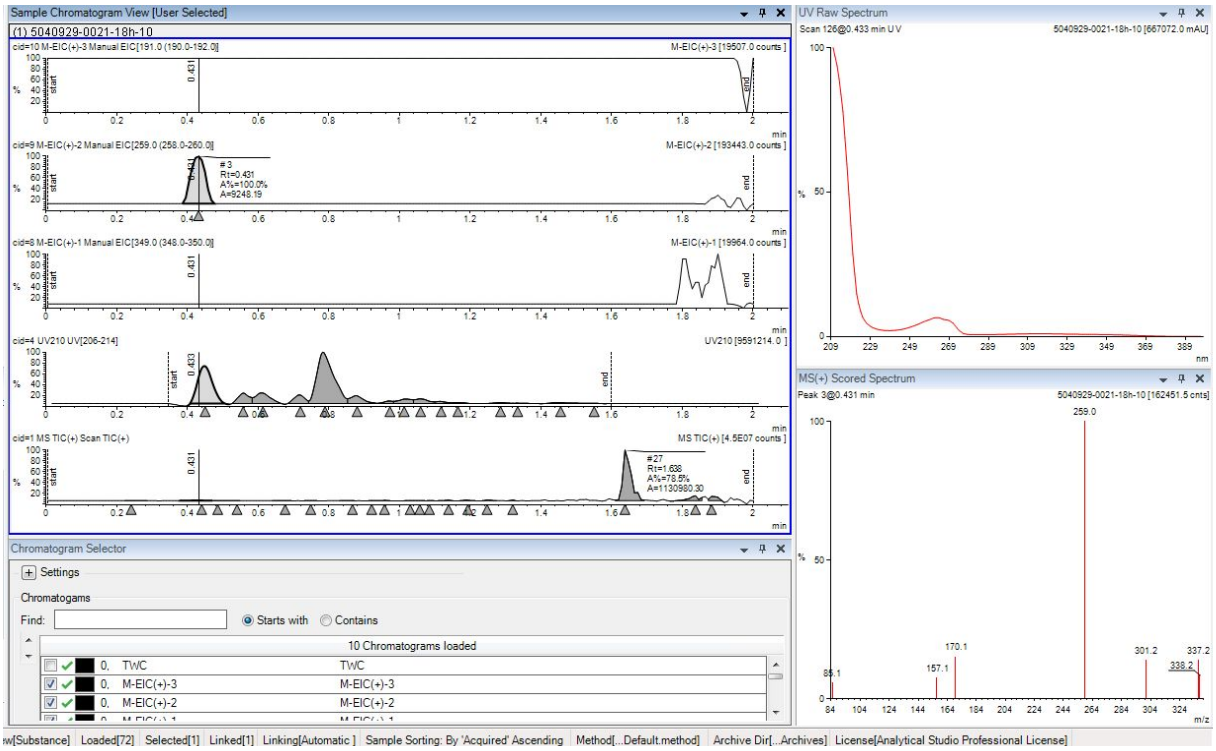

Sample Chromatogram View [User Selected]
(1) 5040929-0021-18h-10
cid=10 M-EIC(+)-3 Manual EIC[191.0 (190.0-192.0)]
M-EIC(+)-3 [19507.0 counts]
cid=9 M-EIC(+)-2 Manual EIC[259.0 (258.0-260.0)]
M-EIC(+)-2 [193443.0 counts]
#3
Rt=0.431
A%=100.0%
A=9248.19
cid=8 M-EIC(+)-1 Manual EIC[349.0 (348.0-350.0)]
M-EIC(+)-1 [19964.0 counts]
cid=4 UV210 UV[206-214]
UV210 [9591214.0]
cid=1 MS TIC(+) Scan TIC(+)
MS TIC(+) [4.5E07 counts]
#27
Rt=1.638
A%=78.5%
A=1130980.30
UV Raw Spectrum
Scan 126@0.433 min UV
5040929-0021-18h-10 [667072.0 mAU]
MS(+) Scored Spectrum
Peak 3@0.431 min
5040929-0021-18h-10 [162451.5 cnts]
259.0
170.1
157.1
301.2
337.2
338.2
Chromatogram Selector
Settings
Chromatogams
Find:
Starts with
Contains
10 Chromatograms loaded
0, TWC TWC
0, M-EIC(+)-3 M-EIC(+)-3
0, M-EIC(+)-2 M-EIC(+)-2
w[Substance] Loaded[72] Selected[1] Linked[1] Linking[Automatic] Sample Sorting: By 'Acquired' Ascending Method[...Default.method] Archive Dir[...Archives] License[Analytical Studio Professional License]

Reaction with *N*-phenylpyrrolidine-2-carboxamide (38)

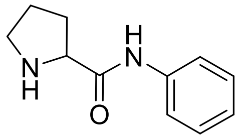

38

m/z: 190.11 (100.0%), 191.11 (12.7%)

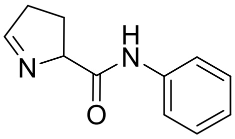

38a

m/z: 188.09 (100.0%), 189.10 (12.1%)

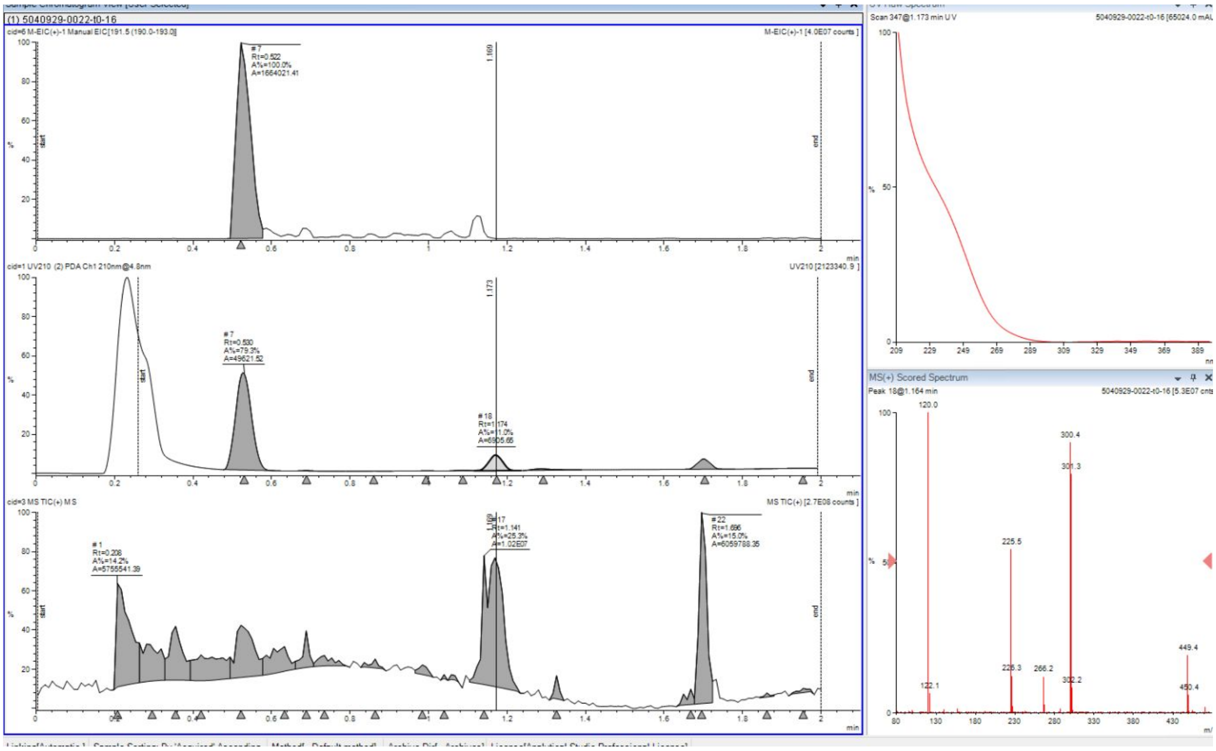

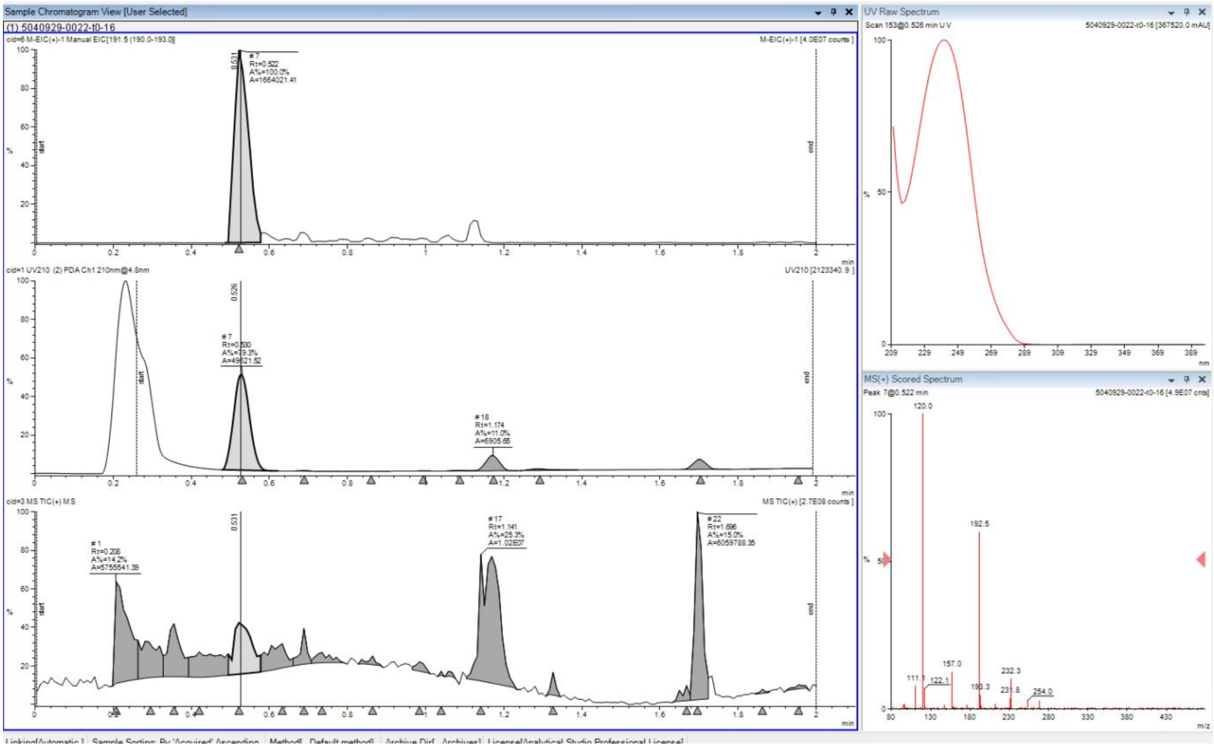

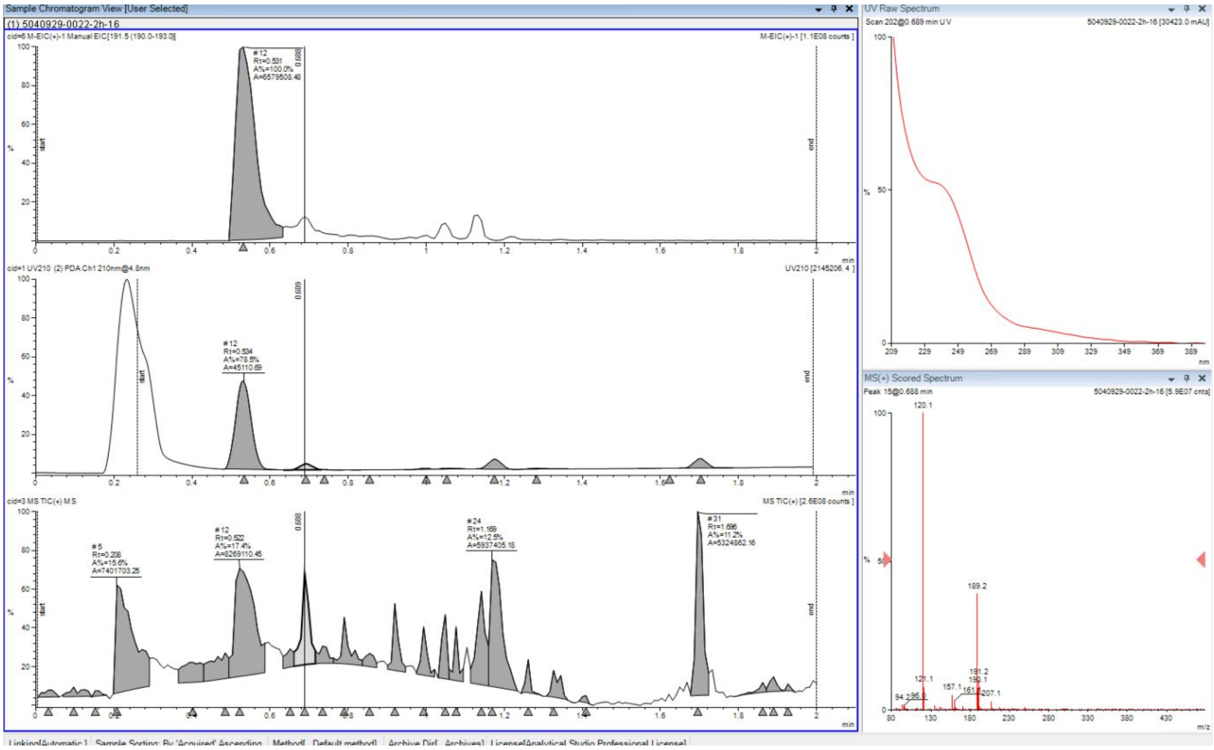

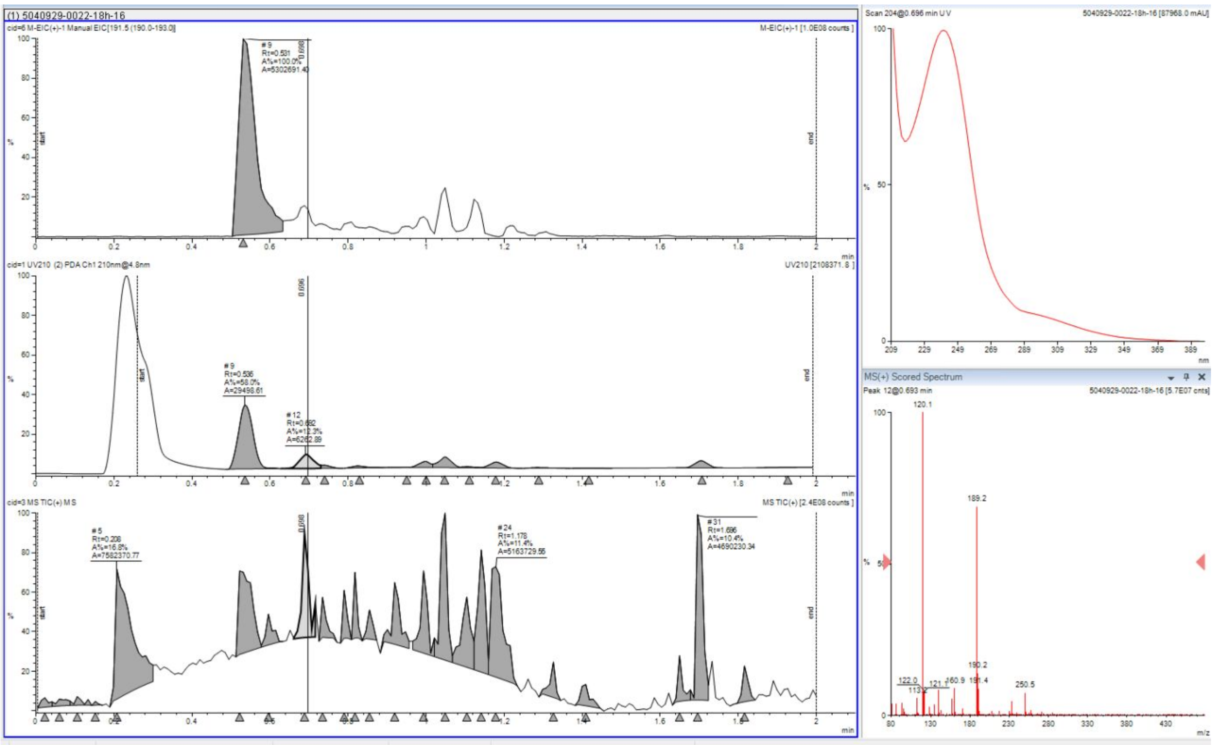

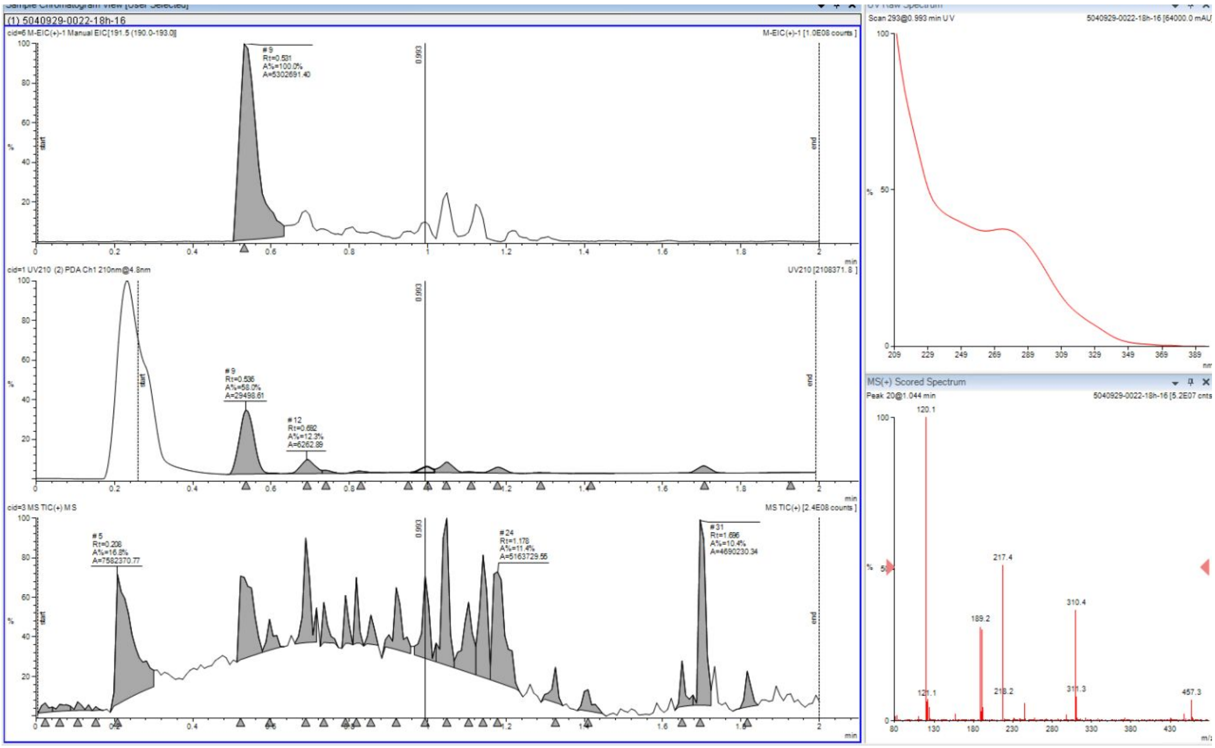

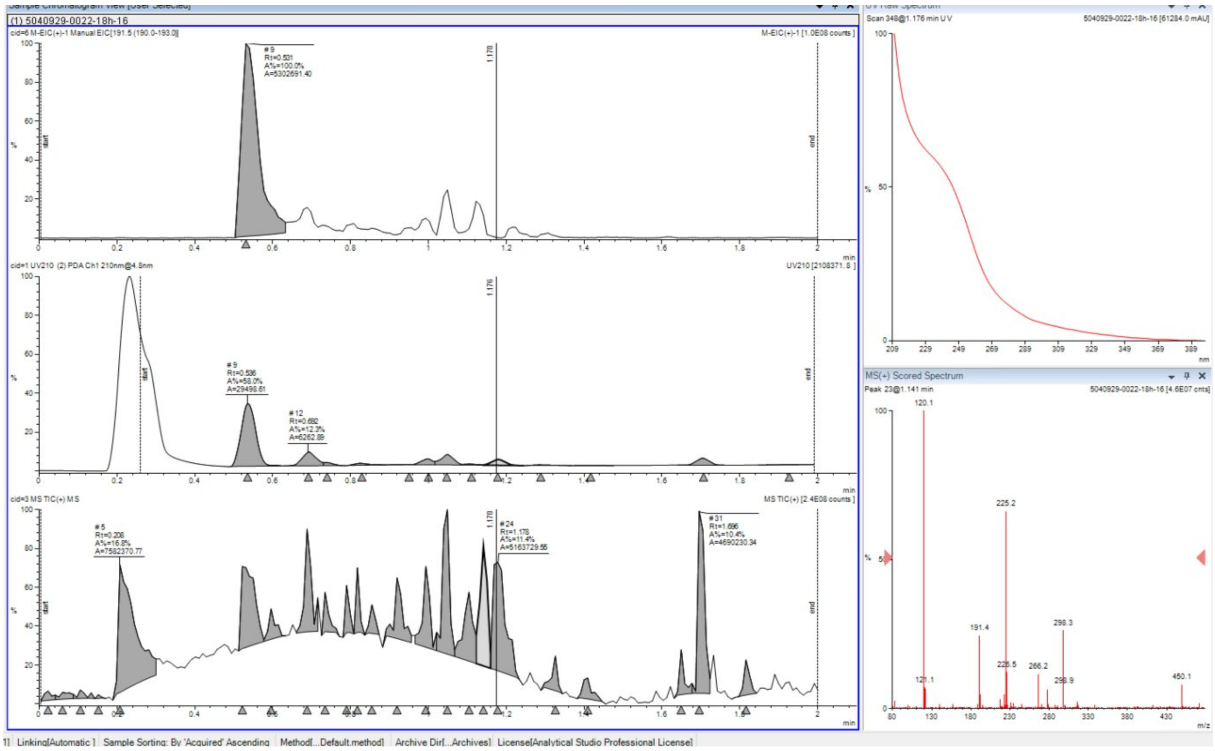

Reaction with 3-bromo-5-(piperidin-2-yl)pyridine (39)

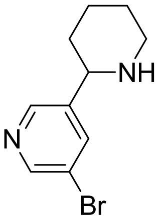

39

m/z: 240.03 (100.0%), 242.02 (97.3%), 241.03 (11.0%), 243.03 (10.7%)

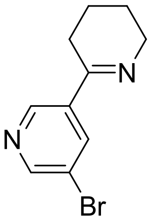

39a

m/z: 238.01 (100.0%), 240.01 (97.4%), 239.01 (11.6%), 241.01 (11.4%)

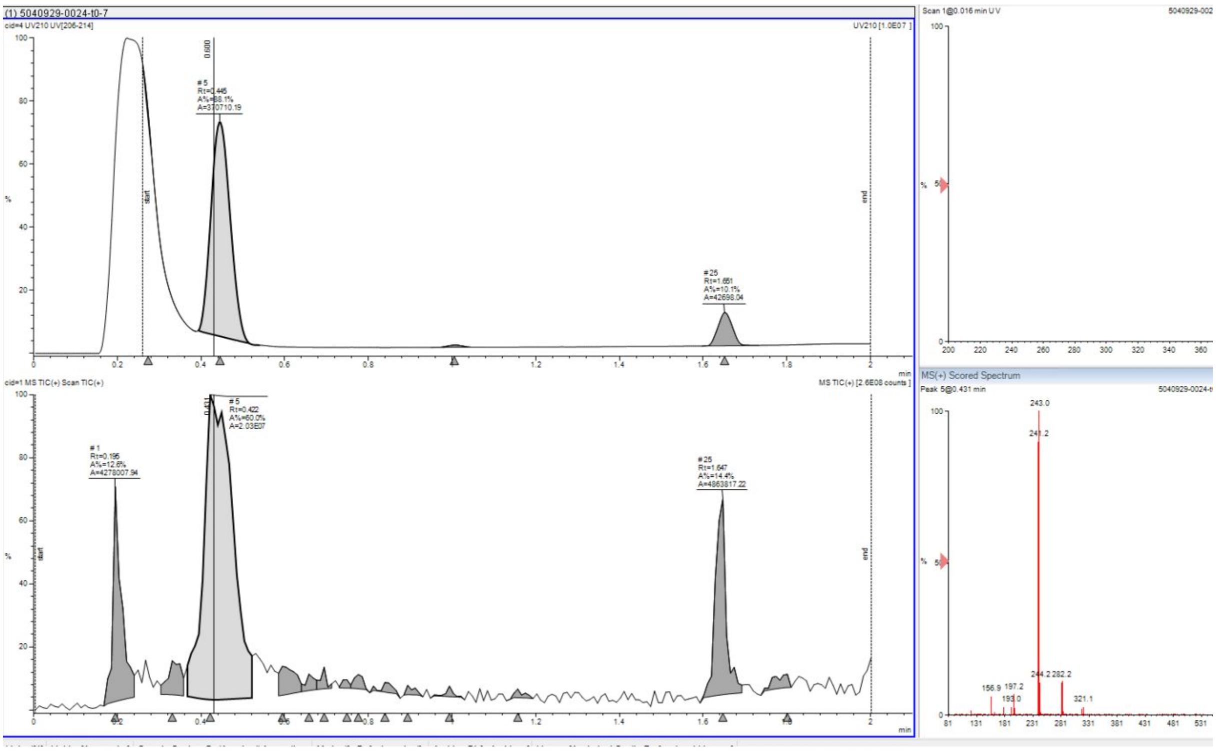

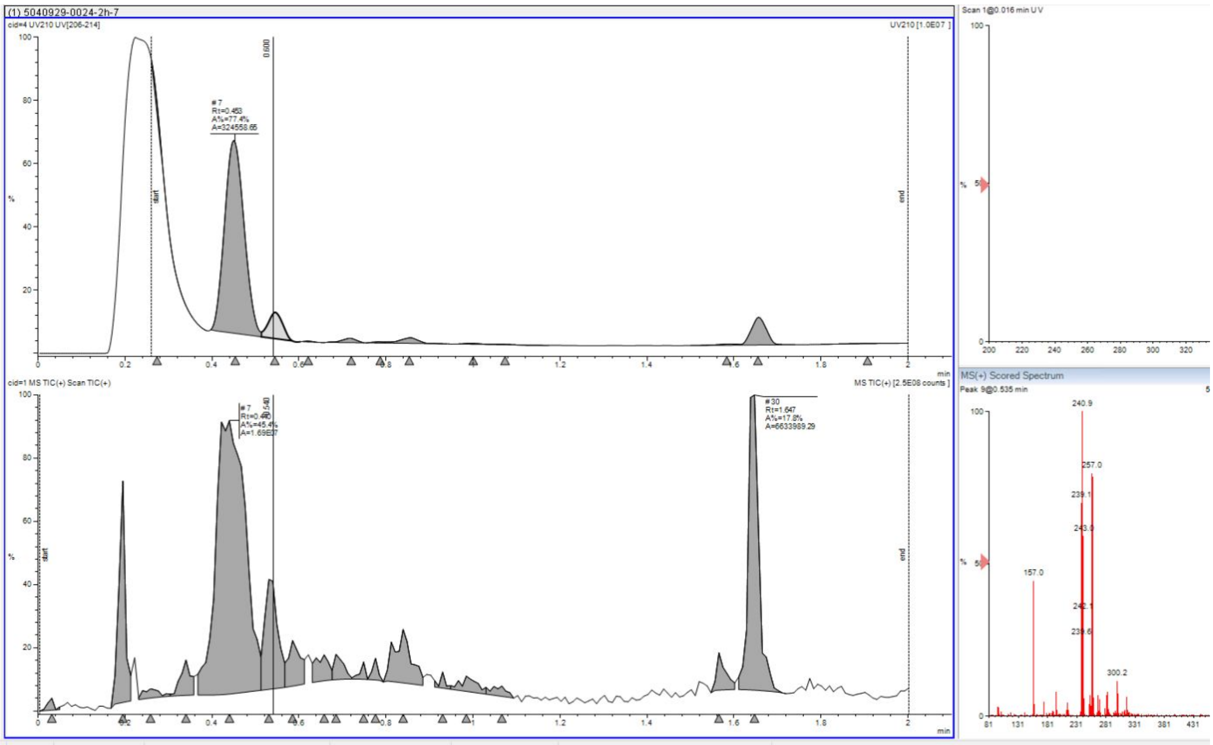

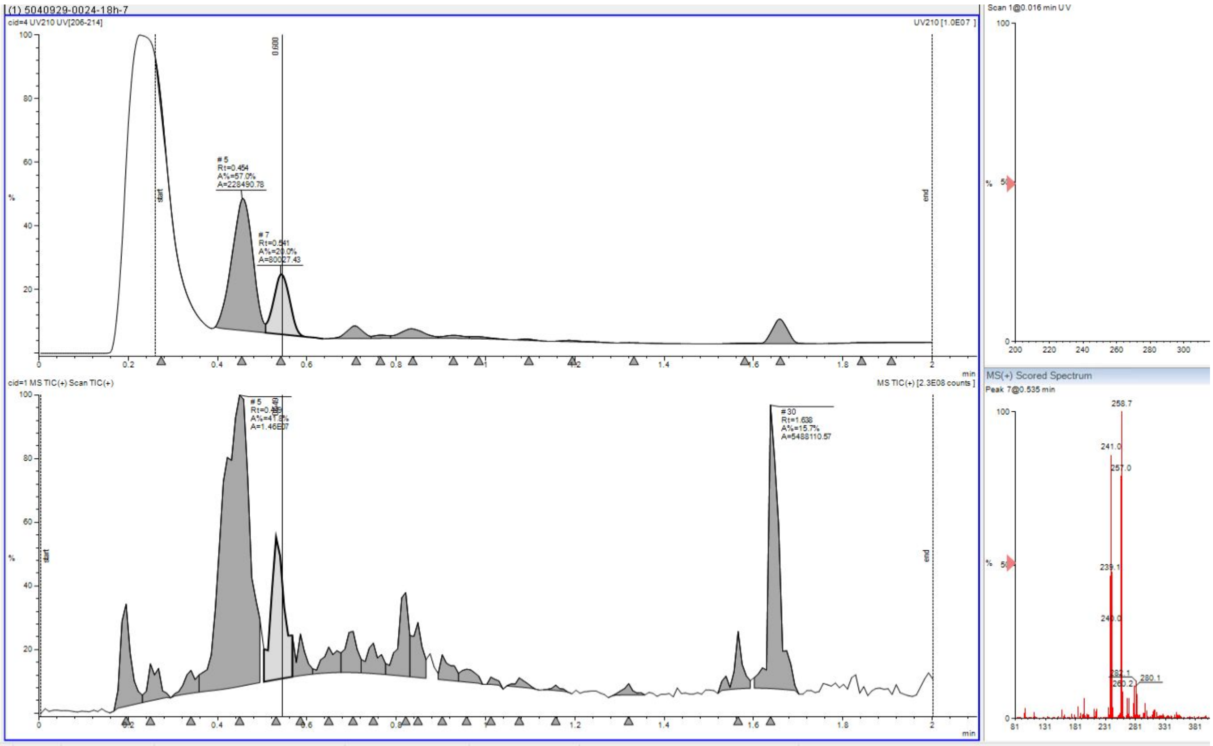

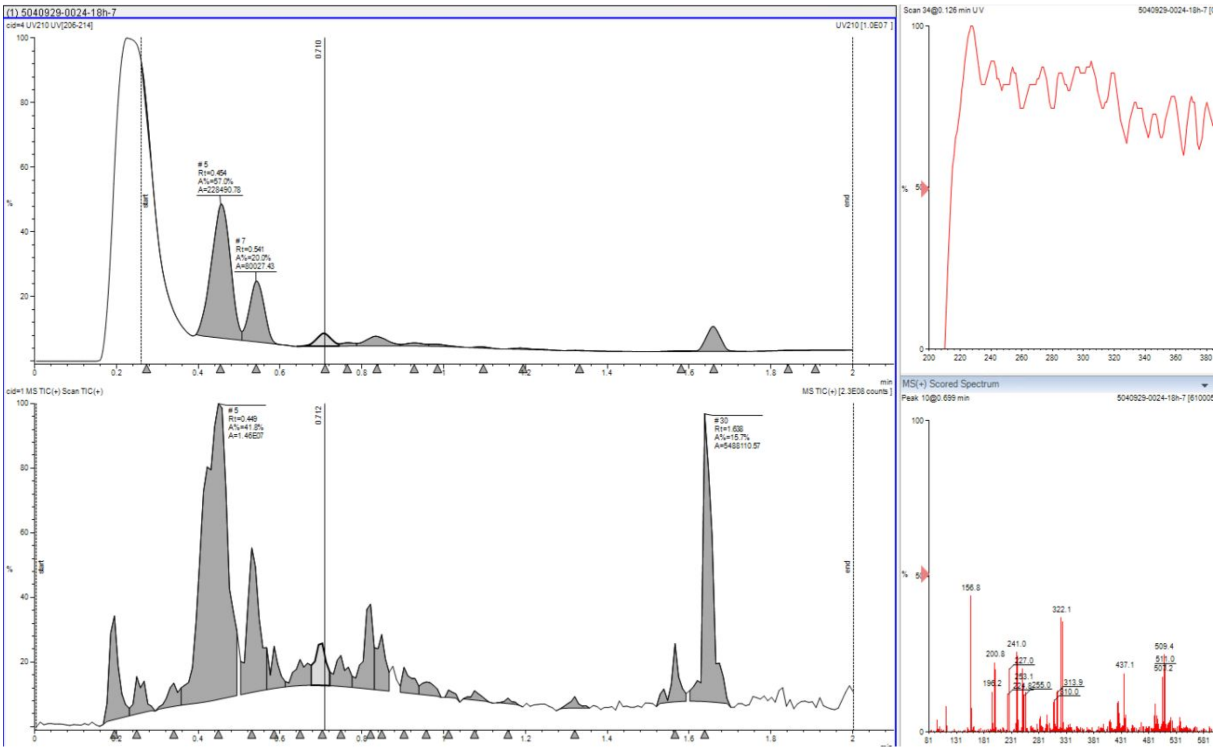
(1) 5040929-0024-18h-7
cid=4 UV210 UV[206-214]
UV210 [1.0E07]
# 5
Rt=0.454
A%=57.0%
A=228490.78
# 7
Rt=0.541
A%=20.0%
A=80027.43
cid=1 MS TIC(+) Scan TIC(+)
MS TIC(+) [2.3E08 counts]
# 5
Rt=0.449
A%=41.8%
A=1.46E07
# 30
Rt=1.638
A%=15.7%
A=5488110.57
MS(+) Scored Spectrum
Peak 10@0.699 min
156.8
322.1
241.0
200.8
509.4
437.1

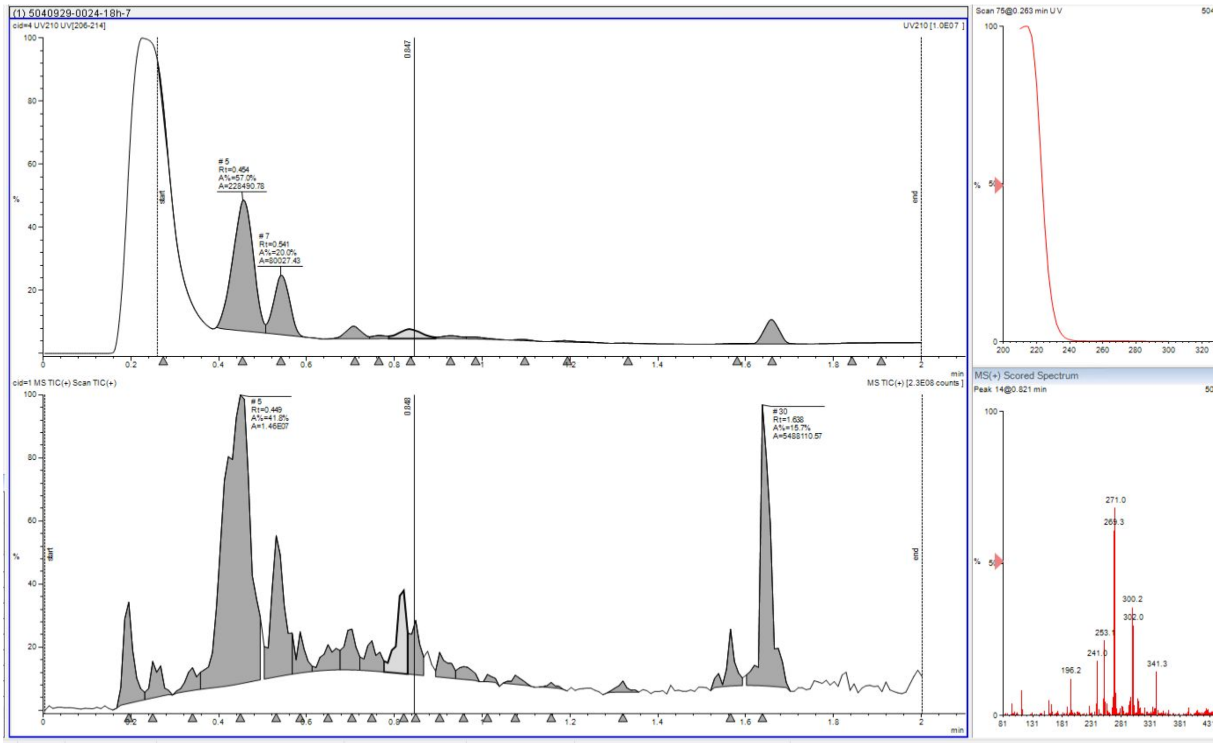
(1) 5040929-0024-18h-7
cid=4 UV210 UV[206-214]
UV210 [1.0E07]
# 5
Rt=0.454
A%=57.0%
A=228490.78
# 7
Rt=0.541
A%=20.0%
A=80027.43
cid=1 MS TIC(+) Scan TIC(+)
MS TIC(+) [2.3E08 counts]
# 5
Rt=0.449
A%=41.8%
A=1.46E07
# 30
Rt=1.638
A%=15.7%
A=5488110.57
MS(+) Scored Spectrum
Peak 14@0.821 min
271.0
300.2
302.0
196.2
341.3

Reaction with (S)-2-(diphenyl((trimethylsilyl)oxy)methyl)pyrrolidine (40)

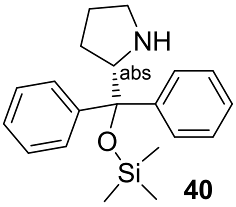

40

m/z: 325.19 (100.0%), 326.19 (27.1%), 327.19 (3.6%), 327.18 (3.4%)

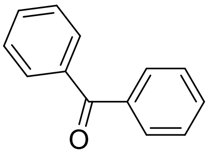

40a

m/z: 182.07 (100.0%), 183.08 (14.2%), 184.08 (1.1%)

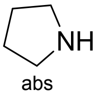

40b

m/z: 71.07 (100.0%), 72.08 (4.4%)

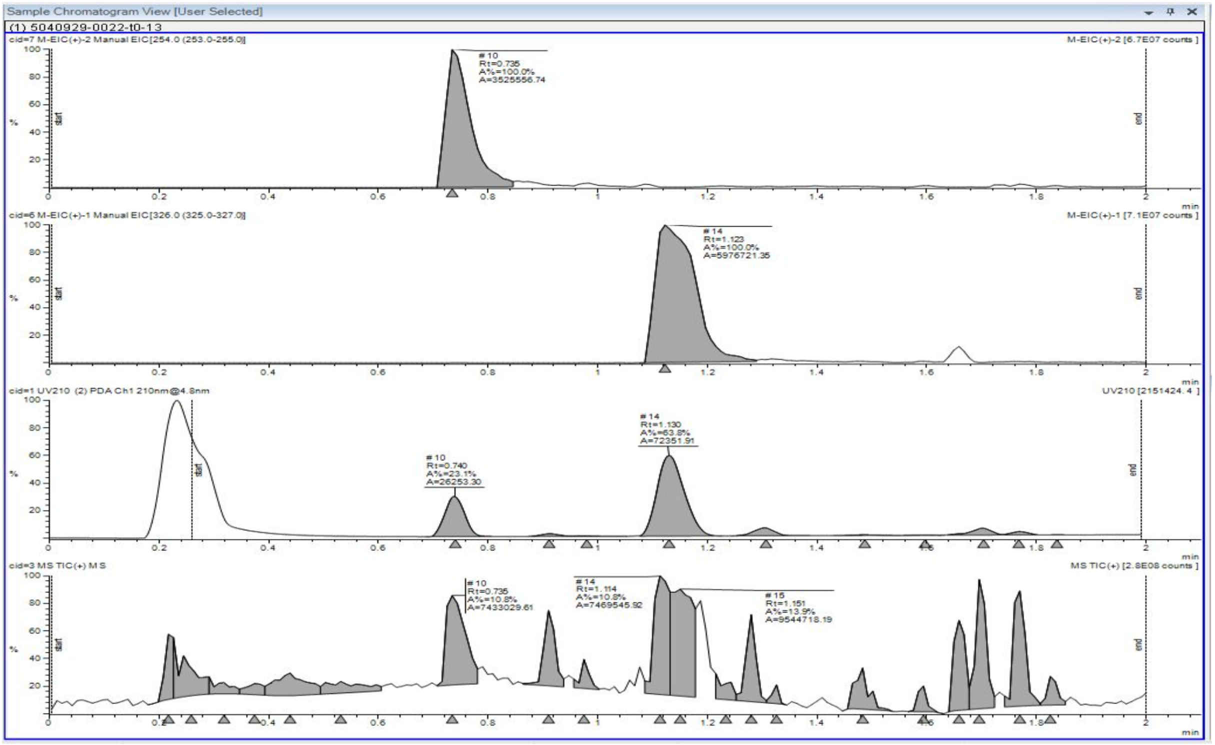

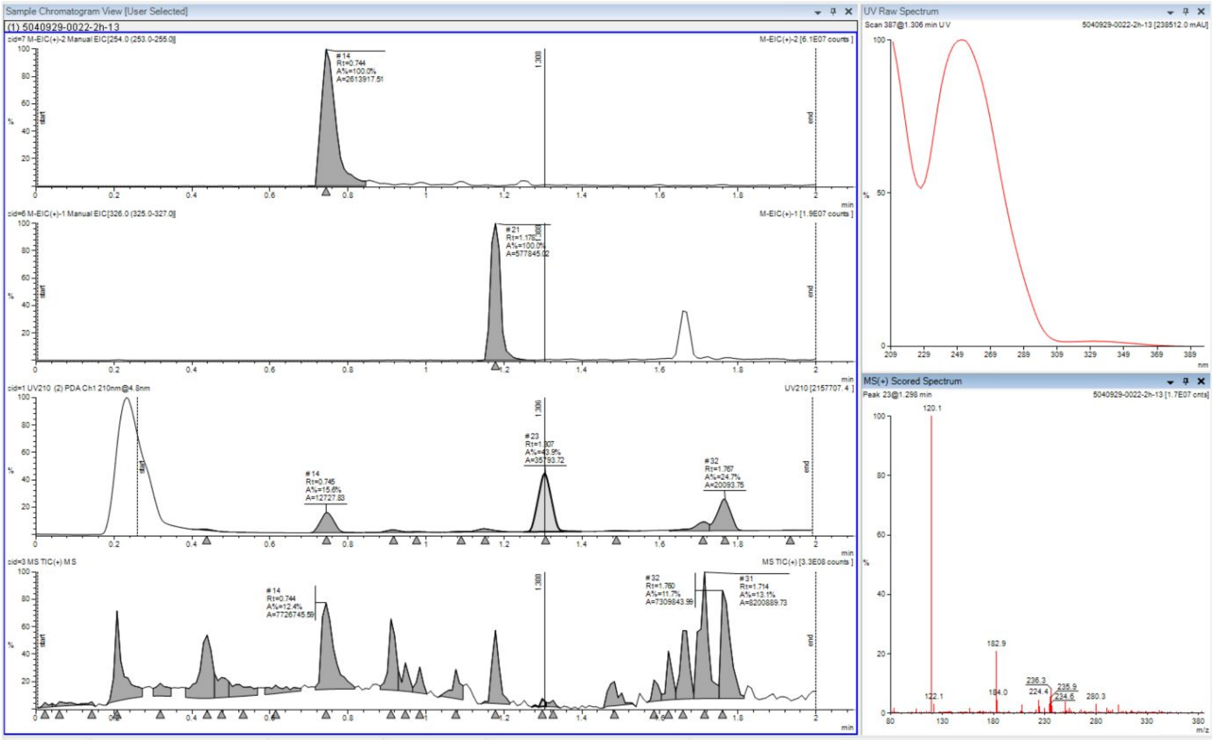
Sample Chromatogram View [User Selected]
(1) 5040929-0022-2h-13
UV Raw Spectrum
MS(+) Scored Spectrum

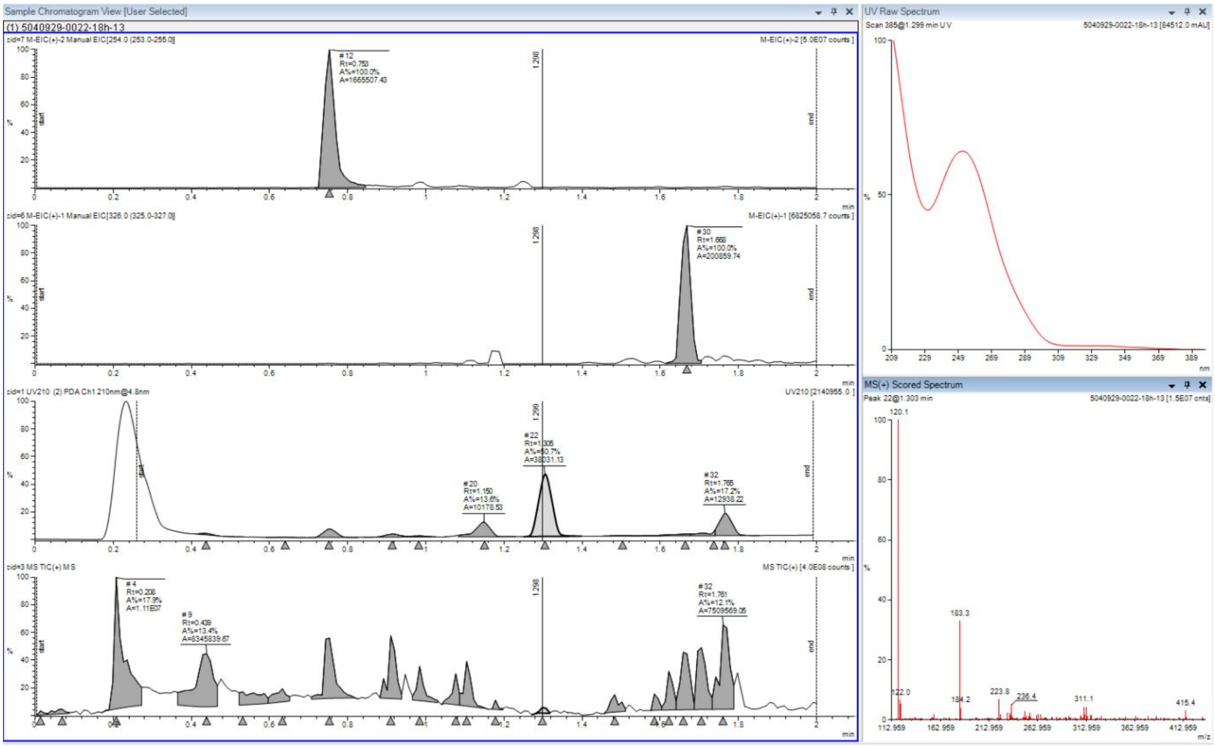
Sample Chromatogram View [User Selected]
(1) 5040929-0022-18h-13
UV Raw Spectrum
MS(+) Scored Spectrum

Reaction with 2,3,4,9-tetrahydro-1H-pyrido[3,4-b]indole-3-carboxylic acid (41)

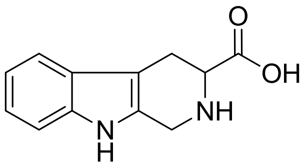

41
m/z: 216.09 (100.0%), 217.09 (13.8%)

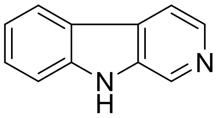

41a
m/z: 168.07 (100.0%), 169.07 (12.6%)

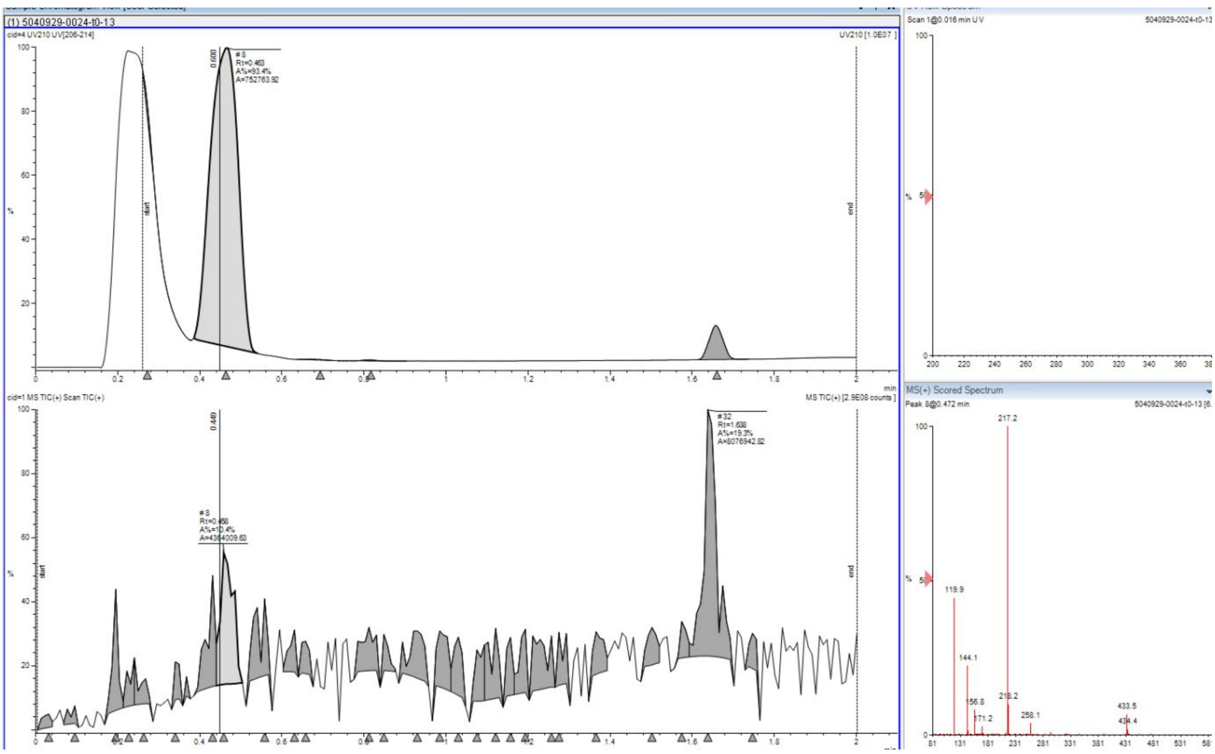

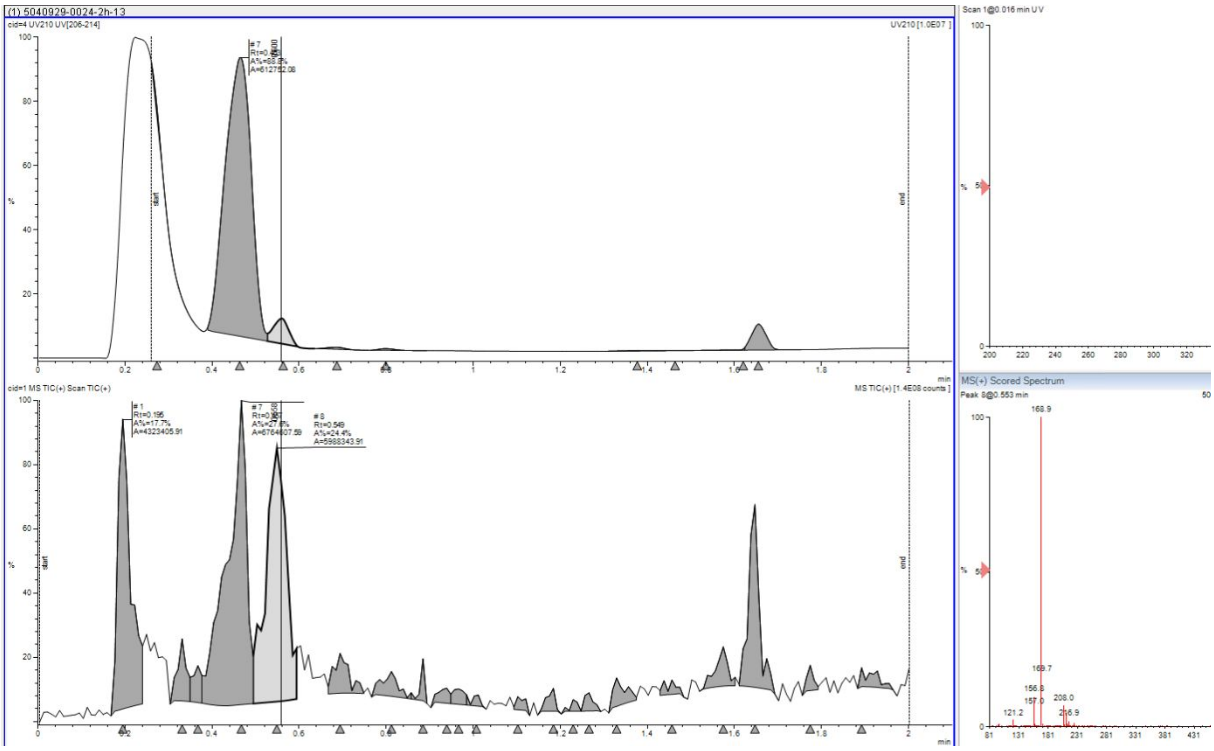
(1) 5040929-0024-2h-13
cid=4 UV210 UV[206-214]
UV210 [1.0E07]
Scan 1@0.016 min UV
cid=1 MS TIC(+) Scan TIC(+)
MS TIC(+) [1.4E08 counts]
# 1
Rt=0.195
A%=17.7%
A=4323405.91
# 8
Rt=0.549
A%=24.4%
A=5988343.91
MS(+) Scored Spectrum
Peak 8@0.553 min
168.9

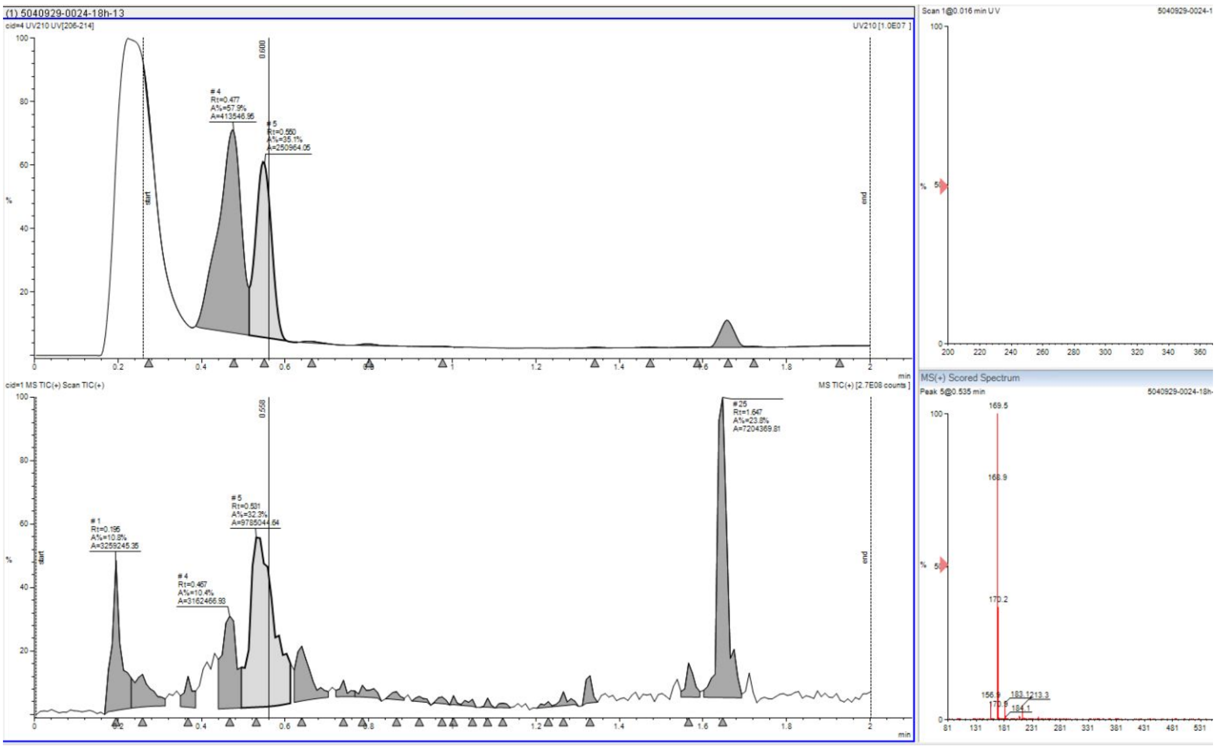
(1) 5040929-0024-18h-13
cid=4 UV210 UV[206-214]
UV210 [1.0E07]
# 4
Rt=0.477
A%=57.9%
A=413546.95
# 5
Rt=0.550
A%=35.1%
A=250964.05
Scan 1@0.016 min UV
cid=1 MS TIC(+) Scan TIC(+)
MS TIC(+) [2.7E08 counts]
# 1
Rt=0.195
A%=10.8%
A=3259245.35
# 4
Rt=0.467
A%=10.4%
A=3162466.93
# 5
Rt=0.531
A%=32.3%
A=9785044.64
# 25
Rt=1.647
A%=23.8%
A=7204369.81
MS(+) Scored Spectrum
Peak 5@0.535 min
169.5

Reaction with 2-(diethylamino)ethyl 4-amino-3-butoxybenzoate (86)

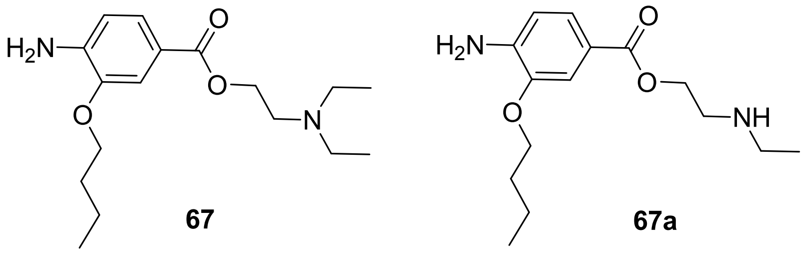

67 **67a**

m/z: 308.21 (100.0%), 309.21 (19.2%), 310.22 (1.7%)

m/z: 280.18 (100.0%), 281.18 (17.4%), 282.19 (1.3%)

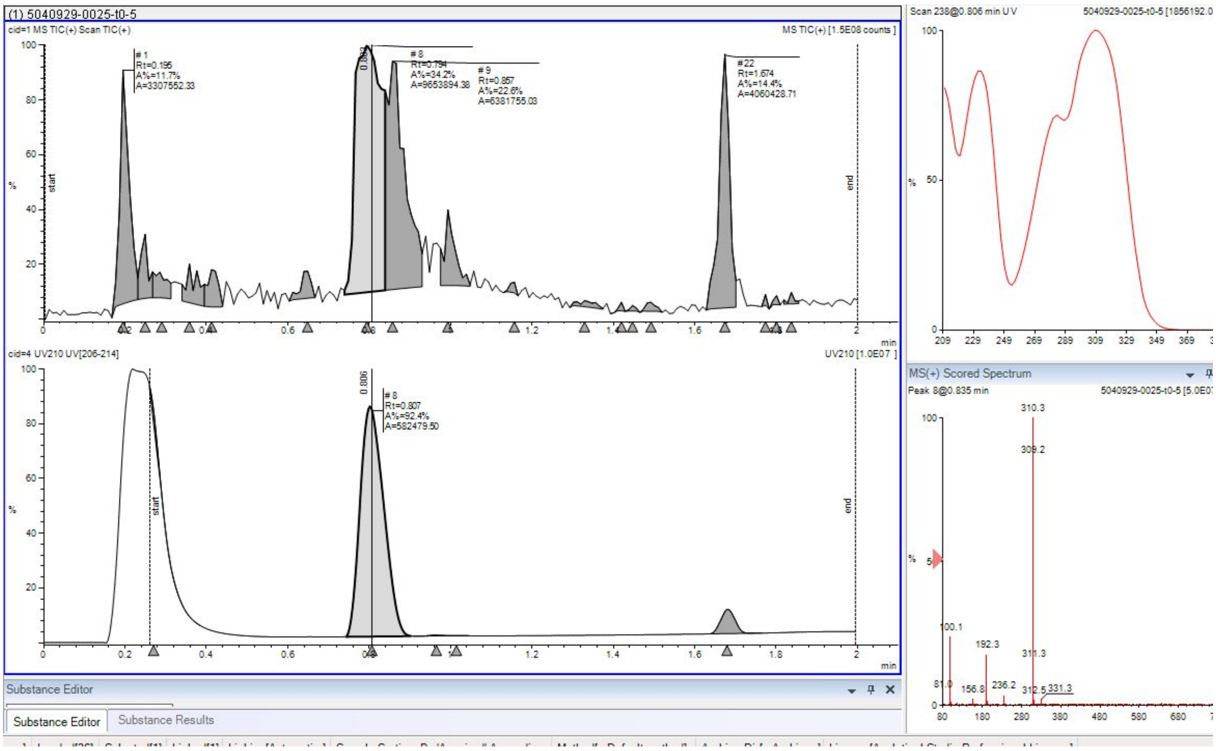

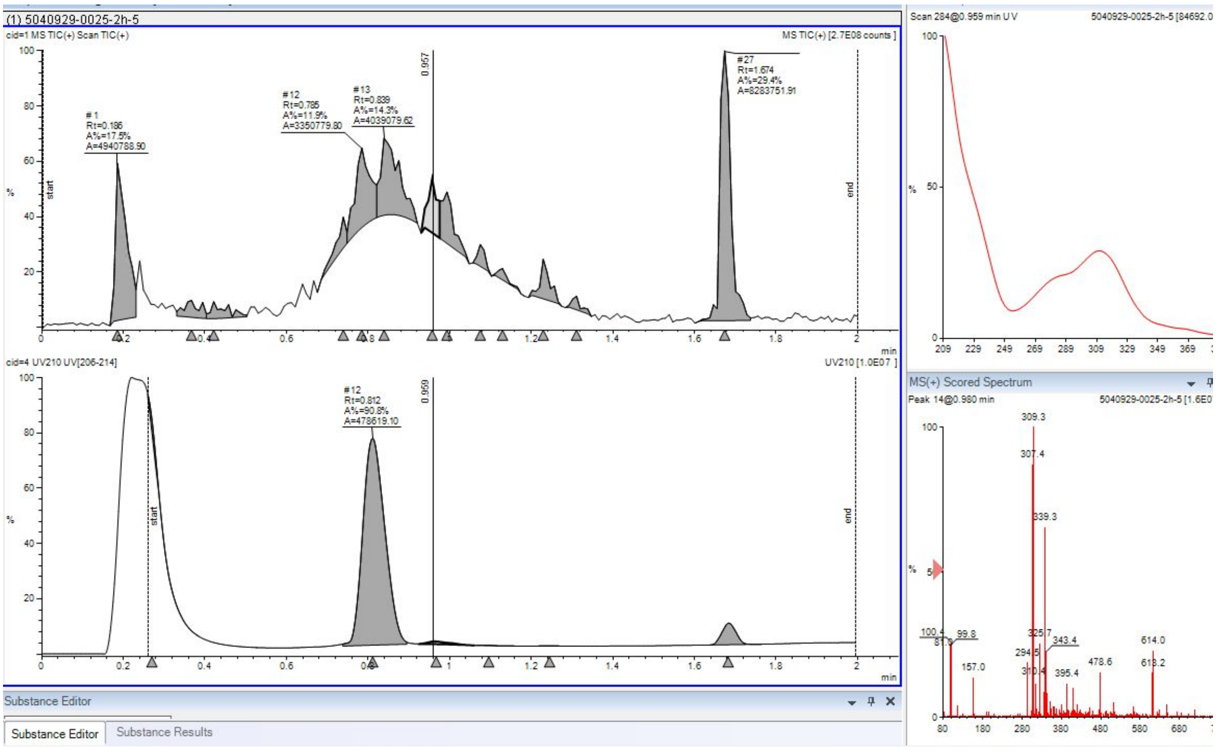
(1) 5040929-0025-2h-5
cid=1 MS TIC(+) Scan TIC(+)
MS TIC(+) [2.7E08 counts]
cid=4 UV210 UV[206-214]
UV210 [1.0E07]
Scan 284@0.959 min UV
MS(+) Scored Spectrum
Peak 14@0.980 min
Substance Editor
Substance Results

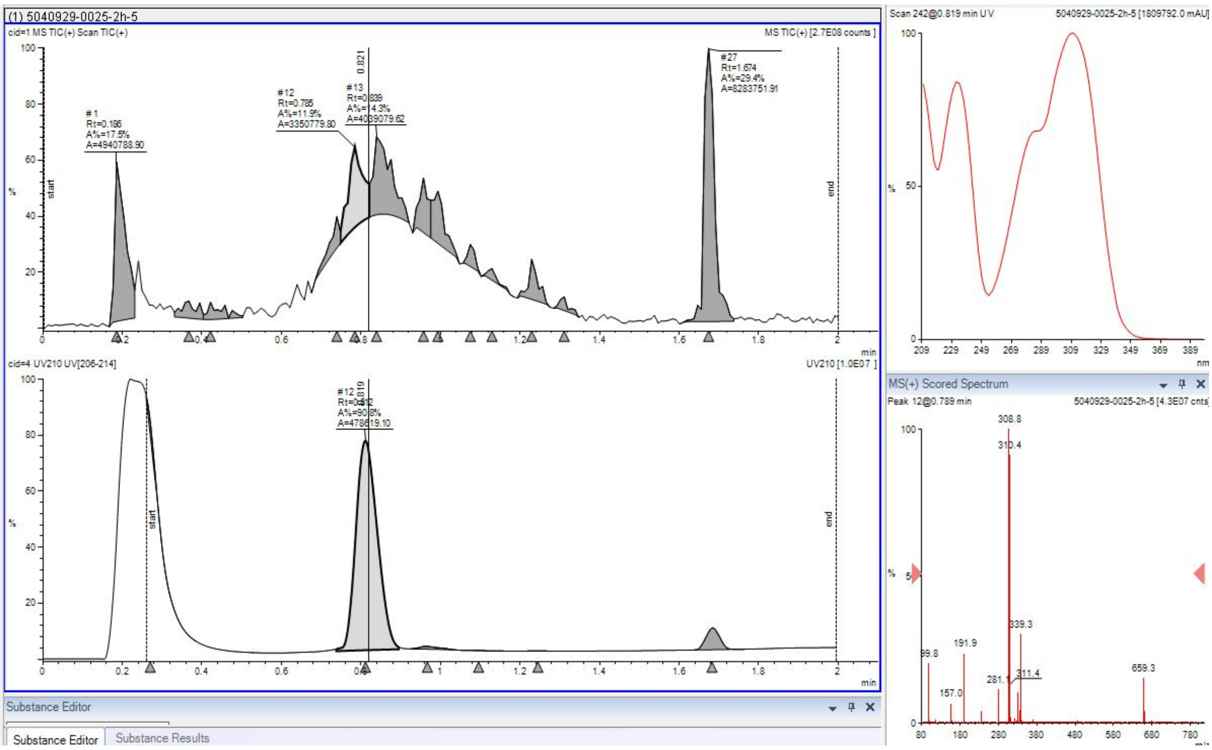
(1) 5040929-0025-2h-5
cid=1 MS TIC(+) Scan TIC(+)
MS TIC(+) [2.7E08 counts]
cid=4 UV210 UV[206-214]
UV210 [1.0E07]
Scan 242@0.819 min UV
5040929-0025-2h-5 [1809792.0 mAU]
MS(+) Scored Spectrum
Peak 12@0.789 min
5040929-0025-2h-5 [4.3E07 cnts]
Substance Editor
Substance Results

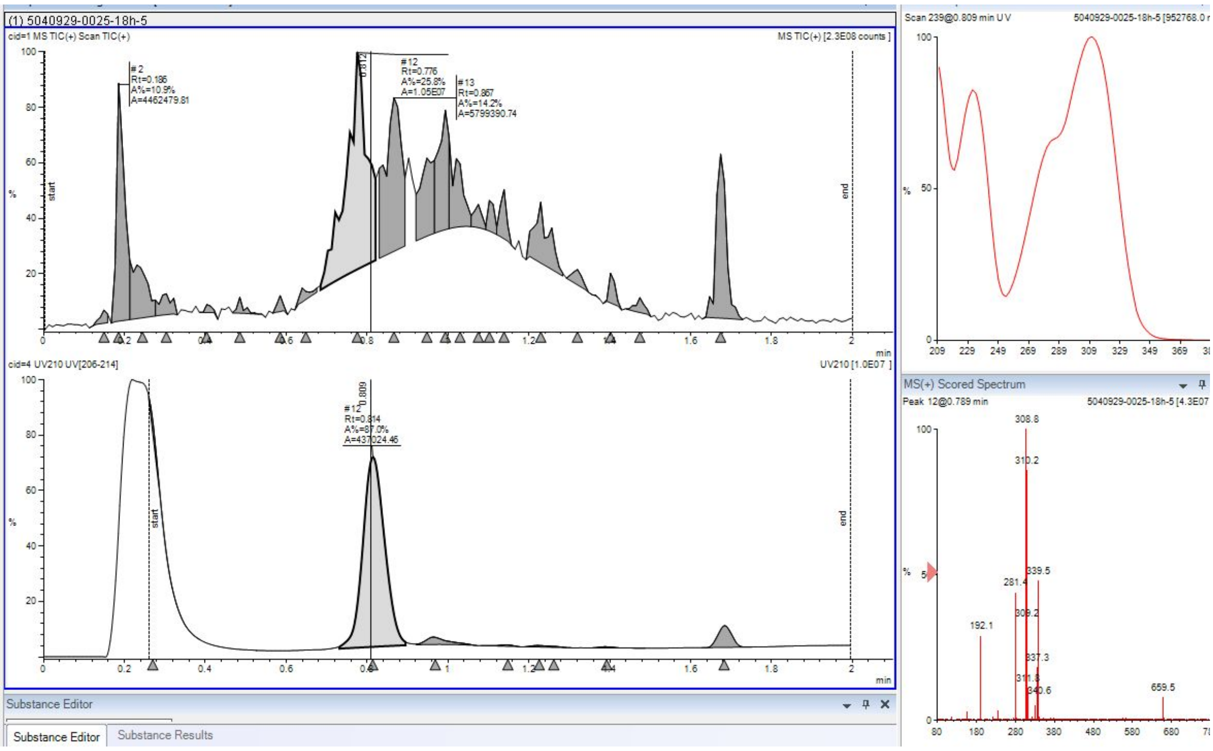

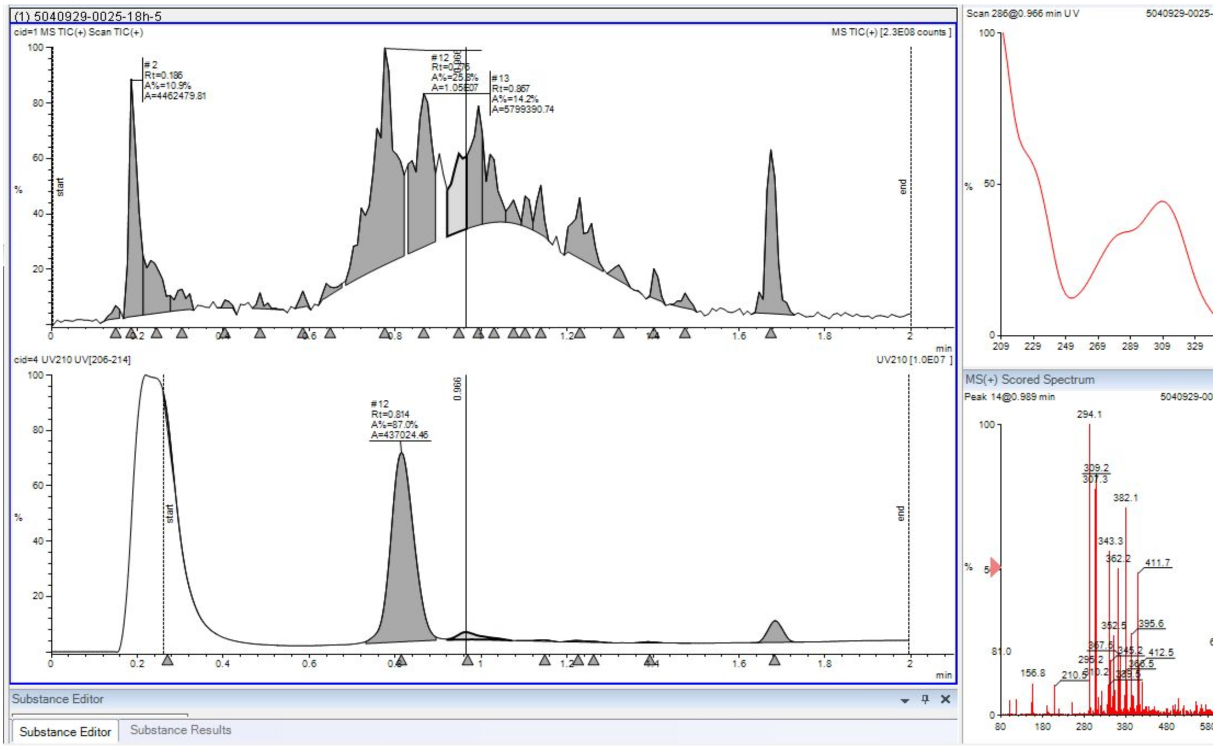

GC-MS and NMR results for mechanistic studies

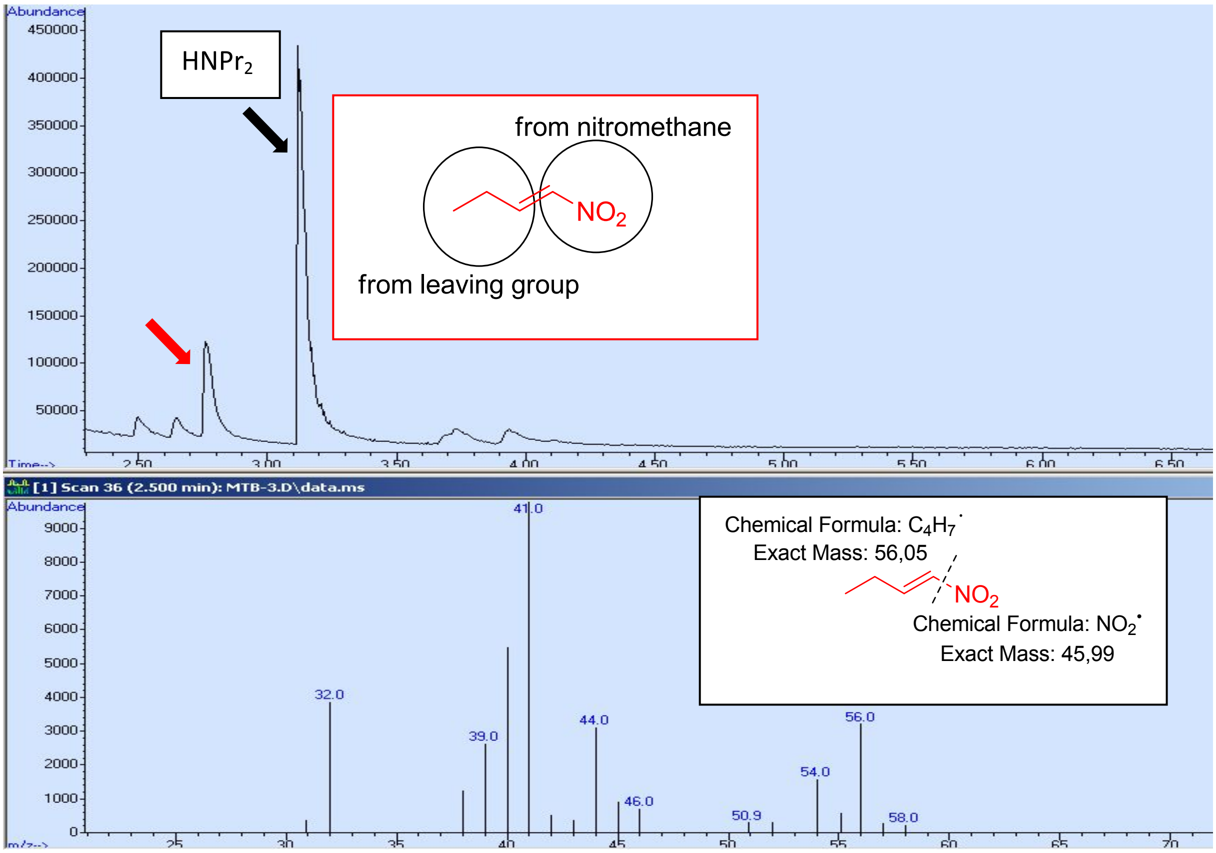

*Figure S25. GC-MS result for reaction with tripropylamine (**3**).*

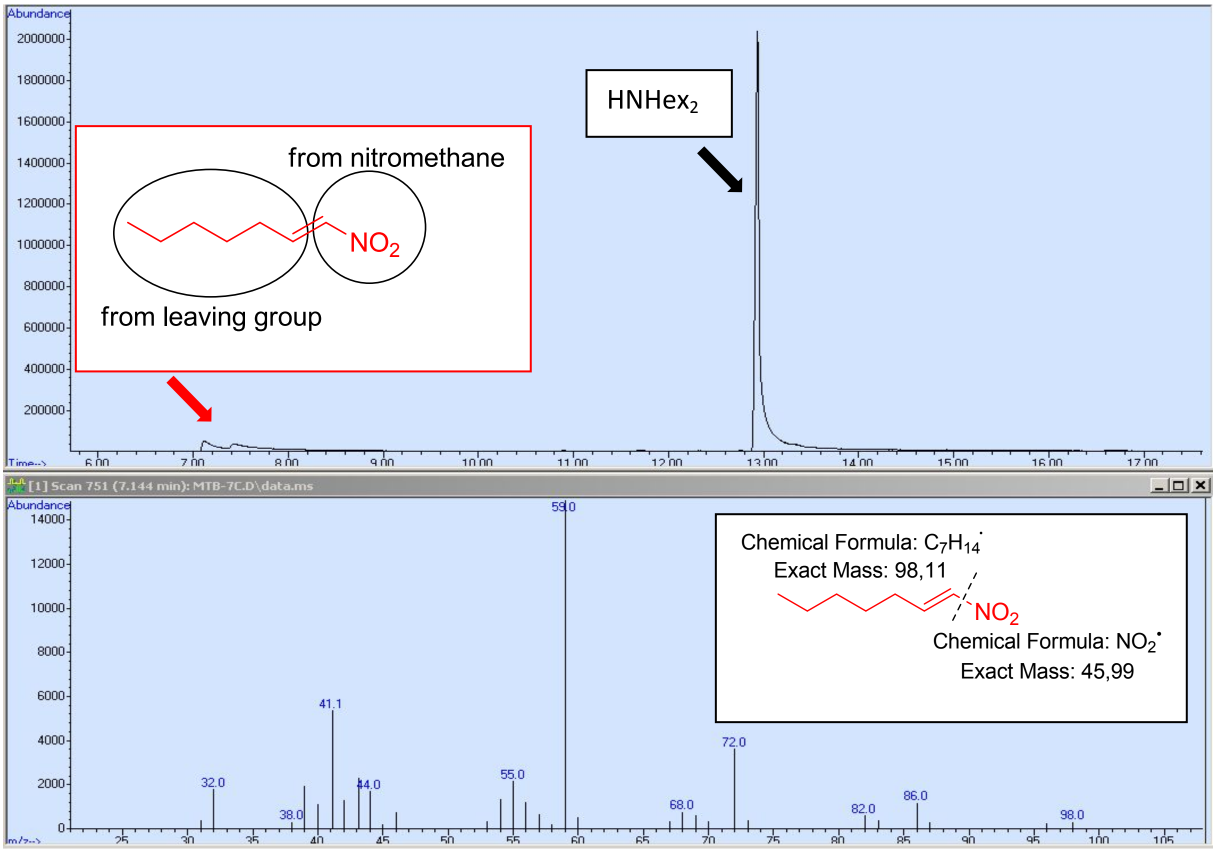

*Figure S26. GC-MS result for reaction with trihexylamine (**5**).*

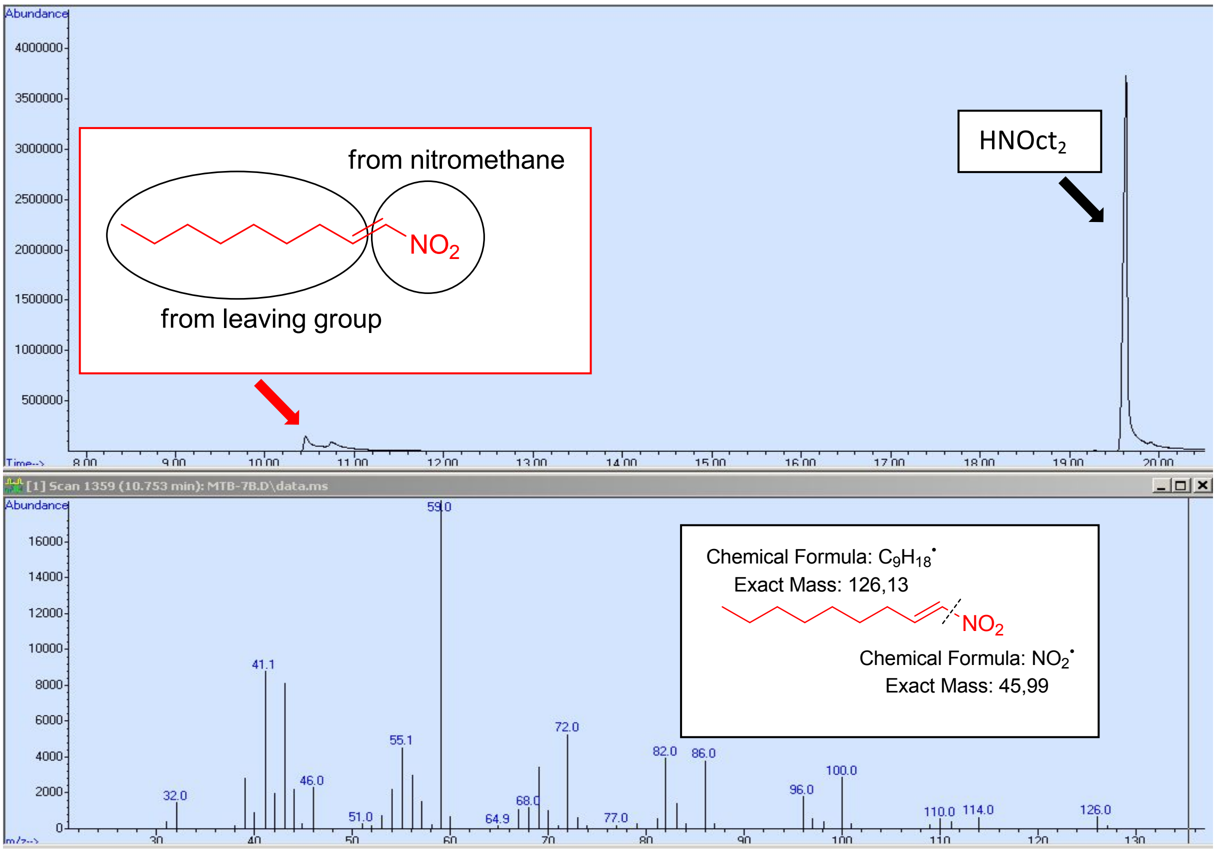

*Figure S27. GC-MS result for reaction with trioctylamine (**6**).*

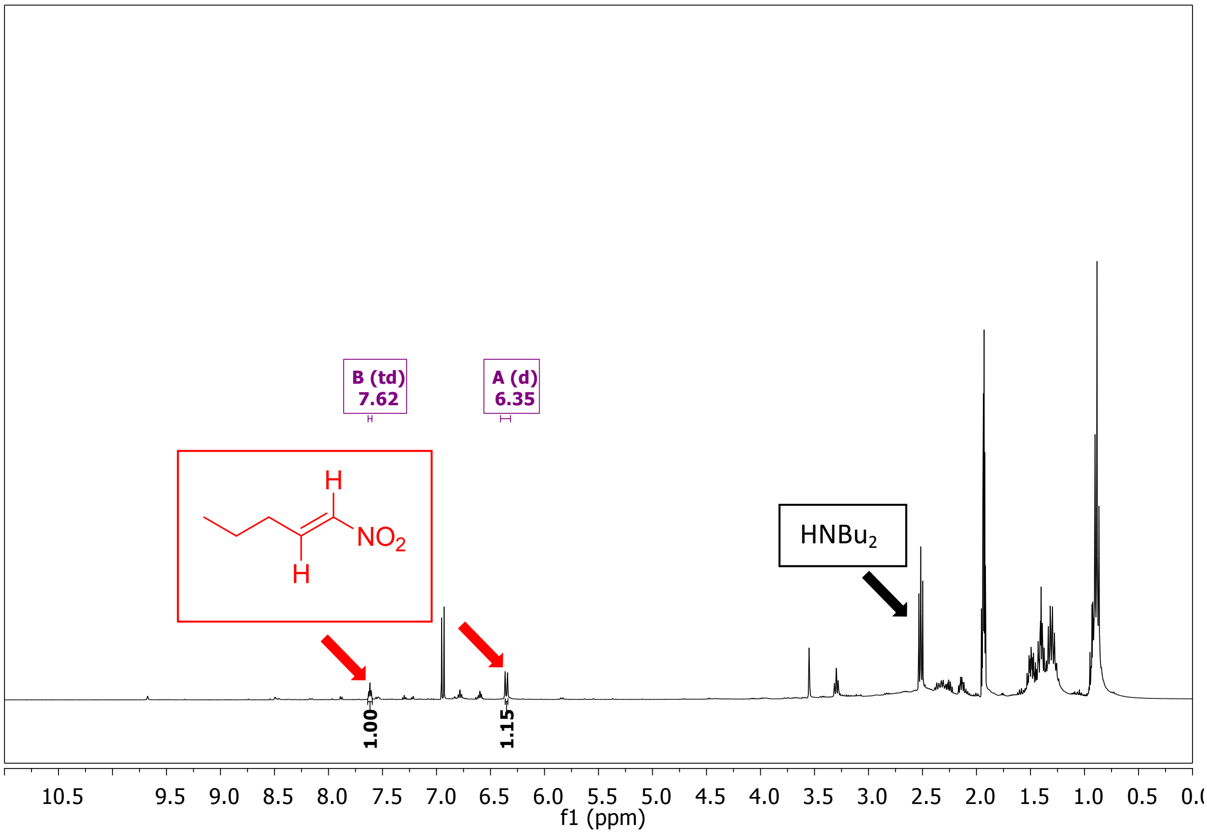

Figure S28. Direct ^{1}H-NMR spectrum of crude mixture after N-dealkylation reaction of tributylamine in D-MeCN.

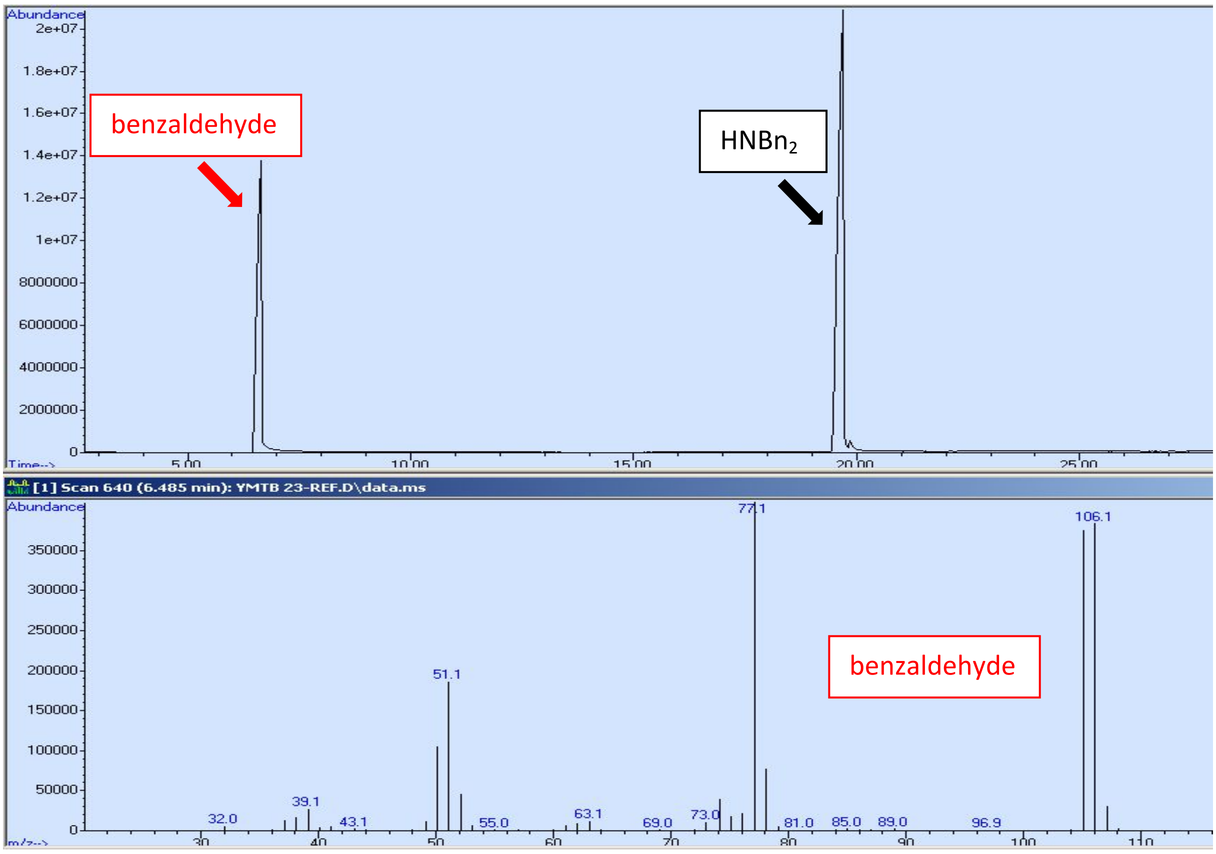

*Figure S29. GC-MS result for reaction with tribenzylamine (**9**).*
